# Electrochemical Glycosylation via Halogen-Atom-Transfer for *C*-Glycoside Assembly

**DOI:** 10.1021/acscatal.4c02322

**Published:** 2024-07-19

**Authors:** Jun Wu, Rajeshwaran Purushothaman, Felix Kallert, Simon L. Homölle, Lutz Ackermann

**Affiliations:** Wöhler-Research Institute for Sustainable Chemistry, Georg-August-Universität Göttingen, Tammannstraße 2, Göttingen 37077, Germany

**Keywords:** electrochemical halogen-atom-transfer, electrochemical C-glycosylation, α-aminoalkyl radical, electroreductive cross-electrophile glycosylation, glycosyl radical, C-glycosides

## Abstract

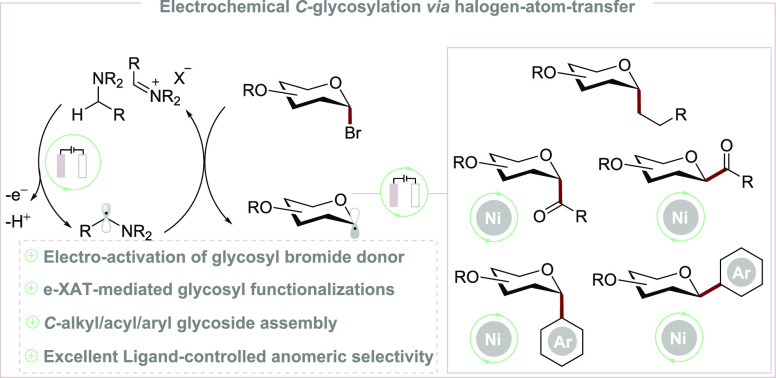

Glycosyl donor activation emerged as an enabling technology for anomeric functionalization, but aimed primarily at *O*-glycosylation. In contrast, we herein disclose mechanistically distinct electrochemical glycosyl bromide donor activations via halogen-atom transfer and anomeric *C*-glycosylation. The anomeric radical addition to alkenes led to *C*-alkyl glycoside synthesis under precious metal-free reaction conditions from readily available glycosyl bromides. The robustness of our e-XAT strategy was further mirrored by *C*-aryl and *C*-acyl glycosides assembly through nickela-electrocatalysis. Our approach provides an orthogonal strategy for glycosyl donor activation with expedient scope, hence representing a general method for direct *C*-glycosides assembly.

## 1 Introduction

*C*-glycosides represent a privileged carbohydrate motif in natural products and pharmaceutical compounds.^1^ The diverse biological functions led to a shift in focus from *O*-glycosides to more stable *C*-glycosides over the last decades and have thus revolutionized antiviral treatments, exemplified by the widespread use of *C*-ribosyl nucleoside prodrug, Remdesivir, in the early stage of COVID-19 infection.^2^ Additionally, the replacement of uridine with *C*-glycoside pseudo uridine led to the development of effective mRNA vaccines against COVID-19, culminating in the 2023 Nobel Prize for Physiology and Medicine for groundbreaking nucleoside base modification.^3^ Thus, selective glycosidic C–C bond construction methods are of significant value for enriching the viable *C*-glycoside motifs.^4^ Recent advances in metal-catalyzed cross-couplings,^5^ photochemistry,^6^ and C–H glycosylation^7^ provide transformative platforms for *C*-glycosylation. Despite indisputable advances, limitations, such as the tedious synthesis of glycosyl donors, expensive photocatalysts, and the use of stochiometric amounts of oxidants or reductants, remain to be considered for a sustainable transformation.

**Scheme 1:**
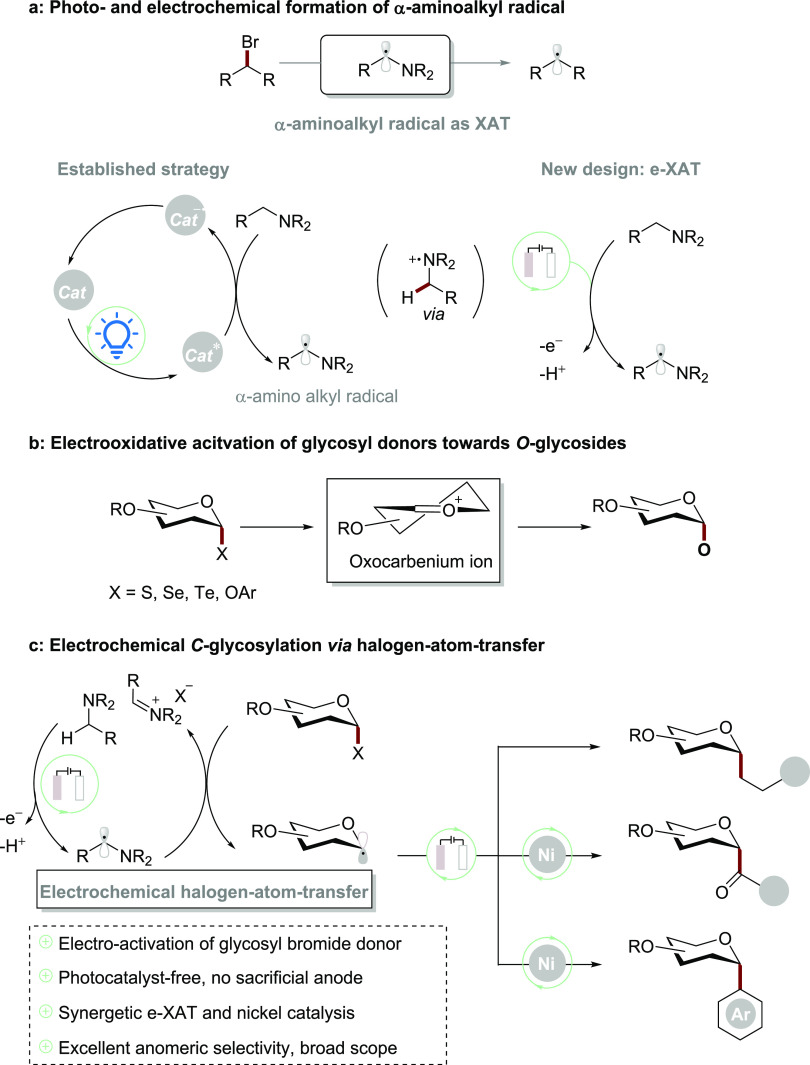
Halogen-Atom Transfer (XAT) and Electrochemical *C*-Glycosylation

In the recent decade, halogen-atom transfer (XAT) has gathered considerable attention,^8^ since it proved to be an efficient strategy to activate alkyl halides by exploiting, *inter alia*, nucleophilic α-amino alkyl radicals as XAT reagent, which can be formed through the single-electron (SET) oxidation of simple tertiary amines by excited photocatalysts (Scheme 1a).^9^ Notably, while the electrochemical anodic oxidation of tertiary amines is a known reaction, it is typically regarded as a sacrificial oxidation event aimed at preserving anodic electrodes in reductive electrochemical transformations (Scheme 1a).^10^ The effort to further explore the ability of α-aminoalkyl radicals as HAT reagents in electrochemistry remains rare.^11^

Electrochemical glycosylation, pioneered by Noyori and Kurimoto^12^ and Yoshida et al.,^13^ was established as a reliable method for glycosyl donor activation using thio-/seleno-/telluro-glycoside donors, particularly in synthesizing *O*-glycosides via oxocarbenium cation species (Scheme 1b).^14^ However, the electrochemical *C*-glycosylation has, to the best of our knowledge, thus far not been described. Within our program on *C*-glycosylation,^15^ and electrocatalysis,^16^ we questioned whether the electrochemically generated α-amino alkyl radical could serve as an *in situ* generated XAT reagent to enable glycosyl halide activation, affording glycosyl radical intermediate for subsequent glycosylations. This indeed may induce unprecedented reactivity profiles not achievable with oxocarbenium ion intermediates. Herein, we have devised an e-XAT-mediated anomeric addition for *C*-alkyl glycoside synthesis with the dual-functional Hünig base diisopropylethylamine (DIPEA) as the XAT reagent and anodic sacrificial reagent at exceptionally mild reaction conditions. Remarkably, the e-XAT process was likewise paired with nickel-electrocatalysis, enabling the assembly of *C*-aryl glycosides. Moreover, both anomers of *C*-aryl glycosides and *C*-acyl glycosides could be stereoselectively obtained through the judicious choice of the supporting ligand control^17^ (Scheme 1c).

## 2 Results and Discussion

Glycosyl halides represent user-friendly glycosyl donors^18^ and have been explored for anomeric Giese-addition with organotin hydrides via tin-radical initiated XAT process.^19^ In the pursuit of sustainable *C*-glycosylation, tin-free variants have been developed utilizing either photoredox catalysis^20^ or metal catalysis.^21^ In this context, we initially commenced our studies for the envisioned electrochemical anomeric addition of glycosyl donors with 4-vinyl-1,1′-biphenyl (**2aa**) to access *C*-alkyl glycoside (Table 1). Thus, glycosyl sulfones **1c**–**1f** were probed with a zinc plate as the anode and graphite felt (GF) as the cathode material, affording product **3a** either in a trace amount of product or low yields. Glycosyl bromide **1a** proved efficient to generate the desired product **3a** in 87% yield (Table 1, entry 1). By contrast, glycosyl chloride **1b** was an unsuitable glycosyl donor for electrochemical *C*-glycosylation. Notably, the byproducts, such as **3b**, were observed due to the electrochemical 2e^–^ reduction^22^ and the subsequent β-OAc-elimination. Next, we explored replacing the Zn anode with GF and screened different sacrificial amines, such as Et_3_N, DIPEA, and piperidine. DIPEA was identified as the best choice and thus avoided the use of the Zn plate as a sacrificial anode in undivided cell (Table 1, entries 2–4). Replacing GF anode with Pt delivered product **3a** in 63% yield (Table 1, entry 6). A slight modification greatly improved the yield to 89% with glycosyl bromide **1a** as the limiting reagent which helps to reduce the formation of byproduct **3b** (Table 1, entries 7–9). The control experiments verified the essential roles of the amine and the electricity (Table 1, entries 10 and 11).

**Table 1:**
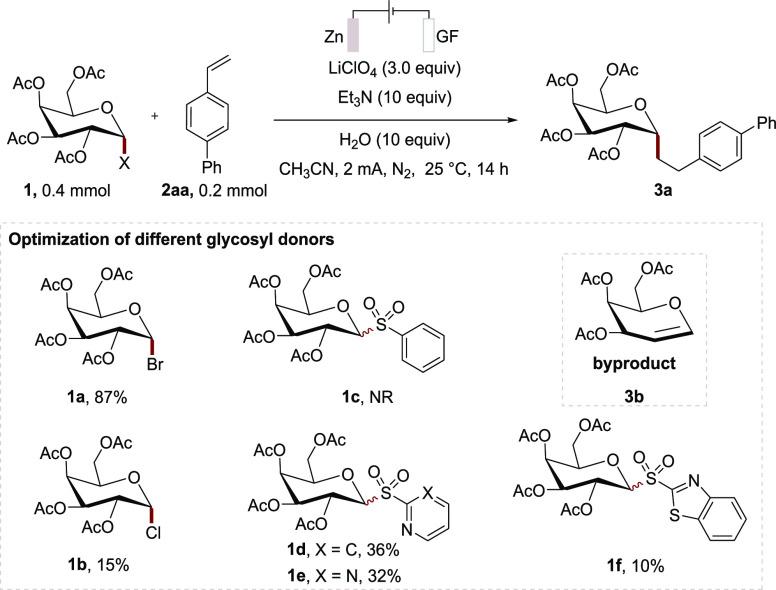
Optimization of the Reaction Conditions^a^

entry	derivatives	yields **3a**/**3b**%
1	none	87/5^a^^,^^b^
2	Et_3_N	20/62^c^^,^^e^
3	DIPEA	56/32^c^^,^^e^
4	piperidine	trace^c^^,^^e^
5	Et_3_N	32/53^d^^,^^e^
6	Pt/GF	63/26^d^^,^^e^
7	3.0 equiv of DIPEA	94/trace^b^^,^^d^
8	2.0 equiv of DIPEA	70/21^d^^,^^e^
9	1.5 equiv of **2aa**	89/trace^b^
10	without DIPEA	NR^e^
11	without current	NR^e^

Reaction conditions.

a **1a** (0.4 mmol), **2aa** (0.2 mmol), LiClO_4_ (0.6 mmol), DIPEA (0.6 mmol), H_2_O (20 mmol), CH_3_CN (4.0 mL) at 25 °C, 14 h under N_2_, Zn as anode, GF as cathode.

b Yield of isolated product.

c **1a** (0.4 mmol), **2aa** (0.2 mmol), LiClO_4_ (0.6 mmol), Et_3_N (2.0 mmol, 10 equiv), H_2_O (10 equiv), CH_3_CN (4.0 mL), GF as anode and GF as cathode.

d **1a** (0.4 mmol), **2aa** (0.2 mmol), LiClO_4_ (0.6 mmol), DIPEA (2.0 mmol, 10 equiv), H_2_O (10 equiv), CH_3_CN (4.0 mL), the Pt as anode, and GF as cathode.

e Yields were determined by NMR with 1,3,5 trimethoxybenzene as the standard.

**Scheme 2:**
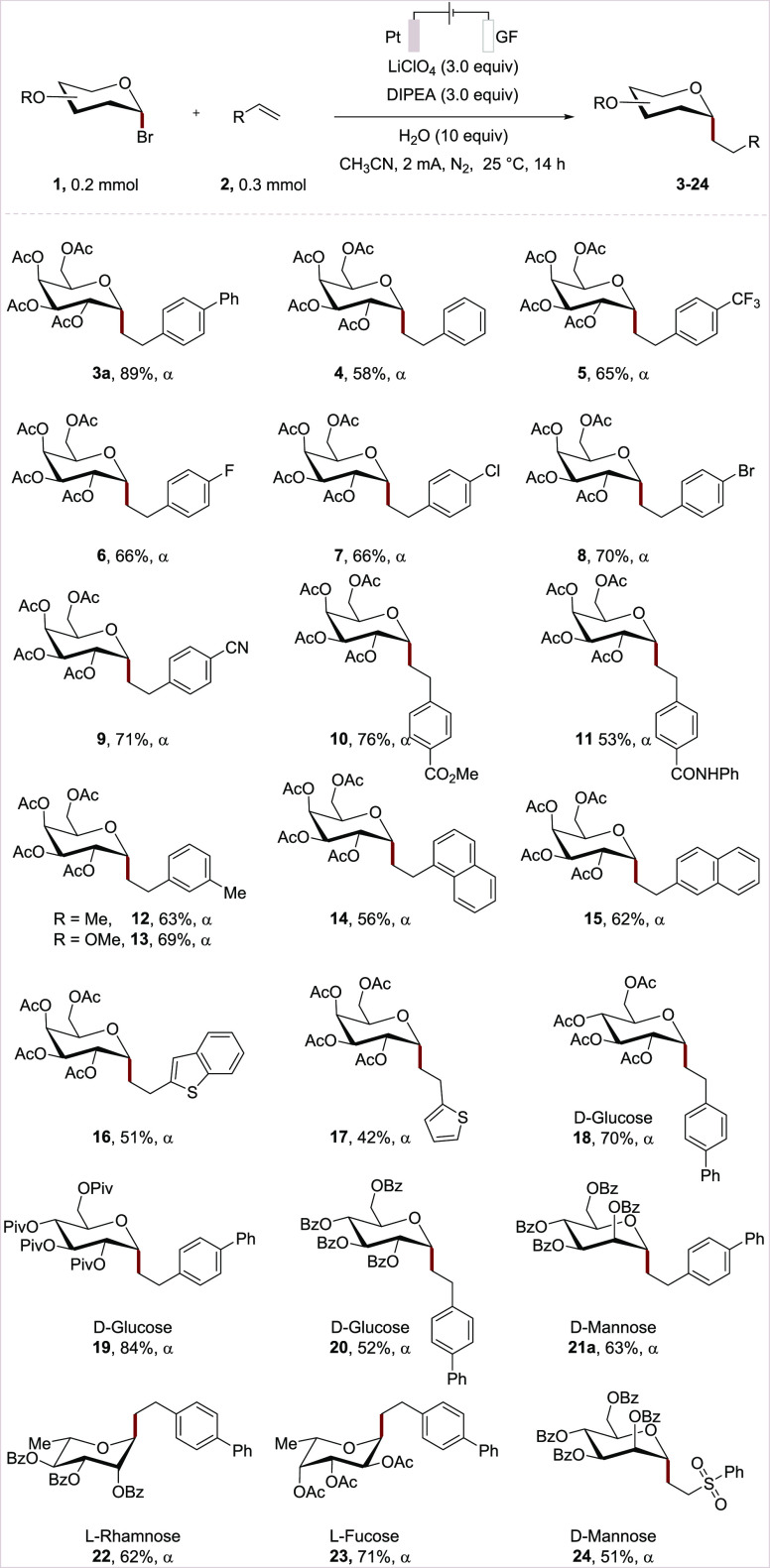
Scope of the Alkenes and Glycosyl Bromides

With the optimized reaction conditions for the e-XAT-mediated *C*-alkyl glycosides synthesis in hand, we examined the generality of the electrochemical anomeric addition (Scheme 2). Initially, substituents on the arene were tested, showing that functional groups, such as fluoro (**2ad**), chloro (**2ae**), bromo (**2af**), cyanide (**2ag**), ester (**2ah**), and amide (**2ai**), were compatible under electrocatalysis (**3**–**11**). Electro-donating substituents at the *meta*-position were also feasible (**12** and **13**). Vinyl naphthalene **2al** and **2am** gave the products **14** and **15** in moderate yields. Notably, the reaction with heteroarenes **2an** and **2ao** was performed to afford *cis*-*C*-alkyl glycosides **16** and **17b**. Further examinations indicated that electrochemical radical *C*-glycosylation was tolerated with a series of glycosyl bromides. Glucosyl bromide **1h**–**1i** with different *O*-groups, such as acetyl, pivaloyl, and benzoyl groups, were amenable (**18**–**20**). Similarly, different glycosyl donors, such as mannosyl bromide **1g**, rhamnosyl bromide **1k**, and fucosyl bromide **1l**, resulted in the formation of corresponding *C-*alkyl glycosides with exclusive anomeric selectivity (**21a–23**). In addition, activated alkene (vinyl sulfonyl) benzene **2ap** was also explored, leading to the formation of *C*-glycoside **24** in 51% yield.

**Scheme 3:**
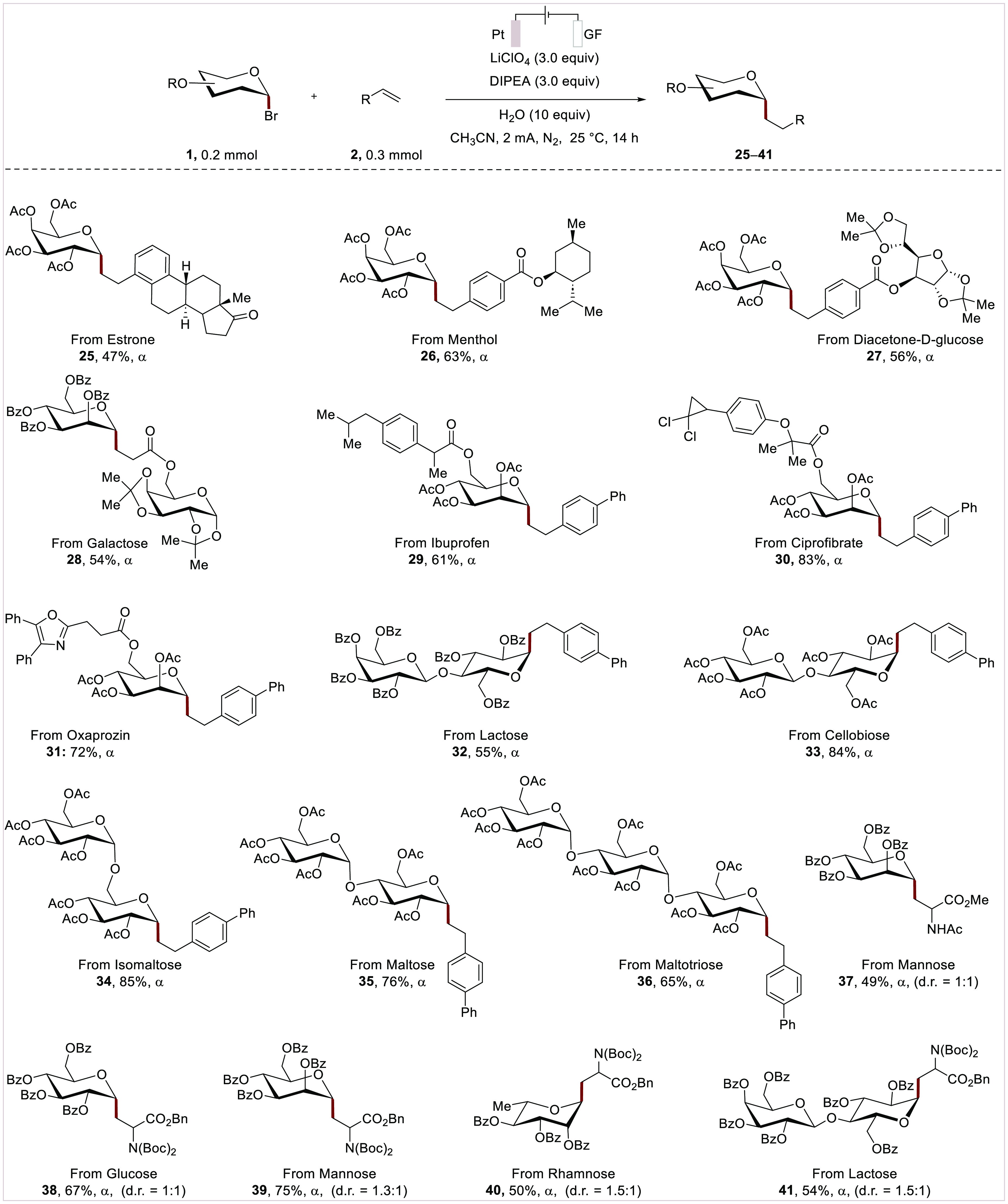
Scope of the Electrochemical Glycoconjugation

To demonstrate the applicability of the electrochemical anomeric addition reaction (Scheme 3), structurally complex alkenes **2aq**–**2as** were examined for direct late-stage modification, giving products **25**–**27**. The reaction with acrylate **2at** bearing a galactose moiety enabled the *C*-disaccharide assembly (**28**). When hybrid glycosyl bromide donors bearing natural products and drug derivatives, such as ibuprofen **1m**, ciprofibrate **1n**, and oxaprozin **1o**, were employed as the substrates, highly functionalized glycoconjugates (**29**–**31**) were selectively and efficiently obtained. Furthermore, disaccharides, including lactose **1p**, cellobiose **1q**, isomaltose **1r**, and maltose **1s**, proved suitable for versatile *C*-glycosides assembly in a single reaction (**32**–**35**). The robustness of our e-XAT protocol was mirrored by the maltotriosyl radical addition, yielding product **36** in 65% yield. Additionally, the glycosyl radical with the dehydroalanine moiety gave glycosylated-amino acid (**37**–**41)**

Encouraged by the success of our e-XAT for *C-*alkyl glycoside synthesis, we wondered whether the merger of the anodic e-XAT process with cathodic reductive nickel catalysis would indeed set the stage for the assembly of synthetically relevant *C*-aryl glycoside. This paired nickel-catalyzed reductive electrocatalysis would thereby offer a sustainable solution to address the current limitations of metal-catalyzed reductive *C*-glycosylation, such as the use of external stochiometric metal reductants, expensive photocatalysts in metalla-photoredox catalysis, sacrificial anode materials, or difficult to access glycosyl donors. Moreover, this paired electrocatalysis represents a conceptually distinct strategy for halogen-atom transfer in molecular synthesis.

**Scheme 4:**
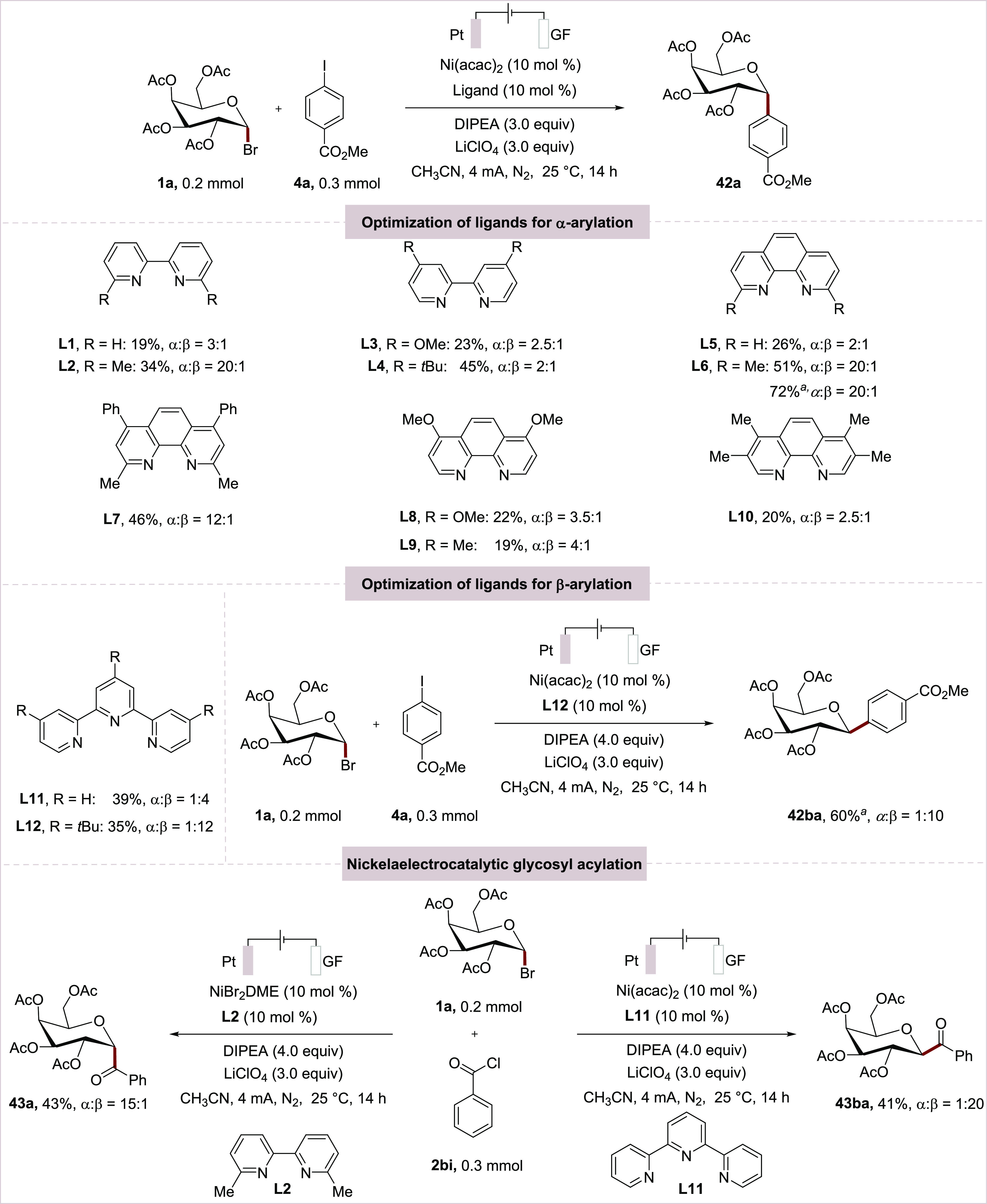
Optimization of Nickelaelectro-Catalyzed Reductive Glycosyl Functionalization Reaction conditions: **1a** (0.2 mmol), electrophiles (0.3 mmol), Ni catalyst (10 mol %), Ligand (10 mol %), LiClO_4_ (0.6 mmol), DIPEA (0.6 mmol), CH_3_CN (4.0 mL) at 25 °C, 14 h under N_2_, Pt as anode, GF as cathode. ^*a*^Using 4.0 equiv of DIPEA.

To this end, we probed the envisioned e-XAT reductive cross-electrophile coupling^23^ using galactosyl bromide **1a** and methyl 4-iodobenzoate **2ba** with the aid of nickelaelectro-catalysis (Scheme 4).

The desired product **42a** was obtained in 19% yield, albeit with a low anomeric selectivity (α: β = 3:1) with Ni(acac)_2_ as catalyst, bipyridine **L1** as ligand, and DIPEA serving both as XAT-agent and reductant in an undivided cell. Next, a set of bidentate bipyridine ligands **L2**–**L4** were tested and revealed that the substituents at the *para*-position can considerably alter the yield (**L3** and **L4**). With a substituent at the *ortho*-position (**L2**) the α-anomeric arylation product was selectively formed. A similar substituent effect was observed when employing phenanthroline ligands. Also, here the ligand-bearing C2-substituents outperformed ligands with groups at the C3- or C4-position (**L5**-**L10**) in terms of reactivity and selectivity. Notably, the ligand **L6** gave the desired product in 51% yield with an excellent α:β ratio of 20:1. A slight increase in the amount of DIPEA resulted in a further improved yield of 72%, notably without loss of anomeric selectivity. Interestingly, this selectivity could be switched to anomeric β-arylation product **42ba**, when exploiting the tridentate ligands **L11** and **L12**. Here, **L12** afforded the desired product **42ba** with a moderate yield and excellent anomeric selectivity. The yield could be further improved with increased amounts of the Hünig base. Remarkably, this e-XAT-mediated nickel-electrocatalysis could be transferred to glycosyl acylation using commercially available benzoyl chloride as the acylating reagent. The anomeric selectivity could again be controlled by the judicious choice of the ligand. Hence, **L2** afforded the *C*-acyl glycosides **43a** with a high level of α-selectivity, while the β-acylation product **43ba** was obtained with tridentate ligand **L11**. Thus, our strategy provides a stereodivergent, and sustainable assembly of *C*-acyl-glycosides,^24^ thereby avoiding rather difficult-to-access glycosyl donors.^25^

**Scheme 5:**
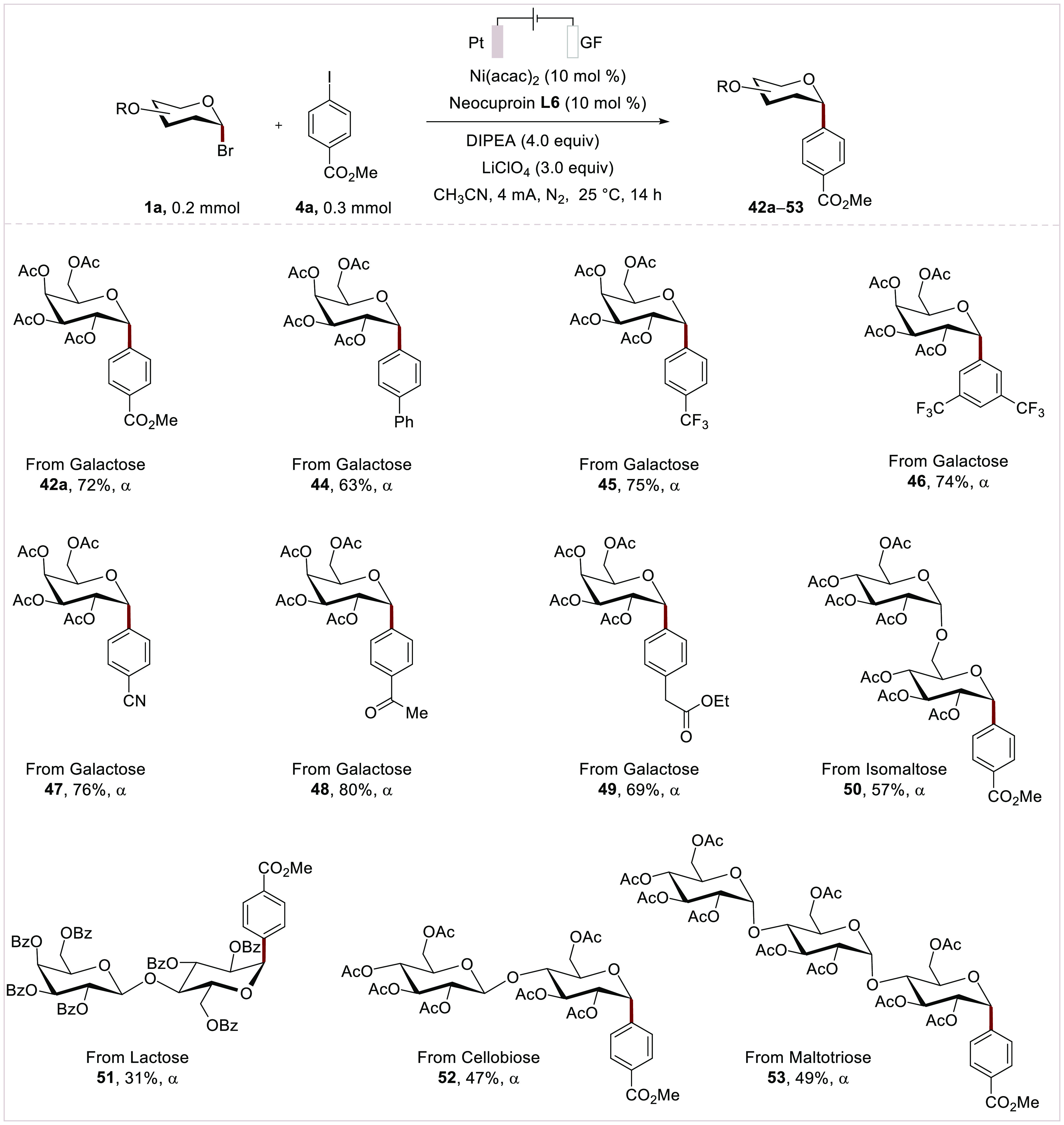
Scope of Nickelaelectro-Catalyzed Glycosyl α-Arylation

Then, the robustness of our nickelaelectro-catalyzed reductive glycosyl arylation was probed with a diverse array of substrates featuring functional groups, such as ester (**42a** and **49**), and trifluoromethyl (**45** and **46**). Likewise, cyano (**47**) and ketone (**48**) groups were well tolerated in the nickelaelectro-catalyzed cross-electrophile coupling. Furthermore, structurally complex glycosyl bromides, such as isomaltose, lactose, cellobiose, and maltotriose, were arylated with excellent α-selectivities (**50**-**53**) (Scheme 5).

**Scheme 6:**
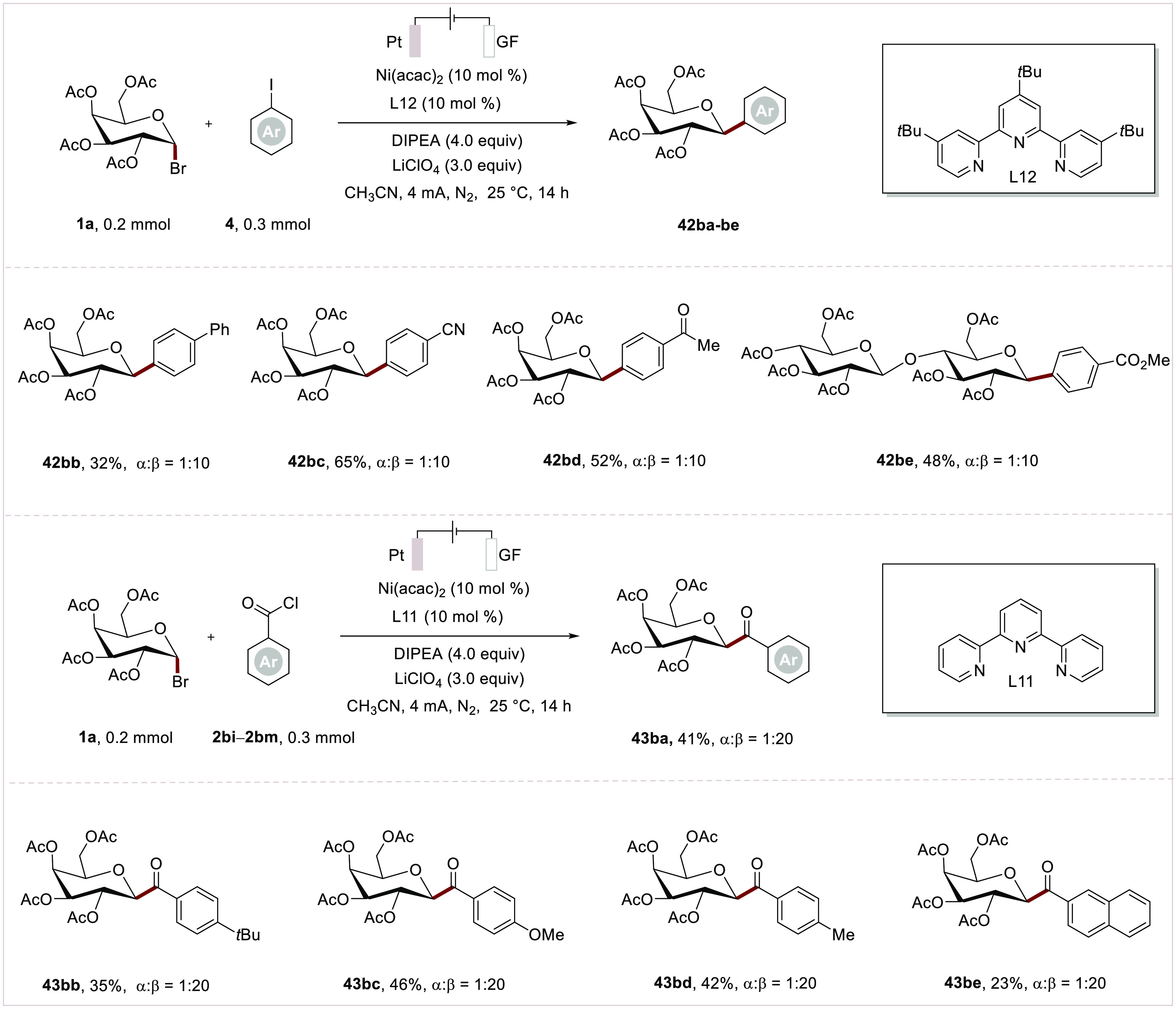
Scope of Nickelaelectro-Catalyzed Glycosyl β-Arylation and β-Acylation

Of particular note, our nickelaelectrocatalyzed C-glycosylation offers stereodivergent access to both α- and β-isomers of C-aryl glycosides and C-acyl glycosides by nickel catalyst with slightly different tridentate terpyridine ligands (Scheme 6). The terpyridine/Nickel catalytic system outcompetes the inherent stereoelectronic effects of α-isomer due to the steric hindrance of C2 substituents with axial ligated nickel complex, leading to exclusive β-isomers. The scope of nickelaelectrocatalytic β-C-aryl glycosylation was explored, and excellent anomeric selectivities and functional group tolerance were observed (**42ba**–**42be**). Notably, the β-acylation, which has not been reported, likewise demonstrated feasible albeit with moderate yields with galactosyl bromide (**43ba**–**43be**).

**Scheme 7:**
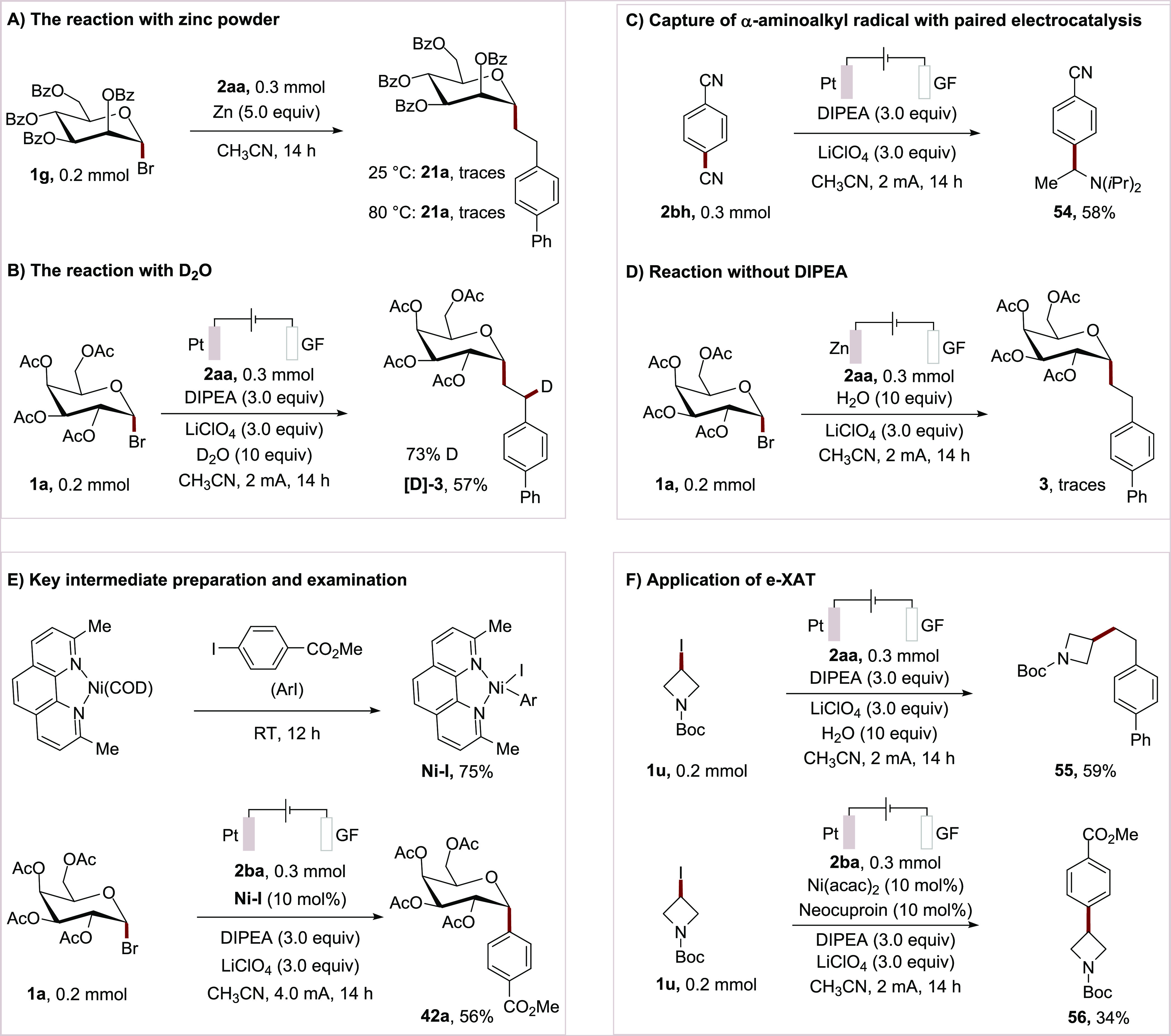
Mechanistic Studies

To gain insights into the working mode of the e-XAT-mediated *C*-glycosylation, the formal electroreductive *C*-alkyl glycoside synthesis was initially probed using stoichiometric zinc powder as a reductant. The failure to form the Giese-addition product **21a** suggested that single-electron reduction of glycosyl bromide **1g** was not operative (Scheme 7A).^26^ Furthermore, a deuterium labeling experiment yielded the product **[D]-3** with 70% deuterium incorporation at the benzylic position, being suggestive of a cathodic reduction of the benzylic radical to the corresponding anion to be involved(Scheme 7B). To elucidate the e-XAT process, a paired electrolysis was attempted to trap the α-amino alkyl radical. With dicyanobenzene **2bh** as a cathodic reducing reagent product **54** was isolated in 58% yield, highlighting a formed α-amino alkyl radical through the anodic oxidation of the Hünig base (*E*_ox_ = 0.86 V) (Scheme 7C).^27^ A control experiment without the Hünig base resulted only in trace amounts of the product, further supporting the key role of the α-amino alkyl radical in the e-XAT process (Scheme 7D). Then, the **Ni–I** complex was prepared from the facile oxidative addition.^28^ A catalytic amount of **Ni–I** complex was employed in the electroreductive cross-electrophile coupling reaction, and a comparable yield of 56% was observed (Scheme 7E). These findings support that a nickel(0/II) manifold could be catalytically relevant.

To further assess the versatility of our e-XAT strategy, we investigated the reductive arylation of *tert*-butyl 3-iodoazetidine-1-carboxylate (**1u**), resulting in the formation of product **55** in 59% yield. Likewise, methyl 4-iodobenzoate (**2ba**) was identified as a viable substrate for the e-XAT cross electrophile coupling, indicating the enabling potential of our strategy beyond glycosyl bromide electrophiles (Scheme 7F).

**Scheme 8:**
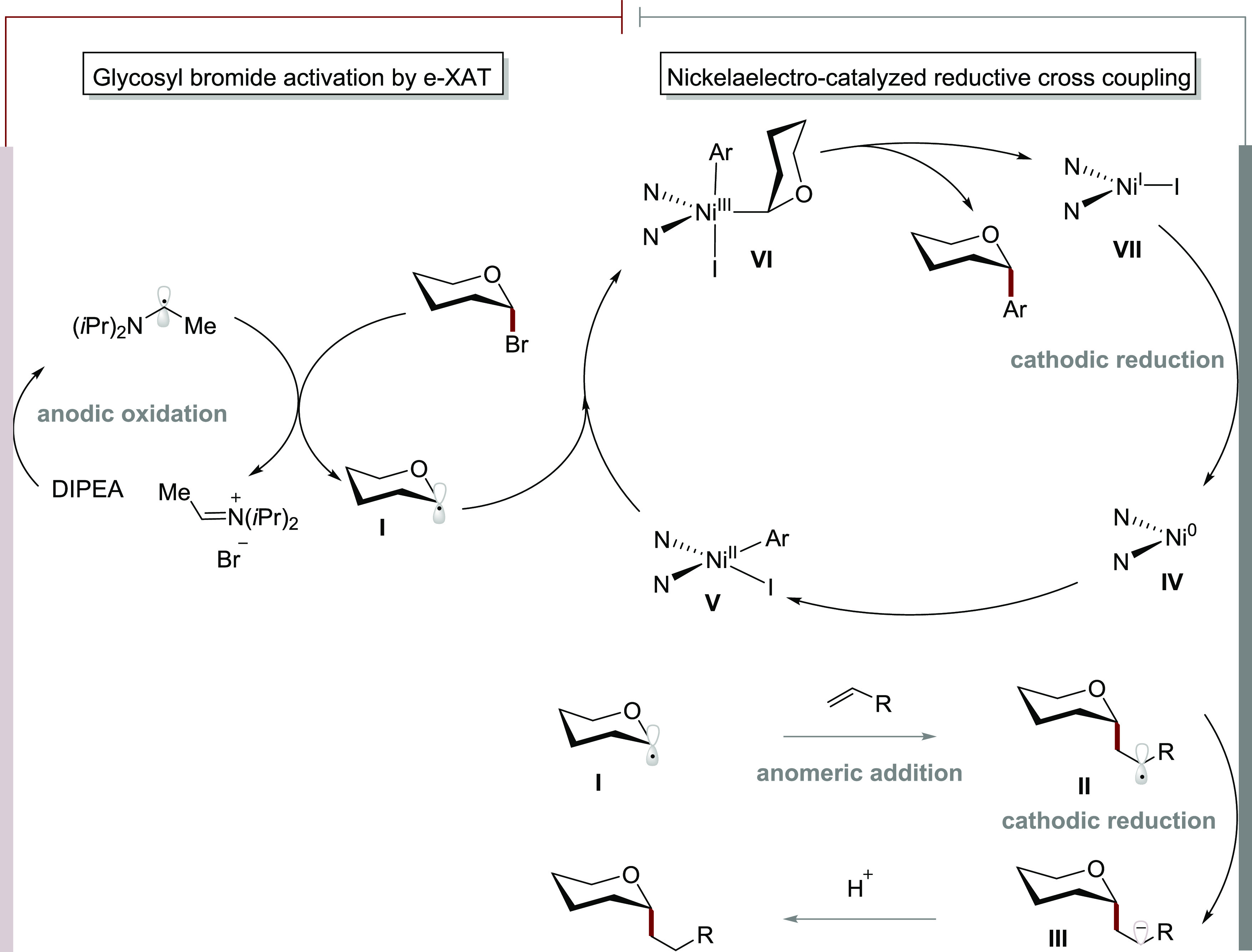
Plausible Catalytic Cycle

Based on the above-mentioned investigations, a plausible mechanism for our electrochemical *C*-glycosylation was suggested (Scheme 8). Initially, anodic oxidation generates an α-amino alkyl radical of the Hünig base. Subsequently, XAT occurs via homolytic C–Br cleavage, generating glycosyl radical **I**. The *C*-alkyl glycoside product is then formed by anomeric radical addition, cathodic reduction, and final protonation.

In contrast, when merging this manifold with nickel catalysis, a plausible catalytic cycle for nickelaelectro-catalyzed reductive glycosyl arylation proceeds via a nickel(0/II/III/I) pathway, with initial oxidative addition of the aryl iodide onto nickel(0) **IV** to nickel(II) **V**. In the meantime, the e-XAT generates the persistent glycosyl radical **I**, which in turn delivers nickel(III) species **VI**. Finally, reductive elimination from nickel(III) **VI** provides the desired *C*-aryl glycosides, while the thus-obtained nickel(I) species **VII** undergoes cathodic reduction.

## 3 Conclusions

In conclusion, we have reported on an efficient and selective electrochemical *C*-glycosylation, providing a platform for the assembly of diverse *C*-alkyl glycosides and structurally complex *C*-glycoconjugates. The photocatalyst-free e-XAT strategy was not limited to radical conjugate additions. Indeed, paired electrocatalysis by nickelaelectro-catalysis enabled glycosyl anomeric arylation and acylation. This merger of e-XAT with reductive nickel catalysis reflects the outstanding versatility of our e-XAT approach for *C-*aryl and *C*-acyl glycoside synthesis. It is noteworthy, that the judicious choice of the supporting ligand allowed here for full selectivity control at the anomeric center.

## Supporting Information Available

Experimental procedures, characterization, and spectral data (PDF)
