# Supplementary material for: Electrochemical Glycosylation via Halogen-Atom-Transfer for C-Glycoside Assembly
Source: ACS Catal. 2024 Jul 19;14(15):11532–44. doi: 10.1021/acscatal.4c02322 (PMC11301629; doi:10.1021/acscatal.4c02322)

Supporting Information

Electrochemical Glycosylation *via* Halogen-Atom-Transfer for *C*−Glycosides Assembly

Jun Wu+, [a] Rajeshwaran Purushothaman+,[a] Felix Kallert[a] Simon L. Homölle[a] and Lutz Ackermann*[a]

[a] Institut für Organische und Biomolekulare Chemie Georg-August-Universität

Tammannstraße 2, 37077 Göttingen, Germany

Email : Lutz.Ackermann@chemie.uni-goettingen.de

Table of Contents

General Remarks **S3**

Optimization of Reaction Condition **S4**

General Procedures **S12**

Characterization Data of Products **S15**

Mechanistic Studies **S81**

References **S89**

^{1}H- and ^{13}C-NMR Spectra **S90**

General Remarks

Catalytic reactions were performed under an N_2 atmosphere using pre-dried glassware and standard Schlenk techniques. Glycoside bromides were synthesized according to the literature.[1] Dehydroalanines were synthesized according to the literature.[2] 1,4-Dioxane was dried over Na and freshly distilled under N_2. Other chemicals were obtained from commercial sources and were used without further purification. Yields refer to isolated compounds mentioned in (Table S1) estimated to be >98% pure as determined by ^{1}H NMR and other yields are determined by NMR with 1,3,5 trimethoxy benzene as a standard reference. The α/β ratios are determined by NMR and both isomers are inseparable by column chromatography. Flash chromatography: Merck silica gel 60 (40– 63 μm). NMR: Spectra were recorded on a Varian Inova 500, Varian Inova 600, Bruker Avance III 400, Bruker Avance III HD 400, and a Bruker Avance III HD 500 instrument in the solvent indicated; chemical shifts (δ) are provided in ppm. IR spectra were recorded on a Bruker FT-IR alpha-P device. EI-MS was recorded on Joel AccuTof at 70 eV. ESI-MS was recorded on Bruker Daltonic micrOTOF. High-resolution mass spectrometry (HR-MS) was recorded on micrOTOF, Bruker Daltonic.

Optimization of the Reaction Condition

Table S1. Optimization of electrochemical *C*-alkyl glycosides synthesis.[a]

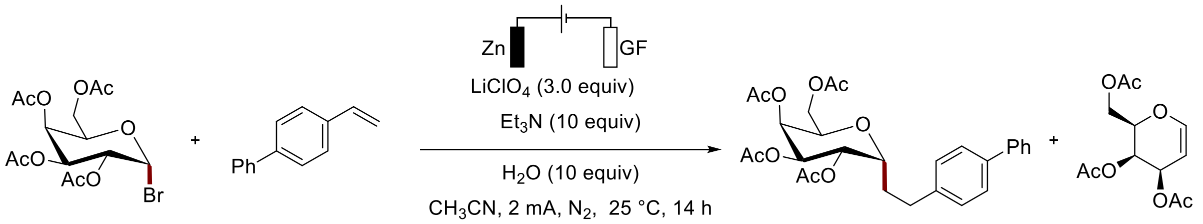

1a, (0.4 mmol) **2aa, (**0.2 mmol) **3a** **3b**

Entry	Deviations from the standard conditions	Yields% **(3a/3b)**
1	None	87/05[b]
2	Et_3N	20/62[ce]
3	DIPEA	56/32[ce]
4	Piperidine	Trace[ce]
5	Et_3N	32/53[de]
6	Pt/GF	63/26[de]
7	3.0 equiv of DIPEA	94/trace[bd]
8	2.0 equiv. of DIPEA	70/21[de]
9	1.5 equiv of **2aa**	89/trace[b]
10	Without DIPEA	NR[e]
11	Without current	NR[e]

[a] Reaction conditions: **1a** (0.4 mmol), **2aa** (0.2 mmol**),** $LiClO_4$ (0.6 mmol), DIPEA (0.6 mmol), H_2O (20 mmol), CH_3CN (4.0 mL) at 25 ℃, 14 h under N_2, Zn as anode, GF as cathode. [b] Yield of isolated product. [c] **1a** (0.4 mmol), **2aa** (0.2 mmol), $LiClO_4$ (0.6 mmol), NEt_3 (2.0 mmol, 10 equiv), H_2O (10 equiv), CH_3CN (4.0 mL), GF as anode and GF as cathode. [d] **1a** (0.4 mmol), **2aa** (0.2 mmol), $LiClO_4$ (0.6 mmol), DIPEA (2.0 mmol, 10 equiv), H_2O (10 equiv), CH_3CN (4.0 mL) Pt as anode, and GF as cathode. [e] The Yield and α-/β-ratio are quantified by NMR with 1,3,5 trimethoxy benzene as a standard reference.

Table S2. Optimization of different electrolytes

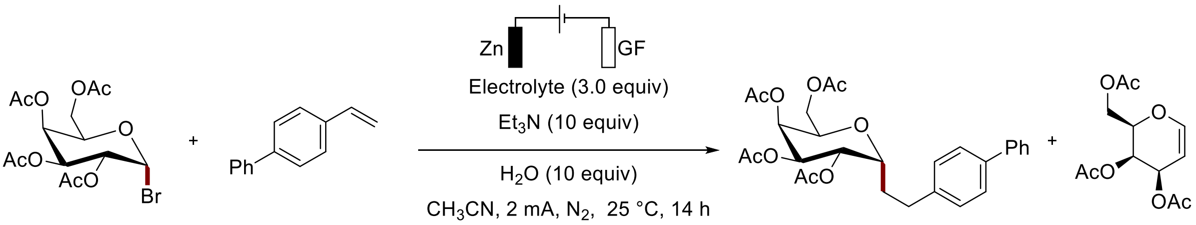

1a, (0.4 mmol) **2aa**, (0.2 mmol) **3a** **3b**

Entry	Electrolytes	Yields% (**3a**/**3b**)
1	*n*Bu_4NI	40:12
2	*n*Bu_4NBr	59:03
3	*n*Bu_4NCl	21: 34
4	*n*Bu_4NPF_6	76:11
5	*n*Bu_4NBF_4	69:15
6	*n*NBu_4NO_3	NR

Table S3. Optimization of different solvents

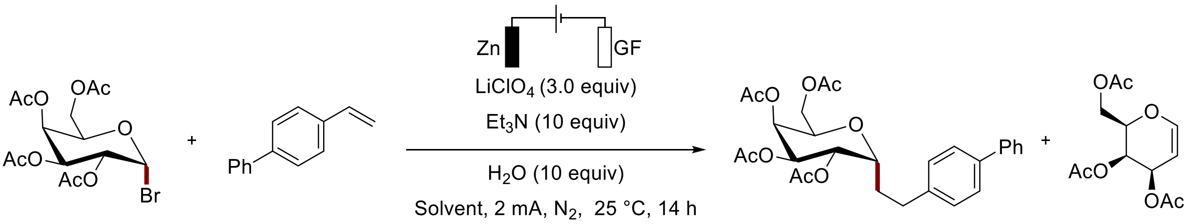

1a, (0.4 mmol) **2aa**, (0.2 mmol) **3a** **3b**

Entry	Solvents	Yields% (**3a**/**3b**)
1	H_2O	NR
2	DMF	10:32
3	Dioxane	NR
4	THF	trace
5	DMSO	NR
6	MeOH	NR

Table S4. Optimization of different amines

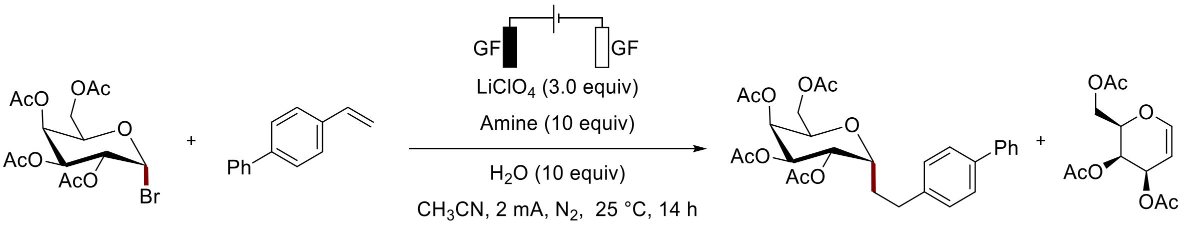

1a, (0.4 mmol) **2aa**, (0.2 mmol) **3a** **3b**

Entry	Amines	Yields% (**3a**/**3b**)
1	Et_3N	20:62
2	DIPEA	56:29
3	Piperidine	NR
4	Pyrrolidine	trace
5	Piperazine	NR
6	TMG	NR

DIPEA: *N*-ethyl-*N*-isopropylpropan-2-amine; TMG: 1,1,3,3-Tetramethylguanidine

Table S5. Optimization of different electrodes

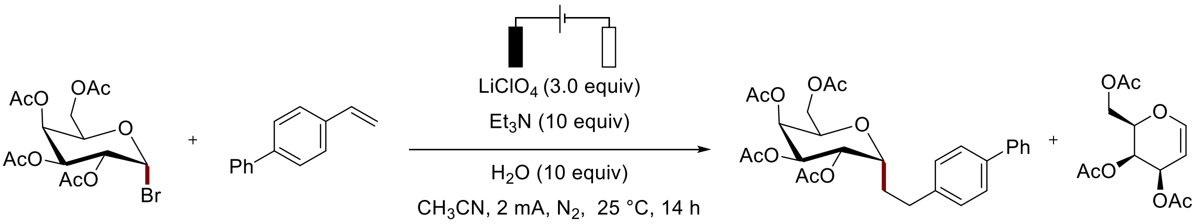

1a, (0.4 mmol) **2aa**, (0.2 mmol) **3a** **3b**

Entry	Electrodes	Yields% (**3a**/**3b**)
1	GF/Pt	NR
2	Pt/GF	65:34
3	Pt/Pt	NR
4	C/Pt	NR
5	GF/C	NR
6	Pt/C	NR

Table S6. Optimization of different equivalents of DIPEA

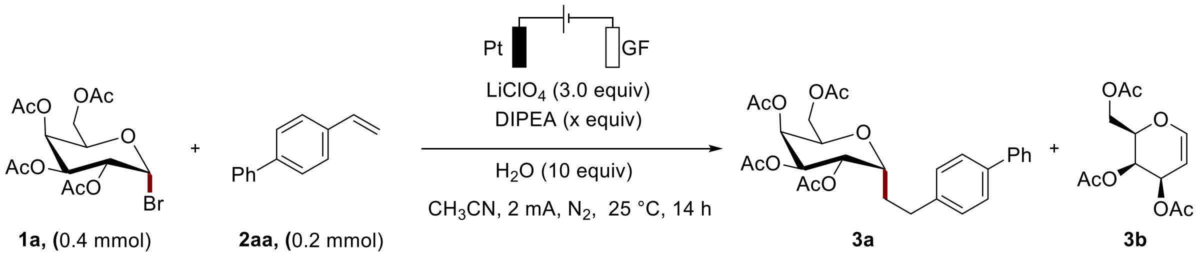

Entry	Equiv of amine	Yields% (**3a**/**3b**)
1	1 equiv	26:64
2	2 equiv	70:21
3	3 equiv	94: trace
4	4 equiv	32:51

Table S7. Optimization of different equivalents of glycosyl bromide

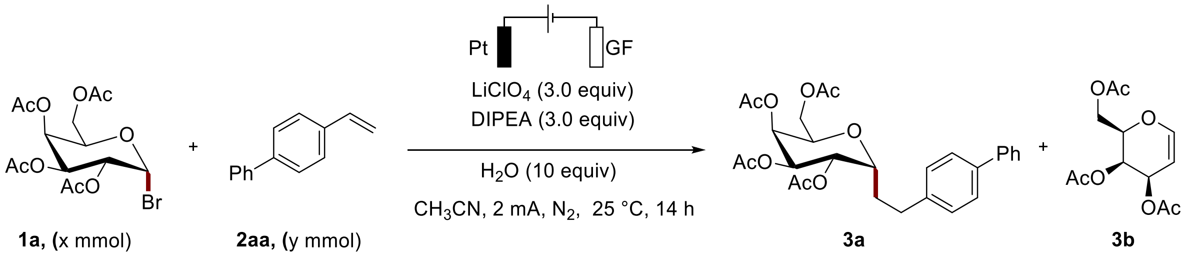

Entry	x:y	Yields% (**3a**/**3b**)
1	1:1	69:21
2	1:2	62:34
3	1:1.5	89: trace

Table S8: Optimization of glycosyl donors

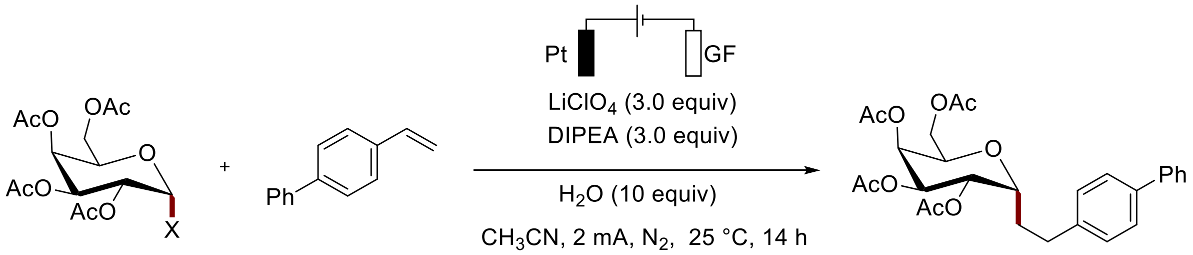

1a, (0.3 mmol) **2aa,** (0.2 mmol) **3a**

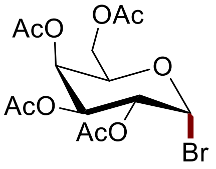

1a, 89%

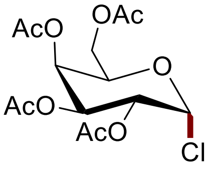

1b, 15%

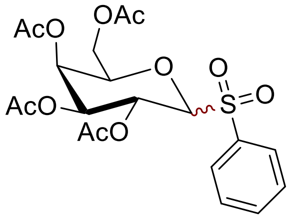

1c, NR

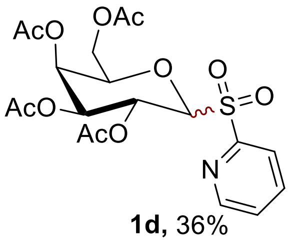

1d, 36%

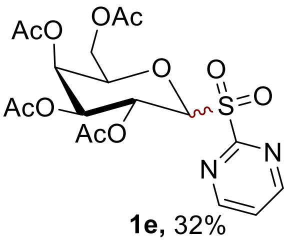

1e, 32%

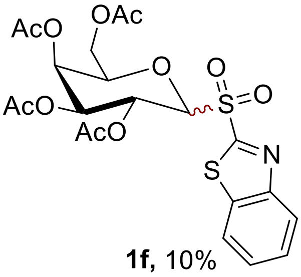

1f, 10%

Table S9: Optimization of ligands for nickelaeletrocatalytic glycosyl arylation

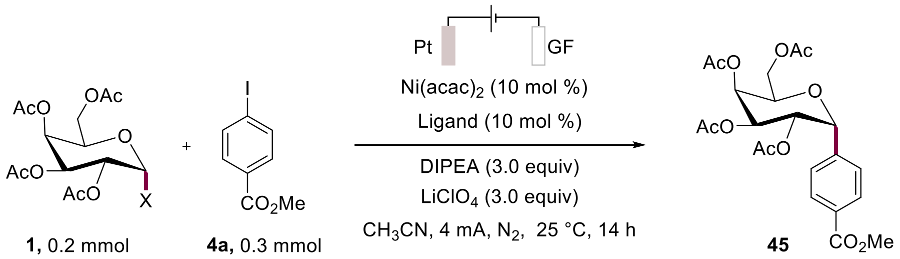

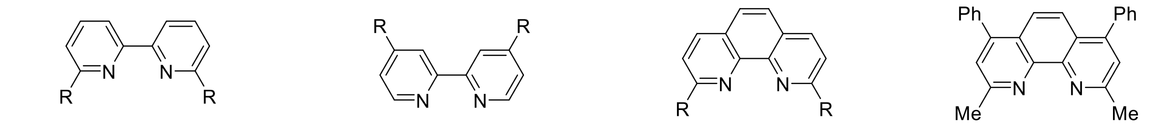

L1, R = H: 19%, α:β = 3:1
L2, R = Me: 34%, α:β = 20:1

L3, R = OMe: 23%, α:β = 2.5:1
L4, R = *t*Bu: 45%, α:β = 2:1

L5, R = H: 26%, α:β = 2:1
L6, R = Me: 51%, α:β = 20:1
72%[a], α:β = 20:1

L7, 46%, α:β = 12:1

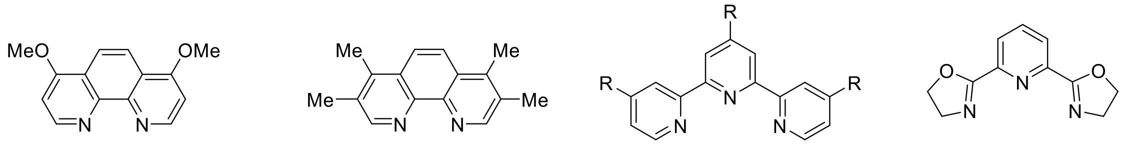

L8, R = OMe: 22%, α:β = 3.5:1
L9, R = Me: 19%, α:β = 4:1

L10, 20%, α:β = 2.5:1

L11, R = H: 39%, α:β = 1:4
L12, R = *t*Bu: 35%, α:β = 1:12

L13, 46%, α:β = 1.5:1

The yields and α-/β-ratio are quantified by NMR with 1,3,5 trimethoxy benzene as a standard reference.

Scope of olefins

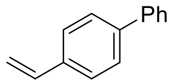

2aa

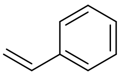

2ab

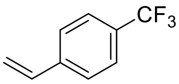

2ac

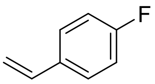

2ad

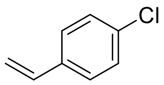

2ae

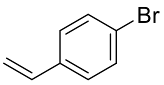

2af

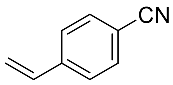

2ag

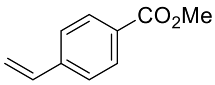

2ah

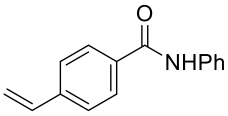

2ai

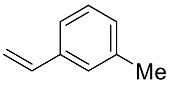

2aj

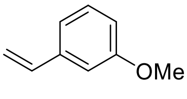

2ak

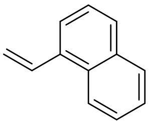

2al

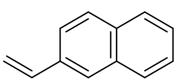

2am

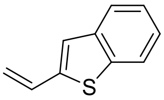

2an

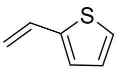

2ao

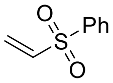

2ap

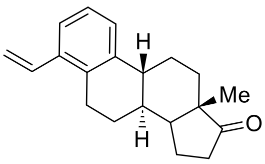

2aq

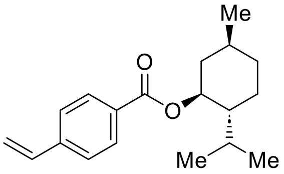

2ar

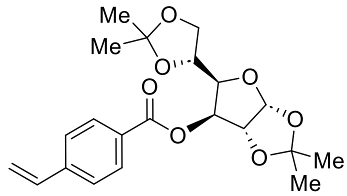

2as

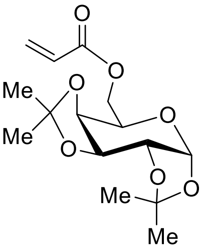

2at

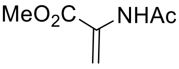

2au

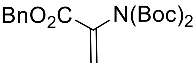

2av

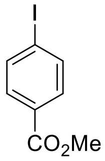

2ba

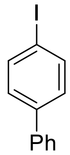

2bb

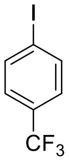

2bc

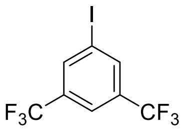

2bd

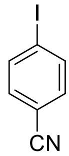

2be

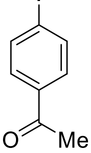

2bf

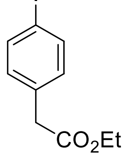

2bg

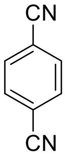

2bh

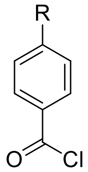

R= H, 2bi
R= tBu, 2bj
R= Me, 2bk
R= MeO, 2bl
R= 2-Nap,2bm

Scope of glycosyl donors

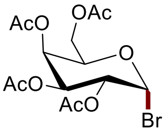

1a

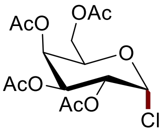

1b

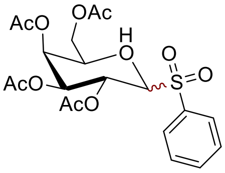

1c

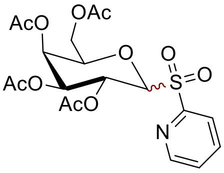

1d

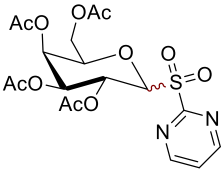

1e

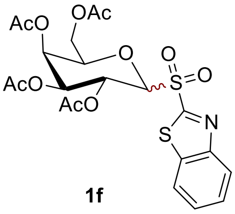

1f

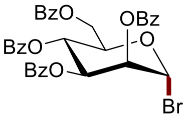

1g

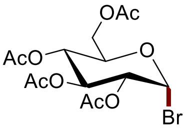

1h

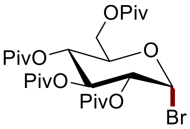

1i

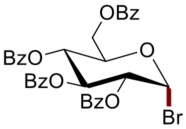

1j

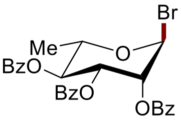

1k

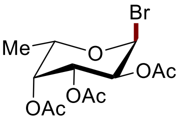

1l

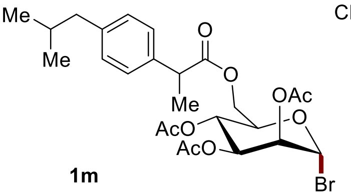

1m

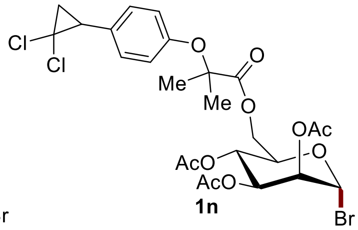

1n

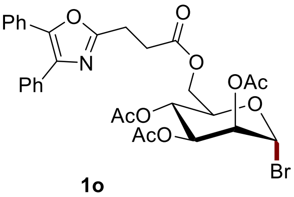

1o

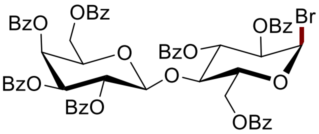

1p

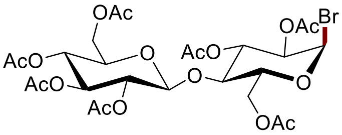

1q

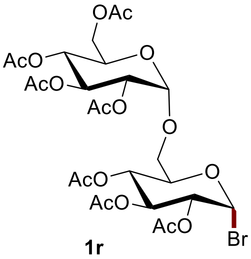

1r

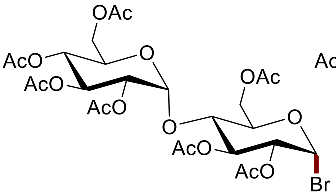

1s

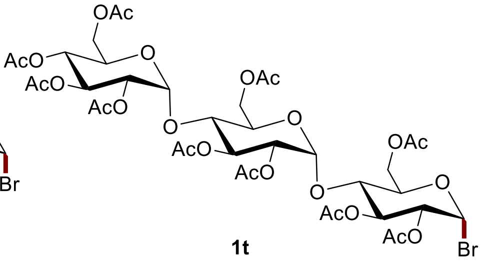

1t

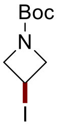

1u

General Procedure:

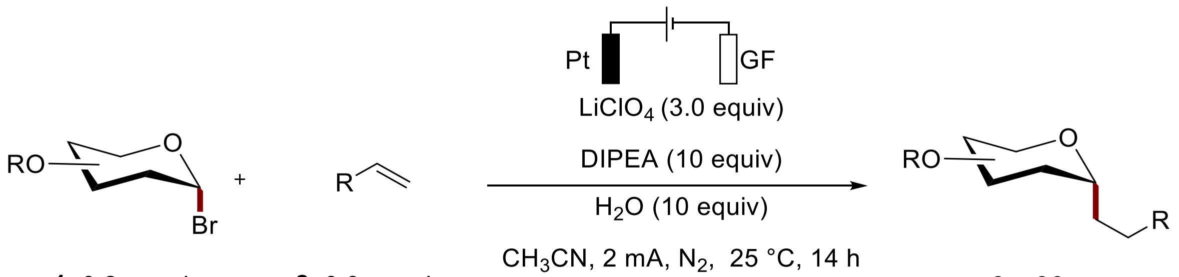

1, 0.2 mmol **2,** 0.3 mmol **3a–36**

General Procedure A: The electrolysis is carried out in an undivided cell with a Pt anode (10 mm × 15mm × 0.25 mm) and a GF cathode (10 mm × 15mm × 6 mm). Glycosyl bromide **1** (0.20 mmol), alkene **2** (0.30 mmol), $LiClO_4$ (63.6 mg, 0.60 mmol), DIPEA (77.6 mg, 0.6 mmol), H_2O (36 mg, 0.6 mmol), were dissolved in CH_3CN (4 mL) under N_2 atmosphere. The electrolysis was conducted with a constant current of 2.0 mA for 14 h at 25 ℃. Then the mixture was transferred to a flask and the electrodes were rinsed with DCM (3 × 5.0 mL). The solvent was removed under reduced pressure and purification by column chromatography on silica gel (*n*hexane/EtOAc) yielded the desired product.

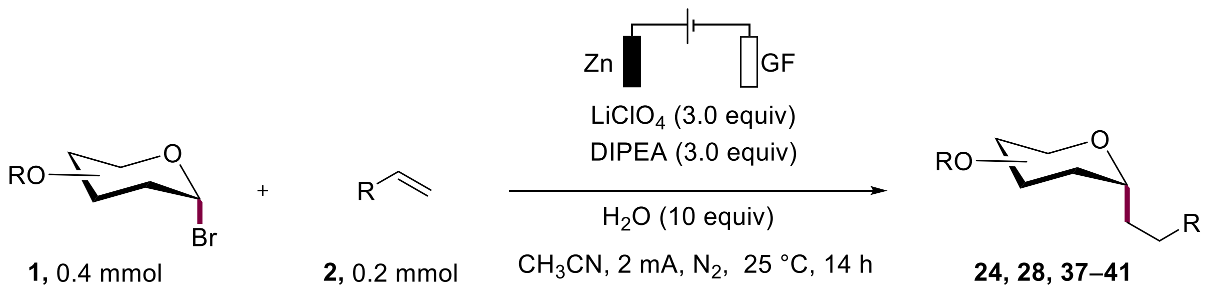

1, 0.4 mmol **2,** 0.2 mmol **24, 28, 37–41**

General Procedure B: The electrolysis is carried out in an undivided cell with a Zn anode (10 mm × 15mm × 0.25 mm) and a GF cathode (10 mm × 15mm × 6 mm). Glycosyl bromide **1** (0.40 mmol), alkene **2** (0.20 mmol), $LiClO_4$ (63.6 mg, 0.60 mmol), DIPEA (77.6 mg, 0.60 mmol), H_2O (36 mg, 0.60 mmol), were dissolved in CH_3CN (4.0 mL) under N_2 atmosphere. The electrolysis was conducted with a constant current of 2.0 mA for 14 h at 25 ℃. Then the mixture was transferred to a flask and the electrodes were rinsed with DCM (3 × 5.0 mL). The solvent was removed under reduced pressure and purification by column chromatography on silica gel (*n*hexane/EtOAc) yielded the desired product.

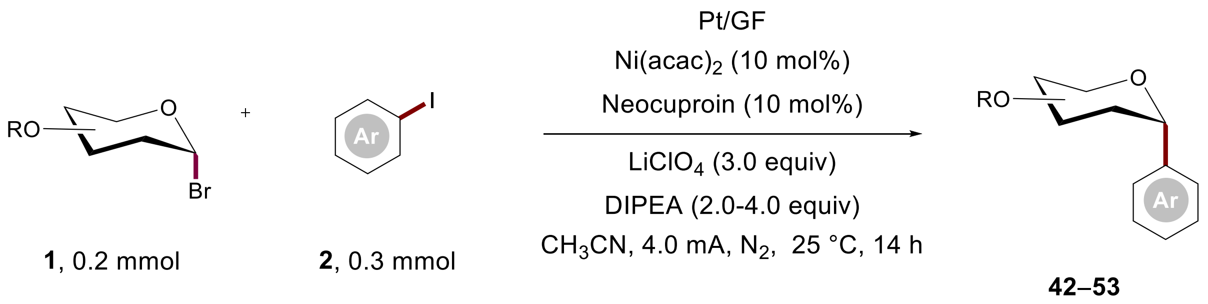

General Procedure C: The electrolysis is carried out in an undivided cell with a Pt anode (10 mm × 15mm × 0.25 mm) and a GF cathode (10 mm × 15mm × 6 mm). Glycosyl bromide **1** (0.20 mmol), aryl iodide (0.40 mmol), $Ni(acac)_2$ (10 mol %), Neocuproin (10 mol %), $LiClO_4$ (63.6 mg, 0.60 mmol), DIPEA (0.40−0.80 mmol), were dissolved in CH_3CN (4.0 mL) under N_2 atmosphere. The electrolysis was conducted with a constant current of 4.0 mA for 14 h at 25 ℃. Then the mixture was transferred to a flask and the electrodes were rinsed with DCM (3 × 5.0 mL). The solvent was removed under reduced pressure and purification by column chromatography on silica gel (*n*hexane/EtOAc) yielded the desired product.

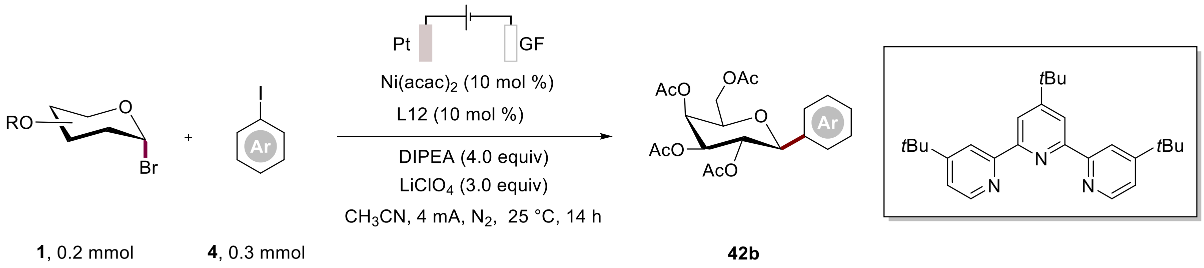

General Procedure D: The electrolysis is carried out in an undivided cell with a Pt anode (10 mm × 15mm × 0.25 mm) and a GF cathode (10 mm × 15mm × 6 mm). Glycosyl bromide **1** (0.20 mmol), aryl iodide (0.40 mmol), $Ni(acac)_2$ (10 mol %), *t*-Bu-Terpyridine **L12** (10 mol %), $LiClO_4$ (63.6 mg, 0.60 mmol), DIPEA (0.80 mmol), were dissolved in CH_3CN (4.0 mL) under N_2 atmosphere. The electrolysis was conducted with a constant current of 4.0 mA for 14 h at 25 ℃. Then the mixture was transferred to a flask and the electrodes were rinsed with DCM (3 × 5.0 mL). The solvent was removed under reduced pressure and purification by column chromatography on silica gel (*n*hexane/EtOAc) yielded the desired product **42ba−42be**.

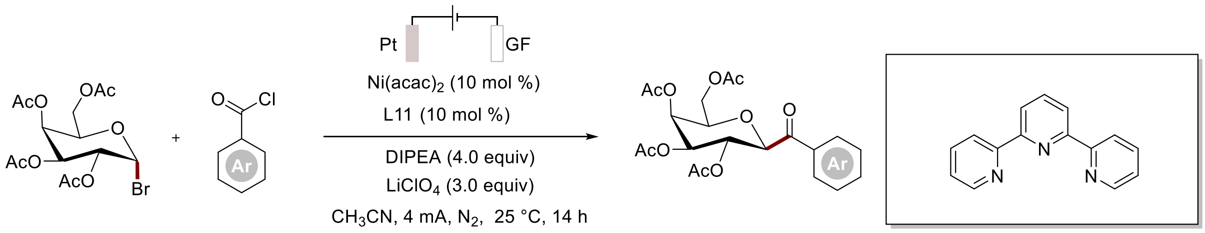

1a, 0.2 mmol **2bi–2bm**, 0.3 mmol **43ba-43be**

General Procedure E: The electrolysis is carried out in an undivided cell with a Pt anode (10 mm × 15mm × 0.25 mm) and a GF cathode (10 mm × 15mm × 6 mm). Glycosyl bromide **1a** (0.20 mmol), aryl iodide (0.40 mmol), $Ni(acac)_2$ (10 mol %), Terpyridine **L11** (10 mol %), $LiClO_4$ (63.6 mg, 0.60 mmol), DIPEA (0.80 mmol), were dissolved in CH_3CN (4.0 mL) under N_2 atmosphere. The electrolysis was conducted with a constant current of 4.0 mA for 14 h at 25 ℃. Then the mixture was transferred to a flask and the electrodes were rinsed with DCM (3 × 5.0 mL). The solvent was removed under reduced pressure and purification by column chromatography on silica gel (*n*hexane/EtOAc) yielded the desired product **43ba−43be**.

Characterization Data of Products

(2*R*,3*S*,4*R*,5*S*,6*R*)-2-[2-([1,1'-Biphenyl]-4-yl)ethyl]-6-(acetoxymethyl)tetrahydro-2*H*-pyran-3,4,5-triyl triacetate (3a)

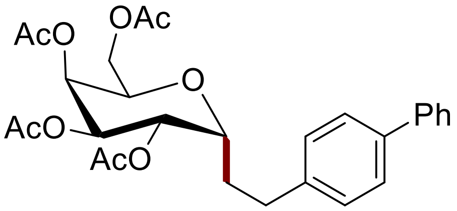

The general procedure **A** was followed using 4-vinyl-1,1'-biphenyl **2aa** (54.0 mg, 0.3 mmol), (2*R*,3*S*,4*S*,5*R*,6*R*)-2-(acetoxymethyl)-6-bromotetrahydro-2*H*-pyran-3,4,5-triyl triacetate **1a** (82.0 mg, 0.2 mmol), Purification by column chromatography on silica gel (*n*hexane/EtOAc: 6/1 to 2/1) yielded **3a** (91.2 mg, 89%) as a syrup.

^{1}H NMR (300 MHz, $CDCl_3$): δ = 7.58 (d, *J* = 7.5 Hz, 2H), 7.54 (d, *J* = 8.1 Hz, 2H), 7.43 (dd, *J* = 8.3, 6.8 Hz, 2H), 7.34 (d, *J* = 7.4 Hz, 1H), 7.27 (d, *J* = 8.1 Hz, 2H), 5.45 (t, *J* = 2.8 Hz, 1H), 5.31 (dd, *J* = 9.4, 5.2 Hz, 1H), 5.22 (dd, *J* = 9.4, 3.2 Hz, 1H), 4.26 (td, *J* = 9.0, 4.8 Hz, 2H), 4.13 (dt, *J* = 7.6, 4.8 Hz, 2H), 2.83 (ddd, *J* = 14.4, 9.8, 4.8 Hz, 1H), 2.66 (ddd, *J* = 14.0, 9.3, 7.2 Hz, 1H), 2.13 (s, 3H), 2.08 (s, 3H), 2.06 (s, 3H), 2.05 – 2.02 (m, 1H), 2.02 (s, 3H), 1.86 – 1.74 (m, 1H).

^{13}C NMR (75 MHz, $CDCl_3$): δ = 170.56 (C_q), 170.12 (C_q), 169.96 (C_q), 169.81 (C_q), 140.92, (C_q) 140.19 (C_q), 139.19 (C_q), 128.86 (CH), 128.76 (CH), 127.29 (CH), 127.14 (CH), 127.00 (CH), 71.44 (CH), 68.33 (CH), 68.19 (CH), 68.12 (CH), 67.70 (CH), 61.70 (CH_2), 31.03 (CH_2), 27.76 (CH_2), 20.81 (CH_3), 20.78 (CH_3), 20.73 (CH_3), 20.68 (CH_3).

IR (ATR): ṽ = 1736, 1372, 1270, 1234, 1043, 917, 847, 733, 634 cm^{-1}.
MS (ESI): m/z (relative intensity) 536 (100) $[M+Na]^+$,1047 (50) [2M+Na].
HR-MS (ESI): *m/z* calcd for $C_{28}H_{32}NaO_9^+$ $[M+Na]^+$: 535.1939, found: 535.1940.

(2R,3R,4R)-2-(Acetoxymethyl)-3,4-dihydro-2*H*-pyran-3,4-diyl diacetate (3b)

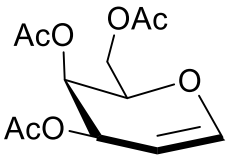

^{1}H NMR (300 MHz, $CDCl_3$): δ = 6.62 – 6.38 (m, 1H), 5.35 (t, J = 4.6 Hz, 1H), 5.23 (dd, J = 7.4, 5.7 Hz, 1H), 4.85 (dd, J = 6.2, 3.2 Hz, 1H), 4.41 (dd, J = 11.7, 5.4 Hz, 1H), 4.30 – 4.16 (m, 2H), 2.10 (s, 3H), 2.08 (s, 3H), 2.05 (s, 3H).

^{13}C NMR (75 MHz, $CDCl_3$): δ = 170.55 (C_q), 170.28 (C_q), 170.12 (C_q), 145.41 (CH), 98.84 (CH), 72.80 (CH), 63.88 (CH), 63.74 (CH), 61.92 (CH_2), 20.82 (CH_3), 20.76 (CH_3), 20.66 (CH_3).

(2*R*,3*S*,4*R*,5*S*,6*R*)-2-(Acetoxymethyl)-6-phenethyltetrahydro-2*H*-pyran-3,4,5-triyl triacetate (4)

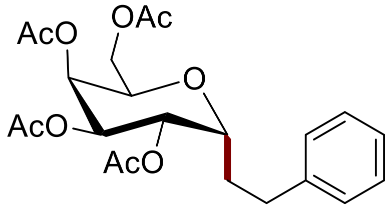

The general procedure **A** was followed using styrene **2ab** (31.2 mg, 0.3 mmol), (2*R*,3*S*,4*S*,5*R*,6*R*)-2-(acetoxymethyl)-6-bromotetrahydro-2*H*-pyran-3,4,5-triyl triacetate **1a** (82.0 mg, 0.2 mmol), Purification by column chromatography on silica gel (*n*hexane/EtOAc: 6/1 to 2/1) yielded **4** (50.6 mg, 58%) as a syrup.

^{1}H NMR (300 MHz, $CDCl_3$): δ = 7.30 (t, *J* = 7.3 Hz, 2H), 7.24 – 7.15 (m, 3H), 5.42 (t, *J* = 2.7 Hz, 1H), 5.28 (dd, *J* = 9.4, 5.1 Hz, 1H), 5.19 (dd, *J* = 9.5, 3.2 Hz, 1H), 4.22 (td, *J* = 9.0, 7.4, 3.5 Hz, 2H), 4.11 (dq, *J* = 8.1, 5.0, 3.6 Hz, 2H), 2.77 (ddd, *J* = 14.5, 9.9, 4.9 Hz, 1H), 2.70 – 2.52 (m, 1H), 2.13 (s, 3H), 2.07 (s, 3H), 2.04 (s, 3H), 2.01 (s, 3H), 2.00 – 1.95 (m, 1H), 1.81 – 1.64 (m, 1H).

^{13}C NMR (75 MHz, $CDCl_3$): δ = 170.55 (C_q), 170.10 (C_q), 169.94 (C_q), 169.78 (C_q), 141.06 (C_q), 128.53 (CH), 128.38 (CH), 126.16 (CH), 71.41 (CH), 68.29 (CH), 68.12 (CH), 68.08 (CH), 67.67 (CH), 61.66 (CH_2), 31.39 (CH_2), 27.73 (CH_2), 20.78 (CH_3), 20.76 (CH_3), 20.70 (CH_3), 20.66 (CH_3).

IR (ATR): $\tilde{\nu}$ =1745, 1738, 1369, 1214, 1109, 1046, 948, 701 cm^{-1}.
MS (ESI): m/z (relative intensity) 459 (100) $[M+Na]^+$,895.4 (75) [2M+Na].
HR-MS (ESI): *m/z* calcd for $C_{22}H_{28}NaO_9^+$ $[M+Na]^+$: 459.1626, found: 459.1628

(2*R*,3*S*,4*R*,5*S*,6*R*)-2-(Acetoxymethyl)-6-[4-(trifluoromethyl)phenethyl]tetrahydro-2*H*-pyran-3,4,5-triyl triacetate (5)

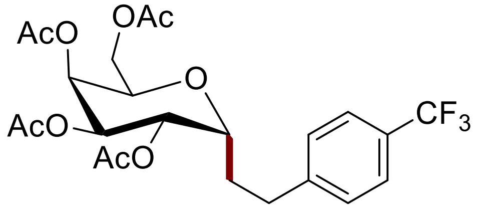

The general procedure **A** was followed using 1-(trifluoromethyl)-4-vinylbenzene **2ac** (51.6 mg, 0.3 mmol), (2*R*,3*S*,4*S*,5*R*,6*R*)-2-(acetoxymethyl)-6-bromotetrahydro-2*H*-pyran-3,4,5-triyl triacetate **1a** (82.0 mg, 0.2 mmol). Purification by column chromatography on silica gel (*n*hexane/EtOAc: 6/1 to 2/1) yielded **5** (65.5 mg, 65%) as a syrup.

^{1}H NMR (400 MHz, $CDCl_3$): δ = 7.55 (d, J = 8.2 Hz, 2H), 7.30 (d, J = 8.0 Hz, 2H), 5.42 (t, J = 2.9 Hz, 1H), 5.27 (dd, J = 9.2, 5.1 Hz, 1H), 5.19 (dd, J = 9.2, 3.3 Hz, 1H), 4.25 (dd, J = 12.8, 8.9 Hz, 1H), 4.19 (ddd, J = 11.5, 5.1, 3.8 Hz, 1H), 4.10 (dt, J = 7.4, 5.2 Hz, 2H), 2.83 (ddd, J = 14.4, 9.8, 4.9 Hz, 1H), 2.67 (ddd, J = 13.9, 9.2, 7.3 Hz, 1H), 2.12 (s, 3H), 2.07 (s, 3H), 2.05 (s, 3H), 2.01 (s, 3H), 2.01 – 1.96 (m, 1H), 1.73 (dddd, J = 14.1, 10.4, 7.3, 3.6 Hz, 1H).

^{13}C NMR (101 MHz, $CDCl_3$): δ = 170.54 (C_q), 170.06 (C_q), 169.91 (C_q), 169.76 (C_q), 145.18 (C_q), 128.77 (CH), 127.04 (d, J = 289.9 Hz, C_q), 125.48 (q, J = 3.8 Hz, CH), 71.09 (CH), 68.38 (CH), 68.33 (CH), 68.04 (CH), 67.54 (CH), 61.53 (CH_2), 31.26 (CH_2), 27.62 (CH_2), 20.76 (CH_3), 20.75 (CH_3), 20.69 (CH_3), 20.65 (CH_3).

^{19}F NMR (377 MHz, $CDCl_3$): δ = -62.37.

IR (ATR): ṽ =1745, 1618, 1369, 1324, 1209, 1161, 1124, 1066, 846, 733 cm^{-1}.

MS (ESI): m/z (relative intensity) 527 (100) $[M+Na]^+$,1031 (50) [2M+Na].

HR-MS (ESI): *m/z* calcd for $C_{23}H_{27}F_3NaO_9^+$ $[M+Na]^+$: 527.1499, found: 527.1501.

(2*R*,3*S*,4*R*,5*S*,6*R*)-2-(Acetoxymethyl)-6-(4-fluorophenethyl)tetrahydro-2*H*-pyran-3,4,5-triyl triacetate (6)

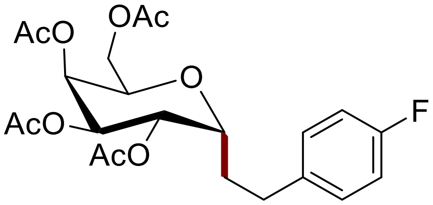

The general procedure **A** was followed using 1-fluoro-4-vinylbenzene **2ad** (36.6 mg, 0.3 mmol), (2*R*,3*S*,4*S*,5*R*,6*R*)-2-(acetoxymethyl)-6-bromotetrahydro-2*H*-pyran-3,4,5-triyl triacetate **1a** (82.0 mg, 0.2 mmol). Purification by column chromatography on silica gel (*n*hexane/EtOAc: 6/1 to 2/1) yielded **10** (59.9 mg, 66%) as a syrup.

^{1}H NMR (400 MHz, $CDCl_3$): δ = 7.14 (dd, *J* = 8.5, 5.5 Hz, 2H), 7.07 – 6.92 (m, 2H), 5.42 (dd, *J* = 3.3, 2.3 Hz, 1H), 5.27 (dd, *J* = 9.4, 5.1 Hz, 1H), 5.19 (dd, *J* = 9.4, 3.3 Hz, 1H), 4.30 – 4.14 (m, 2H), 4.14 – 4.07 (m, 2H), 2.74 (ddd, *J* = 14.3, 9.7, 4.9 Hz, 1H), 2.58 (ddd, *J* = 13.9, 9.2, 7.4 Hz, 1H), 2.13 (s, 3H), 2.07 (s, 3H), 2.05 (s, 3H), 2.01 (s, 3H), 1.99 – 1.93 (m, 1H), 1.79 – 1.64 (m, 1H).

^{13}C NMR (101 MHz, $CDCl_3$): δ = 170.59 (C_q), 170.12 (C_q), 169.98 (C_q), 169.81 (C_q), 161.43 (d, ^{1}J = 244.0 Hz, C_q), 136.62 (d, ^{4}J = 3.3 Hz, C_q), 129.80 (d, ^{3}J = 7.8 Hz, CH), 115.32 (d, ^{2}J = 21.2 Hz, CH), 71.17 (CH), 68.30 (CH), 68.21 (CH), 68.07 (CH), 67.62 (CH), 61.63 (CH_2), 30.56 (CH_2), 27.87 (CH_2), 20.82 (CH_3), 20.80 (CH_3), 20.74 (CH_3), 20.70 (CH_3).

^{19}F NMR (377 MHz, $CDCl_3$): δ = -117.11 (q, *J* = 7.9, 6.6 Hz).

IR (ATR): ṽ =1736, 1672, 1599, 1440, 1370, 1219, 1047, 759, 694 cm^{-1}.

MS (ESI): m/z (relative intensity) 477 (100) $[M+Na]^+$,931 (50) [2M+Na].

HR-MS (ESI): *m/z* calcd for $C_{22}H_{27}FNaO_9^+$ $[M+Na]^+$: 477.1531, found: 477.1531.

(2*R*,3*S*,4*R*,5*S*,6*R*)-2-(Acetoxymethyl)-6-(4-chlorophenethyl)tetrahydro-2*H*-pyran-3,4,5-triyl triacetate (7)

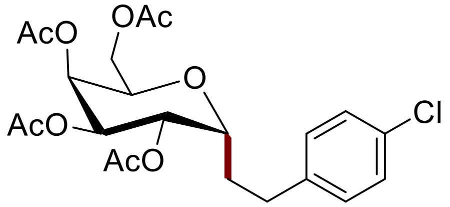

The general procedure **A** was followed using 1-chloro-4-vinylbenzene **2ae** (41.4 mg, 0.3 mmol), (2*R*,3*S*,4*S*,5*R*,6*R*)-2-(acetoxymethyl)-6-bromotetrahydro-2*H*-pyran-3,4,5-triyl triacetate **1a** (82.0 mg, 0.2 mmol). Purification by column chromatography on silica gel (*n*hexane/EtOAc: 6/1 to 2/1) yielded **7** (62.1 mg, 66%) as a syrup.

^{1}H NMR (400 MHz, $CDCl_3$): δ = 7.29 – 7.24 (m, 2H), 7.13 (d, *J* = 8.4 Hz, 2H), 5.43 (dd, *J* = 3.3, 2.3 Hz, 1H), 5.28 (dd, *J* = 9.3, 5.1 Hz, 1H), 5.20 (dd, *J* = 9.3, 3.3 Hz, 1H), 4.30 – 4.16 (m, 2H), 4.16 – 4.05 (m, 2H), 2.75 (ddd, *J* = 14.4, 9.7, 4.8 Hz, 1H), 2.59 (ddd, *J* = 13.9, 9.2, 7.4 Hz, 1H), 2.14 (s, 3H), 2.08 (s, 3H), 2.06 (s, 3H), 2.03 (s, 3H), 2.01 – 1.95 (m, 1H), 1.78 – 1.66 (m, 1H).

^{13}C NMR (101 MHz, $CDCl_3$): δ = 170.54 (C_q), 170.07 (C_q), 169.93 (C_q), 169.76, (C_q) 139.46 (C_q), 131.96 (C_q), 129.78 (CH), 128.65 (CH), 71.11 (CH), 68.33 (CH), 68.30 (CH), 68.07 (CH), 67.59 (CH), 61.58 (CH_2), 30.73 (CH_2), 27.72 (CH_2), 20.77 (CH_3), 20.76 (CH_3), 20.71 (CH_3), 20.66 (CH_3).

IR (ATR): ṽ =1746, 1493, 1370, 1227, 1218, 1050, 526, $^{-1}$.
MS (ESI): m/z (relative intensity) 493 (100) [M+Na]$^{+}$,963 (25) [2M+Na].
HR-MS (ESI): *m/z* calcd for $C_{22}H_{27}ClNaO_9^{+}$ [M+Na]$^{+}$: 493.1236, found: 493.1240.

(2*R*,3*S*,4*R*,5*S*,6*R*)-2-(Acetoxymethyl)-6-(4-bromophenethyl)tetrahydro-2*H*-pyran-3,4,5-triyl triacetate (8)

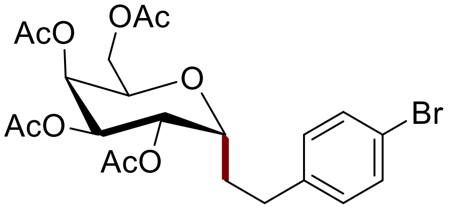

The general procedure **A** was followed using 1-bromo-4-vinylbenzene **2af** (54.6 mg, 0.3 mmol), (2*R*,3*S*,4*S*,5*R*,6*R*)-2-(acetoxymethyl)-6-bromotetrahydro-2*H*-pyran-3,4,5-triyl triacetate **1a** (82.0 mg, 0.2 mmol). Purification by column chromatography on silica gel (*n*hexane/EtOAc: 6/1 to 2/1) yielded **8** (80.0 mg, 70%) as a syrup.

^{1}H NMR (300 MHz,): δ = 7.40 (d, *J* = 8.0 Hz, 2H), 7.05 (d, *J* = 8.0 Hz, 2H), 5.41 (d, *J* = 2.8 Hz, 1H), 5.26 (dd, *J* = 9.3, 5.0 Hz, 1H), 5.18 (dd, *J* = 9.3, 3.3 Hz, 1H), 4.31 – 4.13 (m, 2H), 4.15 – 4.03 (m, 2H), 2.71 (ddd, *J* = 14.4, 9.7, 4.9 Hz, 1H), 2.55 (dt, *J* = 14.1, 8.5 Hz, 1H), 2.12 (s, 3H), 2.06 (s, 3H), 2.04 (s, 3H), 2.00 (s, 3H), 1.98 – 1.90 (m, 1H), 1.75 – 1.60 (m, 1H).

^{13}C NMR (75 MHz, $CDCl_3$): δ = 170.48 (C_q), 170.02 (C_q), 169.88 (C_q), 169.71 (C_q), 139.92 (C_q), 131.54 (CH), 130.14 (CH), 119.89 (C_q), 71.03 (CH), 68.22 (2 CH), 67.98 (CH), 67.51 (CH), 61.52 (CH_2), 30.72 (CH_2), 27.59 (CH_2), 20.73 (2 CH_3), 20.66 (CH_3), 20.62 (CH_3).

IR (ATR): ṽ = 1739, 1372, 1234, 1044, 847, 751, 667, 634, 607 cm^{-1}.
MS (ESI): m/z (relative intensity) 539 (100) $[M+Na]^+$,1053 (50) [2M+Na].
HR-MS (ESI): *m/z* calcd for $C_{22}H_{27}BrNaO_9^+$ $[M+Na]^+$: 539.0731, found: 539.0732.

(2*R*,3*S*,4*R*,5*S*,6*R*)-2-(Acetoxymethyl)-6-(4-cyanophenethyl)tetrahydro-2*H*-pyran-3,4,5-triyl triacetate (9)

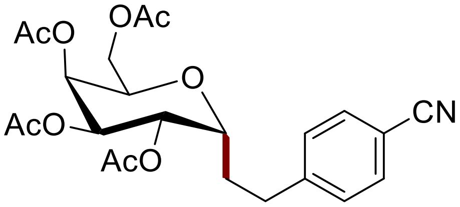

The general procedure **A** was followed using 4-vinylbenzonitrile **2ag** (38.7 mg, 0.3 mmol), (2*R*,3*S*,4*S*,5*R*,6*R*)-2-(acetoxymethyl)-6-bromotetrahydro-2*H*-pyran-3,4,5-triyl triacetate **1a** (82.0 mg, 0.2 mmol). Purification by column chromatography on silica gel (*n*hexane/EtOAc: 6/1 to 2/1) yielded **9** (65.5 mg, 71%) as a syrup.

^{1}H NMR (400 MHz, $CDCl_3$): δ = 7.58 (d, *J* = 8.3 Hz, 2H), 7.29 (d, *J* = 8.2 Hz, 2H), 5.41 (t, *J* = 2.9 Hz, 1H), 5.25 (dd, *J* = 9.1, 5.0 Hz, 1H), 5.18 (dd, *J* = 9.2, 3.3 Hz, 1H), 4.26 (dd, *J* = 12.8, 9.0 Hz, 1H), 4.17 (ddd, *J* = 11.4, 5.1, 3.6 Hz, 1H), 4.08 (dt, *J* = 8.1, 5.1 Hz, 2H), 2.82 (ddd, *J* = 14.4, 9.9, 4.9 Hz, 1H), 2.66 (ddd, *J* = 14.0, 9.4, 7.3 Hz, 1H), 2.11 (s, 3H), 2.06 (s, 3H), 2.04 (s, 3H), 2.03 – 2.02 (m, 1H), 2.01 (s, 3H), 1.74 – 1.65 (m, 1H).

^{13}C NMR (101 MHz, $CDCl_3$): *δ* = 170.47 (C_q), 169.96 (C_q), 169.82 (C_q), 169.68 (C_q), 146.64 (C_q), 132.33 (CH), 129.21 (CH), 118.82 (C_q), 110.17 (C_q), 70.85 (CH), 68.40 (CH), 68.26 (CH), 67.90 (CH), 67.39 (CH), 61.39 (CH_2), 31.51 (CH_2), 27.41 (CH_2), 20.71 2 (CH_3), 20.64 (CH_3), 20.60 (CH_3).

IR (ATR): ṽ = 2934, 1746, 1369, 1211, 1057, 1046, 905, 734, cm^{-1}.
MS (ESI): m/z (relative intensity) 484 (100) [M+Na]$^+$,945 (50) [2M+Na].
HR-MS (ESI): *m/z* calcd for $C_{23}H_{27}NNaO_9^+$ [M+Na]$^+$: 484.1578, found: 484.1580.

(2*R*,3*S*,4*R*,5*S*,6*R*)-2-(Acetoxymethyl)-6-[4-(methoxycarbonyl)phenethyl]tetrahydro-2*H*-pyran-3,4,5-triyl triacetate (10)

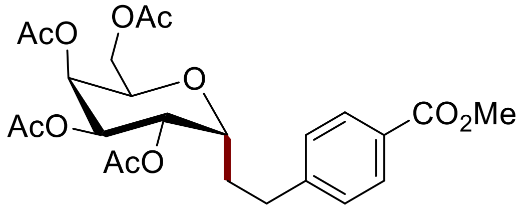

The general procedure **A** was followed using methyl 4-vinylbenzoate **2ah** (48.6 mg, 0.3 mmol), (2*R*,3*S*,4*S*,5*R*,6*R*)-2-(acetoxymethyl)-6-bromotetrahydro-2*H*-pyran-3,4,5-triyl triacetate **1a** (82.0 mg, 0.2 mmol). Purification by column chromatography on silica gel (*n*hexane/EtOAc: 6/1 to 2/1) yielded **10** (75.1 mg, 76%) as a syrup.

^{1}H NMR (300 MHz, $CDCl_3$): δ = 7.96 (d, J = 8.2 Hz, 2H), 7.25 (d, J = 8.1 Hz, 2H), 5.42 (t, J = 2.8 Hz, 1H), 5.27 (dd, J = 9.3, 5.0 Hz, 1H), 5.18 (dd, J = 9.3, 3.2 Hz, 1H), 4.27 – 4.14 (m, 2H), 4.13 – 4.03 (m, 2H), 3.90 (s, 3H), 2.82 (ddd, J = 14.4, 9.7, 4.8 Hz, 1H), 2.66 (ddd, J = 14.0, 9.2, 7.3 Hz, 1H), 2.12 (s, 3H), 2.07 (s, 3H), 2.04 (s, 3H), 2.04 – 2.01 (m, 1H), 2.01 (s, 3H), 1.80 – 1.69 (m, 1H).

^{13}C NMR (75 MHz, $CDCl_3$): δ = 170.53 (C_q), 170.07 (C_q), 169.92 (C_q), 169.75 (C_q), 166.97 (C_q), 146.51 (C_q), 129.88 (CH), 128.45 (CH), 128.23 (C_q), 71.15 (CH), 68.28 (CH), 68.01 (CH), 67.55 (CH), 61.54 (CH_2), 52.03 (CH), 31.42 (CH_2), 27.46 (CH_2), 20.77 (CH_3), 20.70 (CH_3), 20.66 (CH_3).

IR (ATR): $\tilde{\nu}$ = 1731, 1372, 1281, 1239, 1215, 1111, 1044, 750, 667 cm^{1}.
MS (ESI): m/z (relative intensity) 517 (100) $[M+Na]^+$,1011 (50) [2M+Na].
HR-MS (ESI): *m/z* calcd for $C_{24}H_{30}NaO_{11}^+$ $[M+Na]^+$: 517.1680, found: 517.1682.

(2*R*,3*S*,4*R*,5*S*,6*R*)-2-(Acetoxymethyl)-6-[4-(phenylcarbamoyl)phenethyl]tetrahydro-2*H*-pyran-3,4,5-triyl triacetate (11)

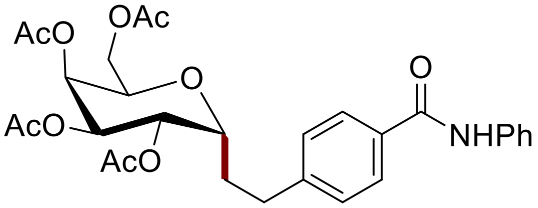

The general procedure **A** was followed using *N*-phenyl-4-vinylbenzamide **2ai** (66.9 mg, 0.3 mmol), (2*R*,3*S*,4*S*,5*R*,6*R*)-2-(acetoxymethyl)-6-bromotetrahydro-2*H*-pyran-3,4,5-triyl triacetate **1a** (82.0 mg, 0.2 mmol). Purification by column chromatography on silica gel (*n*hexane/EtOAc: 6/1 to 2/1) yielded **11** (58.9 mg, 53%) as a syrup.

^{1}H NMR (400 MHz, $CDCl_3$): δ = 7.91 (s, 1H), 7.85 (d, *J* = 8.3 Hz, 2H), 7.70 (d, *J* = 7.4 Hz, 2H), 7.42 (dd, *J* = 8.5, 7.4 Hz, 2H), 7.36 (d, *J* = 8.2 Hz, 2H), 7.20 (t, *J* = 7.4 Hz, 1H), 5.47 (dd, *J* = 3.3, 2.2 Hz, 1H), 5.32 (dd, *J* = 9.3, 5.1 Hz, 1H), 5.24 (dd, *J* = 9.3, 3.3 Hz, 1H), 4.25 (td, *J* = 9.3, 8.4, 4.4 Hz, 2H), 4.14 – 4.01 (m, 2H), 2.87 (ddd, *J* = 14.2, 9.1, 5.3 Hz, 1H), 2.81 – 2.70 (m, 1H), 2.17 (s, 3H), 2.12 (s, 3H), 2.11 (s, 3H), 2.09 – 2.07 (m, 1H), 2.06 (s, 3H), 1.85 – 1.76 (m, 1H).

^{13}C NMR (101 MHz, $CDCl_3$): δ = 170.66 (C_q), 170.09 (C_q), 169.99 (C_q), 169.81 (C_q), 165.61 (C_q), 145.37 (C_q), 138.05 (C_q), 133.14 (C_q), 129.12 (CH), 128.89 (CH), 127.36 (CH), 124.50 (CH), 120.10 (CH), 71.37 (CH), 68.31 (CH), 68.23 (CH), 68.04 (CH), 67.54 (CH), 61.43 (CH_2), 31.44 (CH_2), 30.96 (CH_2), 20.84 (2 CH_3), 20.75 (CH_3), 20.71 (CH_3).

IR (ATR): ṽ = 1748, 1672, 1599, 1537, 1440, 1370, 1219, 1047, 759, 694 cm^1.
MS (ESI): m/z (relative intensity) 578 (100) $[M+Na]^+$,1133 (50) [2M+Na].
HR-MS (ESI): *m/z* calcd for $C_{29}H_{33}NNaO_{10}^+$ $[M+Na]^+$: 578.1997, found: 578.1997.

(2*R*,3*S*,4*R*,5*S*,6*R*)-2-(Acetoxymethyl)-6-(3-methylphenethyl)tetrahydro-2*H*-pyran-3,4,5-triyl triacetate (12)

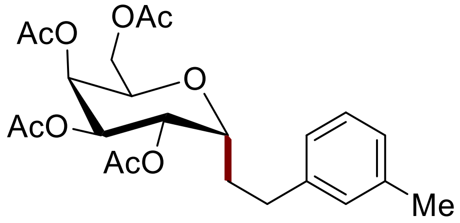

The general procedure **A** was followed using 1-methyl-3-vinylbenzene **2aj** (35.4 mg, 0.3 mmol), (2*R*,3*S*,4*S*,5*R*,6*R*)-2-(acetoxymethyl)-6-bromotetrahydro-2*H*-pyran-3,4,5-triyl triacetate **1a** (82.0 mg, 0.2 mmol). Purification by column chromatography on silica gel (*n*hexane/EtOAc: 6/1 to 2/1) yielded **12** (56.7 mg, 63%) as a syrup.

^{1}H NMR (400 MHz, $CDCl_3$): δ = 7.18 (t, J = 7.5 Hz, 1H), 7.07 – 6.90 (m, 3H), 5.42 (dd, J = 3.4, 2.2 Hz, 1H), 5.29 (dd, J = 9.5, 5.2 Hz, 1H), 5.20 (dd, J = 9.5, 3.3 Hz, 1H), 4.27 – 4.18 (m, 2H), 4.16 – 4.05 (m, 2H), 2.73 (ddd, J = 14.5, 10.1, 4.9 Hz, 1H), 2.56 (ddd, J = 13.8, 9.6, 7.0 Hz, 1H), 2.34 (s, 3H), 2.13 (s, 3H), 2.07 (s, 3H), 2.04 (s, 3H), 2.01 (s, 3H), 2.00 – 1.94 (m, 1H), 1.80 – 1.66 (m, 1H).

^{13}C NMR (101 MHz, $CDCl_3$): δ = 170.52 (C_q), 170.09 (C_q), 169.94 (C_q), 169.77 (C_q), 141.03 (C_q), 138.09 (C_q), 129.14 (CH), 128.40 (CH), 126.86 (CH), 125.35 (CH), 71.52 (CH), 68.26 (CH), 68.06 (CH), 68.04 (CH), 67.67 (CH), 61.65 (CH_2), 31.30 (CH_2), 27.74 (CH_2), 21.36 (CH_3), 20.77 (CH_3), 20.74 (CH_3), 20.69 (CH_3), 20.65 (CH_3).

IR (ATR): $\tilde{v}$ = 1756, 1368, 1207, 1109, 1044, 948, 911, 792 cm^{-1}.
MS (ESI): m/z (relative intensity) 473 (100) $[M+Na]^+$, 923 (50) [2M+Na].
HR-MS (ESI): *m/z* calcd for $C_{23}H_{30}NaO_9^+$ $[M+Na]^+$: 473.1782, found: 473.1786.

(2*R*,3*S*,4*R*,5*S*,6*R*)-2-(Acetoxymethyl)-6-(3-methoxyphenethyl)tetrahydro-2*H*-pyran-3,4,5-triyl triacetate (13)

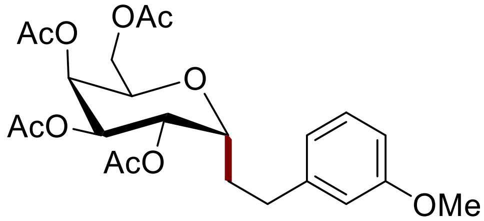

The general procedure **A** was followed using 1-methoxy-3-vinylbenzene **2ak** (40.2 mg, 0.3 mmol), (2*R*,3*S*,4*S*,5*R*,6*R*)-2-(acetoxymethyl)-6-bromotetrahydro-2*H*-pyran-3,4,5-triyl triacetate **1a** (82.0 mg, 0.2 mmol). Purification by column chromatography on silica gel (*n*hexane/EtOAc: 6/1 to 2/1) yielded **13** (64.3 mg, 69%) as a syrup.

^{1}H NMR (400 MHz, $CDCl_3$): δ = 7.24 (td, J = 7.5, 1.2 Hz, 1H), 6.84 – 6.75 (m, 3H), 5.45 (dd, J = 3.4, 2.2 Hz, 1H), 5.31 (dd, J = 9.4, 5.2 Hz, 1H), 5.22 (dd, J = 9.4, 3.3 Hz, 1H), 4.30 – 4.20 (m, 2H), 4.14 – 4.10 (m, 2H), 3.83 (s, 3H), 2.78 (ddd, J = 14.5, 10.0, 4.8 Hz, 1H), 2.61 (ddd, J = 13.8, 9.5, 7.1 Hz, 1H), 2.16 (s, 3H), 2.10 (s, 3H), 2.08 (s, 3H), 2.07 – 2.05 (m, 1H), 2.04 (s, 3H), 1.83 – 1.71 (m, 1H).

^{13}C NMR (101 MHz, $CDCl_3$): δ = 170.55 (C_q), 170.09 (C_q), 169.93 (C_q), 169.78 (C_q), 159.73 (C_q), 142.68 (C_q), 129.50 (CH), 120.74 (CH), 114.36 (CH), 111.21 (CH), 71.37 (CH), 68.27 (CH), 68.11 (CH), 68.05 (CH), 67.65 (CH), 61.65 (CH_2), 55.14 (CH_3), 31.41 (CH_2), 27.64 (CH_2), 20.78 (CH_3), 20.76 (CH_3), 20.70 (CH_3), 20.66 (CH_3).

IR (ATR): $\tilde{v}$ = 1746, 1368, 1257, 1207, 1041, 908, 729, 697, 602 cm^{-1}.
MS (ESI): m/z (relative intensity) 489 (100) $[M+Na]^+$, 955 (50) [2M+Na].
HR-MS (ESI): *m/z* calcd for $C_{23}H_{30}NaO_{10}^+$ $[M+Na]^+$: 489.1731, found: 489.1739.

(2*R*,3*S*,4*R*,5*S*,6*R*)-2-(Acetoxymethyl)-6-[2-(naphthalen-1-yl)ethyl]tetrahydro-2*H*-pyran-3,4,5-triyl triacetate (14)

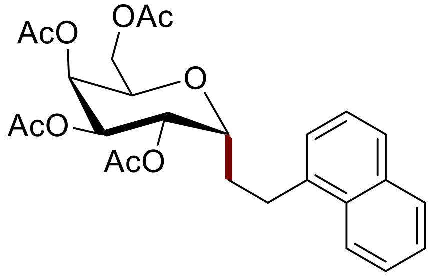

The general procedure **A** was followed using 1-vinylnaphthalene **2al** (46.2 mg, 0.3 mmol), (2*R*,3*S*,4*S*,5*R*,6*R*)-2-(acetoxymethyl)-6-bromotetrahydro-2*H*-pyran-3,4,5-triyl triacetate **1a** (82.0 mg, 0.2 mmol). Purification by column chromatography on silica gel (*n*hexane/EtOAc: 6/1 to 2/1) yielded **14** (54.5 mg, 56%) as a syrup.

^{1}H NMR (400 MHz, $CDCl_3$): δ = 8.02 (d, *J* = 8.3 Hz, 1H), 7.87 (dd, *J* = 8.0, 1.6 Hz, 1H), 7.74 (d, *J* = 8.0 Hz, 1H), 7.57 – 7.45 (m, 2H), 7.45 – 7.33 (m, 2H), 5.44 (dd, *J* = 3.3, 2.2 Hz, 1H), 5.30 (dd, *J* = 9.4, 5.3 Hz, 1H), 5.18 (dd, *J* = 9.4, 3.3 Hz, 1H), 4.41 – 4.26 (m, 2H), 4.23 – 4.08 (m, 2H), 3.32 (ddd, *J* = 14.6, 10.3, 4.8 Hz, 1H), 3.03 (ddd, *J* = 14.2, 9.8, 6.7 Hz, 1H), 2.14 (s, 3H), 2.09 (s, 3H), 2.00 (s, 3H), 1.98 (s, 3H), 1.93 – 1.85 (m, 1H).

^{13}C NMR (101 MHz, $CDCl_3$): δ = 170.58 (C_q), 170.08 (C_q), 169.88 (C_q), 169.76 (C_q), 137.15 (C_q), 133.90 (C_q), 131.61 (C_q), 128.92 (CH), 126.99 (CH), 126.16 (CH), 126.03 (CH), 125.56 (CH), 125.55 (CH), 123.31 (CH), 71.86 (CH), 68.31 (CH), 68.03 (CH), 67.70 (CH), 61.81 (CH_2), 28.63 (CH_2), 26.89 (CH_2), 20.78 (CH_3), 20.71 (CH_3), 20.68 (CH_3), 20.67 (CH_3).

IR (ATR): ṽ = 1746, 1368, 1208, 1109, 1047, 906, 779, 727, 648 cm^{-1}.
MS (ESI): m/z (relative intensity) 509 (100) $[M+Na]^+$, 995 (50) [2M+Na].
HR-MS (ESI): *m/z* calcd for $C_{26}H_{30}NaO_9^+$ $[M+Na]^+$: 509.1782, found: 509.1794.

(2*R*,3*S*,4*R*,5*S*,6*R*)-2-(Acetoxymethyl)-6-[2-(naphthalen-2-yl)ethyl]tetrahydro-2*H*-pyran-3,4,5-triyl triacetate (15)

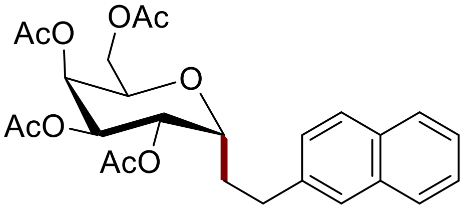

The general procedure **A** was followed using 2-vinyl naphthalene **2am** (46.2 mg, 0.3 mmol), (2*R*,3*S*,4*S*,5*R*,6*R*)-2-(acetoxymethyl)-6-bromotetrahydro-2*H*-pyran-3,4,5-triyl triacetate **1a** (82.0 mg, 0.2 mmol). Purification by column chromatography on silica gel (*n*hexane/EtOAc: 6/1 to 2/1) yielded **15** (60.3 mg, 62%) as a syrup.

^{1}H NMR (300 MHz, $CDCl_3$): δ = 7.80 (dd, *J* = 8.9, 6.5 Hz, 3H), 7.63 (s, 1H), 7.45 (tt, *J* = 7.0, 5.2 Hz, 2H), 7.33 (dd, *J* = 8.4, 1.7 Hz, 1H), 5.44 (d, *J* = 2.7 Hz, 1H), 5.30 (dd, *J* = 9.4, 5.0 Hz, 1H), 5.22 (dd, *J* = 9.4, 3.2 Hz, 1H), 4.38 – 4.19 (m, 2H), 4.18 – 4.06 (m, 2H), 2.94 (ddd, *J* = 14.3, 9.7, 4.9 Hz, 1H), 2.88 – 2.69 (m, 1H), 2.22 – 2.15 (m, 1H), 2.13 (s, 3H), 2.08 (s, 3H), 2.04 (s, 3H), 2.01 (s, 3H), 1.92 – 1.76 (m, 1H).

^{13}C NMR (75 MHz, $CDCl_3$): δ = 170.57 (C_q), 170.12 (C_q), 169.97 (C_q), 169.79 (C_q), 138.55 (2 C_q), 133.60 (C_q), 132.09 (C_q), 128.17 (CH), 127.64 (CH), 127.40 (CH), 127.08 (CH), 126.53 (CH), 126.09 (CH), 125.37 (CH), 71.40 (CH), 68.33 (CH), 68.25 (CH), 68.13 (CH), 67.68 (CH), 61.66 (CH_2), 31.53 (CH_2), 27.71 (CH_2), 20.80 (CH_3), 20.72 (CH_3), 20.68 (CH_3).

IR (ATR): $\tilde{\nu}$ = 1730, 1372, 1239, 1215, 1044, 746, 666, 607 cm^{-1}.
MS (ESI): m/z (relative intensity) 509 (100) $[M+Na]^+$, 995 (50) [2M+Na].
HR-MS (ESI): *m/z* calcd for $C_{26}H_{30}NaO_9^+$ $[M+Na]^+$: 509.1782, found: 509.1789.

(2*R*,3*S*,4*R*,5*S*,6*R*)-2-(Acetoxymethyl)-6-[2-(benzo[b]thiophen-2-yl)ethyl)tetrahydro-2*H*-pyran-3,4,5-triyl triacetate (16)

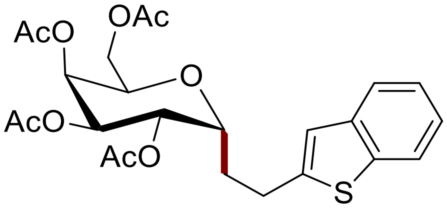

The general procedure **A** was followed using 2-vinylbenzo[*b*]thiophene **2an** (48.0 mg, 0.3 mmol), (2*R*,3*S*,4*S*,5*R*,6*R*)-2-(acetoxymethyl)-6-bromotetrahydro-2*H*-pyran-3,4,5-triyl triacetate **1a** (82.0 mg, 0.2 mmol). Purification by column chromatography on silica gel (*n*hexane/EtOAc: 6/1 to 2/1) yielded **16** (50.2 mg, 51%) as a syrup.

^{1}H NMR (400 MHz, $CDCl_3$): δ = 7.76 (d, *J* = 8.3 Hz, 1H), 7.67 (d, *J* = 8.0 Hz, 1H), 7.36 – 7.22 (m, 2H), 7.03 (s, 1H), 5.43 (dd, *J* = 3.4, 2.3 Hz, 1H), 5.30 (dd, *J* = 9.3, 5.2 Hz, 1H), 5.21 (dd, *J* = 9.3, 3.3 Hz, 1H), 4.34 – 4.23 (m, 2H), 4.19 – 4.07 (m, 2H), 3.13 – 3.00 (m, 1H), 2.93 (dddd, *J* = 15.1, 8.7, 7.5, 0.9 Hz, 1H), 2.12 (s, 3H), 2.12 – 2.09 (m, 1H), 2.08 (s, 3H), 2.07 (s, 3H), 2.01 (s, 3H), 1.94 – 1.82 (m, 1H).

^{13}C NMR (101 MHz, $CDCl_3$): δ = 170.52 (C_q), 170.05 (C_q), 169.90 (C_q), 169.74 (C_q), 144.45 (C_q), 139.99 (C_q), 139.25 (C_q), 124.21 (CH), 123.68 (CH), 122.80 (CH), 122.10 (CH), 121.22 (CH), 70.98 (CH), 68.34 (CH), 68.16 (CH), 68.01 (CH), 67.53 (CH), 61.57 (CH_2), 27.42 (CH_2), 26.30 (CH_2), 20.75 (2 CH_3), 20.68 (CH_3), 20.63 (CH_3).

IR (ATR): $\tilde{v}$ =1745, 1435, 1368, 1210, 1046, 905, 750, 727, 602 cm^{-1}.
MS (ESI): m/z (relative intensity) 515 (100) $[M+Na]^+$, 1007 (50) [2M+Na].
HR-MS (ESI): *m/z* calcd for $C_{24}H_{28}SNaO_9^+$ $[M+Na]^+$: 515.1346, found: 515.1360.

(2*R*,3*S*,4*R*,5*S*,6*R*)-2-(Acetoxymethyl)-6-[2-(thiophen-2-yl)ethyl]tetrahydro-2*H*-pyran-3,4,5-triyl triacetate (17)

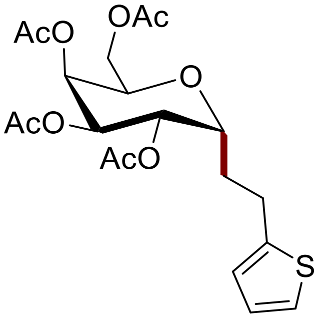

The general procedure **A** was followed using 2-vinylthiophene **2ao** (33.0 mg, 0.3 mmol), (2*R*,3*S*,4*S*,5*R*,6*R*)-2-(acetoxymethyl)-6-bromotetrahydro-2*H*-pyran-3,4,5-triyl triacetate **1a** (82.0 mg, 0.2 mmol). Purification by column chromatography on silica gel (*n*hexane/EtOAc: 6/1 to 2/1) yielded **17** (37.1 mg, 42%) as a syrup.

^{1}H NMR (400 MHz, $CDCl_3$): δ = 7.18 (dd, *J* = 5.1, 1.2 Hz, 1H), 6.97 (dd, *J* = 5.2, 3.4 Hz, 1H), 6.85 (dd, *J* = 3.5, 1.1 Hz, 1H), 5.47 (dd, *J* = 3.3, 2.2 Hz, 1H), 5.33 (dd, *J* = 9.4, 5.2 Hz, 1H), 5.24 (dd, *J* = 9.4, 3.3 Hz, 1H), 4.35 – 4.21 (m, 2H), 4.23 – 4.08 (m, 2H), 3.10 – 2.98 (m, 1H), 2.95 – 2.82 (m, 1H), 2.17 (s, 3H), 2.15 – 2.12 (m, 1H), 2.12 (s, 3H), 2.11 (s, 3H), 2.06 (s, 3H), 1.91 – 1.77 (m, 1H).

^{13}C NMR (101 MHz, $CDCl_3$): δ = 170.54 (C_q), 170.08 (C_q), 169.94 (C_q), 169.78 (C_q), 143.55 (C_q), 126.88 (CH), 124.67 (CH), 123.42 (CH), 71.05 (CH), 68.26 (CH), 68.21 (CH), 68.04 (CH), 67.58 (CH), 61.59 (CH_2), 28.00 (CH_2), 25.46 (CH_2), 20.78 (CH_3), 20.76 (CH_2), 20.70 (CH_2), 20.66 (CH_2).

IR (ATR): $\tilde{v}$ = 1744, 1738, 1390, 1369, 1213, 1057, 949, 704 cm^{-1}.
MS (ESI): m/z (relative intensity) 465 (100) $[M+Na]^+$, 907 (50) [2M+Na].
HR-MS (ESI): *m/z* calcd for $C_{20}H_{26}SNaO_9^+$ $[M+Na]^+$: 465.1190, found: 465.1191.

(2*R*,3*S*,4*R*,5*R*,6*R*)-2-[2-([1,1'-Biphenyl]-4-yl)ethyl]-6-(acetoxymethyl)tetrahydro-2*H*-pyran-3,4,5-triyl triacetate (18)

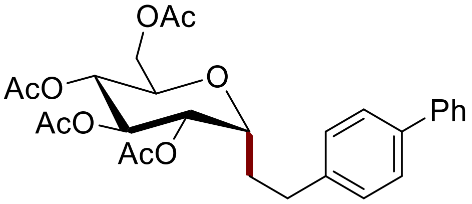

The general procedure **A** was followed using 4-vinyl-1,1'-biphenyl **2aa** (54.0 mg, 0.3 mmol), (2*R*,3*R*,4*S*,5*R*,6*R*)-2-(acetoxymethyl)-6-bromotetrahydro-2*H*-pyran-3,4,5-triyl triacetate **1h** (82.0 mg, 0.2 mmol), Purification by column chromatography on silica gel (*n*hexane/EtOAc: 6/1 to 2/1) yielded **18** (71.7 mg, 70%) as a syrup.

^{1}H NMR (400 MHz, $CDCl_3$): δ = 7.62 – 7.49 (m, 4H), 7.47 – 7.39 (m, 2H), 7.35 – 7.30 (m, 1H), 7.29 – 7.24 (m, 2H), 5.33 (dd, *J* = 9.5, 8.7 Hz, 1H), 5.10 (dd, *J* = 9.5, 5.8 Hz, 1H), 5.00 (dd, *J* = 9.5, 8.7 Hz, 1H), 4.29 – 4.18 (m, 2H), 4.12 – 4.09 (m, 1H), 3.89 (ddd, *J* = 9.4, 5.5, 2.6 Hz, 1H), 2.81 (ddd, *J* = 14.6, 10.0, 5.0 Hz, 1H), 2.65 (ddd, *J* = 13.8, 9.3, 7.0 Hz, 1H), 2.14 – 2.11 (m, 1H), 2.10 (s, 3H), 2.03 (s, 3H), 2.03 (s, 3H), 2.01 (s, 3H), 1.90 – 1.76 (m, 1H).

^{13}C NMR (101 MHz, $CDCl_3$): δ = 170.67 (C_q), 170.14 (C_q), 169.63 (C_q), 169.56 (C_q), 140.91 (C_q), 140.02 (C_q), 139.27 (C_q), 128.85 (CH), 128.77 (CH), 127.31 (CH), 127.17 (CH), 127.02 (CH), 72.05 (CH), 70.51 (CH), 70.32 (CH), 68.95 (CH), 68.79 (CH), 60.39 (CH_2), 30.78 (CH_2), 27.31 (CH_2), 20.79 (CH_3), 20.74 (CH_2), 20.71 (CH_2), 20.68 (CH_2).

IR (ATR): ṽ = 1745, 1486, 1366, 1210, 1031, 908, 727, 698, 648 cm^{-1}.
MS (ESI): m/z (relative intensity) 535 (100) $[M+Na]^+$, 1047 (50) [2M+Na].
HR-MS (ESI): *m/z* calcd for $C_{28}H_{32}NaO_9^+$ $[M+Na]^+$: 535.1939, found: 535.1942.

(2*R*,3*S*,4*R*,5*R*,6*R*)-2-{2-([1,1'-biphenyl]-4-yl)ethyl}-6-[(pivaloyloxy)methyl]tetrahydro-2*H*-pyran-3,4,5-triyl tris(2,2-dimethylpropanoate) (19)

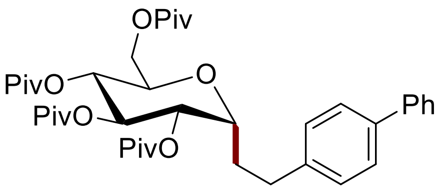

The general procedure **A** was followed using 4-vinyl-1,1'-biphenyl **2aa** (54.0 mg, 0.3 mmol), (2*R*,3*R*,4*S*,5*R*,6*R*)-2-(acetoxymethyl)-6-bromotetrahydro-2*H*-pyran-3,4,5-triyl triacetate **1i** (82.0 mg, 0.2 mmol), Purification by column chromatography on silica gel (*n*hexane/EtOAc: 6/1 to 2/1) yielded **19** (114.3 mg, 84%) as a syrup.

^{1}H NMR (400 MHz, $CDCl_3$): δ = 7.68 – 7.61 (m, 2H), 7.60 – 7.54 (m, 2H), 7.52 – 7.45 (m, 2H), 7.42 – 7.35 (m, 1H), 7.33 – 7.28 (m, 2H), 5.48 (t, *J* = 9.6 Hz, 1H), 5.15 (dd, *J* = 9.9, 6.1 Hz, 1H), 5.09 (dd, *J* = 10.0, 9.2 Hz, 1H), 4.24 (ddd, *J* = 12.1, 5.1, 2.4 Hz, 2H), 4.10 (dd, *J* = 12.1, 6.4 Hz, 1H), 3.96 (ddd, *J* = 10.1, 6.4, 1.9 Hz, 1H), 2.89 (ddd, *J* = 14.2, 9.7, 4.7 Hz, 1H), 2.69 (dt, *J* = 14.0, 8.3 Hz, 1H), 2.26 (dddd, *J* = 14.0, 12.1, 9.0, 4.7 Hz, 1H), 1.98 – 1.73 (m, 1H), 1.30 (s, 9H), 1.23 (s, 9H), 1.19 (s, 9H), 1.17 (s, 9H).

^{13}C NMR (101 MHz, $CDCl_3$): δ = 178.13 (C_q), 177.10 (C_q), 176.93 (C_q), 176.66 (C_q), 140.89 (C_q), 139.85 (C_q), 139.19 (C_q), 128.73 (CH), 128.71 (CH), 127.28 (CH), 127.10 (CH), 126.99 (CH), 71.93 (CH), 70.56 (CH), 70.04 (CH), 68.86 (CH), 68.71 (CH), 62.87 (CH_2), 38.87 (C_q), 38.75 (C_q), 38.70 (C_q), 30.43 (CH_2), 27.22 (CH_3), 27.16 (CH_3), 27.11 (CH_3), 27.07 (CH_3), 26.56 (CH_2).

IR (ATR): ṽ = 1729, 1479, 1460, 1367, 1279, 1128, , 1033, 905, 729, 697, 648cm^{-1}.
MS (ESI): m/z (relative intensity) 703 (100) $[M+Na]^+$, 1383 (25) [2M+Na].
HR-MS (ESI): *m/z* calcd for $C_{28}H_{32}NaO_9^+$ $[M+Na]^+$: 703.3817, found: 703.3818.

(2*R*,3*S*,4*R*,5*R*,6*R*)-2-[2-([1,1'-Biphenyl]-4-yl)ethyl]-6-[(benzoyloxy)methyl]tetrahydro-2*H*-pyran-3,4,5-triyl tribenzoate (20)

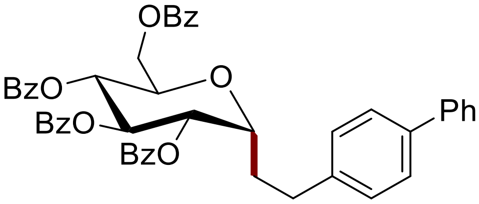

The general procedure **A** was followed using 4-vinyl-1,1'-biphenyl **2aa** (54.0 mg, 0.3 mmol), (2*R*,3*R*,4*S*,5*R*,6*R*)-2-[(benzoyloxy)methyl]-6-bromotetrahydro-2*H*-pyran-3,4,5-triyl tribenzoate **1j** (131.6 mg, 0.2 mmol). Purification by column chromatography on silica gel (*n*hexane/EtOAc: 10/1 to 5/1) yielded **20** (79.1 mg, 52%) as a syrup.

^{1}H NMR (400 MHz, $CDCl_3$): δ = 8.10 (d, J = 7.2 Hz, 2H), 8.00 (d, J = 7.2 Hz, 2H), 7.96 (d, J = 7.2 Hz, 2H), 7.91 (d, J = 7.2 Hz, 2H), 7.60 – 7.54 (m, 4H), 7.50 (d, J = 8.2 Hz, 3H), 7.47 – 7.39 (m, 7H), 7.34 (p, J = 7.5, 7.0 Hz, 6H), 7.25 (d, J = 8.2 Hz, 1H), 6.01 (t, J = 8.6 Hz, 1H), 5.59 (d, J = 8.6 Hz, 1H), 5.56 (dd, J = 7.5, 4.0 Hz, 1H), 4.68 – 4.61 (m, 2H), 4.60 – 4.51 (m, 1H), 4.42 (ddd, J = 9.5, 6.4, 3.3 Hz, 1H), 2.93 (ddd, J = 14.5, 10.2, 4.7 Hz, 1H), 2.75 (ddd, J = 14.0, 9.5, 6.9 Hz, 1H), 2.55 – 2.34 (m, 1H), 2.05 (dddd, J = 14.0, 10.3, 6.9, 3.5 Hz, 1H).

^{13}C NMR (101 MHz, $CDCl_3$): δ = 166.20 (C_q), 165.63 (C_q), 165.35 (C_q), 140.92 (C_q), 139.95 (C_q), 139.13 (C_q), 133.43 (CH), 133.39 (CH), 133.26 (CH), 133.14 (CH), 129.85 (CH), 129.70 (CH), 129.68 (CH), 129.65 (CH), 128.99 (C_q), 128.96 (C_q), 128.88 (C_q), 128.79 (CH), 128.68 (CH), 128.47 (CH), 128.40 (CH), 128.35 (CH), 127.21 (CH), 127.06 (C_q), 126.98 (CH), 71.85 (CH), 71.08 (CH), 70.38 (CH), 69.84 (CH), 69.67 (CH), 63.32 (CH_2), 30.82 (CH_2), 27.90 (CH_2).

IR (ATR): ṽ = 1747, 1396, 1372, 1253, 1115, 1043, 938, 862, 786, 742 cm^1.
MS (ESI): m/z (relative intensity) 783 (100) $[M+Na]^+$, 1543 (25) [2M+Na].
HR-MS (ESI): *m/z* calcd for $C_{40}H_{56}NaO_9^+$ $[M+Na]^+$: 783.2565, found: 783.2560.

(2*R*,3*R*,4*R*,5*R*,6*R*)-2-[2-([1,1'-Biphenyl]-4-yl)ethyl]-6-[(benzoyloxy)methyl]tetrahydro-2*H*-pyran-3,4,5-triyl tribenzoate (21a)

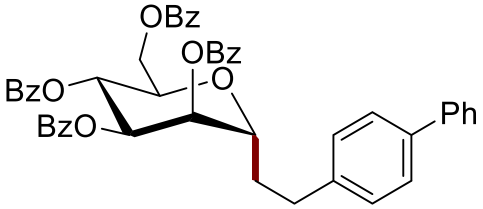

The general procedure **A** was followed using 4-vinyl-1,1'-biphenyl **2aa** (54.0 mg, 0.3 mmol), (2*R*,3*R*,4*S*,5*S*,6*R*)-2-[(benzoyloxy)methyl]-6-bromotetrahydro-2*H*-pyran-3,4,5-triyl tribenzoate **1g** (131.6 mg, 0.2 mmol). Purification by column chromatography on silica gel (*n*hexane/EtOAc: 10/1 to 5/1) yielded **21a** (95.8 mg, 63%) as a syrup.

^{1}H NMR (300 MHz, $CDCl_3$): δ = 8.12 (d, *J* = 7.2 Hz, 2H), 8.05 (d, *J* = 7.2 Hz, 2H), 7.99 (d, *J* = 7.2 Hz, 2H), 7.86 (d, *J* = 7.6 Hz, 2H), 7.63 – 7.50 (m, 8H), 7.47 – 7.34 (m, 10H), 7.33 – 7.26 (m, 4H), 6.02 (t, *J* = 9.1 Hz, 1H), 5.84 (dd, *J* = 9.3, 3.2 Hz, 1H), 5.70 (t, *J* = 2.9 Hz, 1H), 4.74 – 4.58 (m, 2H), 4.42 – 4.27 (m, 2H), 3.06 – 2.77 (m, 2H), 2.54 – 2.32 (m, 1H), 2.26 – 2.07 (m, 1H).

^{13}C NMR (75 MHz, $CDCl_3$): δ = 166.22 (C_q), 165.62 (C_q), 165.45 (C_q), 140.94 (C_q), 139.66 (C_q), 139.23 (C_q), 133.47 (CH), 133.34 (CH), 133.29 (CH), 133.10 (CH), 129.80 (CH), 129.72 (CH), 129.51 (C_q), 128.95 (C_q), 128.93 (C_q), 128.90 (CH), 128.70 (CH), 128.51 (CH), 128.46 (CH), 128.43 (CH), 128.36 (CH), 127.30 (CH), 127.08 (CH), 127.01 (CH), 74.77 (CH), 71.81 (CH), 70.50 (CH), 70.14 (CH), 67.77 (CH), 63.28 (CH_2), 31.16 (CH_2), 30.31 (CH_2).

IR (ATR): ṽ = 1737, 1451, 1372, 1233, 1095, 1043, 938, 846, 756, 712 cm^{1}.
MS (ESI): m/z (relative intensity) 783 (100) $[M+Na]^+$, 1543 (50) [2M+Na].
HR-MS (ESI): *m/z* calcd for $C_{40}H_{56}NaO_9^+$ $[M+Na]^+$: 783.2565, found: 783.2560.

(2*R*,3*R*,4*S*,5*S*,6*S*)-2-[(Benzoyloxy)methyl]-6-hydroxytetrahydro-2*H*-pyran-3,4,5-triyl tribenzoate (21b)

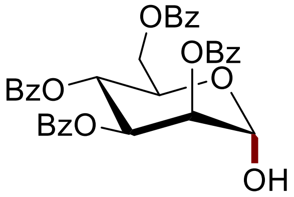

^{1}H NMR (300 MHz, $CDCl_3$) δ = 8.13 (d, J = 6.9 Hz, 1H), 8.03 (d, J = 7.3 Hz, 2H), 7.97 (d, J = 6.9 Hz, 1H), 7.86 (d, J = 6.9 Hz, 1H), 7.64 – 7.45 (m, 4H), 7.44 – 7.33 (m, 6H), 7.33 – 7.24 (m, 2H), 6.20 (t, J = 10.1 Hz, 1H), 6.03 (dd, J = 10.1, 3.2 Hz, 1H), 5.76 (dd, J = 3.2, 1.9 Hz, 1H), 5.55 (dd, J = 4.2, 1.9 Hz, 1H), 4.79 (dd, J = 12.2, 2.7 Hz, 1H), 4.69 (dt, J = 10.0, 3.1 Hz, 1H), 4.45 (dd, J = 12.2, 3.5 Hz, 1H), 4.10 (d, J = 4.2 Hz, 1H, *OH*).

^{13}C NMR (75 MHz, $CDCl_3$): δ = 166.40 (C_q), 165.59 (C_q), 165.50 (C_q), 165.47 (C_q), 133.40 (CH), 133.15 (CH), 133.07 (CH), 129.79 (CH), 129.72 (CH), 129.25 (C_q), 129.04 (C_q), 128.98 (C_q), 128.52 (CH), 128.42 (CH), 128.27 (CH), 92.30 (CH), 70.92 (CH), 69.83 (CH), 68.79 (CH), 66.85 (CH), 62.71 (CH_2).

IR (ATR): ṽ = 1720, 1602, 1452, 1263, 1093, 1068, 1062, 907, 706, 685 cm^{-1}.
MS (ESI): m/z (relative intensity) 619 (100) $[M+Na]^+$.
HR-MS (ESI): *m/z* calcd for $C_{34}H_{28}NaO_{10}^+$ $[M+Na]^+$: 619.1575, found: 619.1579.

(2*S*,3*R*,4*R*,5*R*,6*S*)-2-[2-([1,1'-Biphenyl]-4-yl)ethyl]-6-methyltetrahydro-2*H*-pyran-3,4,5-triyl tribenzoate (22)

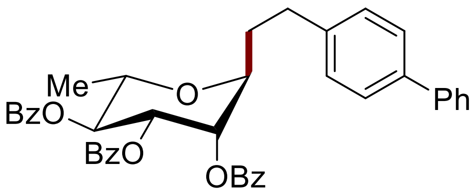

The general procedure **A** was followed using 4-vinyl-1,1'-biphenyl **2aa** (54.0 mg, 0.3 mmol), (2*S*,3*R*,4*R*,5*S*,6*S*)-2-bromo-6-methyltetrahydro-2*H*-pyran-3,4,5-triyl tribenzoate **1k** (107.6 mg, 0.2 mmol). Purification by column chromatography on silica gel (*n*hexane/EtOAc: 10/1 to 5/1) yielded **22** (79.4 mg, 62%) as a syrup.

^{1}H NMR (400 MHz, $CDCl_3$): δ = 8.08 (d, J = 7.2 Hz, 2H), 8.00 (d, J = 7.2 Hz, 2H), 7.84 (d, J = 7.2 Hz, 2H), 7.64 – 7.54 (m, 5H), 7.52 (d, J = 7.4 Hz, 1H), 7.50 – 7.40 (m, 7H), 7.39 – 7.33 (m, 3H), 7.33 – 7.27 (m, 2H), 5.76 (dd, J = 9.5, 3.3 Hz, 1H), 5.67 (dd, J = 5.6, 2.9 Hz, 2H), 4.24 (ddd, J = 10.5, 4.6, 2.4 Hz, 1H), 4.10 (dq, J = 8.5, 6.2 Hz, 1H), 2.94 (ddd, J = 14.5, 9.3, 5.4 Hz, 1H), 2.84 (ddd, J = 14.0, 8.8, 7.2 Hz, 1H), 2.52 – 2.32 (m, 1H), 2.12 (dddd, J = 14.0, 9.3, 7.3, 4.5 Hz, 1H), 1.42 (d, J = 6.2 Hz, 3H).

^{13}C NMR (101 MHz, $CDCl_3$): δ = 165.80 (C_q), 165.70 (C_q), 165.67 (C_q), 141.00 (C_q), 139.91 (C_q), 139.19 (C_q), 133.30 (CH), 133.16 (CH), 129.85 (CH), 129.71 (CH), 129.67 (CH), 129.34 (C_q), 129.14 (C_q), 128.94 (CH), 128.70 (CH), 128.48 (CH), 128.42 (CH), 128.30 (CH), 127.27 (CH), 127.07 (C_q), 127.01 (CH), 74.72 (CH), 72.35 (CH), 72.22 (CH), 70.26 (CH), 68.26 (CH), 31.46 (CH_2), 30.50 (CH_2), 18.02 (CH_3).

IR (ATR): ṽ =1722, 1601, 1451, 1263, 1174, 1094, 1069, 1027, 708 cm^{-1}.
MS (ESI): m/z (relative intensity) 663 (100) $[M+Na]^+$, 1303 (50) [2M+Na].
HR-MS (ESI): *m/z* calcd for $C_{41}H_{36}NaO_7^+$ $[M+Na]^+$: 663.2353, found: 663.2356.

(2*S*,3*R*,4*R*,5*R*,6*S*)-2-[2-([1,1'-Biphenyl]-4-yl)ethyl]-6-methyltetrahydro-2*H*-pyran-3,4,5-triyl triacetate (23)

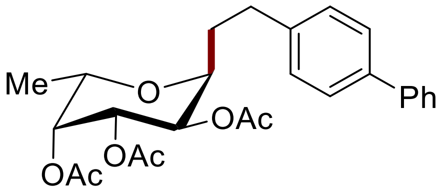

The general procedure **A** was followed using 4-vinyl-1,1'-biphenyl **2aa** (54.0 mg, 0.3 mmol), (2*S*,3*S*,4*R*,5*R*,6*S*)-2-bromo-6-methyltetrahydro-2*H*-pyran-3,4,5-triyl triacetate **1l** (70.4 mg, 0.2 mmol). Purification by column chromatography on silica gel (*n*hexane/EtOAc: 10/1 to 5/1) yielded **23** (64.5 mg, 71%) as a syrup.

^{1}H NMR (400 MHz, $CDCl_3$): δ = 7.58 (d, J = 7.4 Hz, 2H), 7.53 (d, J = 8.0 Hz, 2H), 7.43 (t, J = 7.6 Hz, 2H), 7.33 (t, J = 7.3 Hz, 1H), 7.27 (d, J = 8.3 Hz, 2H), 5.34 (dd, J = 10.0, 5.8 Hz, 1H), 5.28 (dd, J = 3.5, 1.8 Hz, 1H), 5.22 (dd, J = 10.1, 3.4 Hz, 1H), 4.23 (ddd, J = 11.7, 5.8, 3.8 Hz, 1H), 4.00 (qd, J = 6.4, 1.8 Hz, 1H), 2.81 (ddd, J = 14.5, 9.7, 5.2 Hz, 1H), 2.66 (ddd, J = 14.1, 9.0, 7.1 Hz, 1H), 2.16 (s, 3H), 2.13 – 2.10 (m, 1H), 2.04 (s, 3H), 2.00 (s, 3H), 1.79 (dddd, J = 13.8, 10.0, 7.1, 3.6 Hz, 1H), 1.17 (d, J = 6.4 Hz, 3H).

^{13}C NMR (101 MHz, $CDCl_3$): δ = 170.55 (C_q), 170.14 (C_q), 169.87 (C_q), 140.96 (C_q), 140.32 (C_q), 139.11 (C_q), 128.82 (CH), 128.71 (CH), 127.20 (CH), 127.08 (CH), 126.98 (CH), 72.02 (CH), 70.76 (CH), 68.68 (CH), 68.25 (CH), 65.58 (CH_2), 31.41 (CH_2), 27.19 (CH_2), 20.82 (CH_3), 20.71 (CH_3), 20.67 (CH_3), 16.04 (CH_3).

IR (ATR): $\tilde{v}$ =1745, 1488, 1369, 1246, 1213, 1055, 1020, 936, 763, 699 cm^{-1}.
MS (ESI): m/z (relative intensity) 477 (100) $[M+Na]^+$, 931 (50) [2M+Na].
HR-MS (ESI): *m/z* calcd for $C_{26}H_{30}NaO_7^+$ $[M+Na]^+$: 477.1884, found: 477.1883.

(2*R*,3*R*,4*R*,5*R*,6*R*)-2-[(Benzoyloxy)methyl]-6-[2-(phenylsulfonyl)ethyl]tetrahydro-2*H*-pyran-3,4,5-triyl tribenzoate (24)

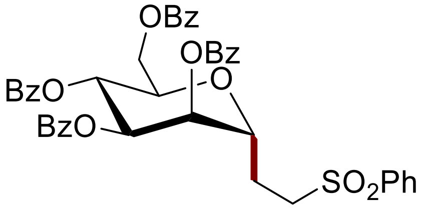

The general procedure **A** was followed using (vinylsulfonyl)benzene **2ap** (50.4 mg, 0.3 mmol), (2*R*,3*R*,4*S*,5*S*,6*R*)-2-[(benzoyloxy)methyl]-6-bromotetrahydro-2*H*-pyran-3,4,5-triyl tribenzoate **1g** (131.6 mg, 0.2 mmol). Purification by column chromatography on silica gel (*n*hexane/EtOAc: 10/1 to 5/1) yielded **24** (76 mg, 51%) as a syrup.

^{1}H NMR (300 MHz, $CDCl_3$): δ = 8.03 (ddd, *J* = 8.6, 2.2, 1.3 Hz, 4H), 7.99 – 7.84 (m, 6H), 7.68 – 7.48 (m, 7H), 7.47 – 7.32 (m, 8H), 5.99 – 5.75 (m, 2H), 5.55 (dd, *J* = 4.8, 2.5 Hz, 1H), 4.87 (dd, *J* = 12.1, 7.1 Hz, 1H), 4.49 (dd, *J* = 12.1, 3.1 Hz, 1H), 4.35 (dt, *J* = 11.4, 4.1 Hz, 2H), 3.50 – 3.11 (m, 2H), 2.49 – 2.30 (m, 1H), 2.28 – 2.10 (m, 1H).

^{13}C NMR (101 MHz, $CDCl_3$): δ = 166.15 (C_q), 165.47 (C_q), 165.38 (C_q), 165.33 (C_q), 138.82 (C_q), 133.88 (CH), 133.71 (CH), 133.58 (CH), 133.56 (CH), 133.25 (CH), 129.93 (CH), 129.80 (CH), 129.78 (CH), 129.69 (CH), 129.57 (C_q), 129.45 (CH), 129.12 (C_q), 128.81 (C_q), 128.80 (C_q), 128.59 (CH), 128.58 (CH), 128.56 (CH), 128.02 (CH), 71.84 (CH), 71.47 (CH), 70.76 (CH), 69.16 (CH), 67.84 (CH), 62.05 (CH_2), 52.32 (CH_2), 22.88 (CH_2).

IR (ATR): ṽ = 1721, 1601, 1451, 1266, 1148, 1093, 1070 1027, 709, 687 cm^{-1} .
MS (ESI): m/z (relative intensity) 771 (100) $[M+Na]^+$, 1519 (20) $[2M+Na]^+$.
HR-MS (ESI): *m/z* calcd for $C_{42}H_{36}NaO_{11}S^+$ $[M+Na]^+$: 771.1871, found: 771.1879.

(2*R*,3*S*,4*R*,5*S*,6*R*)-2-(Acetoxymethyl)-6-{2-[(8*S*,9*R*,13*S*)-13-methyl-17-oxo-7,8,9,11,12,13,14,15,16,17-decahydro-6*H*-cyclopenta[*a*]phenanthren-4-yl]ethyl}tetrahydro-2*H*-pyran-3,4,5-triyl triacetate (25)

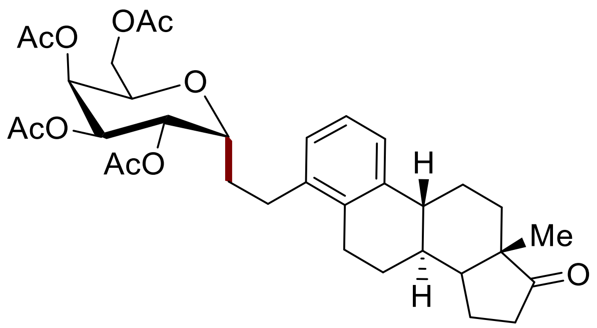

The general procedure **A** was followed using (*8S*,*9R*,*13S*)-13-methyl-4-vinyl-6,7,8,9,11,12,13,14,15,16-decahydro-17*H*-cyclopenta[*a*]phenanthren-17-one **2aq** (84.0 mg, 0.3 mmol), (2*R*,3*S*,4*S*,5*R*,6*R*)-2-(acetoxymethyl)-6-bromotetrahydro-2*H*-pyran-3,4,5-triyl triacetate **1a** (82.0 mg, 0.2 mmol). Purification by column chromatography on silica gel (*n*hexane/EtOAc: 6/1 to 2/1) yielded **25** (57.6 mg, 47%) as a syrup.

^{1}H NMR (400 MHz, $CDCl_3$): δ = 7.23 (dd, J = 8.0, 1.0 Hz, 1H), 6.98 (dd, J = 8.0, 2.0 Hz, 1H), 6.92 (d, J = 2.0 Hz, 1H), 5.43 (t, J = 3.3, 2.0 Hz, 1H), 5.29 (dd, J = 9.6, 5.3 Hz, 1H), 5.20 (dd, J = 9.6, 3.3 Hz, 1H), 4.30 – 4.17 (m, 2H), 4.15 – 4.06 (m, 2H), 2.90 (dd, J = 8.9, 4.2 Hz, 2H), 2.71 (ddd, J = 14.6, 10.2, 4.8 Hz, 1H), 2.60 – 2.38 (m, 3H), 2.34 – 2.24 (m, 1H), 2.21 – 2.16 (m, 1H), 2.13 (s, 3H), 2.07 (s, 3H), 2.04 (s, 3H), 2.03 – 2.02 (m, 1H), 2.01 (s, 3H), 1.99 – 1.95 (m, 1H), 1.78 – 1.69 (m, 1H), 1.67 – 1.39 (m, 8H), 0.91 (s, 3H).

^{13}C NMR (101 MHz, $CDCl_3$): δ = 220.93 (C_q), 170.58 (C_q), 170.16 (C_q), 170.03 (C_q), 169.86 (C_q), 138.61 (C_q), 137.65 (C_q), 136.66 (C_q), 129.05 (CH), 125.86 (CH), 125.60 (CH), 71.70 (CH), 68.30 (CH), 68.12 (CH), 68.01 (CH), 67.76 (CH), 61.75 (CH_2), 50.52 (CH), 48.03 (C_q), 44.32 (CH), 38.23 (CH), 35.89 (CH_2), 31.62 (CH_2), 30.90 (CH_2), 29.42 (CH_2), 27.77 (CH_2), 26.55 (CH_2), 25.76 (CH_2), 21.61 (CH_2), 20.85 (CH_3), 20.80 (CH_3), 20.75 (CH_3), 20.71 (CH_3), 13.87 (CH_3).

IR (ATR): $\tilde{\nu}$ =1748, 1735, 1369, 1212, 1109, 1048, 909, 822, 728 cm^{-1}.
MS (ESI): m/z (relative intensity) 635 (100) $[M+Na]^+$, 1247 (50) [2M+Na].
HR-MS (ESI): *m/z* calcd for $C_{34}H_{44}NaO_{10}^+$ $[M+Na]^+$: 635.2827, found: 635.2827.

(2*R*,3*S*,4*R*,5*S*,6*R*)-2-(Acetoxymethyl)-6-[4-({[(1*S*,2*R*,5*S*)-2-isopropyl-5-methylcyclohexyl]oxy}carbonyl)phenethyl]tetrahydro-2*H*-pyran-3,4,5-triyl triacetate (26)

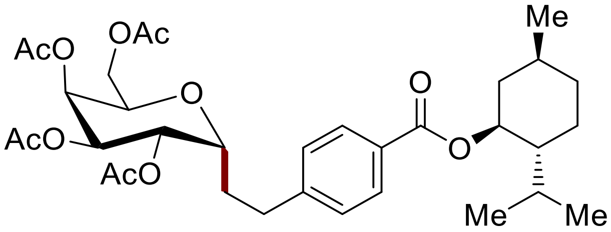

The general procedure **A** was followed using (1*S*,2*R*,5*S*)-2-isopropyl-5-methylcyclohexyl 4-vinylbenzoate **2ar** (85.8 mg, 0.3 mmol), (2*R*,3*S*,4*S*,5*R*,6*R*)-2-(acetoxymethyl)-6-bromotetrahydro-2*H*-pyran-3,4,5-triyl triacetate **1a** (82.0 mg, 0.2 mmol). Purification by column chromatography on silica gel (*n*hexane/EtOAc: 6/1 to 2/1) yielded **26** (77.9 mg, 63%) as a syrup.

^{1}H NMR (400 MHz, $CDCl_3$) δ 7.96 (d, *J* = 8.2 Hz, 2H), 7.25 (d, *J* = 8.5 Hz, 2H), 5.42 (dd, *J* = 3.3, 2.3 Hz, 1H), 5.27 (dd, *J* = 9.3, 5.1 Hz, 1H), 5.18 (dd, *J* = 9.3, 3.3 Hz, 1H), 4.91 (td, *J* = 10.8, 4.4 Hz, 1H), 4.36 – 4.15 (m, 2H), 4.16 – 4.04 (m, 2H), 2.82 (ddd, *J* = 14.4, 9.9, 4.8 Hz, 1H), 2.65 (ddd, *J* = 13.8, 9.3, 7.2 Hz, 1H), 2.12 (s, 3H), 2.11 – 2.08 (m, 1H), 2.07 (s, 3H), 2.04 (s, 3H), 2.03 – 2.01 (m, 1H), 2.00 (s, 3H), 1.98 – 1.92 (m, 1H), 1.78 – 1.67 (m, 3H), 1.62 – 1.48 (m, 2H), 1.18 – 1.02 (m, 2H), 0.98 – 0.93 (m, 1H), 0.92 (d, *J* = 4.8 Hz, 3H), 0.90 (d, *J* = 5.2 Hz, 3H), 0.78 (d, *J* = 7.0 Hz, 3H).

^{13}C NMR (101 MHz, $CDCl_3$): δ = 170.49 (C_q), 170.02 (C_q), 169.87 (C_q), 169.71 (C_q), 165.89 (C_q), 146.23 (C_q), 129.82 (CH), 128.88 (C_q), 128.35 (CH), 74.66 (CH), 71.07 (CH), 68.22 (CH), 67.97 (CH), 67.51 (CH), 61.54 (CH_2), 47.22 (CH), 40.93 (CH_2), 34.28 (CH_2), 31.39 (CH_2), 31.33 (CH), 27.53 (CH_2), 26.47 (CH), 23.61 (CH_2), 22.00 (CH_3), 20.72 (2 CH_3), 20.66 (CH_3), 20.62 (CH_3), 16.50 (CH_3).

IR (ATR): ṽ = 1745, 1738, 1711, 1611, 1368, 1274, 1210, 1109, 917, 731cm^{-1}.
MS (ESI): m/z (relative intensity) 641 (100) [M+Na]$^{+}$, 1259 (50) [2M+Na].
HR-MS (ESI): *m/z* calcd for $C_{33}H_{46}NaO_{11}^{+}$ [M+Na]$^{+}$: 641.2932, found: 641.2939.

(2*R*,3*S*,4*R*,5*S*,6*R*)-2-(Acetoxymethyl)-6-[4-({[(3*aR*,5*R*,6*S*,6*aR*)-5-[(*R*)-2,2-dimethyl-1,3-dioxolan-4-yl]-2,2-dimethyltetrahydrofuro[2,3-*d*][1,3]dioxol-6-yl]oxy}carbonyl)phenethyl]tetrahydro-2*H*-pyran-3,4,5-triyl triacetate (27)

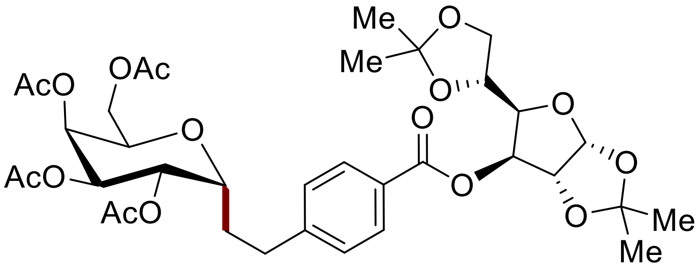

The general procedure **A** was followed using (3*aR*,5*R*,6*S*,6*aR*)-5-[(*R*)-2,2-dimethyl-1,3-dioxolan-4-yl]-2,2-dimethyltetrahydrofuro[2,3-*d*][1,3]dioxol-6-yl 4-vinylbenzoate **2as** (117.0 mg, 0.3 mmol), (2*R*,3*S*,4*S*,5*R*,6*R*)-2-(acetoxymethyl)-6-bromotetrahydro-2*H*-pyran-3,4,5-triyl triacetate **1a** (82.0 mg, 0.2 mmol). Purification by column chromatography on silica gel (*n*hexane/EtOAc: 6/1 to 2/1) yielded **27** (80.9 mg, 56%) as a syrup.

^{1}H NMR (400 MHz, $CDCl_3$): δ = 7.95 (d, *J* = 8.2 Hz, 2H), 7.27 (d, *J* = 8.2 Hz, 2H), 5.94 (d, *J* = 3.7 Hz, 1H), 5.48 (d, *J* = 2.6 Hz, 1H), 5.42 (t, *J* = 2.8 Hz, 1H), 5.27 (dd, *J* = 9.3, 5.1 Hz, 1H), 5.18 (dd, *J* = 9.3, 3.3 Hz, 1H), 4.62 (d, *J* = 3.7 Hz, 1H), 4.40 – 4.30 (m, 2H), 4.29 – 4.22 (m, 1H), 4.19 (ddd, *J* = 11.5, 5.2, 3.6 Hz, 1H), 4.14 – 4.05 (m, 4H), 2.83 (ddd, *J* = 14.4, 9.8, 4.8 Hz, 1H), 2.67 (ddd, *J* = 14.0, 9.3, 7.3 Hz, 1H), 2.12 (s, 3H), 2.07 (s, 3H), 2.04 (s, 3H), 2.03 (d, *J* = 3.6 Hz, 1H), 2.01 (s, 3H), 1.72 (dddd, *J* = 13.8, 10.3, 7.3, 3.5 Hz, 1H), 1.55 (s, 3H), 1.41 (s, 3H), 1.31 (s, 3H), 1.27 (s, 3H).

^{13}C NMR (101 MHz, $CDCl_3$): δ = 170.51 (C_q), 170.04 (C_q), 169.90 (C_q), 169.73 (C_q), 165.03 (C_q), 147.20 (C_q), 130.02 (CH), 128.63 (CH), 127.57 (C_q), 112.33 (C_q), 109.35 (C_q), 105.11 (CH), 83.36 (CH), 79.92 (CH), 76.52 (CH), 72.57 (CH), 71.03 (CH), 68.27 (CH), 68.23 (CH), 67.96 (CH), 67.50 (CH), 67.19 (CH), 61.86 (CH_2), 61.52 (CH_2), 31.41 (CH_2), 27.46 (CH_2), 26.81 (CH_3), 26.72 (CH_3), 26.19 (CH_3), 25.22 (CH_3), 20.76 (CH_3), 20.70 (CH_3), 20.65 (CH_3).

IR (ATR): $\tilde{\nu}$ = 1747, 1720, 1611, 1370, 1267, 1213, 1165, 1111, 1076, 733 cm^{-1}.
MS (ESI): m/z (relative intensity) 745 (100) $[M+Na]^+$.
HR-MS (ESI): *m/z* calcd for $C_{33}H_{46}NaO_{11}^+$ $[M+Na]^+$: 745.2678, found: 745.2682.

(2*R*,3*R*,4*R*,5*R*,6*R*)-2-[(Benzoyloxy)methyl]-6-(3-oxo-3-{[(3aR,5R,5aS,8aS,8bR)-2,2,7,7-tetramethyltetrahydro-5*H*-bis([1,3]dioxolo)[4,5-b:4',5'-d]pyran-5-yl]methoxy}propyl)tetrahydro-2*H*-pyran-3,4,5-triyl tribenzoate (28)

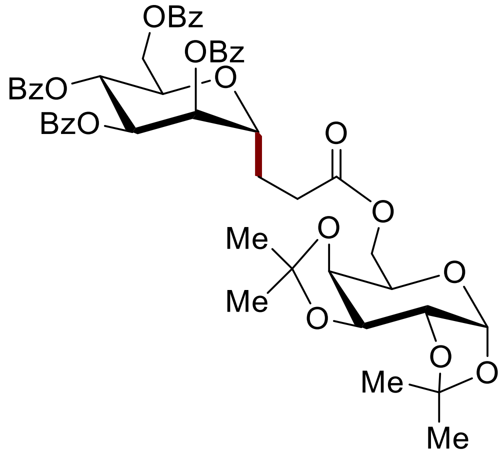

The general procedure **A** was followed using [(*3aR*,*5R*,*5aS*,*8aS*,*8bR*)-2,2,7,7-tetramethyltetrahydro-5*H*-bis([1,3]dioxolo)[4,5-*b*:4',5'-*d*]pyran-5-yl]methyl acrylate **2at** (94.2 mg, 0.3 mmol), (2*R*,3*R*,4*S*,5*S*,6*R*)-2-[(benzoyloxy)methyl]-6-bromotetrahydro-2*H*-pyran-3,4,5-triyl tribenzoate **1g** (131.6 mg, 0.2 mmol). Purification by column chromatography on silica gel (*hexane*/EtOAc: 10/1 to 5/1) yielded **28** (96.6 mg, 54%) as a syrup.

^{1}H NMR (400 MHz, $CDCl_3$): δ = 8.10 (dd, J = 8.4, 1.3 Hz, 2H), 8.06 – 8.02 (m, 2H), 7.98 (dd, J = 8.3, 1.2 Hz, 2H), 7.86 (dd, J = 8.4, 1.4 Hz, 2H), 7.61 – 7.53 (m, 2H), 7.53 – 7.47 (m, 1H), 7.47 – 7.35 (m, 7H), 7.30 (t, J = 7.8 Hz, 2H), 6.04 (t, J = 9.0 Hz, 1H), 5.81 (dd, J = 9.2, 3.3 Hz, 1H), 5.66 (t, J = 3.0 Hz, 1H), 5.52 (d, J = 5.0 Hz, 1H), 4.75 – 4.57 (m, 3H), 4.47 – 4.28 (m, 4H), 4.27 – 4.15 (m, 2H), 4.04 (ddd, J = 7.8, 4.6, 1.9 Hz, 1H), 2.73 – 2.51 (m, 2H), 2.44 (ddt, J = 14.1, 11.2, 6.9 Hz, 1H), 2.22 – 2.05 (m, 1H), 1.49 (s, 3H), 1.45 (s, 3H), 1.33 (s, 3H), 1.27 (s, 3H).

^{13}C NMR (101 MHz, $CDCl_3$): δ = 172.54 (C_q), 166.13 (C_q), 165.53 (C_q), 165.49 (C_q), 165.35 (C_q), 133.44 (CH), 133.31 (CH), 133.27 (CH), 133.00 (CH), 129.82 (C_q), 129.78 (CH), 129.76 (CH), 129.73 (CH), 129.71 (CH), 129.50 (C_q), 128.96 (C_q), 128.93 (Cq), 128.49 (CH), 128.44 (CH), 128.40 (CH), 128.35 (CH), 109.63 (C_q), 108.77 (C_q), 96.24 (CH), 74.83 (CH), 71.66 (CH), 70.99 (CH), 70.68 (CH), 70.46 (CH), 70.40 (CH), 70.02 (CH), 67.41 (CH), 65.88 (CH), 63.65(CH_2), 62.81 (CH_2), 30.35 (CH_2), 25.98 (CH_3), 25.94 (CH_3), 24.82 (CH_3), 24.46 (CH_3), 23.88 (CH_2).

IR (ATR): $\tilde{v}$ = 1729, 1315, 1266, 1212, 1178, 1108, 1069, 1027, 1002, 710 cm^{-1} .

MS (ESI): m/z (relative intensity) 917 (100) [M+Na]$^+$.

HR-MS (ESI): *m/z* calcd for $C_{49}H_{50}NaO_{16}^+$ [M+Na]$^+$: 917.2991, found: 917.2990.

(2*R*,3*R*,4*R*,5*R*,6*R*)-2-[2-([1,1'-biphenyl]-4-yl)ethyl]-6-({[2-(4-isobutylphenyl)propanoyl]oxy}methyl)tetrahydro-2*H*-pyran-3,4,5-triyl triacetate (29)

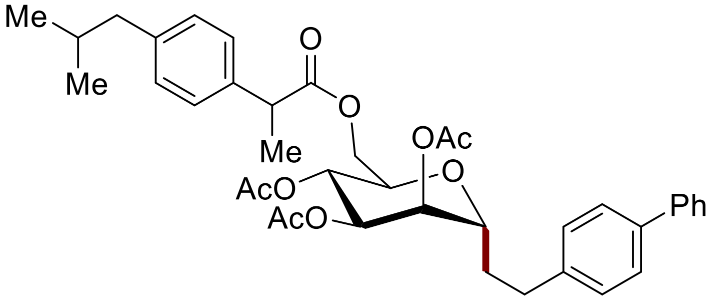

The general procedure **A** was followed using 4-vinyl-1,1'-biphenyl **2aa** (54.0 mg, 0.3 mmol), (2*R*,3*S*,4*S*,5*R*,6*R*)-2-bromo-6-({[2-(4-isobutyl phenyl)propanoyl]oxy}methyl)tetrahydro-2*H*-pyran-3,4,5-triyl triacetate **1am** (111.2 mg, 0.2 mmol). Purification by column chromatography on silica gel (*n*hexane/EtOAc: 6/1 to 1/1) yielded **29** (80.3 mg, 61%) as a syrup.

^{1}H NMR (400 MHz, $CDCl_3$): δ = 7.59 (dt, *J* = 8.2, 1.2 Hz, 2H), 7.52 (dd, *J* = 8.2, 3.9 Hz, 2H), 7.44 (t, *J* = 7.6 Hz, 2H), 7.37 – 7.31 (m, 1H), 7.22 (ddd, *J* = 8.0, 7.2, 2.0 Hz, 4H), 7.05 (d, *J* = 8.0 Hz, 2H), 5.24 (dt, *J* = 8.8, 3.6 Hz, 1H), 5.20 – 5.10 (m, 2H), 4.44 – 4.09 (m, 2H), 3.93 (dtd, *J* = 15.0, 7.5, 3.7 Hz, 2H), 3.77 (qd, *J* = 7.2, 2.7 Hz, 1H), 2.74 (dddd, *J* = 14.1, 9.3, 5.0, 1.8 Hz, 1H), 2.66 – 2.53 (m, 1H), 2.38 (dd, *J* = 7.2, 4.4 Hz, 2H), 2.09 (s, 3H), 2.05 (s, 3H), 2.00 (s, 3H), 1.54 (d, *J* = 1.5 Hz, 1.5H), 1.52 (d, *J* = 1.5 Hz, 1.5H), 0.87 (d, *J* = 2.0 Hz, 3H), 0.86 (d, *J* = 2.0 Hz, 3H).

^{13}C NMR (101 MHz, $CDCl_3$): δ = 174.46 (C_q), 174.44 (C_q), 170.23 (C_q), 170.21 (C_q), 169.95 (C_q), 169.71 (C_q), 169.67 (C_q), 140.97 (C_q), 140.69 (C_q), 140.67 (C_q), 139.80 (C_q), 139.21 (C_q), 139.19 (C_q), 137.34 (C_q), 137.27 (C_q), 129.40 (CH), 129.38 (CH), 129.02 (CH), 129.01 (CH), 128.77 (CH), 127.28 (CH), 127.24 (CH), 127.15 (CH), 127.02 (CH), 73.51 (CH), 73.35 (CH), 70.66 (CH), 70.62 (CH), 68.96 (CH), 67.40 (CH), 67.23 (CH), 63.15 (CH_2), 62.98 (CH_2), 45.20 (CH), 45.08 (CH), 45.02 (CH_2), 30.83 (CH_2), 30.81 (CH_2), 30.62 (CH_2), 30.54 (CH_2), 30.15 (CH), 22.41 (CH_3), 20.96 (CH_3), 20.81 (CH_3), 20.73 (CH_3), 20.71 (CH_3), 18.65 (CH_3), 18.60 (CH_3).

IR (ATR): ṽ = 1736, 1486, 1368, 1247, 1221, 1047, 910, 846, 731, 699 cm1.

MS (ESI): m/z (relative intensity) 681 (100) [M+Na]+,1339 (25) [2M+Na].

HR-MS (ESI): *m/z* calcd for $C_{33}H_{46}NaO_{11}^+$ [M+Na]+: 681.3034, found: 681.3036.

(2*R*,3*R*,4*R*,5*R*,6*R*)-2-[2-([1,1'-biphenyl]-4-yl)ethyl]-6-({[2-(4-(2,2-dichlorocyclopropyl)phenoxy)-2-methylpropanoyl]oxy}methyl)tetrahydro-2*H*-pyran-3,4,5-triyl triacetate (30)

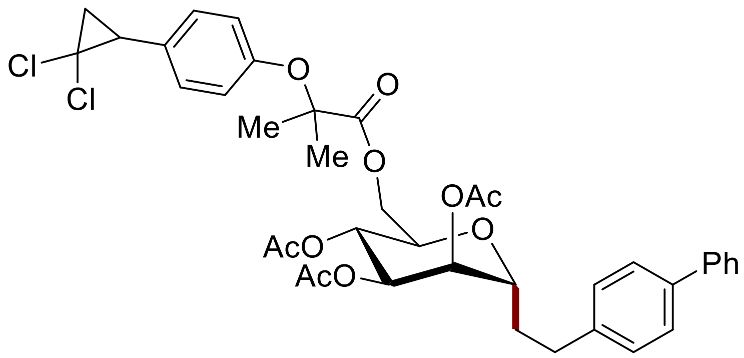

The general procedure **A** was followed using 4-vinyl-1,1'-biphenyl **2aa** (54.0 mg, 0.3 mmol), (2*R*,3*S*,4*S*,5*R*,6*R*)-2-bromo-6-[({2-[4-(2,2-dichlorocyclopropyl)phenoxy]-2-methylpropanoyl}oxy)methyl]tetrahydro-2*H*-pyran-3,4,5-triyl triacetate **1n** (127.6 mg, 0.2 mmol). Purification by column chromatography on silica gel (*n*hexane/EtOAc: 6/1 to 1/1) yielded **30** (122.9 mg, 83%) as a syrup.

^{1}H NMR (400 MHz, $CDCl_3$): δ = 7.60 – 7.54 (m, 2H), 7.51 (d, *J* = 8.2 Hz, 2H), 7.43 (dd, *J* = 8.5, 6.9 Hz, 2H), 7.39 – 7.30 (m, 1H), 7.26 – 7.22 (m, 2H), 7.07 (d, *J* = 8.6 Hz, 2H), 6.92 – 6.82 (m, 2H), 5.41 – 5.12 (m, 3H), 4.49 – 4.28 (m, 2H), 4.12 – 3.79 (m, 2H), 2.84 – 2.74 (m, 2H), 2.71 – 2.58 (m, 1H), 2.15 – 2.10 (m, 1H), 2.08 (s, 3H), 2.05 (d, *J* = 2.4 Hz, 3H), 2.02 (s, 3H), 1.88 (ddd, *J* = 10.7, 7.4, 1.6 Hz, 2H), 1.73 (ddd, *J* = 8.3, 7.4, 2.8 Hz, 1H), 1.65 (s, 3H), 1.63 (s, 3H).

^{13}C NMR (101 MHz, $CDCl_3$): δ = 173.77 (C_q), 170.14 (C_q), 169.93 (C_q), 169.63 (C_q), 154.74 (C_q), 140.84 (C_q), 139.61 (C_q), 139.16 (C_q), 129.56 (CH), 128.93 (CH), 128.72 (CH), 128.38 (C_q), 127.24 (CH), 127.11 (CH), 126.96 (CH), 119.29 (CH), 79.20 (C_q), 73.77 (CH), 70.59 (CH), 70.45 (CH), 68.96 (CH), 67.01 (CH), 63.29 (CH_2), 60.83 (C_q), 34.78 (CH), 30.83 (CH_2), 30.30 (CH_2), 25.78 (CH), 25.45 (CH_3), 25.41 (CH), 25.36 (CH_3), 20.87 (CH_3), 20.78 (CH_3), 20.69 (CH_3).

IR (ATR): ṽ = 1745, 1611, 1510, 1368, 1212, 1046, 907, 833, 725, 698 cm^{-1}.

MS (ESI): m/z (relative intensity) 763 (100) [M+Na]$^+$,1505 (50) [2M+Na].
HR-MS (ESI): *m/z* calcd for $C_{40}H_{48}NaO_{17}^+$ [M+Na]$^+$: 763.2047, found: 763.2050.

(2*R*,3*R*,4*R*,5*R*,6*R*)-2-[2-([1,1'-biphenyl]-4-yl)ethyl]-6-({[3-(4,5-diphenyloxazol-2-yl)propanoyl]oxy}methyl)tetrahydro-2*H*-pyran-3,4,5-triyl triacetate (31)

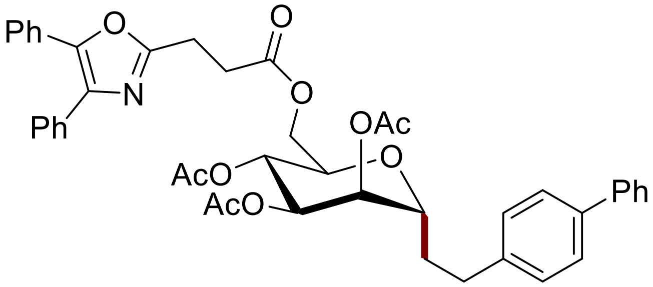

The general procedure **A** was followed using 4-vinyl-1,1'-biphenyl **2aa** (54.0 mg, 0.3 mmol), (2*R*,3*S*,4*S*,5*R*,6*R*)-2-bromo-6-({[3-(4,5-diphenyloxazol-2 yl)propanoyl]oxy}methyl)tetrahydro-2*H*-pyran-3,4,5-triyl triacetate **1o** (128.6 mg, 0.2 mmol). Purification by column chromatography on silica gel (*n*hexane/EtOAc: 6/1 to 1/1) yielded **31** (107.3 mg, 72%) as a syrup.

^{1}H NMR (400 MHz, $CDCl_3$) δ 7.67 (d, *J* = 1.7 Hz, 1H), 7.65 (d, *J* = 1.4 Hz, 1H), 7.60 (dt, *J* = 7.7, 1.4 Hz, 3H), 7.55 (d, *J* = 8.2 Hz, 2H), 7.50 – 7.43 (m, 2H), 7.41 – 7.35 (m, 7H), 7.30 (d, *J* = 8.2 Hz, 2H), 5.33 – 5.27 (m, 2H), 5.24 (t, *J* = 2.9 Hz, 1H), 4.45 (dd, *J* = 12.0, 6.5 Hz, 1H), 4.27 (dd, *J* = 12.0, 2.7 Hz, 1H), 4.01 (dddd, *J* = 11.8, 8.6, 5.6, 3.6 Hz, 2H), 3.27 (dd, *J* = 8.1, 6.3 Hz, 2H), 3.05 (dd, *J* = 8.5, 6.6 Hz, 2H), 2.85 (ddd, *J* = 14.2, 9.2, 5.2 Hz, 1H), 2.73 (dt, *J* = 13.9, 8.1 Hz, 1H), 2.24 – 2.17 (m, 1H), 2.16 (s, 3H), 2.11 (s, 3H), 2.06 (s, 3H), 1.96 – 1.85 (m, 1H).

^{13}C NMR (101 MHz, $CDCl_3$): *δ* = 171.70 (C_q), 170.26 (C_q), 169.97 (C_q), 169.65 (C_q), 161.49 (C_q), 145.44 (C_q), 140.88 (C_q), 139.59 (C_q), 139.21 (C_q), 135.12 (C_q), 132.41 (CH), 128.93 (CH), 128.91 (CH), 128.71 (CH), 128.62 (CH), 128.52 (CH), 128.44 (CH), 128.04 (C_q), 127.86 (CH), 127.28 (CH), 127.09 (CH), 126.98 (CH), 126.46 (CH), 74.09 (CH), 70.64 (CH), 70.39 (CH), 69.04 (CH), 66.98 (CH), 62.98 (CH_2), 30.99 (CH_2), 30.95 (CH_2), 30.27 (CH_2), 23.44 (CH_3), 20.98 (CH_3), 20.75 (CH_3), 20.70 (CH_3).

IR (ATR): ṽ =1745, 1486, 1448, 1369, 1248, 1223, 1053, 914, 764, 696 cm^{-1}.
MS (ESI): m/z (relative intensity) 800 (100) [M+Na]$^+$.
HR-MS (ESI): *m/z* calcd for $C_{44}H_{43}NNaO_{10}^+$ [M+Na]$^+$: 800.2677, found: 800.2683.

(2*R*,3*R*,4*R*,6*S*)-6-{[(2*R*,3*R*,4*S*,5*S*,6*R*)-6-{2-([1,1'-biphenyl]-4-yl)ethyl}-4,5-bis(benzoyloxy)-2-[(benzoyloxy)methyl]tetrahydro-2*H*-pyran-3-yl]oxy}-2-[(benzoyloxy)methyl]tetrahydro-2*H*-pyran-3,4-diyl dibenzoate (32)

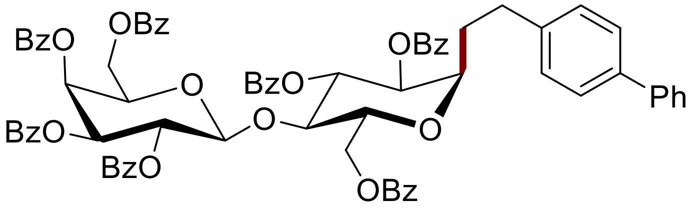

The general procedure **A** was followed using 4-vinyl-1,1'-biphenyl **2aa** (54.0 mg, 0.3 mmol), (2*R*,3*S*,4*S*,5*R*,6*S*)-2-[(benzoyloxy)methyl]-6-{[(2*R*,3*R*,4*S*,5*R*,6*R*)-4,5-bis(benzoyloxy)-2-[(benzoyloxy)methyl]-6-bromotetrahydro-2*H*-pyran-3-yl]oxy}tetrahydro-2*H*-pyran-3,4,5-triyl tribenzoate **1p** (202.4 mg, 0.2 mmol). Purification by column chromatography on silica gel (*n*hexane/EtOAc: 6/1 to 2/1) yielded **32** (122.6 mg, 55%) as a syrup.

^{1}H NMR (400 MHz, $CDCl_3$): δ = 8.10 – 7.99 (m, 10H), 7.96 – 7.89 (m, 2H), 7.83 – 7.74 (m, 2H), 7.70 – 7.64 (m, 1H), 7.59 (ddd, *J* = 7.0, 3.3, 1.9 Hz, 3H), 7.54 – 7.41 (m, 18H), 7.32 (d, *J* = 7.8 Hz, 2H), 7.29 – 7.19 (m, 6H), 6.01 (dd, *J* = 9.6, 8.3 Hz, 1H), 5.92 – 5.73 (m, 2H), 5.55 (dd, *J* = 9.6, 5.9 Hz, 1H), 5.48 (dd, *J* = 10.3, 3.4 Hz, 1H), 4.99 (d, *J* = 7.9 Hz, 1H), 4.54 (d, *J* = 3.6 Hz, 2H), 4.46 (ddd, *J* = 11.6, 5.9, 3.5 Hz, 1H), 4.21 (dd, *J* = 9.5, 8.3 Hz, 1H), 4.07 (dt, *J* = 9.5, 3.7 Hz, 1H), 4.01 (t, *J* = 6.8 Hz, 1H), 3.93 – 3.81 (m, 2H), 2.87 (ddd, *J* = 14.3, 9.7, 4.8 Hz, 1H), 2.70 (ddd, *J* = 14.0, 9.1, 7.1 Hz, 1H), 2.39 (dddd, *J* = 14.3, 11.4, 9.3, 5.0 Hz, 1H), 2.06 – 1.92 (m, 1H).

^{13}C NMR (101 MHz, $CDCl_3$): δ = 165.88 (C_q), 165.56 (C_q), 165.43 (C_q), 165.41 (C_q), 165.22 (C_q), 164.88 (C_q), 140.96 (C_q), 139.88 (C_q), 139.07 (C_q), 133.47 (CH), 133.37 (CH), 133.33 (CH), 133.29 (CH), 133.24 (CH), 133.20 (CH), 129.95 (CH), 129.83 (CH), 129.73 (CH), 129.69 (CH), 129.64 (CH), 129.60 (CH), 129.54 (CH), 129.39 (CH), 129.01 (CH), 128.85 (CH), 128.79 (CH), 128.73 (CH), 128.68 (CH), 128.66 (CH), 128.61 (CH), 128.54 (CH), 128.51 (CH), 128.46 (CH), 128.29 (CH), 128.22 (CH), 127.16 (CH), 127.01 (CH), 101.39 (CH), 72.36 (CH), 71.85 (CH), 71.37 (CH), 71.21 (CH), 70.83 (CH), 69.97 (CH), 69.91 (CH), 67.54 (CH), 62.96 (CH_2), 61.09 (CH_2), 30.81 (CH_2), 27.08 (CH_2).

IR (ATR): ṽ = 1719, 1601, 1584, 1451, 1314, 1266, 1177, 1067, 1025, 905, 704cm^{-1}.
MS (ESI): m/z (relative intensity) 1257 (100) [M+Na].

HR-MS (ESI): *m/z* calcd for $C_{40}H_{48}NaO_{17}^+$ [M+Na]$^+$: 1257.3879, found: 1257.3889.

(2*S*,3*R*,4*S*,5*R*,6*R*)-2-{[(2*R*,3*R*,4*S*,5*S*,6*R*)-6-[2-([1,1'-Biphenyl]-4-yl)ethyl]-4,5-diacetoxy-2-(acetoxymethyl)tetrahydro-2*H*-pyran-3-yl]oxy}-6-(acetoxymethyl)tetrahydro-2*H*-pyran-3,4,5-triyl triacetate (33)

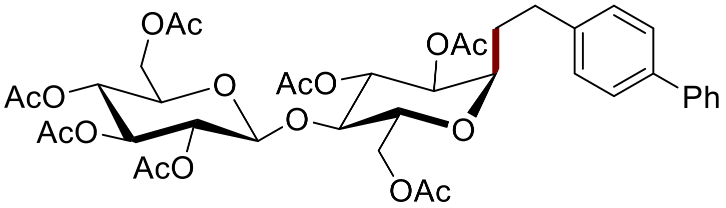

The general procedure **A** was followed using 4-vinyl-1,1'-biphenyl **2aa** (54.0 mg, 0.3 mmol), (2*R*,3*R*,4*S*,5*R*,6*S*)-2-(acetoxymethyl)-6-{[(2*R*,3*R*,4*S*,5*R*,6*R*)-4,5-diacetoxy-2-(acetoxymethyl)-6-bromotetrahydro-2*H*-pyran-3-yl]oxy}tetrahydro-2*H*-pyran-3,4,5-triyl triacetate **1q** (139.6 mg, 0.2 mmol). Purification by column chromatography on silica gel (*n*hexane/EtOAc: 6/1 to 1/1) yielded **33** (134.4 mg, 84%) as a syrup.

^{1}H NMR (400 MHz, $CDCl_3$): δ = 7.58 (d, *J* = 7.2 Hz, 2H), 7.53 (d, *J* = 8.1 Hz, 2H), 7.43 (t, *J* = 7.6 Hz, 2H), 7.33 (t, *J* = 7.3 Hz, 1H), 7.28 (d, *J* = 2.4 Hz, 2H), 5.35 (dd, *J* = 8.9, 7.8 Hz, 1H), 5.21 – 5.07 (m, 2H), 5.06 – 4.99 (m, 1H), 4.94 (dd, *J* = 9.3, 7.9 Hz, 1H), 4.55 (d, *J* = 8.0 Hz, 1H), 4.38 (ddd, *J* = 16.7, 12.1, 3.5 Hz, 2H), 4.21 – 4.10 (m, 2H), 4.06 (dd, *J* = 12.5, 2.4 Hz, 1H), 3.89 – 3.77 (m, 1H), 3.74 – 3.60 (m, 2H), 2.81 (ddd, *J* = 14.4, 9.7, 5.1 Hz, 1H), 2.65 (ddt, *J* = 14.0, 9.0, 6.6 Hz, 1H), 2.14 (s, 3H), 2.13 – 2.11 (m, 1H), 2.08 (s, 3H), 2.03 (s, 6H), 2.01 (s, 3H), 1.99 (s, 3H), 1.84 – 1.78 (m, 1H).

^{13}C NMR (101 MHz, $CDCl_3$): δ = 170.41 (C_q), 170.32 (C_q), 170.11 (C_q), 169.80 (C_q), 169.59 (C_q), 169.22 (C_q), 169.05 (C_q), 140.84 (C_q), 140.00 (C_q), 139.11 (C_q), 128.78 (CH), 128.66 (CH), 127.17 (CH), 127.04 (CH), 126.91 (CH), 100.99 (CH), 72.92 (CH), 71.93 (CH), 71.60 (CH), 71.58 (CH), 70.14 (CH), 69.89 (CH), 69.81 (CH), 67.78 (CH), 62.32 (CH_2), 61.54 (CH_2), 30.72 (CH_2), 27.55 (CH_2), 20.78 (CH_3), 20.67 (CH_3), 20.58 (CH_3), 20.56 (CH_3), 20.47 (CH_3), 20.45 (CH_3).

IR (ATR): ṽ = 1758, 1365, 1205, 1169, 1033, 904, 732, 699, 598 cm^{-1}.
MS (ESI): m/z (relative intensity) 823 (100) [M+Na]$^+$.
HR-MS (ESI): *m/z* calcd for $C_{40}H_{48}NaO_{17}^+$ [M+Na]$^+$: 823.2784, found: 823.2783.

(2*S*,3*R*,4*S*,5*R*,6*R*)-2-{[(2R,3R,4R,5S,6R)-6-[2-([1,1'-Biphenyl]-4-yl)ethyl]-3,4,5-triacetoxytetrahydro-2*H*-pyran-2-yl]methoxy}-6-(acetoxymethyl)tetrahydro-2*H*-pyran-3,4,5-triyl triacetate (34)

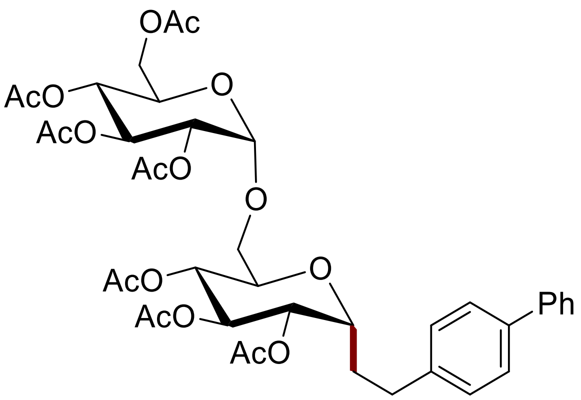

The general procedure **A** was followed using 4-vinyl-1,1'-biphenyl **2aa** (54.0 mg, 0.3 mmol), (2*R*,3*R*,4*S*,5*R*,6*S*)-2-(acetoxymethyl)-6-{[(2*R*,3*R*,4*S*,5*R*,6*R*)-3,4,5-triacetoxy-6-bromotetrahydro-2*H*-pyran-2-yl]methoxy}tetrahydro-2*H*-pyran-3,4,5-triyl triacetate **1r** (139.6 mg, 0.2 mmol). Purification by column chromatography on silica gel (*n*hexane/EtOAc: 6/1 to 1/1) yielded **34** (136.0 mg, 85%) as a syrup.

^{1}H NMR (300 MHz, $CDCl_3$): δ = 7.63 – 7.48 (m, 5H), 7.42 (t, *J* = 7.5 Hz, 2H), 7.37 – 7.27 (m, 2H), 5.52 (dd, *J* = 10.2, 9.3 Hz, 1H), 5.34 (t, *J* = 9.2 Hz, 1H), 5.13 (d, *J* = 3.7 Hz, 1H), 5.11 – 5.01 (m, 2H), 4.96 (t, *J* = 9.2 Hz, 1H), 4.88 (dd, *J* = 10.3, 3.7 Hz, 1H), 4.33 – 4.01 (m, 4H), 3.89 (ddd, *J* = 9.3, 5.9, 3.0 Hz, 1H), 3.73 (dd, *J* = 10.9, 5.9 Hz, 1H), 3.53 (dd, *J* = 10.9, 2.9 Hz, 1H), 2.99 – 2.80 (m, 1H), 2.77 – 2.56 (m, 1H), 2.19 – 2.13 (m, 1H), 2.09 (s, 3H), 2.07 (s, 3H), 2.05 (s, 3H), 2.03 (s, 3H), 2.01 (s, 3H), 1.99 (s, 3H), 1.96 (s, 3H), 1.92 – 1.85 (m, 1H).

^{13}C NMR (75 MHz, $CDCl_3$): δ = 170.57 (C_q), 170.21 (C_q), 170.07 (C_q), 169.76 (C_q), 169.57 (C_q), 169.56 (C_q), 169.47 (C_q), 140.87 (C_q), 139.94 (C_q), 139.07 (C_q), 128.79 (CH), 128.66 (CH), 127.15 (CH), 126.91 (CH), 95.65 (CH), 71.97 (CH), 70.68 (CH), 70.49 (CH), 70.34 (CH), 69.96 (CH), 69.50 (CH), 69.43 (CH), 68.41 (CH), 67.37 (CH), 66.78 (CH_2), 61.81 (CH_2), 30.62 (CH_2), 26.93 (CH_2), 20.67 (CH_2), 20.63 (CH_3), 20.60 (CH_3), 20.51 (CH_3).

IR (ATR): $\tilde{\nu}$ = 1745, 1366, 1217, 1031, 911, 764, 732, 700, 599 cm^{-1}.
MS (ESI): m/z (relative intensity) 823 (100) $[M+Na]^+$.
HR-MS (ESI): *m/z* calcd for $C_{40}H_{48}NaO_{17}^+$ $[M+Na]^+$: 823.2784, found: 823.2782.

(2*R*,3*R*,4*S*,5*R*,6*R*)-2-{[(2*R*,3*R*,4*S*,5*S*,6*R*)-6-[2-([1,1'-biphenyl]-4-yl)ethyl]-4,5-diacetoxy-2-(acetoxymethyl)tetrahydro-2*H*-pyran-3-yl]oxy}-6-(acetoxymethyl)tetrahydro-2*H*-pyran-3,4,5-triyl triacetate (35)

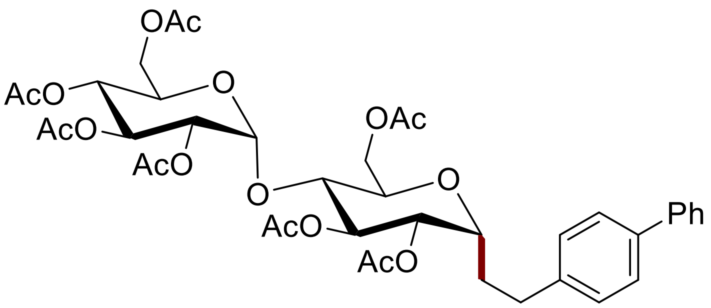

The general procedure **A** was followed using 4-vinyl-1,1'-biphenyl **2aa** (54.0 mg, 0.3 mmol), (2*R*,3*R*,4*S*,5*R*,6*R*)-2-(acetoxymethyl)-6-{[(2*R*,3*R*,4*S*,5*R*,6*R*)-4,5-diacetoxy-2-(acetoxymethyl)-6-bromotetrahydro-2*H*-pyran-3-yl]oxy}tetrahydro-2*H*-pyran-3,4,5-triyl triacetate **1s** (139.6 mg, 0.2 mmol). Purification by column chromatography on silica gel (*n*hexane/EtOAc: 6/1 to 1/1) yielded **35** (121.6 mg, 76%) as a syrup.

^{1}H NMR (400 MHz, $CDCl_3$): δ = 7.63 – 7.52 (m, 4H), 7.44 (dd, *J* = 8.4, 6.9 Hz, 2H), 7.35 (dt, *J* = 8.0, 1.5 Hz, 1H), 7.30 (d, *J* = 8.2 Hz, 2H), 5.40 (dd, *J* = 10.5, 9.5 Hz, 1H), 5.35 (d, *J* = 4.0 Hz, 1H), 5.23 (dd, *J* = 7.8, 6.6 Hz, 1H), 5.06 (t, *J* = 9.5 Hz, 1H), 4.96 (dd, *J* = 7.8, 5.1 Hz, 1H), 4.89 (dd, *J* = 10.5, 4.0 Hz, 1H), 4.35 (dd, *J* = 11.9, 3.3 Hz, 1H), 4.32 – 4.21 (m, 2H), 4.15 – 4.04 (m, 3H), 4.00 (ddd, *J* = 8.9, 5.8, 3.2 Hz, 1H), 3.81 (dd, *J* = 8.0, 6.6 Hz, 1H), 2.84 (ddd, *J* = 14.5, 10.0, 4.9 Hz, 1H), 2.66 (ddd, *J* = 14.5, 9.6, 6.9 Hz, 1H), 2.15 (s, 3H), 2.15 – 2.12 (m, 1H), 2.11 (s, 3H), 2.07 (s, 3H), 2.06 (s, 3H), 2.03 (s, 3H), 2.02 (s, 3H), 2.00 (s, 3H), 1.90 – 1.72 (m, 1H).

^{13}C NMR (101 MHz, $CDCl_3$): δ = 170.56 (C_q), 170.45 (C_q), 170.04 (C_q), 169.98 (C_q), 169.80 (C_q), 169.44 (C_q), 140.92 (C_q), 140.15 (C_q), 139.20 (C_q), 128.89 (CH), 128.74 (CH), 127.29 (CH), 127.12 (CH), 127.00 (CH), 96.37 (CH), 74.01 (CH), 71.94 (CH), 71.05 (CH), 70.20 (CH), 70.01 (CH), 69.92 (CH), 69.49 (CH), 68.45 (CH), 68.12 (CH), 62.99 (CH2), 61.61 (CH_2), 30.95 (CH_2), 28.59 (CH_2), 20.94 (CH_3), 20.87 (CH_3), 20.75 (CH_3), 20.69 (CH_3), 20.61 (CH_3), 20.60 (CH_3), 20.57 (CH_3).

IR (ATR): $\tilde{v}$ = 1757, 1744, 1543, 1368, 1235, 1221, 1032, 732 cm^{-1}.
MS (ESI): m/z (relative intensity) 823 (100) $[M+Na]^+$.
HR-MS (ESI): *m/z* calcd for $C_{40}H_{48}NaO_{17}^+$ $[M+Na]^+$: 823.2784, found: 823.2794.

(2*R*,3*R*,4*S*,5*R*,6*R*)-2-{[(2*R*,3*R*,4*S*,5*R*,6*R*)-6-{[(2*R*,3*R*,4*S*,5*S*,6*R*)-6-[2-([1,1'-biphenyl]-4-yl)ethyl]-4,5-diacetoxy-2-(acetoxymethyl)tetrahydro-2*H*-pyran-3-yl]oxy}-4,5-diacetoxy-2-(acetoxymethyl)tetrahydro-2*H*-pyran-3-yl]oxy}-6-(acetoxymethyl)tetrahydro-2*H*-pyran-3,4,5-triyl triacetate (36)

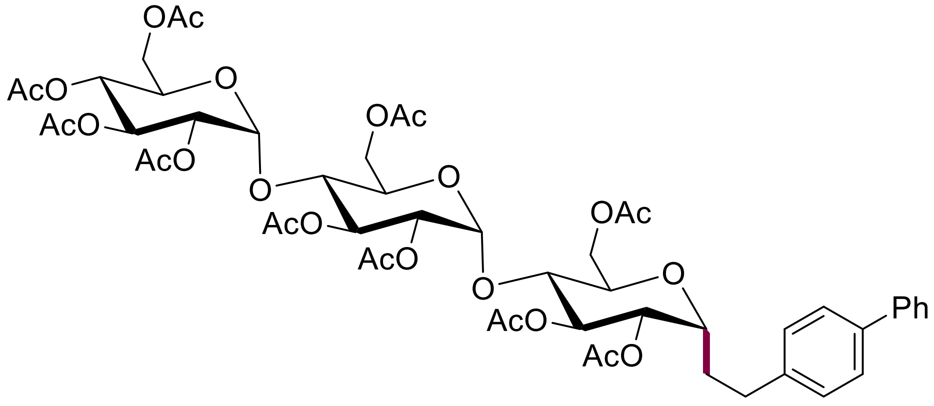

The general procedure **A** was followed using 4-vinyl-1,1'-biphenyl **2aa** (54.0 mg, 0.3 mmol), (2*R*,3*R*,4*S*,5*R*,6*R*)-2-(acetoxymethyl)-6-{[(2*R*,3*R*,4*S*,5*R*,6*R*)-4,5-diacetoxy-2-(acetoxymethyl)-6-{[(2*R*,3*R*,4*S*,5*R*,6*R*)-4,5-diacetoxy-2-(acetoxymethyl)-6-bromotetrahydro-2*H*-pyran-3-yl]oxy}tetrahydro-2*H*-pyran-3-yl]oxy}tetrahydro-2*H*-pyran-3,4,5-triyl triacetate **1t** (197.2 mg, 0.2 mmol). Purification by column chromatography on silica gel (*n*hexane/EtOAc: 10/1 to 3/1) yielded **36** (141.5 mg, 65%) as a syrup.

^{1}H NMR (400 MHz, $CDCl_3$): δ = 7.67 – 7.56 (m, 5H), 7.47 (t, *J* = 7.6 Hz, 2H), 7.39 – 7.33 (m, 2H), 5.48 – 5.42 (m, 2H), 5.39 (d, *J* = 9.4 Hz, 1H), 5.30 – 5.22 (m, 2H), 5.12 (t, *J* = 9.8 Hz, 1H), 5.01 (dd, *J* = 7.5, 5.1 Hz, 1H), 4.91 (dd, *J* = 10.5, 4.1 Hz, 1H), 4.82 (dd, *J* = 10.3, 3.9 Hz, 1H), 4.53 (dd, *J* = 12.3, 2.6 Hz, 1H), 4.38 (d, *J* = 4.6 Hz, 2H), 4.30 (dd, *J* = 12.5, 3.6 Hz, 1H), 4.24 (dd, *J* = 12.4, 3.9 Hz, 1H), 4.18 – 4.05 (m, 4H), 4.05 – 3.92 (m, 2H), 3.81 (dd, *J* = 8.0, 6.1 Hz, 1H), 3.04 – 2.84 (m, 1H), 2.80 – 2.61 (m, 1H), 2.28 – 2.22 (m, 1H), 2.22 (s, 3H), 2.20 (s, 3H), 2.14 (s, 3H), 2.13 (s, 3H), 2.09 (s, 3H), 2.07 (s, 3H), 2.07 (s, 3H), 2.06 (s, 3H), 2.04 (s, 3H), 2.04 (s, 3H), 1.95 – 1.81 (m, 1H).

^{13}C NMR (101 MHz, $CDCl_3$): δ = 170.59 (C_q), 170.52 (C_q), 170.49 (C_q), 170.37 (C_q), 170.09 (C_q), 169.82 (C_q), 169.73 (C_q), 169.70 (C_q), 169.40 (C_q), 140.93 (C_q), 140.19 (C_q), 139.11 (C_q), 128.89 (CH), 128.69 (CH), 127.24 (CH), 126.97 (CH), 96.39 (CH), 95.72 (CH), 74.81 (CH), 72.67 (CH), 71.94 (CH), 71.69 (CH), 70.98 (CH), 70.40 (CH), 70.05 (CH), 70.02 (CH), 69.83 (CH), 69.35 (CH), 68.74 (CH), 68.49 (CH), 67.85 (CH),

63.20 (CH_2), 62.40 (CH_2), 61.34 (CH_2), 30.94 (CH_2), 28.72 (CH_2), 20.89 (CH_3), 20.87 (CH_3), 20.76 (CH_3), 20.75 (CH_3), 20.64 (CH_3), 20.56 (CH_3), 20.49 (CH_3).

IR (ATR): $\tilde{\nu}$ = 1736, 1367, 1204, 1022, 943, 898, 765, 734, 700, 601 cm^{-1} .

MS (ESI): m/z (relative intensity) 1111 (100) $[M+Na]^+$.

HR-MS (ESI): *m/z* calcd for $C_{52}H_{64}NaO_{25}^+$ $[M+Na]^+$: 1111.3629, found: 1111.3631.

(3*R*,4*R*,5*R*,6*R*)-2-(2-acetamido-3-methoxy-3-oxopropyl)-6-[(benzoyloxy)methyl]tetra-hydro-2*H*-pyran-3,4,5-triyl tribenzoate (37)

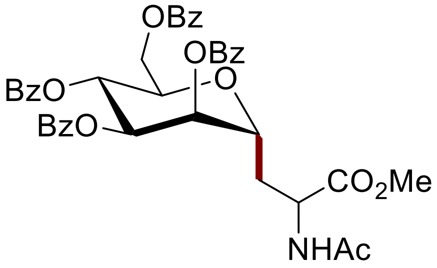

The general procedure **A** was followed using methyl 2-acetamidoacrylate **2au** (42.9 mg, 0.3 mmol), (2*R*,3*R*,4*S*,5*S*)-2-((benzoyloxy)methyl)-6-bromotetrahydro-2*H*-pyran-3,4,5-triyl tri-benzoate **1g** (131.6 mg, 0.2 mmol). Purification by column chromatography on silica gel (DCM/MeOH: 33/1 to 10/1) yielded **37** (71.3 mg, 49%, d.r. = 1:1) as a syrup.

^{1}H NMR (400 MHz, $CDCl_3$): δ = 8.12 (td, *J* = 8.0, 1.3 Hz, 2H), 8.06 – 7.96 (m, 4H), 7.94 – 7.82 (m, 2H), 7.62 – 7.36 (m, 10H), 7.36 – 7.29 (m, 2H), 6.52 (d, *J* = 6.9 Hz, 0.48H), 6.38 (d, *J* = 8.1 Hz, 0.7H), 6.00 (t, *J* = 8.2 Hz, 1H), 5.78 (d, *J* = 3.2 Hz, 0.5H), 5.75 (d, *J* = 3.2 Hz, 0.5H), 5.59 (dt, *J* = 5.1, 3.3 Hz, 1H), 4.86 (tt, *J* = 6.4, 3.4 Hz, 1H), 4.78 – 4.55 (m, 2H), 4.53 – 4.32 (m, 2H), 3.78 (s, 1.5H), 3.74 (s, 1.5H), 2.81 – 2.50 (m, 1H), 2.42 – 2.17 (m, 1H), 2.05 (s, 1.5H), 1.98 (s, 1.5H).

^{13}C NMR (101 MHz, $CDCl_3$): δ = 172.2 (C_q), 172.0, 170.2 (C_q), 170.0 (C_q), 166.6 (C_q), 166.4 (C_q), 165.7 (C_q), 165.6 (C_q), 165.6 (C_q), 165.5 (C_q), 133.7 (CH), 133.7 (CH), 133.6 (CH), 133.6 (CH), 133.6 (CH), 133.6 (CH), 133.3 (CH), 133.3 (CH), 130.0 (CH), 130.0 (CH), 129.9 (CH), 129.9 (CH), 129.9 (CH), 129.8 (CH), 129.4 (C_q), 129.4 (C_q), 129.0 (C_q), 129.0 (C_q), 128.9 (C_q), 128.7 (CH), 128.7 (CH), 128.6 (CH), 128.6 (CH), 128.6 (CH), 72.11 (CH), 71.7 (CH), 71.6 (CH), 71.5 (CH), 71.3 (CH), 69.8 (CH), 69.7 (CH), 67.6 (CH), 67.6 (CH), 62.7 (CH_2), 62.4 (CH_2), 52.9 (CH_3), 52.9 (CH_3), 50.0 (CH), 49.5 (CH), 31.4 (CH_2), 31.0 (CH_2), 23.2 (CH_3).

MS (ESI): m/z (relative intensity) 724 (15) $[M+H]^+$, 746 (100) $[M+Na]^+$.
HR-MS (ESI): *m/z* calcd for $C_{40}H_{38}NO_{12}^+$ $[M+H]^+$: 724.2389, found: 724.2392.

(2*R*,3*R*,4*R*,5*S*,6*R*)-2-[(benzoyloxy)methyl]-6-[3-(benzyloxy)-2-[bis(tert-butoxycarbonyl)amino]-3-oxopropyl]tetrahydro-2*H*-pyran-3,4,5-triyl tribenzoate (38)

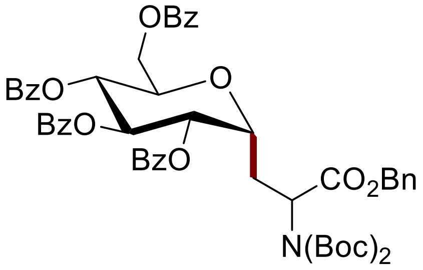

The general procedure **B** was followed using benzyl 2-(bis(tert-butoxycarbonyl)amino)acrylate **2av** (75.4 mg, 0.2 mmol), (2*R*,3*R*,4*S*,5*R*,6*R*)-2-[(benzoyloxy)methyl]-6-bromotetrahydro-2*H*-pyran-3,4,5-triyl tribenzoate **1j** (263.2 mg, 0.4 mmol). Purification by column chromatography on silica gel (*n*hexane/EtOAc: 10/1 to 5/1) yielded **38** (128.3 mg, 67%, d.r. = 1:1) as a syrup.

^{1}H NMR (400 MHz, $CDCl_3$): δ = 8.27 – 7.80 (m, 9H), 7.57 – 7.23 (m, 16H), 6.02 (t, *J* = 9.2 Hz, 0.5H), 5.96 (t, *J* = 8.8 Hz, 0.5H), 5.71 – 5.54 (m, 2H), 5.34 – 5.23 (m, 1H), 5.20 – 4.82 (m, 2H), 4.96 – 4.27 (m, 4H), 3.02 (ddd, *J* = 15.9, 12.8, 3.2 Hz, 0.5H), 2.70 (ddd, *J* = 15.0, 7.9, 3.1 Hz, 0.5H), 2.46 (ddd, *J* = 14.9, 11.8, 5.3 Hz, 0.5H), 2.28 (ddd, *J* = 15.8, 10.2, 2.7 Hz, 0.5H), 1.40 (s, 10H), 1.30 (s, 8H).

^{13}C NMR (101 MHz, $CDCl_3$): δ = 170.79 (C_q), 170.01 (C_q), 166.23 (C_q), 166.09 (C_q), 165.75 (C_q), 165.71 (C_q), 165.26 (C_q), 165.22 (C_q), 165.20 (C_q), 165.13 (C_q), 152.17 (C_q), 152.01 (C_q), 135.42 (C_q), 133.49 (CH), 133.41 (CH), 133.32 (CH), 133.29 (CH), 133.24 (CH), 132.97 (CH), 129.91 (CH), 129.83 (CH), 129.82 (CH), 129.78 (CH), 129.75 (CH), 129.07 (C_q), 129.03 (C_q), 129.01 (C_q), 128.88 (C_q), 128.83 (C_q), 128.49 (CH), 128.47 (CH), 128.39 (CH), 128.35 (CH), 128.33 (CH), 128.32 (CH), 128.30 (CH), 128.23 (CH), 128.17 (CH), 128.06 (CH), 128.03 (CH), 83.54 (C_q), 83.41 (C_q), 71.03 (CH), 70.81 (CH), 70.68 (CH), 70.60 (CH), 70.40 (CH), 69.95 (CH), 69.85 (CH), 69.66 (CH), 69.43 (CH), 69.25 (CH), 67.20 (CH_2), 67.00 (CH_2), 63.36 (CH_2), 62.67 (CH_2), 54.88 (CH), 54.40 (CH), 27.91 (CH_3), 27.77 (CH_3), 27.63 (CH_2), 26.01 (CH_2).

IR (ATR): $\tilde{\nu}$ = 1721, 1600, 1453, 1369, 1266, 1121, 1091, 1069, 1027, 711 cm^{-1}.
MS (ESI): m/z (relative intensity) 980 (100) $[M+Na]^+$.
HR-MS (ESI): *m/z* calcd for $C_{54}H_{55}NNaO_{15}^+$ $[M+Na]^+$: 980.3464, found: 980.3463.

(2*R*,3*S*,4*R*,5*S*,6*R*)-2-[(benzoyloxy)methyl]-6-[3-(benzyloxy)-2-[bis(tert-butoxycarbonyl)amino]-3-oxopropyl]tetrahydro-2*H*-pyran-3,4,5-triyl tribenzoate (39)

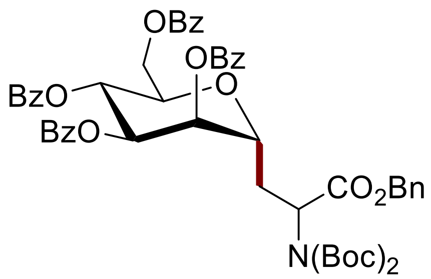

The general procedure **B** was followed using benzyl 2-(bis(tert-butoxycarbonyl)amino)acrylate **2av** (75.4 mg, 0.2 mmol), (2*R*,3*R*,4*S*,5*S*,6*R*)-2-[(benzoyloxy)methyl]-6-bromotetrahydro-2*H*-pyran-3,4,5-triyl tribenzoate **1g** (263.2 mg, 0.4 mmol). Purification by column chromatography on silica gel (*n*hexane/EtOAc: 10/1 to 5/1) yielded **39** (143.6 mg, 75%, d.r. = 1.3:1) as a syrup.

^{1}H NMR (400 MHz, $CDCl_3$): δ = 8.20 – 8.03 (m, 4H), 8.01 – 7.93 (m, 2H), 7.85 (ddd, *J* = 8.7, 5.8, 1.4 Hz, 2H), 7.63 – 7.42 (m, 5H), 7.41 – 7.28 (m, 12H), 6.05 (td, *J* = 9.0, 4.5 Hz, 1H), 5.83 (ddd, *J* = 9.3, 6.2, 3.2 Hz, 1H), 5.69 (t, *J* = 3.0 Hz, 1H), 5.32 (dd, *J* = 8.5, 4.2 Hz, 1H), 5.24 – 4.96 (m, 2H), 4.77 – 4.57 (m, 2H), 4.53 – 4.25 (m, 2H), 3.09 (dd, *J* = 12.4, 3.5 Hz, 0.5H), 2.77 (dd, *J* = 7.9, 3.4 Hz, 0.5H), 2.46 (dd, *J* = 11.5, 5.1 Hz, 0.5H), 2.29 (dd, *J* = 9.2, 2.9 Hz, 0.5H), 1.44 (s, 18H).

^{13}C NMR (75 MHz, $CDCl_3$): δ = 170.59 (C_q), 169.97 (C_q), 166.20 (C_q), 166.08 (C_q), 165.64 (C_q), 165.51 (C_q), 165.49 (C_q), 165.39 (C_q), 165.36 (C_q), 165.27 (C_q), 152.19 (C_q), 152.17 (C_q), 135.44 (C_q), 135.42 (C_q), 133.42 (CH), 133.37 (CH), 133.35 (CH), 133.25 (CH), 132.89 (CH), 129.84 (CH), 129.77 (CH), 129.73 (CH), 129.64 (CH), 129.10 (C_q), 129.02 (C_q), 128.49 (CH), 128.47 (CH), 128.43 (CH), 128.35 (CH), 128.23 (CH), 128.18 (CH), 128.12 (CH), 128.06 (CH), 83.59 (C_q), 83.56 (C_q), 73.52 (CH), 72.64 (CH), 71.66 (CH), 71.59 (CH), 70.67 (CH), 70.44 (CH), 70.16 (CH), 70.04 (CH), 67.59 (CH), 67.49 (CH), 67.24 (CH_2), 67.08 (CH_2), 63.21 (CH_2), 62.71 (CH_2), 54.93 (CH), 54.82 (CH), 30.10 (CH_2), 29.32 (CH_2), 27.94 (CH_3), 27.91(CH_3).

IR (ATR): $\tilde{\nu}$ = 1722, 1602, 1452, 1368, 1264, 1108, 1094 1069, 1027, 710 cm^{-1} .
MS (ESI): m/z (relative intensity) 980 (100) $[M+Na]^+$.
HR-MS (ESI): *m/z* calcd for $C_{54}H_{55}NNaO_{15}^+$ $[M+Na]^+$: 980.3464, found: 980.3472.

(2R,3R,4S,5R,6R)-2-[3-(Benzyloxy)-2-[bis(tert-butoxycarbonyl)amino]-3-oxopropyl]-6-methyltetrahydro-2H-pyran-3,4,5-triyl tribenzoate (40)

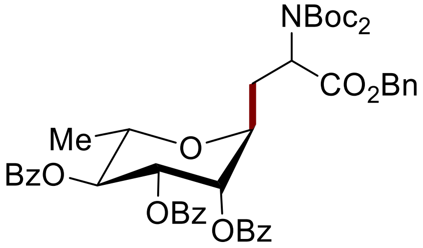

The general procedure **B** was followed using benzyl 2-(bis(tert-butoxycarbonyl)amino)acrylate **2av** (75.4 mg, 0.2 mmol), (2R,3S,4S,5R,6R)-2-bromo-6-methyltetrahydro-2H-pyran-3,4,5-triyl tribenzoate **1k** (215.2 mg, 0.4 mmol). Purification by column chromatography on silica gel ($n$hexane/EtOAc: 10/1 to 5/1) yielded **40** (83.7 mg, 50%, d.r. = 1.5:1) as a syrup.

^{1}H NMR (400 MHz, $CDCl_3$): δ = 8.12 – 7.94 (m, 4H), 7.84 (ddd, J = 8.6, 7.2, 1.4 Hz, 2H), 7.64 – 7.50 (m, 2H), 7.48 – 7.39 (m, 4H), 7.36 – 7.28 (m, 8H), 5.76 (dd, J = 8.5, 3.4 Hz, 0.4H), 5.71 (dd, J = 9.1, 3.4 Hz, 0.6H), 5.67 – 5.53 (m, 2H), 5.33 – 5.22 (m, 1H), 5.22 – 5.11 (m, 2H), 4.53 (dt, J = 11.5, 3.1 Hz, 0.6H), 4.20 (dt, J = 12.2, 3.1 Hz, 0.4H), 4.16 – 3.98 (m, 1H), 3.10 – 1.93 (m, 2H), 1.47 (s, 10H), 1.44 (s, 8H), 1.39 (dd, J = 6.3, 3.6 Hz, 3H).

^{13}C NMR (101 MHz, $CDCl_3$): δ = 170.67 (C_q), 170.40 (C_q), 170.27 (C_q), 170.12 (C_q), 165.62 (C_q), 165.60 (C_q), 165.58 (C_q), 165.51 (C_q), 165.44 (C_q), 152.17 (C_q), 152.09 (C_q), 152.01 (C_q), 151.98 (C_q), 135.64 (C_q), 135.48 (C_q), 135.44 (C_q), 133.32 (CH), 133.27 (CH), 133.24 (CH), 133.21 (CH), 133.17 (CH), 133.14 (CH), 129.84 (CH), 129.81 (CH), 129.78 (CH), 129.71 (CH), 129.68 (CH), 129.66 (CH), 129.58 (C_q), 129.33 (C_q), 129.28 (C_q), 129.17 (C_q), 129.14 (C_q), 128.45 (CH), 128.44 (CH), 128.42 (CH), 128.33 (CH), 128.29 (CH), 128.18 (CH), 128.16 (CH), 128.10 (CH), 128.06 (CH), 127.97 (CH), 127.95 (CH), 83.38 (C_q), 83.34 (C_q), 83.11 (C_q), 83.09 (C_q), 73.43 (CH), 72.29 (CH), 72.01 (CH), 71.74 (CH), 70.65 (CH), 70.00 (CH), 69.91 (CH), 68.85 (CH), 68.54 (CH), 67.13 (CH_2), 67.04 (CH_2), 66.72 (CH_2), 66.70 (CH_2), 58.24 (CH), 57.53 (CH), 55.34 (CH), 54.75 (CH), 27.95 (CH_2), 27.89 (CH_2), 17.65 (CH_3), 17.47 (CH_3).

IR (ATR): $\tilde{v}$ = 1729, 1453, 1368, 1315, 1264, 1177, 1107, 1069, 1027, 711 cm^{-1} .
MS (ESI): m/z (relative intensity) 860 (100) $[M+Na]^+$.
HR-MS (ESI): m/z calcd for $C_{47}H_{51}NNaO_{13}^+$ $[M+Na]^+$: 860.3253, found: 860.3263.

(2*R*,3*S*,4*S*,5*R*,6*S*)-2-[(benzoyloxy)methyl]-6-{[(2*R*,3*R*,4*S*,5*S*,6*R*)-4,5-bis(benzoyloxy)-2-[(benzoyloxy)methyl]-6-[3-(benzyloxy)-2-[bis(tert-butoxycarbonyl)amino]-3-oxopropyl]tetrahydro-2*H*-pyran-3-yl]oxy}tetrahydro-2*H*-pyran-3,4,5-triyl tribenzoate (41)

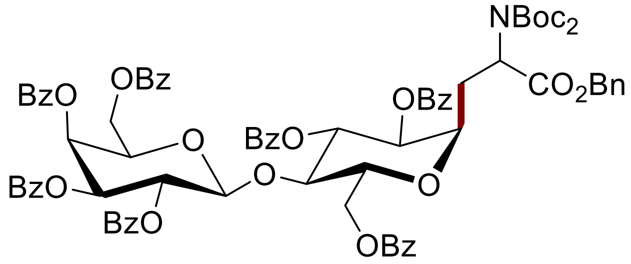

The general procedure **B** was followed using benzyl 2-(bis(tert-butoxycarbonyl)amino)acrylate **2av** (75.4 mg, 0.2 mmol), (2*R*,3*S*,4*S*,5*R*,6*S*)-2-[(benzoyloxy)methyl]-6-{[(2*R*,3*R*,4*S*,5*R*,6*R*)-4,5-bis(benzoyloxy)-2-[(benzoyloxy)methyl]-6-bromotetrahydro-2*H*-pyran-3-yl]oxy}tetrahydro-2*H*-pyran-3,4,5-triyl tribenzoate **1p** (452.8 mg, 0.4 mmol). Purification by column chromatography on silica gel (*n*hexane/EtOAc: 10/1 to 5/1) yielded **41** (154.5 mg, 54%, d.r. = 1.5:1) as a syrup.

^{1}H NMR (400 MHz, $CDCl_3$): δ = 8.11 – 7.94 (m, 10H), 7.86 (ddd, *J* = 8.4, 3.2, 1.3 Hz, 2H), 7.73 (ddd, *J* = 8.1, 6.4, 1.4 Hz, 2H), 7.61 (td, *J* = 8.8, 8.4, 4.4 Hz, 2H), 7.57 – 7.51 (m, 2H), 7.50 – 7.43 (m, 6H), 7.41 – 7.28 (m, 11H), 7.23 – 7.15 (m, 5H), 5.97 (dd, *J* = 9.7, 8.4 Hz, 0.4H), 5.90 (dd, *J* = 9.7, 8.4 Hz, 0.6H), 5.79 – 5.65 (m, 2H), 5.59 (dd, *J* = 8.5, 4.9 Hz, 0.5H), 5.55 (dd, *J* = 8.5, 4.9 Hz, 0.5H), 5.37 (dd, *J* = 10.2, 3.5 Hz, 0.4H), 5.33 (dd, *J* = 10.2, 3.5 Hz, 0.6H), 5.23 – 5.00 (m, 3H), 4.92 (d, *J* = 8.0 Hz, 0.4H), 4.88 (d, *J* = 8.0 Hz, 0.6H), 4.83 – 4.36 (m, 4H), 4.31 – 4.14 (m, 1H), 4.09 – 3.88 (m, 1H), 3.88 – 3.66 (m, 2H), 2.95 – 1.83 (m, 2H), 1.29 (s, 10H), 1.26 (s, 8H).

^{13}C NMR (101 MHz, $CDCl_3$): δ = 170.53 (C_q), 169.91 (C_q), 165.91 (C_q), 165.70 (C_q), 165.54 (C_q), 165.52 (C_q), 165.46 (C_q), 165.40 (C_q), 165.37 (C_q), 165.33 (C_q), 165.28 (C_q), 165.23 (C_q), 165.19 (C_q), 164.79 (C_q), 164.71 (C_q), 151.90 (C_q), 151.84 (C_q), 135.36 (C_q), 135.34 (C_q), 133.42 (CH), 133.36 (CH), 133.30 (CH), 133.26 (CH), 133.17 (CH), 133.13 (CH), 133.10 (CH), 129.93 (CH), 129.87 (CH), 129.81 (CH), 129.72 (CH), 129.70 (CH), 129.68 (CH), 129.60 (CH), 129.58 (CH), 129.50 (CH), 129.42 (C_q), 129.05 (C_q), 128.85 (C_q), 128.82 (C_q), 128.70 (C_q), 128.67 (C_q), 128.61 (C_q), 128.54 (CH), 128.51 (CH), 128.46 (CH), 128.41 (CH), 128.40 (CH), 128.37 (CH), 128.23 (CH), 128.19 (CH), 128.14 (CH), 128.06 (CH), 128.01 (CH), 127.98 (CH), 101.18 (CH),

101.06 (CH), 83.29 (C_q), 83.20 (C_q), 76.47 (CH), 76.08 (CH), 72.05 (CH), 71.95 (CH), 71.69 (CH), 71.34 (CH), 71.30 (CH), 70.71 (CH), 70.67 (CH), 70.65 (CH), 69.91 (CH), 69.83 (CH), 69.76 (CH), 67.52 (CH), 67.50 (CH), 67.04 (CH_2), 66.88 (CH_2), 62.81 (CH_2), 62.44 (CH_2), 61.14 (CH_2), 61.08 (CH_2), 54.73 (CH), 54.28 (CH), 27.73 (CH_3), 27.63 (CH_3).

IR (ATR): $\tilde{v}$ = 1720, 1602, 1452, 1265, 1090, 1067, 1026, 907, 731, 705 cm^{-1} .
MS (ESI): m/z (relative intensity) 1454 (100) $[M+Na]^+$.
HR-MS (ESI): *m/z* calcd for $C_{81}H_{77}NNaO_{23}^+$ $[M+Na]^+$: 1454.4779, found: 1454.1766.

(2*R*,3*S*,4*R*,5*S*,6*R*)-2-(Acetoxymethyl)-6-[4-(methoxycarbonyl)phenyl]tetrahydro-2*H*-pyran-3,4,5-triyl triacetate (42a)

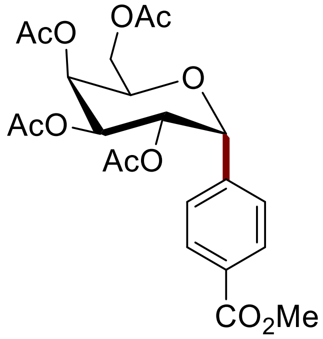

The general procedure **C** was followed using (2*R*,3*S*,4*S*,5*R*,6*R*)-2-(acetoxymethyl)-6-bromotetrahydro-2*H*-pyran-3,4,5-triyl triacetate **1a** (82.0 mg, 0.2 mmol) and methyl 4-iodobenzoate **2ba** (78.3 mg, 0.3 mmol), $Ni(acac)_2$ (10 mol %), Neocuproin **L6** (10 mol %), $LiClO_4$ (63.6 mg, 06 mmol), DIPEA (0.8 mmol), Purification by column chromatography on silica gel (*n*hexane/EtOAc: 3/1) yielded **42a** (67 mg, 72%) as a syrup.

^{1}H NMR (400 MHz, $CDCl_3$) δ 8.04 (d, *J* = 8.4 Hz, 2H), 7.77 – 7.43 (m, 2H), 5.52 (dd, *J* = 4.9, 3.1 Hz, 1H), 5.41 (qd, *J* = 6.3, 3.1 Hz, 2H), 5.34 (d, *J* = 3.1 Hz, 1H), 4.67 (dd, *J* = 12.1, 8.6 Hz, 1H), 4.28 (dt, *J* = 8.7, 4.4 Hz, 1H), 4.18 (dd, *J* = 12.1, 3.9 Hz, 1H), 3.94 (s, 3H), 2.18 (s, 3H), 2.15 (s, 3H), 2.05 (s, 3H), 1.93 (s, 3H).

^{13}C NMR (101 MHz, $CDCl_3$): δ = 170.67 (C_q), 169.67 (C_q), 169.28 (C_q), 169.11 (C_q), 166.61 (C_q), 141.11 (C_q), 129.80 (C_q), 129.48 (CH), 127.03 (CH), 71.27 (CH), 70.59 (CH), 69.88 (CH), 67.62 (CH), 66.36 (CH), 59.94 (CH_2), 52.10 (CH_3), 20.81 (CH_3), 20.71 (CH_3), 20.66 (CH_3), 20.46 (CH_3).

IR (ATR): ṽ = 1745, 1719, 1369, 1278, 1205, 1106, 914, 728, 602 cm^{-1} .
MS (ESI): m/z (relative intensity) 489 (100) $[M+Na]^+$, 955 (50) [2M+Na].
HR-MS (ESI): *m/z* calcd for $C_{22}H_{26}NaO_{11}^+$ $[M+Na]^+$: 489.1367, found: 489.1378

(2*R*,3*S*,4*R*,5*S*,6*R*)-2-(Acetoxymethyl)-6-[4-(methoxycarbonyl)phenyl]tetrahydro-2*H*-pyran-3,4,5-triyl triacetate (42ba)

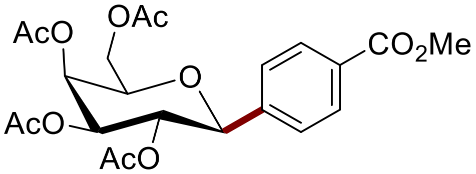

The general procedure **D** was followed using (2*R*,3*S*,4*S*,5*R*,6*R*)-2-(acetoxymethyl)-6-bromotetrahydro-2*H*-pyran-3,4,5-triyl triacetate **1a** (82.0 mg, 0.2 mmol) and methyl 4-iodobenzoate **2ba** (78.3 mg, 0.3 mmol), $Ni(acac)_2$ (10 mol %), 4,4',4''-tri-*tert*-butyl-2,2':6',2''-terpyridine **L12** (10 mol %), $LiClO_4$ (63.6 mg, 06 mmol), DIPEA (0.8 mmol), Purification by column chromatography on silica gel (*n*hexane/EtOAc: 3/1) yielded **42ba** (55.9 mg, 60%) as a syrup.

^{1}H NMR (400 MHz, $CDCl_3$): δ = 8.02 (d, *J* = 8.4 Hz, 2H), 7.45 (d, *J* = 8.3 Hz, 2H), 5.53 (dd, *J* = 3.4, 1.2 Hz, 1H), 5.30 (t, *J* = 9.9 Hz, 1H), 5.19 (dd, *J* = 10.2, 3.4 Hz, 1H), 4.42 (**d, *J* = 9.6 Hz, 1H**), 4.18 (dd, *J* = 6.6, 2.8 Hz, 2H), 4.10 – 4.04 (m, 1H), 3.91 (s, 3H), 2.21 (s, 3H), 2.03 (s, 3H), 1.98 (s, 3H), 1.81 (s, 3H).

^{13}C NMR (101 MHz, $CDCl_3$): δ = 170.44 (C_q), 170.25 (C_q), 170.16 (C_q), 168.82 (C_q), 166.62 (C_q), 141.51 (C_q), 130.56 (C_q), 129.63 (CH), 127.22 (CH), 80.25 (CH), 74.73 (CH), 72.00 (CH), 69.84 (CH), 67.73 (CH), 61.70 (CH_2), 52.16 (CH_3), 20.74 (CH_3), 20.70 (CH_3), 20.62 (CH_3), 20.45 (CH_3).

IR (ATR): $\tilde{v}$ = 1742, 1725, 1326, 1278, 1204, 1106, 912, 713, 617 cm^{-1} .
MS (ESI): m/z (relative intensity) 489 (100) $[M+Na]^+$, 955 (50) [2M+Na].
HR-MS (ESI): *m/z* calcd for $C_{22}H_{26}NaO_{11}^+$ $[M+Na]^+$: 489.1367, found: 489.1380.

(2*R*,3*S*,4*R*,5*S*,6*R*)-2-[(1,1'-biphenyl)-4-yl]-6-(acetoxymethyl)tetrahydro-2*H*-pyran-3,4,5-triyl triacetate (42bb)

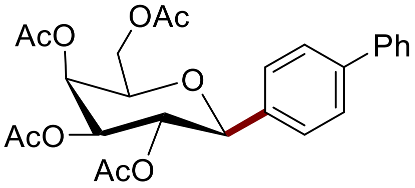

The general procedure **D** was followed using (2*R*,3*S*,4*S*,5*R*,6*R*)-2-(acetoxymethyl)-6-bromotetrahydro-2*H*-pyran-3,4,5-triyl triacetate **1a** (82.0 mg, 0.2 mmol) and **2aa** (54.0 mg, 0.3 mmol), $Ni(acac)_2$ (10 mol %), 4,4',4''-tri-*tert*-butyl-2,2':6',2''-terpyridine **L12** (10 mol %), $LiClO_4$ (63.6 mg, 06 mmol), DIPEA (0.8 mmol), Purification by column chromatography on silica gel (*n*hexane/EtOAc: 3/1) yielded **42bb** (30 mg, 32%) as a syrup.

^{1}H NMR (400 MHz, $CDCl_3$): δ 7.66 – 7.59 (m, 4H), 7.53 – 7.46 (m, 4H), 7.43 – 7.36 (m, 1H), 5.59 (dd, *J* = 3.4, 1.2 Hz, 1H), 5.46 (t, *J* = 9.9 Hz, 1H), 5.26 (d, *J* = 3.4 Hz, 1H), 4.26 – 4.21 (m, 2H), 4.14 (m, 1H), 2.27 (s, 3H), 2.08 (s, 3H), 2.04 (s, 3H), 1.88 (s, 3H).
^{13}C NMR (101 MHz, $CDCl_3$): *δ* = 170.53 (C_q), 170.37 (C_q), 170.30 (C_q), 169.03 (C_q), 141.73 (C_q), 140.55 (C_q), 135.51 (C_q), 128.83 (CH), 128.80 (CH), 127.84 (CH), 127.51 (C_q), 127.14 (CH), 80.64 (CH), 74.73 (CH), 69.82 (CH) (CH), 69.86 (CH), 67.91(CH), 61.83 (CH_2), 20.82 (CH_3), 20.78 (CH_3), 20.71 (CH_3), 20.59 (CH_3).

IR (ATR): ṽ = 1745, 1662, 1560, 1547, 1369, 1223, 913, 731, 720 cm^{-1} .
MS (ESI): m/z (relative intensity) 507 (100) $[M+Na]^+$, 991 (50) [2M+Na].
HR-MS (ESI): *m/z* calcd for $C_{26}H_{28}NaO_9^+$ $[M+Na]^+$: 507.1626, found: 507.1649.

(2*R*,3*S*,4*R*,5*S*,6*R*)-2-(acetoxymethyl)-6-(4-cyanophenyl)tetrahydro-2*H*-pyran-3,4,5-triyl triacetate (42bc)

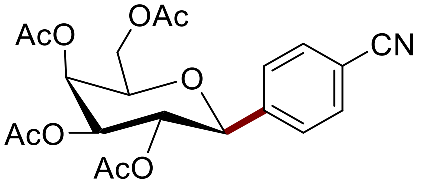

The general procedure **D** was followed using (2*R*,3*S*,4*S*,5*R*,6*R*)-2-(acetoxymethyl)-6-bromotetrahydro-2*H*-pyran-3,4,5-triyl triacetate **1a** (82.0 mg, 0.2 mmol) and 4-iodobenzonitrile **2be** (68.7 mg, 0.3 mmol), Ni(acac)$_2$ (10 mol %), Neocuproin **L6** (10 mol %), $LiClO_4$ (63.6 mg, 06 mmol), DIPEA (0.8 mmol), Purification by column chromatography on silica gel (*n*hexane/EtOAc: 3/1) yielded **42bc** (55.8 mg, 65%) as a syrup.

^{1}H NMR (400 MHz, $CDCl_3$): δ 7.70 – 7.67 (m, 2H), 7.56 – 7.52 (m, 2H), 5.56 (dd, *J* = 3.3, 1.2 Hz, 1H), 5.34 – 5.26 (m, 1H), 5.22 (dd, *J* = 10.1, 3.3 Hz, 1H), 4.46 (d, *J* = 9.5 Hz, 1H), 4.23 – 4.17 (m, 2H), 4.12 (ddd, *J* = 7.0, 5.9, 1.2 Hz, 1H), 2.24 (s, 3H), 2.06 (s, 3H), 2.01 (s, 3H), 1.88 (s, 3H).

^{13}C NMR (101 MHz, $CDCl_3$): *δ* = 170.45 (C_q), 170.19 (C_q), 170.13 (C_q), 168.82 (C_q), 141.84 (C_q), 132.19 (CH), 127.59 (CH), 118.43 (C_q), 112.80 (C_q), 79.86 (CH), 74.83 (CH), 71.87 (C_q), 69.75 (CH), 67.68 (CH), 61.69 (CH_2), 20.74 (CH_3), 20.72 (CH_3), 20.63 (CH_3), 20.48 (CH_3).

IR (ATR): ṽ = 1751, 1745, 1362, 1237, 1221, 976, 946, 716, 604 cm^{-1} .
MS (ESI): m/z (relative intensity) 456 (100) $[M+Na]^+$, 889 (50) [2M+Na].
HR-MS (ESI): *m/z* calcd for $C_{21}H_{23}NNaO_9^+$ $[M+Na]^+$: 456.1265, found: 456.1276.

(2*R*,3*S*,4*R*,5*S*,6*R*)-2-(Acetoxymethyl)-6-(4-acetylphenyl)tetrahydro-2*H*-pyran-3,4,5-triyl triacetate (42bd)

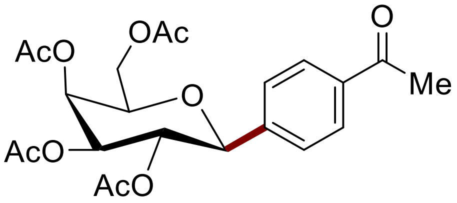

The general procedure **D** was followed using (2*R*,3*S*,4*S*,5*R*,6*R*)-2-(acetoxymethyl)-6-bromotetrahydro-2*H*-pyran-3,4,5-triyl triacetate **1a** (82.0 mg, 0.2 mmol) and 1-(4-iodophenyl)ethan-1-one **2bf** (73.8 mg, 0.3 mmol), Ni(acac)$_2$ (10 mol %), Neocuproin **L6** (10 mol %), $LiClO_4$ (63.6 mg, 06 mmol), DIPEA (0.8 mmol), Purification by column chromatography on silica gel (*n*hexane/EtOAc: 3/1) yielded **42bd** (47 mg, 52%) as a syrup.

^{1}H NMR (400 MHz, $CDCl_3$): δ 7.97 (d, *J* = 8.5 Hz, 2H), 7.54 – 7.49 (m, 2H), 5.57 (dd, *J* = 3.4, 1.2 Hz, 1H), 5.35 (t, *J* = 9.9 Hz, 1H), 5.23 (dd, *J* = 10.2, 3.4 Hz, 1H), 4.47 (d, *J* = 9.7 Hz, 1H), 4.22 (dd, *J* = 6.6, 1.7 Hz, 2H), 4.12 (ddd, *J* = 7.0, 6.1, 1.3 Hz, 1H), 2.62 (s, *3*H), 2.25 (s, 3H), 2.07 (s, 3H), 2.02 (s, 3H), 1.86 (s, 3H).

^{13}C NMR (101 MHz, $CDCl_3$): δ = 197.62 (C_q), 170.48 (C_q), 170.28 (C_q), 170.19 (C_q), 168.91 (C_q), 141.73 (C_q), 137.38 (C_q), 128.40 (CH), 127.49 (CH), 80.20 (CH), 74.76 (CH), 72.04 (CH), 69.84(CH), 67.77 (CH), 61.73 (CH_2), 26.68 (CH_3), 20.77 (CH_3), 20.73 (CH_3), 20.65 (CH_3), 20.52 (CH_3).

IR (ATR): ṽ = 1748, 1720, 1570, 1334, 1228, 927, 744, 639 cm^{-1} .
MS (ESI): m/z (relative intensity) 473 (100) $[M+Na]^+$, 923 (50) [2M+Na].
HR-MS (ESI): *m/z* calcd for $C_{22}H_{26}NaO_{10}^+$ $[M+Na]^+$: 473.1418, found: 473.1437.

(2*R*,3*R*,4*S*,5*R*,6*S*)-2-(Acetoxymethyl)-6-{[(2*R*,3*R*,4*S*,5*S*,6*R*)-4,5-diacetoxy-2-(acetoxymethyl)-6-[4-(methoxycarbonyl)phenyl]tetrahydro-2*H*-pyran-3-yl]oxy}tetrahydro-2*H*-pyran-3,4,5-triyl triacetate (42be)

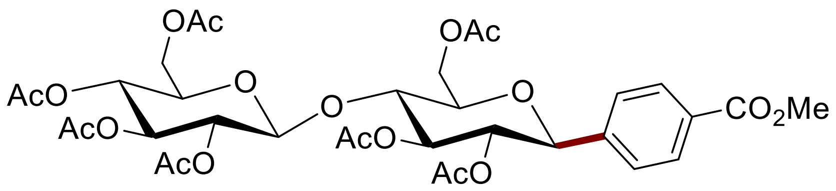

The general procedure **D** was followed using (2*R*,3*R*,4*S*,5*R*,6*S*)-2-(acetoxymethyl)-6-{[(2*R*,3*R*,4*S*,5*R*,6*R*)-4,5-diacetoxy-2-(acetoxymethyl)-6-bromotetrahydro-2*H*-pyran-3-yl]oxy}tetrahydro-2*H*-pyran-3,4,5-triyl triacetate **1q** (139.6 mg, 0.2 mmol), methyl 4-iodobenzoate **2ba** (78.3 mg, 0.3 mmol), $Ni(acac)_2$ (10 mol %), Neocuproin **L6** (10 mol %), $LiClO_4$ (63.6 mg, 06 mmol), DIPEA (0.8 mmol), Purification by column chromatography on silica gel (*n*hexane/EtOAc: 10/1 to 5/1) yielded **42be** (72.1 mg, 48%) as a syrup.

^{1}H NMR (400 MHz, $CDCl_3$): δ 8.14 – 7.98 (m, 2H), 7.41 (d, *J* = 8.4 Hz, 2H), 5.35 (t, *J* = 9.3 Hz, 1H), 5.20 (t, *J* = 9.3 Hz, 1H), 5.12 (t, *J* = 9.6 Hz, 1H), 5.06 – 4.96 (m, 2H), 4.65 – 4.53 (m, 2H), 4.47 – 4.38 (m, 2H), 4.23 – 4.13 (m, 2H), 4.12 – 4.05 (m, 1H), 3.94 (s, 3H), 3.79 (ddd, *J* = 9.9, 5.0, 2.0 Hz, 1H), 3.75 (ddd, *J* = 9.9, 5.0, 2.0 Hz, 1H), 2.16 (s, 3H), 2.12 (s, 3H), 2.08 (s, 3H), 2.05 (s, 3H), 2.04 (s, 3H), 2.02 (s, 3H), 1.85 (s, 3H).

^{13}C NMR (101 MHz, $CDCl_3$): δ = 170.51 (C_q), 170.36 (C_q), 170.24 (C_q), 169.90 (C_q), 169.32 (C_q), 169.10 (C_q), 169.06 (C_q), 166.62 (C_q), 141.40 (C_q), 130.54 (C_q), 129.68 (CH), 126.92 (CH), 100.92 (CH), 79.39 (CH), 77.14 (CH), 73.78 (CH), 72.95 (CH), 72.83 (CH), 72.75 (CH), 72.03 (CH), 71.65 (CH), 67.79 (CH), 62.19(CH), 61.57 (CH), 52.20 (CH_2), 20.90 (CH_3), 20.86 (CH_3), 20.69 (CH_3), 20.58 (CH_3), 20.41 (3 CH_3).

IR (ATR): ṽ = 1740, 1721, 1356, 1278, 1200, 1024, 908, 729, 605 cm^{-1}.

MS (ESI): m/z (relative intensity) 777 (100) $[M+Na]^+$.

HR-MS (ESI): *m/z* calcd for $C_{34}H_{42}NaO_{19}^+$ $[M+Na]^+$: 777.2213, found: 777.2235.

(2*R*,3*S*,4*S*,5*R*,6*S*)-2-(Acetoxymethyl)-6-benzoyltetrahydro-2*H*-pyran-3,4,5-triyl triacetate (43a)

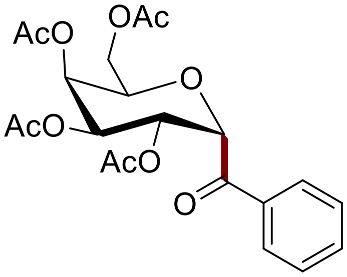

The general procedure **C** was followed using (2*R*,3*S*,4*S*,5*R*,6*R*)-2-(acetoxymethyl)-6-bromotetrahydro-2*H*-pyran-3,4,5-triyl triacetate **1a** (82.0 mg, 0.2 mmol) and benzoyl chloride **2bi** (42.0 mg, 0.3 mmol), $NiBr_2{\cdot}DME$ (10 mol %), 6,6'-dimethyl-2,2'-bipyridine **L2** (10 mol %), $LiClO_4$ (63.6 mg, 06 mmol), DIPEA (0.8 mmol), Purification by column chromatography on silica gel (*n*hexane/EtOAc: 3/1) yielded **43a** (38.5 mg, 43%) as a syrup.

^{1}H NMR (300 MHz, $CDCl_3$): δ = 8.00 – 7.86 (m, 2H), 7.66 – 7.55 (m, 1H), 7.56 – 7.42 (m, 2H), 5.76 (dd, J = 10.3, 3.4 Hz, 1H), 5.69 (d, J = 6.4 Hz, 1H), 5.58 – 5.43 (m, 2H), 4.53 (td, J = 6.4, 1.7 Hz, 1H), 4.08 (dd, J = 6.4, 5.0 Hz, 2H), 2.16 (s, 3H), 2.00 (s, 3H), 1.91 (s, 3H), 1.76 (s, 3H).

^{13}C NMR (75 MHz, $CDCl_3$): δ = 196.55 (C_q), 170.35 (C_q), 170.06 (C_q), 169.71 (C_q), 136.06 (C_q), 133.90 (CH), 128.77 (CH), 128.66 (CH), 71.23 (CH), 71.05 (CH), 67.88 (CH), 67.77 (CH), 66.86 (CH), 61.70 (CH_2), 20.67 (CH_3), 20.63 (CH_3), 20.56 (CH_3), 20.45 (CH_3).

IR (ATR): $\tilde{v}$ = 1749, 1371, 1369, 1266, 1214, 1046, 909, 729, 702 cm^{-1} .

MS (ESI): m/z (relative intensity) 459 (100) $[M+Na]^+$, 895 (90) [2M+Na].

(2*R*,3*S*,4*S*,5*R*,6*R*)-2-(Acetoxymethyl)-6-benzoyltetrahydro-2*H*-pyran-3,4,5-triyl triacetate (43ba)

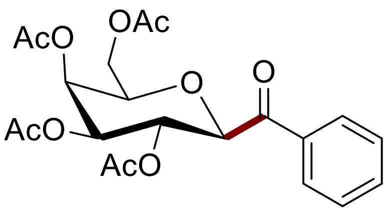

The general procedure **E** was followed using (2*R*,3*S*,4*S*,5*R*,6*R*)-2-(acetoxymethyl)-6-bromotetrahydro-2*H*-pyran-3,4,5-triyl triacetate **1a** (82.0 mg, 0.2 mmol) and benzoyl chloride **2bi** (42.0 mg, 0.3 mmol), Ni(acac)$_2$ (10 mol %), 2,2':6',2''-terpyridine **L11** (10 mol %), $LiClO_4$ (63.6 mg, 06 mmol), DIPEA (0.8 mmol), Purification by column chromatography on silica gel (*n*hexane/EtOAc: 3/1) yielded **43ba** (36.2 mg, 41%) as a syrup.

^{1}H NMR (300 MHz, $CDCl_3$): δ = 8.07 – 7.98 (m, 2H), 7.65 – 7.55 (m, 1H), 7.47 (dd, *J* = 8.4, 7.0 Hz, 2H), 5.68 (t, *J* = 9.8 Hz, 1H), 5.51 (d, *J* = 3.4 Hz, 1H), 5.20 (dd, *J* = 10.1, 3.4 Hz, 1H), 4.68 (**d, *J* = 9.8 Hz, 1H**), 4.20 – 4.05 (m, 3H), 2.19 (s, 3H), 2.04 (s, 3H), 2.00 (s, 3H), 1.84 (s, 3H).

^{13}C NMR (75 MHz, $CDCl_3$): δ = 192.29 (C_q), 170.33 (C_q), 170.24 (C_q), 170.18 (C_q), 168.99 (C_q), 134.85 (C_q), 133.85 (CH), 129.37 (CH), 128.51 (CH), 78.82 (CH), 75.21 (CH), 72.09 (CH), 67.37 (CH), 66.18 (CH), 61.76 (CH_2), 20.70 (CH_3), 20.65 (CH_3), 20.62 (CH_3), 20.48 (CH_3).

IR (ATR): $\tilde{\nu}$ = 1737, 1651, 1369, 1210, 1014, 899, 733, 602 cm^{-1} .

MS (ESI): m/z (relative intensity) 459 (100) $[M+Na]^+$, 895 (50) [2M+Na].

(2*R*,3*S*,4*S*,5*R*,6*R*)-2-(Acetoxymethyl)-6-tertbutylbenzoyltetrahydro-2*H*-pyran-3,4,5-triyl triacetate (43bb)

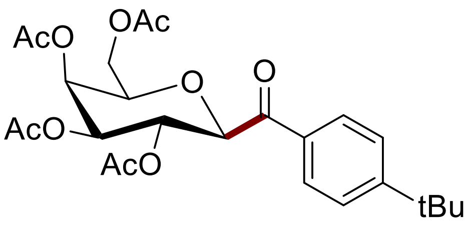

The general procedure **E** was followed using (2*R*,3*S*,4*S*,5*R*,6*R*)-2-(Acetoxymethyl)-6-tertbutylbenzoyltetrahydro-2*H*-pyran-3,4,5-triyl triacetate **1a** (82.0 mg, 0.2 mmol) and benzoyl chloride **2bj** (44.0 mg, 0.3 mmol), Ni(acac)$_2$ (10 mol %), 2,2':6',2''-terpyridine **L11** (10 mol %), $LiClO_4$ (63.6 mg, 06 mmol), DIPEA (0.8 mmol), Purification by column chromatography on silica gel (*n*hexane/EtOAc: 3/1) yielded **43bb** (34.9 mg, 35%) as a syrup.

^{1}H NMR (400 MHz, $CDCl_3$): δ = 8.01 (d, J = 8.6 Hz, 2H), 7.60 – 7.48 (m, 2H), 5.73 (t, J = 9.9 Hz, 1H), 5.58 – 5.55 (m, 1H), 5.28 – 5.23 (m, 1H), 4.71 (d, J = 9.8 Hz, 1H), 4.21 – 4.09 (m, 3H), 2.24 (s, 3H), 2.09 (s, J = 1.7 Hz, 3H), 2.05 (s, 3H), 1.88 (s, 3H), 1.39 (s, 9H).

^{13}C NMR (101 MHz, $CDCl_3$): δ = 191.90 (C_q), 170.41 (C_q), 170.32 (C_q), 170.28 (C_q), 169.07 (C_q), 157.81 (CH), 132.31 (CH), 129.40 (CH), 125.55 (CH), 78.93 (CH), 75.20 (CH), 72.19 (CH), 67.42 (CH), 66.26 (CH), 61.78 (CH_2), 31.04 (CH3), 20.78 (CH_3), 20.71 (CH_3), 20.68 (CH_3), 20.53 (CH_3).

IR (ATR): $\tilde{\nu}$ = 1747, 1695, 1604, 1367, 1218, 1047, 918, 848, 733 cm^{-1} .

MS (ESI): m/z (relative intensity) 515 (100) [M+Na]$^+$, 1007 (50) [2M+Na].

HR-MS (ESI): *m/z* calcd for $C_{25}H_{32}NaO_{10}^+$ [M+Na]$^+$: 515.1900, found: 515.1909.

(2*R*,3*S*,4*S*,5*R*,6*R*)-2-(Acetoxymethyl)-6-methoxybenzoyltetrahydro-2*H*-pyran-3,4,5-triyl triacetate (43bc)

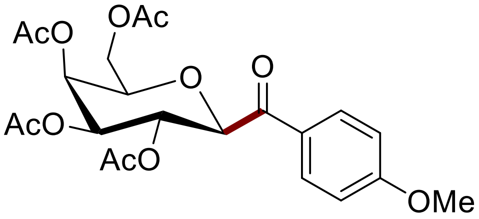

The general procedure **E** was followed using (2*R*,3*S*,4*S*,5*R*,6*R*)-2-(Acetoxymethyl)-6-methoxybenzoyltetrahydro-2*H*-pyran-3,4,5-triyl triacetate **1a** (82.0 mg, 0.2 mmol) and benzoyl chloride **2bk** (46.0 mg, 0.3 mmol), $Ni(acac)_2$ (10 mol %), 2,2':6',2''-terpyridine **L11** (10 mol %), $LiClO_4$ (63.6 mg, 06 mmol), DIPEA (0.8 mmol), Purification by column chromatography on silica gel (*n*hexane/EtOAc: 3/1) yielded **43bc** (42.8 mg, 46%) as a syrup.

^{1}H NMR (400 MHz, $CDCl_3$): δ = 8.17 – 7.90 (m, 2H), 7.05 – 6.94 (m, 2H), 5.72 (t, *J* = 9.9 Hz, 1H), 5.55 (dd, *J* = 3.4, 1.0 Hz, 1H), 5.23 (dd, *J* = 10.1, 3.4 Hz, 1H), 4.26 – 4.08 (m, 1H), 3.92 (s, 3H), 2.23 (s, 3H), 2.09 (s, 3H), 2.04 (s, 3H), 1.91 (s, 3H).

^{13}C NMR (101 MHz, $CDCl_3$): δ = 190.71 (C_q), 170.41 (C_q), 170.32 (C_q), 170.26 (C_q), 169.05 (C_q), 164.10 (CH), 131.83 (CH), 127.90 (CH), 113.79 (CH), 78.94 (CH), 75.18 (CH), 72.19 (CH), 67.45 (CH), 66.33 (CH), 61.82 (CH_2), 55.57 (CH3), 20.76 (CH_3), 20.72 (CH_3), 20.67 (CH_3), 20.60 (CH_3).
IR (ATR): ṽ = 1757, 1667, 1334, 1267, 1023, 902, 767, 712, 605 cm^{-1} .
MS (ESI): m/z (relative intensity) 489 (100) $[M+Na]^+$, 955 (100) [2M+Na].
HR-MS (ESI): *m/z* calcd for $C_{22}H_{26}NaO_{11}^+$ $[M+Na]^+$: 489.1367, found: 489.1357.

(2*R*,3*S*,4*S*,5*R*,6*R*)-2-(Acetoxymethyl)-6-methylbenzoyltetrahydro-2*H*-pyran-3,4,5-triyl triacetate (43bd)

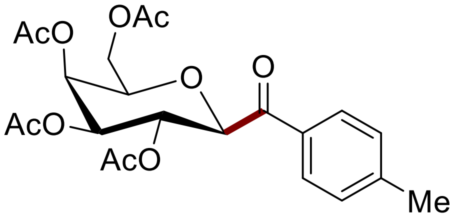

The general procedure **E** was followed using (2*R*,3*S*,4*S*,5*R*,6*R*)-2-(Acetoxymethyl)-6-methylbenzoyltetrahydro-2*H*-pyran-3,4,5-triyl triacetate **1a** (82.0 mg, 0.2 mmol) and benzoyl chloride **2bl** (43.0 mg, 0.3 mmol), $Ni(acac)_2$ (10 mol %), 2,2':6',2''-terpyridine **L11** (10 mol %), $LiClO_4$ (63.6 mg, 06 mmol), DIPEA (0.8 mmol), Purification by column chromatography on silica gel (*n*hexane/EtOAc: 3/1) yielded **43bd** (38 mg, 42%) as a syrup.

^{1}H NMR (400 MHz, $CDCl_3$): δ = 8.02 – 7.93 (m, 2H), 7.31 (d, *J* = 7.3 Hz, 2H), 5.72 (t, *J* = 9.9 Hz, 1H), 5.55 (dd, *J* = 3.4, 1.0 Hz, 1H), 5.24 (dd, *J* = 10.1, 3.4 Hz, 1H), 4.70 (d, *J* = 9.8 Hz, 1H), 4.27 – 4.04 (m, 3H), 2.47 (s, 3H), 2.23 (s, 3H), 2.09 (s, 3H), 2.05 (s, 3H), 1.89 (s, 3H).

^{13}C NMR (101 MHz, $CDCl_3$): δ = 191.91 (C_q), 170.41 (C_q), 170.31 (C_q), 170.25 (C_q), 169.04 (C_q), 144.95 (CH), 132.41 (CH), 129.54 (CH), 129.27 (CH), 78.92 (CH), 75.21 (CH), 72.17 (CH), 67.44 (CH), 66.29 (CH), 61.82 (CH_2), 21.86 (CH3), 20.76 (CH_3), 20.72 (CH_3), 20.67 (CH_3), 20.56 (CH_3).

IR (ATR): ṽ = 1749, 1678, 1323, 1234, 1098, 914, 787 756, 609 cm^{-1} .

MS (ESI): m/z (relative intensity) 473 (100) $[M+Na]^+$, 923 (50) [2M+Na].

HR-MS (ESI): *m/z* calcd for $C_{22}H_{26}NaO_{10}^+$ $[M+Na]^+$: 473.1418, found: 473.1441.

(2*R*,3*S*,4*S*,5*R*,6*R*)-2-(Acetoxymethyl)-6-2-(naphthalen-2-yl benzoyltetrahydro-2*H*-pyran-3,4,5-triyl triacetate (43be)

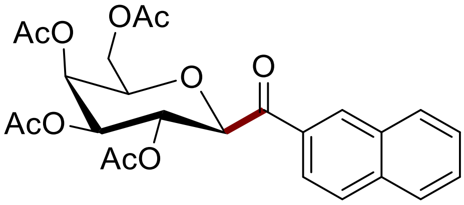

The general procedure **E** was followed using (2*R*,3*S*,4*S*,5*R*,6*R*)-2-(Acetoxymethyl)-6-2-(naphthalen-2-yl benzoyltetrahydro-2*H*-pyran-3,4,5-triyl triacetate **1a** (82.0 mg, 0.2 mmol) and benzoyl chloride **2bm** (50.0 mg, 0.3 mmol), Ni(acac)$_2$ (10 mol %), 2,2':6',2''-terpyridine **L11** (10 mol %), $LiClO_4$ (63.6 mg, 06 mmol), DIPEA (0.8 mmol), Purification by column chromatography on silica gel (*n*hexane/EtOAc: 3/1) yielded **43be** (23.2 mg, 23%) as a syrup.

^{1}H NMR (400 MHz, $CDCl_3$): δ = 8.69 – 8.55 (m, 1H), 8.17 – 8.03 (m, 2H), 7.93 (d, *J* = 7.7 Hz, 1H), 7.77 – 7.50 (m, 3H), 5.83 – 5.69 (m, 1H), 5.55 (dd, *J* = 3.5, 1.0 Hz, 1H), 5.24 (dd, *J* = 10.0, 3.4 Hz, 1H), 4.87 (d, *J* = 9.8 Hz, 1H), 4.25 – 4.05 (m, 3H), 2.25 (s, 3H), 2.07 (s, 3H), 2.04 (s, 3H), 1.73 (s, 3H).

^{13}C NMR (101 MHz, $CDCl_3$): δ = 190.41 (C_q), 170.41 (C_q), 170.34 (C_q), 170.27 (C_q), 168.97 (C_q), 144.95 (CH), 133.94 (CH), 133.91 (CH), 133.89 (CH), 133.14 (CH), 130.67 (CH), 129.38 (CH), 128.53 (CH), 126.77 (CH), 125.66 (CH), 124.08 (CH), 79.66 (CH), 75.16 (CH), 72.28 (CH), 67.39 (CH), 66.69 (CH), 61.67(CH), 20.79 (CH_3), 20.70 (CH_3), 20.67 (CH_3), 20.37 (CH_3).

IR (ATR): ṽ = 1752, 1676, 1371, 1267, 1109, 899, 763, 610 cm^{-1} .
MS (ESI): m/z (relative intensity) 509 (100) [M+Na]$^+$, 995 (50) [2M+Na].
HR-MS (ESI): *m/z* calcd for $C_{25}H_{26}NaO_{10}^+$ [M+Na]$^+$: 509.1418, found: 509.1426.

(2*R*,3*S*,4*R*,5*S*,6*R*)-2-[(1,1'-biphenyl)-4-yl]-6-(acetoxymethyl)tetrahydro-2*H*-pyran-3,4,5-triyl triacetate (44)

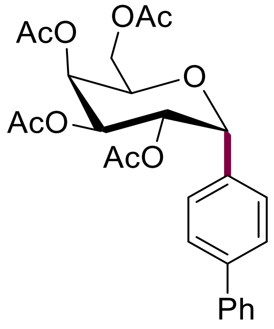

The general procedure **C** was followed using (2*R*,3*S*,4*S*,5*R*,6*R*)-2-(acetoxymethyl)-6-bromotetrahydro-2*H*-pyran-3,4,5-triyl triacetate **1a** (82.0 mg, 0.2 mmol) and **2aa** (54.0 mg, 0.3 mmol), Ni(acac)$_2$ (10 mol %), Neocuproin **L6** (10 mol %), $LiClO_4$ (63.6 mg, 06 mmol), DIPEA (0.8 mmol), Purification by column chromatography on silica gel (*n*hexane/EtOAc: 3/1) yielded **44** (61 mg, 63%) as a syrup.

^{1}H NMR (400 MHz, $CDCl_3$): δ = 7.64 – 7.52 (m, 6H), 7.44 (t, *J* = 7.6 Hz, 2H), 7.39 – 7.31 (m, 1H), 5.57 – 5.42 (m, 3H), 5.35 (d, *J* = 3.6 Hz, 1H), 4.61 – 4.41 (m, 1H), 4.29 – 4.10 (m, 2H), 2.14 (s, 3H), 2.14 (s, 3H), 2.03 (s, 3H), 1.97 (s, 3H).

^{13}C NMR (101 MHz, $CDCl_3$): *δ* = 170.64 (C_q), 169.87 (C_q), 169.54 (C_q), 169.41 (C_q), 140.91 (C_q), 140.41 (C_q), 134.82 (C_q), 128.77 (CH), 127.79 (CH), 127.45 (C_q), 127.03 (CH), 127.02 (CH), 71.38 (CH), 70.54 (CH), 69.82 (CH) (CH), 67.77 (CH), 66.94 (CH), 60.43 (CH_2), 20.83 (CH_3), 20.74 (CH_3), 20.71 (CH_3), 20.66 (CH_3).

IR (ATR): ṽ = 1652, 1558, 1541, 1369, 1205, 907, 725, 724 cm^{-1} .

MS (ESI): m/z (relative intensity) 507 (100) $[M+Na]^+$, 991 (50) [2M+Na].

HR-MS (ESI): *m/z* calcd for $C_{26}H_{28}NaO_9^+$ $[M+Na]^+$: 507.1626, found: 507.1638.

(2*R*,3*S*,4*R*,5*S*,6*R*)-2-(Acetoxymethyl)-6-[4-(trifluoromethyl)phenyl]tetrahydro-2*H*-pyran-3,4,5-triyl triacetate (45)

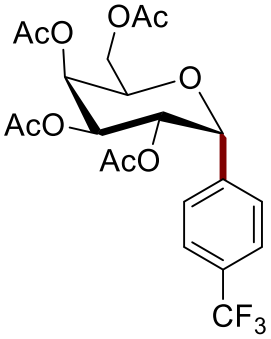

The general procedure **C** was followed using (2*R*,3*S*,4*S*,5*R*,6*R*)-2-(acetoxymethyl)-6-bromotetrahydro-2*H*-pyran-3,4,5-triyl triacetate **1a** (82.0 mg, 0.2 mmol) and 1-iodo-4-(trifluoromethyl)benzene **2bc** (81.6 mg, 0.3 mmol), Ni(acac)$_2$ (10 mol %), Neocuproin **L6** (10 mol %), LiClO$_4$ (63.6 mg, 06 mmol), DIPEA (0.8 mmol), Purification by column chromatography on silica gel (*n*hexane/EtOAc: 3/1) yielded **45** (71.4 mg, 75%) as a syrup.

^{1}H NMR (400 MHz, CDCl$_3$): δ = 7.74 – 7.52 (m, 4H), 5.48 (dd, *J* = 4.9, 3.0 Hz, 1H), 5.42 – 5.34 (m, 2H), 5.31 (d, *J* = 3.0 Hz, 1H), 4.65 (dd, *J* = 12.1, 8.6 Hz, 1H), 4.24 (dt, *J* = 8.8, 4.4 Hz, 1H), 4.12 (dd, *J* = 12.2, 4.0 Hz, 1H), 2.14 (s, 3H), 2.12 (s, 3H), 2.02 (s, 3H), 1.91 (s, 3H).

^{13}C NMR (101 MHz, CDCl$_3$): δ = 170.69 (C$_q$), 169.69 (C$_q$), 169.30 (C$_q$), 169.14 (C$_q$), 140.13 (C$_q$, q, $^4J_{C-F}$ = 1.6 Hz), 130.24 (C$_q$, q, $^2J_{C-F}$ = 32.6 Hz), 127.44 (CH), 125.20 (CH, $^3J_{C-F}$ = 3.9 Hz), 71.33 (CH), 70.44 (CH), 69.86 (CH), 67.56 (CH), 66.36 (CH), 59.89 (CH$_2$), 20.83 (CH$_3$), 20.72 (CH$_3$), 20.68 (CH$_3$), 20.47 (CH$_3$).

^{19}F NMR (377 MHz, CDCl$_3$): δ = -62.65.

IR (ATR): ṽ = 1745, 1729, 1369, 1204, 1002, 967, 914, 731, 602 cm^{-1} .
MS (ESI): m/z (relative intensity) 499 (100) [M+Na]$^+$, 975 (50) [2M+Na].
HR-MS (ESI): *m/z* calcd for $C_{21}H_{23}F_3NaO_9^+$ [M+Na]$^+$: 499.1186, found: 499.1197

(2*R*,3*S*,4*R*,5*S*,6*R*)-2-(Acetoxymethyl)-6-[3,5-bis(trifluoromethyl)phenyl]tetrahydro-2*H*-pyran-3,4,5-triyl triacetate (46)

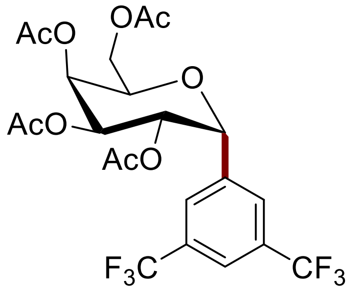

The general procedure **C** was followed using (2*R*,3*S*,4*S*,5*R*,6*R*)-2-(acetoxymethyl)-6-bromotetrahydro-2*H*-pyran-3,4,5-triyl triacetate **1a** (82.0 mg, 0.2 mmol) and 1-iodo-3,5-bis(trifluoromethyl)benzene **2bd** (102.0 mg, 0.3 mmol), Ni(acac)$_2$ (10 mol %), Neocuproin **L6** (10 mol %), LiClO$_4$ (63.6 mg, 06 mmol), DIPEA (0.8 mmol), Purification by column chromatography on silica gel (*n*hexane/EtOAc: 3/1) yielded **46** (80.5 mg, 74%) as a syrup.

^{1}H NMR (300 MHz, CDCl$_3$): δ = 7.90 (s, 2H), 7.83 (s, 1H), 5.52 (dd, *J* = 5.5, 3.2 Hz, 1H), 5.35 (t, *J* = 2.7 Hz, 2H), 5.29 (dd, *J* = 5.5, 2.7 Hz, 1H), 4.81 (dd, *J* = 12.5, 9.4 Hz, 1H), 4.39 – 4.29 (m, 1H), 4.13 (dd, *J* = 12.5, 3.1 Hz, 1H), 2.19 (s, 3H), 2.12 (s, 3H), 2.05 (s, 3H), 1.92 (s, 3H).

^{13}C NMR (101 MHz, CDCl$_3$): δ = 170.84 (C$_q$) , 169.55 (C$_q$), 169.08 (C$_q$), 168.83 (C$_q$), 139.02 (C$_q$), 131.57 (C$_q$, q, $^2J_{C-F}$ = 33.4 Hz), 127.46 (CH, t, $^3J_{C-F}$ = 3.9 Hz), 123.16 (C$_q$, d, $^1J_{C-F}$ = 272.8 Hz), 122.04 (CH, p, $^3J_{C-F}$ = 3.8 Hz), 72.12 (CH), 69.86 (CH), 69.54 (CH), 67.35 (CH), 65.89 (CH), 59.65 (CH$_2$), 20.86 (CH$_3$), 20.71 (CH$_3$), 20.68 (CH$_3$), 20.26 (CH$_3$).

^{19}F NMR (282 MHz, CDCl$_3$): δ = -62.87.

IR (ATR): ṽ = 1745, 1371, 1278, 1207, 1169, 889, 729, 681, 602 cm^{-1} .
MS (ESI): m/z (relative intensity) 567 (100) [M+Na]$^+$, 1111 (50) [2M+Na].
HR-MS (ESI): *m/z* calcd for $C_{22}H_{22}F_6NaO_9^+$ [M+Na]$^+$: 567.1060, found: 567.1072.

(2*R*,3*S*,4*R*,5*S*,6*R*)-2-(acetoxymethyl)-6-(4-cyanophenyl)tetrahydro-2*H*-pyran-3,4,5-triyl triacetate (47)

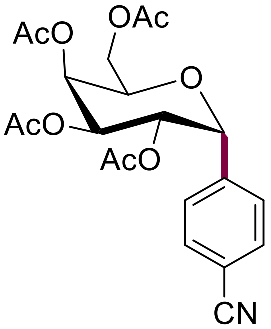

The general procedure **C** was followed using (2*R*,3*S*,4*S*,5*R*,6*R*)-2-(acetoxymethyl)-6-bromotetrahydro-2*H*-pyran-3,4,5-triyl triacetate **1a** (82.0 mg, 0.2 mmol) and 4-iodobenzonitrile **2be** (68.7 mg, 0.3 mmol), Ni(acac)$_2$ (10 mol %), Neocuproin **L6** (10 mol %), $LiClO_4$ (63.6 mg, 06 mmol), DIPEA (0.8 mmol), Purification by column chromatography on silica gel (*n*hexane/EtOAc: 3/1) yielded **47** (65.8 mg, 76%) as a syrup.

^{1}H NMR (400 MHz, $CDCl_3$): δ = 7.64 (d, *J* = 8.4 Hz, 2H), 7.59 – 7.49 (m, 2H), 5.48 (dd, *J* = 5.2, 3.1 Hz, 1H), 5.36 (dd, *J* = 5.8, 3.1 Hz, 1H), 5.32 (dd, *J* = 5.8, 2.9 Hz, 1H), 5.29 (d, *J* = 2.9 Hz, 1H), 4.72 (dd, *J* = 12.2, 8.9 Hz, 1H), 4.28 (ddd, *J* = 8.9, 5.2, 3.8 Hz, 1H), 4.11 (dd, *J* = 12.3, 3.8 Hz, 1H), 2.16 (s, 3H), 2.11 (s, 3H), 2.02 (s, 3H), 1.90 (s, 3H).

^{13}C NMR (101 MHz, $CDCl_3$): δ = 170.73 (C_q), 169.61 (C_q), 169.19 (C_q), 169.00 (C_q), 141.53 (C_q), 132.01 (CH), 127.69 (CH), 118.45 (C_q), 111.98 (C_q), 71.68 (CH), 70.09 (CH), 69.84 (C_q), 67.49 (CH), 66.06 (CH), 59.63 (CH_2), 20.85 (CH_3), 20.74 (CH_3), 20.68 (CH_3), 20.44 (CH_3).

IR (ATR): ṽ = 1748, 1745, 1370, 1278, 1235, 976, 954, 708, 607 cm^{-1} .
MS (ESI): m/z (relative intensity) 456 (100) $[M+Na]^+$, 889 (50) [2M+Na].
HR-MS (ESI): *m/z* calcd for $C_{21}H_{23}NNaO_9^+$ $[M+Na]^+$: 456.1265, found: 456.1265.

(2*R*,3*S*,4*R*,5*S*,6*R*)-2-(Acetoxymethyl)-6-(4-acetylphenyl)tetrahydro-2*H*-pyran-3,4,5-triyl triacetate (48)

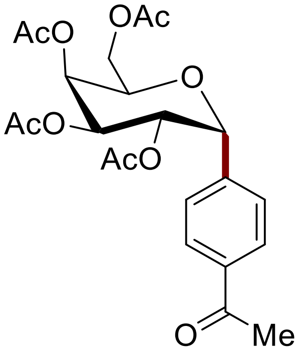

The general procedure **C** was followed using (2*R*,3*S*,4*S*,5*R*,6*R*)-2-(acetoxymethyl)-6-bromotetrahydro-2*H*-pyran-3,4,5-triyl triacetate **1a** (82.0 mg, 0.2 mmol) and 1-(4-iodophenyl)ethan-1-one **2bf** (73.8 mg, 0.3 mmol), Ni(acac)$_2$ (10 mol %), Neocuproin **L6** (10 mol %), LiClO$_4$ (63.6 mg, 06 mmol), DIPEA (0.8 mmol), Purification by column chromatography on silica gel (*n*hexane/EtOAc: 3/1) yielded **48** (72 mg, 80%) as a syrup.

^{1}H NMR (400 MHz, CDCl$_3$): *δ* = 7.93 (d, *J* = 8.4 Hz, 2H), 7.54 (d, *J* = 8.5 Hz, 2H), 5.53 – 5.27 (m, 4H), 4.64 (dd, *J* = 12.0, 8.7 Hz, 1H), 4.24 (dt, *J* = 8.9, 4.4 Hz, 1H), 4.13 (dd, *J* = 12.1, 3.7 Hz, 1H), 2.59 (s, 3H), 2.15 (s, 3H), 2.11 (s, 3H), 2.02 (s, 3H), 1.91 (s, 3H).

^{13}C NMR (101 MHz, CDCl$_3$): δ = 197.66 (C$_q$), 170.77 (C$_q$), 169.76 (C$_q$), 169.37 (C$_q$), 169.22 (C$_q$), 141.39 (C$_q$), 136.74 (C$_q$), 128.32 (CH), 127.32 (CH), 71.36 (CH), 70.66 (CH), 69.94 (CH), 67.67 (CH), 66.44 (CH), 59.99 (CH$_2$), 26.67 (CH$_3$), 20.90 (CH$_3$), 20.80 (CH$_3$), 20.75 (CH$_3$), 20.58 (CH$_3$).

IR (ATR): ṽ = 1745, 1550, 1321, 1235, 901, 728, 649 cm^{-1} .
MS (ESI): m/z (relative intensity) 473 (100) [M+Na]$^+$, 923 (50) [2M+Na].
HR-MS (ESI): *m/z* calcd for $C_{22}H_{26}NaO_{10}^+$ [M+Na]$^+$: 473.1418, found: 473.1436.

(2*R*,3*S*,4*R*,5*S*,6*R*)-2-(Acetoxymethyl)-6-[4-(2-ethoxy-2-oxoethyl)phenyl]tetrahydro-2*H*-pyran-3,4,5-triyl triacetate (49)

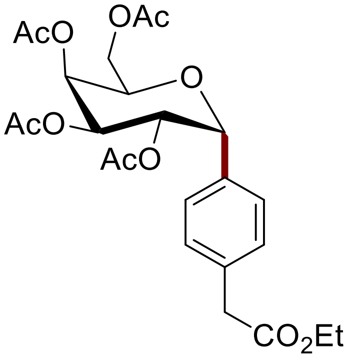

The general procedure **C** was followed using (2*R*,3*S*,4*S*,5*R*,6*R*)-2-(acetoxymethyl)-6-bromotetrahydro-2*H*-pyran-3,4,5-triyl triacetate **1a** (82.0 mg, 0.2 mmol) and ethyl 2-(4-iodophenyl)acetate **2bg** (87.0 mg, 0.3 mmol), $Ni(acac)_2$ (10 mol %), Neocuproin **L6** (10 mol %), $LiClO_4$ (63.6 mg, 06 mmol), DIPEA (0.8 mmol), Purification by column chromatography on silica gel (*n*hexane/EtOAc: 3/1) yielded **49** (68 mg, 69%) as a syrup.

^{1}H NMR (400 MHz, $CDCl_3$): δ = 7.44 (d, J = 8.2 Hz, 2H), 7.27 (d, J = 8.2 Hz, 2H), 5.55 – 5.37 (m, 3H), 5.28 (d, J = 3.6 Hz, 1H), 4.47 (dd, J = 13.0, 9.3 Hz, 1H), 4.21 – 4.07 (m, 4H), 3.60 (s, 2H), 2.13 (s, 3H), 2.12 (s, 3H), 2.02 (s, 3H), 1.95 (s, 3H), 1.25 (t, J = 7.1 Hz, 3H).

^{13}C NMR (101 MHz, $CDCl_3$): δ = 171.36 (C_q), 170.66 (C_q), 169.89 (C_q), 169.56 (C_q), 169.41 (C_q), 134.65 (C_q), 134.06 (C_q), 129.29 (CH), 127.58 (CH), 71.38 (CH), 70.46 (CH), 69.81 (CH), 67.76 (CH), 66.96 (CH), 60.92 (CH_2), 60.44 (CH_2), 41.03 (CH_2), 20.84 (CH_3), 20.76 (CH_3), 20.73 (CH_3), 20.66 (CH_3), 14.16 (CH_3).

IR (ATR): $\tilde{\nu}$ = 1745, 1371, 1278, 1207, 1169, 889, 729, 681, 602 cm^{-1}.
MS (ESI): m/z (relative intensity) 517 (100) $[M+Na]^+$, 1011 (50) [2M+Na].
HR-MS (ESI): *m/z* calcd for $C_{81}H_{77}NNaO_{23}^+$ $[M+Na]^+$: 517.1680, found: 517.1689.

(2*R*,3*R*,4*S*,5*R*,6*S*)-2-(Acetoxymethyl)-6-({[(2*R*,3*R*,4*R*,5*S*,6*R*)-3,4,5-triacetoxy-6-[4-(methoxycarbonyl)phenyl]tetrahydro-2*H*-pyran-2-yl]methoxy}tetrahydro-2*H*-pyran-3,4,5-triyl triacetate (50)

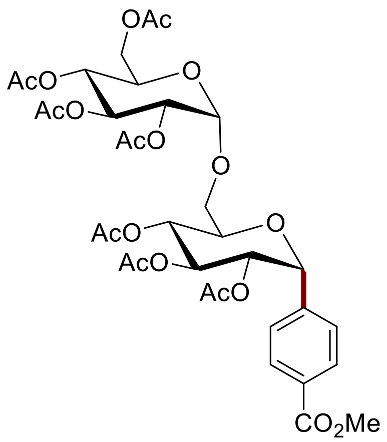

The general procedure **C** was followed using (2*R*,3*R*,4*S*,5*R*,6*S*)-2-(acetoxymethyl)-6-{[(2*R*,3*R*,4*S*,5*R*,6*R*)-3,4,5-triacetoxy-6-bromotetrahydro-2*H*-pyran-2-yl]methoxy}tetrahydro-2*H*-pyran-3,4,5-triyl triacetate **1r** (139.6 mg, 0.2 mmol) and methyl 4-iodobenzoate (78.3 mg, 0.3 mmol), $Ni(acac)_2$ (10 mol %), Neocuproin **L6** (10 mol %), $LiClO_4$ (63.6 mg, 06 mmol), DIPEA (0.6 mmol), Purification by column chromatography on silica gel (*n*hexane/EtOAc: 3/1) yielded **50** (73.9 mg, 49%) as a syrup.

^{1}H NMR (400 MHz, $CDCl_3$): δ = 8.07 (d, *J* = 8.5 Hz, 2H), 7.62 (d, *J* = 8.5 Hz, 2H), 5.52 (dd, *J* = 10.3, 9.3 Hz, 1H), 5.44 (ddd, *J* = 7.1, 5.2, 1.3 Hz, 1H), 5.33 (d, *J* = 5.3 Hz, 2H), 5.16 – 5.03 (m, 3H), 4.84 (dd, *J* = 10.3, 3.6 Hz, 1H), 4.25 – 4.08 (m, 2H), 4.07 – 3.97 (m, 1H), 3.92 (s, 3H), 3.91 – 3.90 (m, 1H), 3.82 (dd, *J* = 10.5, 6.1 Hz, 1H), 3.56 (dd, *J* = 10.5, 3.3 Hz, 1H), 2.11 (s, 3H), 2.08 (s, 3H), 2.05 (s, 3H), 2.02 (s, 3H), 2.02 (s, 3H), 1.92 (s, 6H).

^{13}C NMR (101 MHz, $CDCl_3$): δ = 170.58 (C_q), 170.28 (C_q), 169.94 (C_q), 169.64 (C_q), 169.60 (C_q), 169.49 (C_q), 169.30 (C_q), 166.63 (C_q), 141.07 (C_q), 129.93 (C_q), 129.77 (CH), 127.38 (CH), 95.64 (CH), 72.62 (CH), 72.06 (CH), 70.85 (CH), 70.70 (CH), 70.45 (CH), 69.85 (CH), 68.32 (CH), 68.25 (CH), 67.44 (CH), 66.70 (CH), 61.68 (CH), 52.19 (CH_3), 20.76 (CH_3), 20.72 (2 CH_3), 20.69 (CH_3), 20.60 (CH_3), 20.54 (CH_3), 20.44 (CH_3).

IR (ATR): ṽ = 1750, 1721, 1368, 1283, 1221, 1112, 1038, 704 cm^{-1} .
MS (ESI): m/z (relative intensity) 777 (100) $[M+Na]^+$.
HR-MS (ESI): *m/z* calcd for $C_{34}H_{42}NaO_{19}^+$ $[M+Na]^+$: 777.2213, found: 777.2220.

(2*R*,3*S*,4*S*,5*R*,6*S*)-2-[(Benzoyloxy)methyl]-6-{[(2*R*,3*R*,4*S*,5*S*,6*S*)-4,5-bis(benzoyloxy)-2-[(benzoyloxy)methyl]-6-[4 (methoxycarbonyl)phenyl]tetrahydro-2*H*-pyran-3-yl]oxy}tetrahydro-2*H*-pyran-3,4,5-triyl tribenzoate (51)

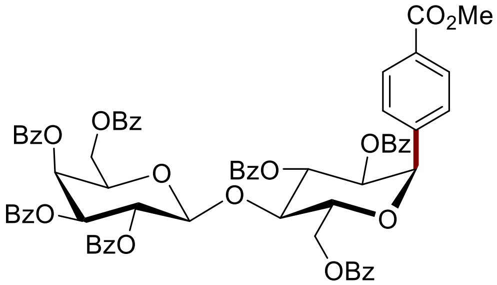

The general procedure **C** was followed using (2*R*,3*S*,4*S*,5*R*,6*S*)-2-[(benzoyloxy)methyl]-6-{[(2*R*,3*R*,4*S*,5*R*,6*R*)-4,5-bis(benzoyloxy)-2-[(benzoyloxy)methyl]-6-bromotetrahydro-2*H*-pyran-3-yl]oxy}tetrahydro-2*H*-pyran-3,4,5-triyl tribenzoate **1p** (226.4 mg, 0.2 mmol) and ethyl 2-(4-iodophenyl)acetate **2ba** (87.0 mg, 0.3 mmol), $Ni(acac)_2$ (10 mol %), Neocuproin **L6** (10 mol %), $LiClO_4$ (63.6 mg, 06 mmol), DIPEA (0.6 mmol), Purification by column chromatography on silica gel (*n*hexane/EtOAc: 3/1) yielded **51** (73.7 mg, 31%) as a syrup.

^{1}H NMR (400 MHz, $CDCl_3$): δ = 8.12 – 8.05 (m, 2H), 8.05 – 7.98 (m, 4H), 7.98 – 7.88 (m, 6H), 7.86 – 7.77 (m, 2H), 7.73 (dd, *J* = 7.7, 2.4 Hz, 4H), 7.63 – 7.54 (m, 2H), 7.53 – 7.44 (m, 4H), 7.44 – 7.31 (m, 12H), 7.25 – 7.16 (m, 3H), 6.19 (dd, *J* = 8.2, 6.2 Hz, 1H), 5.83 – 5.78 (m, 2H), 5.69 (dd, *J* = 10.3, 8.0 Hz, 1H), 5.58 (d, *J* = 5.2 Hz, 1H), 5.47 (dd, *J* = 10.4, 3.4 Hz, 1H), 5.04 (d, *J* = 8.0 Hz, 1H), 4.47 (t, *J* = 3.8 Hz, 2H), 4.26 (dd, *J* = 8.6, 6.2 Hz, 1H), 4.11 – 3.94 (m, 4H), 3.90 (s, 3H).

^{13}C NMR (101 MHz, $CDCl_3$): δ = 166.67 (C_q), 165.82 (C_q), 165.70 (C_q), 165.47 (C_q), 165.44 (C_q), 165.35 (C_q), 165.31 (C_q), 164.98 (C_q), 141.27 (C_q), 133.56 (CH), 133.46 (CH), 133.43 (CH), 133.36 (CH), 133.28 (CH), 133.23 (CH), 130.02 (CH), 129.93 (CH), 129.89 (CH), 129.77 (CH), 129.75 (CH), 129.70 (CH), 129.67 (CH), 129.59 (CH), 129.56 (C_q), 129.49 (C_q), 129.28 (C_q), 128.94 (C_q), 128.88 (C_q), 128.76 (C_q), 128.66 (C_q), 128.61 (C_q), 128.55 (CH), 128.52 (CH), 128.47 (CH), 128.27 (CH), 127.61 (CH), 101.78 (CH), 73.09 (CH), 71.87 (CH), 71.84 (CH), 71.53 (CH), 70.73 (CH), 70.47 (CH), 69.83 (CH), 67.72 (CH), 62.74 (CH_2), 61.42 (CH_2), 52.18 (CH_3).

IR (ATR): ṽ = 1729, 1720, 1271, 1245, 1109, 1092, 1068, 1027, 708 cm^{-1}.

MS (ESI): m/z (relative intensity) 1211 (100) $[M+Na]^+$.

HR-MS (ESI): *m/z* calcd for $C_{69}H_{56}NaO_{19}^+$ [M+Na]+: 1211.3308, found: 1211.3290.

(2*R*,3*R*,4*S*,5*R*,6*S*)-2-(Acetoxymethyl)-6-{[(2*R*,3*R*,4*S*,5*S*,6*R*)-4,5-diacetoxy-2-(acetoxymethyl)-6-[4-(methoxycarbonyl)phenyl]tetrahydro-2*H*-pyran-3-yl]oxy}tetrahydro-2*H*-pyran-3,4,5-triyl triacetate (52)

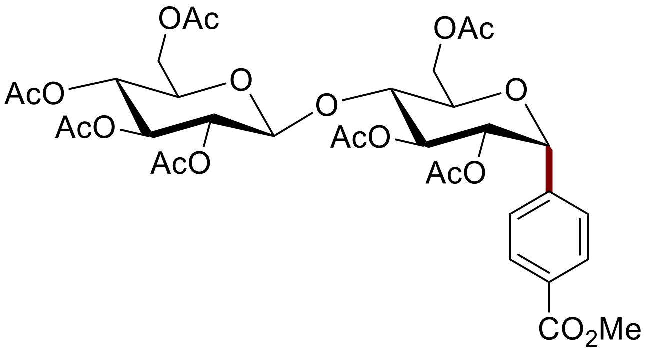

The general procedure **C** was followed using (2*R*,3*R*,4*S*,5*R*,6*S*)-2-(acetoxymethyl)-6-{[(2*R*,3*R*,4*S*,5*R*,6*R*)-4,5-diacetoxy-2-(acetoxymethyl)-6-bromotetrahydro-2*H*-pyran-3-yl]oxy}tetrahydro-2*H*-pyran-3,4,5-triyl triacetate **1q** (139.6 mg, 0.2 mmol), methyl 4-iodobenzoate **2ba** (78.3 mg, 0.3 mmol), Ni(acac)$_2$ (10 mol %), Neocuproin **L6** (10 mol %), $LiClO_4$ (63.6 mg, 06 mmol), DIPEA (0.6 mmol), Purification by column chromatography on silica gel (*n*hexane/EtOAc: 10/1 to 5/1) yielded **52** (70.9 mg, 47%) as a syrup.

^{1}H NMR (400 MHz, $CDCl_3$): *δ* = 8.02 (d, *J* = 8.4 Hz, 2H), 7.53 (d, *J* = 8.3 Hz, 2H), 5.53 (td, *J* = 4.5, 1.7 Hz, 1H), 5.26 (d, *J* = 4.1 Hz, 2H), 5.16 (t, *J* = 9.4 Hz, 1H), 5.05 (t, *J* = 9.7 Hz, 1H), 4.91 (dd, *J* = 9.5, 8.0 Hz, 1H), 4.64 (d, *J* = 8.0 Hz, 1H), 4.31 (ddd, *J* = 12.8, 8.8, 3.9 Hz, 2H), 4.22 (dd, *J* = 11.8, 6.6 Hz, 1H), 4.05 (dd, *J* = 12.3, 2.3 Hz, 1H), 3.91 (s, 3H), 3.89 (dd, *J* = 4.7, 2.5 Hz, 1H), 3.78 – 3.65 (m, 3H), 2.13 (s, 3H), 2.12 (s, 3H), 2.07 (s, 3H), 2.02 (s, 3H), 2.00 (s, 3H), 1.97 (s, 3H), 1.90 (s, 3H).

^{13}C NMR (101 MHz, $CDCl_3$): *δ* = 170.54 (C_q), 170.40 (C_q), 170.18 (C_q), 169.62 (C_q), 169.44 (C_q), 169.32 (C_q), 169.11 (C_q), 166.67 (C_q), 141.63 (C_q), 129.75 (C_q), 129.68 (CH), 126.84 (CH), 101.21 (CH), 76.44 (CH), 72.87 (CH), 72.06 (CH), 71.97 (CH), 71.92 (CH), 71.39 (CH), 69.37 (CH), 68.88 (CH), 67.85 (CH), 62.22 (CH), 61.61 (CH), 52.15 (CH_2), 20.81 (CH_3), 20.80 (CH_3), 20.63 (CH_3), 20.55 (CH_3), 20.53 (3 CH_3).

IR (ATR): ṽ = 1739, 1720, 1366, 1281, 1207, 1033, 906, 734, 600 cm^{-1}.

MS (ESI): m/z (relative intensity) 777 (100) [M+Na]+.

HR-MS (ESI): *m/z* calcd for $C_{34}H_{42}NaO_{19}^+$ [M+Na]+: 777.2213, found: 777.2213.

(2*R*,3*R*,4*S*,5*R*,6*R*)-2-(Acetoxymethyl)-6-{[(2*R*,3*R*,4*S*,5*R*,6*R*)-4,5-diacetoxy-2-(acetoxymethyl)-6-{[(2*R*,3*R*,4*S*,5*S*,6*R*)-4,5-diacetoxy-2-(acetoxymethyl)-6-[4-(methoxycarbonyl)phenyl]tetrahydro-2*H*-pyran-3-yl]oxy}tetrahydro-2*H*-pyran-3-yl]oxy}tetrahydro-2*H*-pyran-3,4,5-triyl triacetate (53)

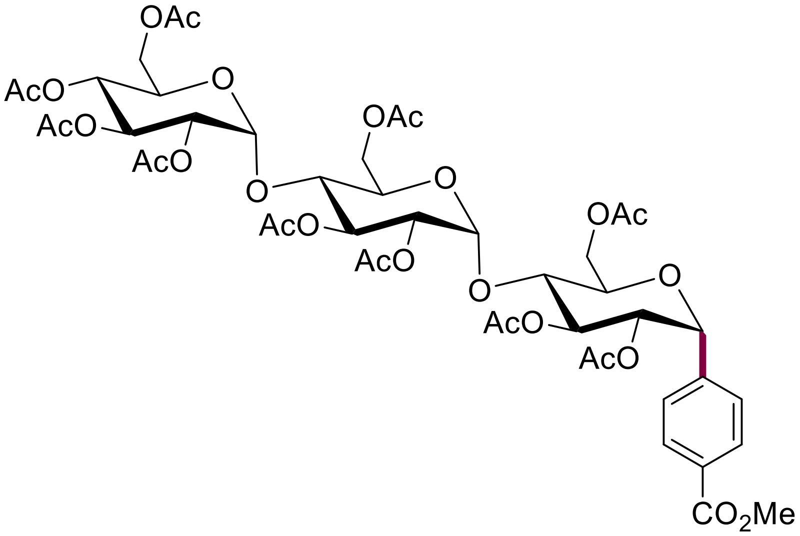

The general procedure **C** was followed using (2*R*,3*R*,4*S*,5*R*,6*R*)-2-(acetoxymethyl)-6-{[(2*R*,3*R*,4*S*,5*R*,6*R*)-4,5-diacetoxy-2-(acetoxymethyl)-6-{[(2*R*,3*R*,4*S*,5*R*,6*R*)-4,5-diacetoxy-2-(acetoxymethyl)-6-bromotetrahydro-2*H*-pyran-3-yl]oxy}tetrahydro-2*H*-pyran-3-yl]oxy}tetrahydro-2*H*-pyran-3,4,5-triyl triacetate **1t** (197.2 mg, 0.2 mmol), methyl 4-iodobenzoate **2ba** (78.3 mg, 0.3 mmol), Ni(acac)$_2$ (10 mol %), Neocuproin **L6** (10 mol %), $LiClO_4$ (63.6 mg, 06 mmol), DIPEA (0.6 mmol), Purification by column chromatography on silica gel (*n*hexane/EtOAc: 10/1 to 5/1) yielded **53** (102.1 mg, 49%) as a syrup.

^{1}H NMR (400 MHz, $CDCl_3$): δ = 8.06 (d, J = 8.4 Hz, 2H), 7.50 (d, J = 8.2 Hz, 2H), 5.41 – 5.36 (m, 2H), 5.35 – 5.29 (m, 3H), 5.23 (d, J = 3.9 Hz, 1H), 5.11 (dd, J = 4.6, 2.6 Hz, 1H), 5.06 (t, J = 9.9 Hz, 1H), 4.86 (dd, J = 10.5, 4.0 Hz, 1H), 4.80 (dd, J = 10.3, 3.8 Hz, 1H), 4.49 (dd, J = 12.2, 2.4 Hz, 1H), 4.43 – 4.35 (m, 2H), 4.31 – 4.16 (m, 3H), 4.12 (ddd, J = 9.9, 4.4, 2.4 Hz, 1H), 4.05 (dd, J = 12.5, 2.4 Hz, 1H), 4.00 – 3.93 (m, 2H), 3.92 (s, 3H), 3.69 (dd, J = 7.1, 2.6 Hz, 1H), 2.17 (s, 3H), 2.14 (s, 3H), 2.14 (s, 3H), 2.09 (s, 3H), 2.03 (s, 3H), 2.01 (s, 3H), 1.99 (s, 3H), 1.98 (s, 3H), 1.96 (s, 3H), 1.95 (s, 3H).

^{13}C NMR (101 MHz, $CDCl_3$): δ = 170.57 (C_q), 170.49 (C_q), 170.43 (C_q), 170.25 (C_q), 169.96 (C_q), 169.85 (C_q), 169.71 (C_q), 169.56 (C_q), 169.40 (C_q), 166.74 (C_q), 142.30 (C_q), 129.74 (CH), 129.57 (C_q), 126.00 (CH), 96.81 (CH), 95.89 (CH), 75.08 (CH),

72.90 (CH), 72.21 (CH), 72.05 (CH), 71.83 (CH), 70.33 (CH), 69.99 (CH), 69.91 (CH), 69.35 (CH), 68.95 (CH), 68.73 (CH), 68.53 (CH), 67.85 (CH), 63.11 (CH_2), 62.55 (CH_2), 61.36 (CH_2), 52.12 (CH_3), 20.92 (CH), 20.85 (CH), 20.84 (CH), 20.74 (CH), 20.65 (CH), 20.57 (CH), 20.56 (CH), 20.50 (CH), 20.45 (CH), 20.41 (CH).

IR (ATR): $\tilde{v}$ = 1737, 1367, 1281, 1237, 1208, 1022, 917, 732, 602 cm^{-1} .
MS (ESI): m/z (relative intensity) 1065 (100) $[M+Na]^+$.
HR-MS (ESI): *m/z* calcd for $C_{46}H_{58}NaO_{27}^+$ $[M+Na]^+$: 1065.3058, found: 1065.3042.

Mechanistic Studies

The reaction with zinc powers.

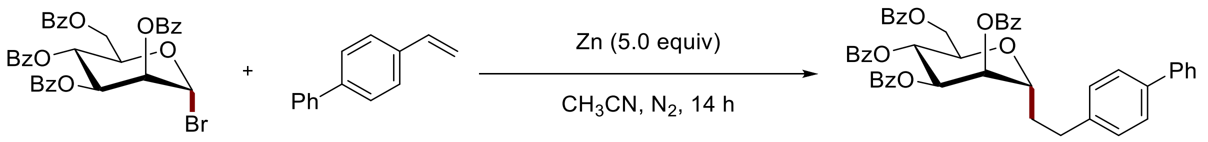

1g, 0.2 mmol **2aa,** 0.3 mmol 25 °C: **21a**, traces

80 °C: **21a**, traces

The reaction with stochiometric amounts of zinc powder at different temperatures failed to give a Giese-addition product, which may be attributed to the 2e⁻ reduction.

The reaction with D_2O.

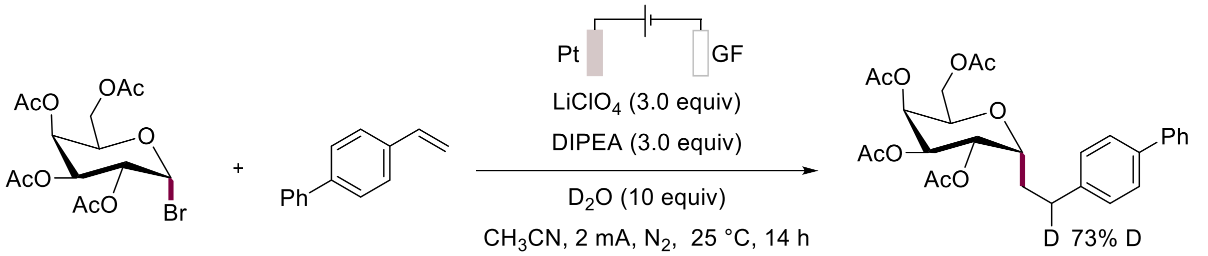

1a, 0.2 mmol **2aa,** 0.3 mmol **[D]-3a**, 57%

The deuterium labeling reaction with D2O suggests a benzylic radical reduction to anion is involved since 73%D is incorporated at the benzylic position.

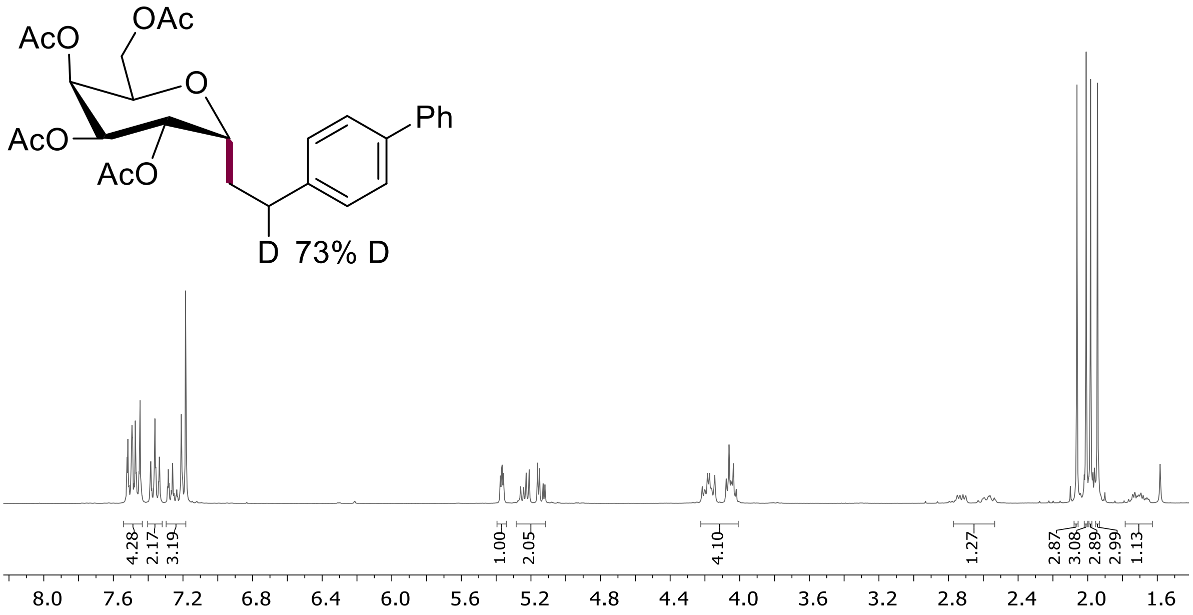

Capture of α-aminoalkyl radical with paired electrocatalysis

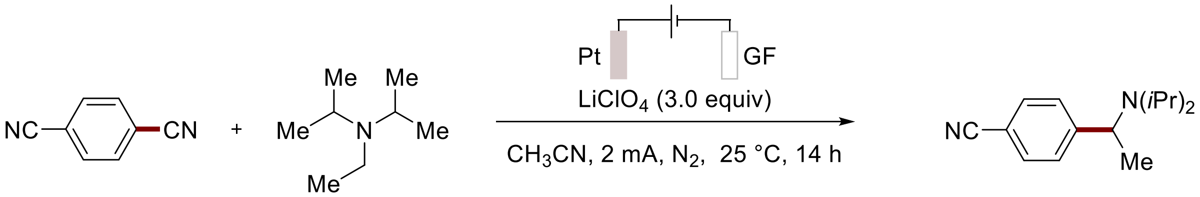

2bi, 0.3 mmol 0.6 mmol **54,** 58%

To capture the α-aminoalkyl radical, the paired electrolysis was conducted with dicyanobenzen*e* **2bi**, and the desired product **54** was isolated in 58% yield, which supports the electrochemical XAT process.

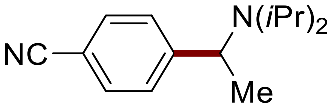

4-(1-(diisopropylamino)ethyl)benzonitrile (54)

^{1}H NMR (300 MHz, $CDCl_3$): δ = 7.56 (s, 4H), 4.13 (q, J = 6.8 Hz, 1H), 3.06 (hept, J = 6.7 Hz, 2H), 1.46 (d, J = 6.8 Hz, 3H), 1.10 (d, J = 6.6 Hz, 6H), 0.99 (d, J = 6.7 Hz, 6H).

^{13}C NMR (75 MHz, $CDCl_3$): δ = 153.47 (C_q), 131.56 (CH), 128.25 (CH), 119.30 (C_q), 109.50 (C_q), 52.01 (CH), 45.40 (CH), 23.33 (CH_3), 22.42 (CH_3), 19.17 (CH_3).

IR (ATR): $\tilde{v}$ = 2962, 2227, 1606, 1459, 1385, 1215, 1199, 1016, 834, 545 cm^{-1}.

MS (ESI): m/z (relative intensity) 231 (100) $[M+H]^+$, 253 (40) $[M+Na]^+$.

HR-MS (ESI): *m/z* calcd for $C_{15}H_{23}N_2^+$ $[M+H]^+$: 231.1856, found: 231.1858.

Paired electrolysis with 1,4-dicyanobenzene

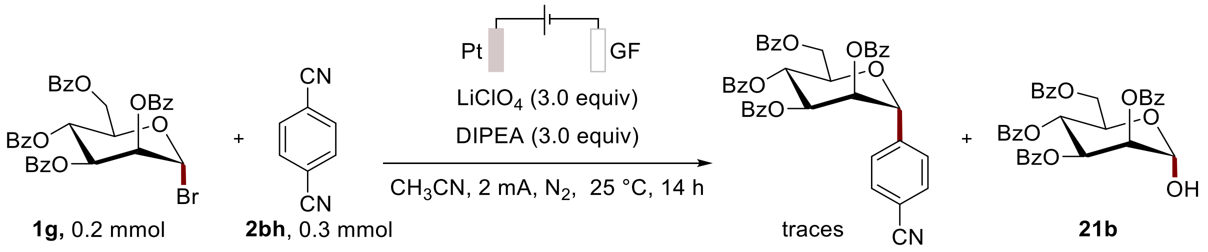

When mannosyl bromide **1g** is reacted dicynobenzene **2bh**, the paired electrolysis failed to form glycosyl arylation product. Instead, the anomeric hydroxylation **21b** byproduct was observed.

Reaction without DIPEA

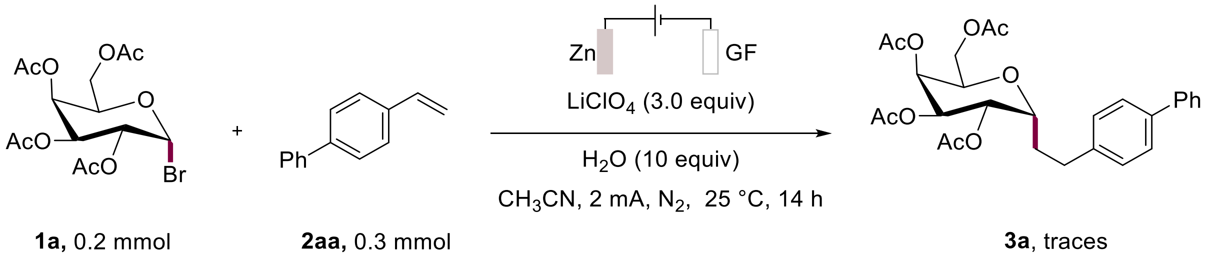

The control reaction without DIPEA as sacrificial amine, but with a Zn plate as anode failed to give the Giese addition product. It indicates the essential role of DIPEA.

Key intermediate preparation

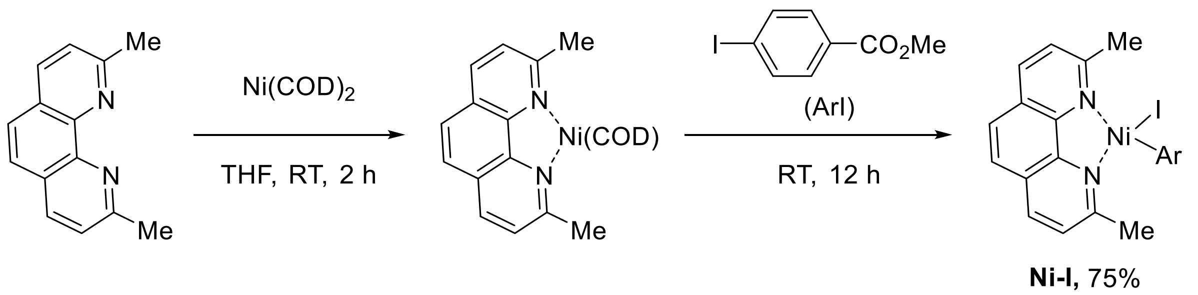

According to modified literature report of synthesizing Ni complex,[3] in a glove box, a 50 mL round bottom flask containing a stirring bar was charged with $Ni(COD)_2$ (1.0

mmol, 1.0 equiv), 2,9-dimethyl-10-phenanthroline (1.0 mmol, 1.0 equiv), and dry THF (10 mL) giving a dark blue mixture which was then stirred for 2 h at room temperature. Next, methyl 4-iodobenzoate (10.0 mmol, 10.0 equiv) was added and stirred for 12 h. Dry pentane was added to the mixture and filtered. The resulting precipitate was washed with pentane and dried under a vacuum to give solid **Ni-I** in 75% yield.

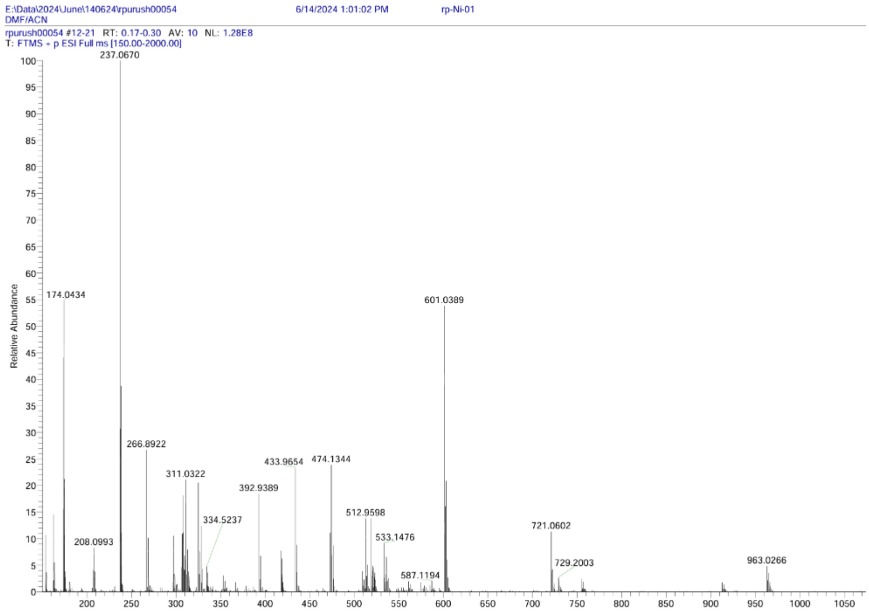

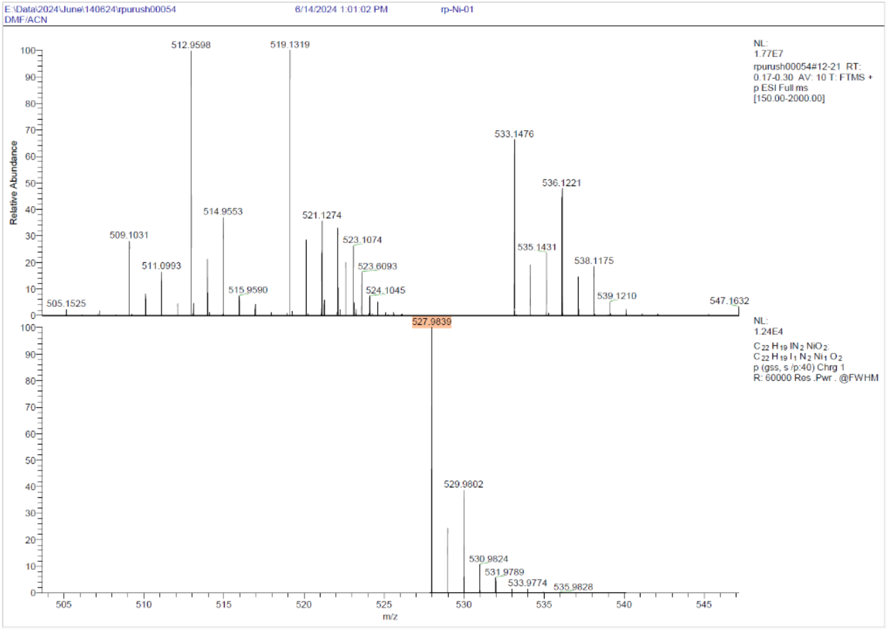

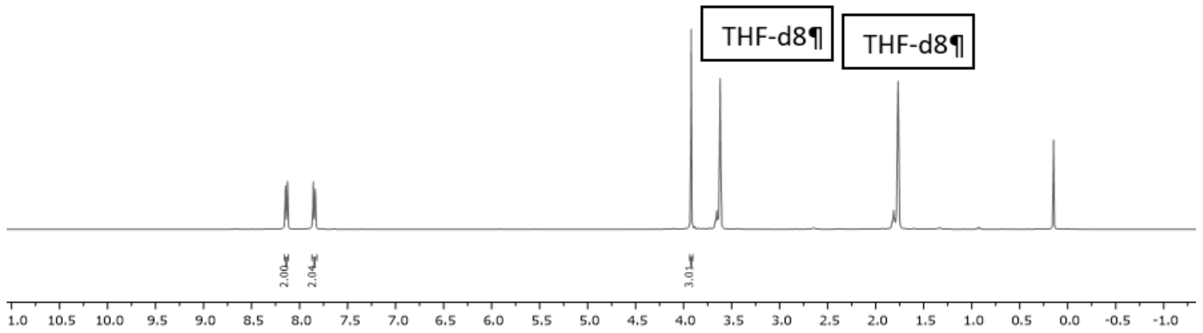

Ni-I complex examination

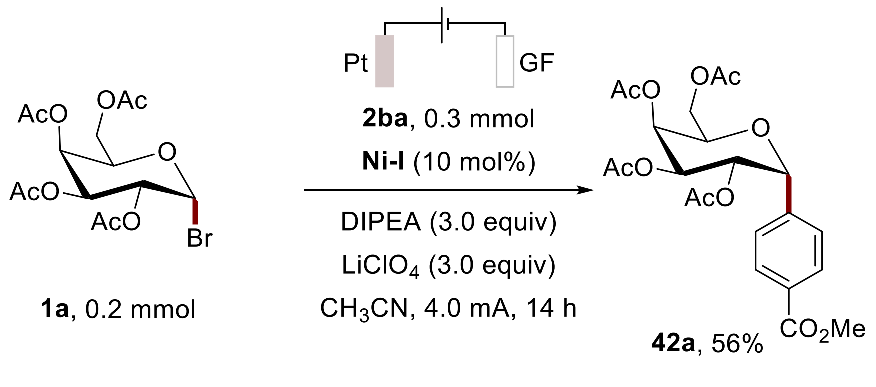

The catalyst **Ni-I** was used as a catalyst for standard electro-catalysed cross-electrophile coupling, releasing the product in 56% yield. It was suggesting the **Ni-I** complex might be catalytically relevant.

e-XAT application to dehalogenative alkylation with *tert*-butyl 3-iodoazetidine-1-carboxylate 1u

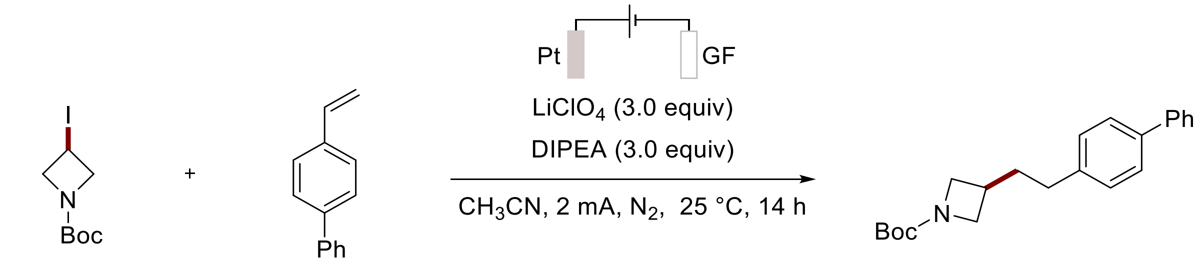

1u, 0.2 mmol **5**, 0.3 mmol **55,** 59%

The electrochemical *C*-glycosylation reaction condition was also feasible with *tert*-butyl 3-iodoazetidine-1-carboxylate**1u,** giving radical addition product **55** in 59% yield

***tert*-Butyl 3-{2-[(1,1'-biphenyl)-4-yl]ethyl}azetidine-1-carboxylate (55)**

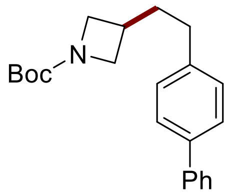

^{1}H NMR (400 MHz, $CDCl_3$): δ = 7.63 (dd, J = 8.3, 1.3 Hz, 2H), 7.59 – 7.55 (m, 2H), 7.51 – 7.45 (m, 2H), 7.41 – 7.35 (m, 1H), 7.27 (d, J = 8.2 Hz, 2H), 4.05 (t, J = 8.4 Hz, 2H), 3.62 (dd, J = 8.5, 5.5 Hz, 2H), 2.66 (dd, J = 8.5, 6.8 Hz, 2H), 2.62 – 2.52 (m, 1H), 1.99 (q, J = 7.7 Hz, 2H), 1.49 (s, 9H).

^{13}C NMR (101 MHz, $CDCl_3$): δ = 156.36 (C_q), 140.92 (C_q), 140.46 (C_q), 138.97 (C_q), 128.73 (CH), 128.70 (CH), 127.14 (CH), 127.05 (CH), 126.95 (CH), 79.17, 36.10 (CH_2), 32.93 (CH_2), 28.40 (CH_3), 28.37 (CH).

IR (ATR): ṽ = 1697, 1486, 1389, 1364, 1257, 1132, 827, 762, 79, 697, 564 cm^{-1} .

MS (ESI): m/z (relative intensity) 360 (90) $[M+Na]^+$, 697 (100) [2M+Na], [3M+Na], 1034 (45).

HR-MS (ESI): *m/z* calcd for $C_{22}H_{27}NNaO_2^+$ $[M+Na]^+$: 360.1934, found: 360.1937.

The application of e-XAT-mediated nickel catalysis to dehalogenative arylation with *tert*-butyl 3-iodoazetidine-1-carboxylate 1u

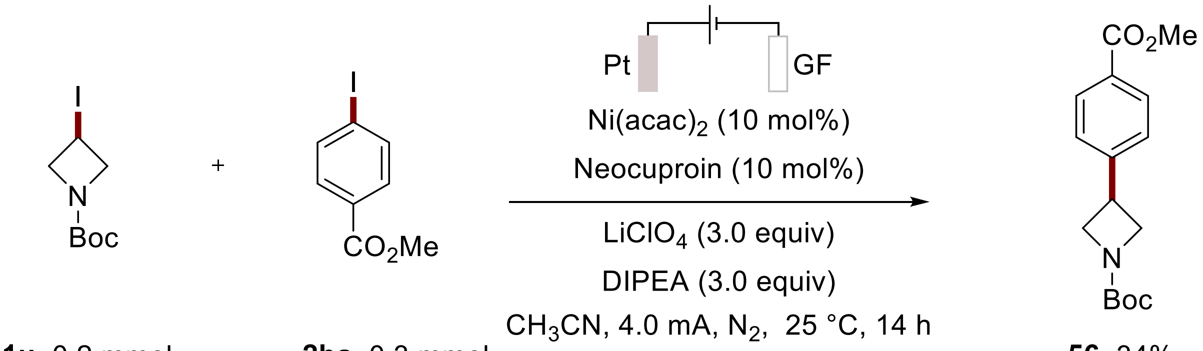

1u, 0.2 mmol **2ba**, 0.3 mmol **56,** 34%

The paired e-XAT and nickel catalysis was applied to simple alkyl halide **1u** *tert*-butyl 3-iodoazetidine-1-carboxylate, and the desired dehalogenative arylation product **56** was isolated in 34% yield.

***tert*-Butyl 3-[4-(methoxycarbonyl)phenyl]azetidine-1-carboxylate 56**

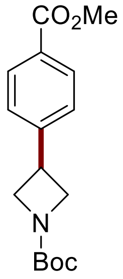

^{1}H NMR (300 MHz, $CDCl_3$): δ = 8.02 (d, J = 8.4 Hz, 2H), 7.38 (d, J = 8.0 Hz, 2H), 4.35 (t, J = 8.7 Hz, 2H), 4.03 – 3.94 (m, 2H), 3.91 (s, 3H), 3.88 – 3.71 (m, 1H), 1.47 (s, 9H).

^{13}C NMR (75 MHz, $CDCl_3$): δ = 166.81 (C_q), 156.34 (C_q), 147.47 (C_q), 130.06 (CH), 128.92 (C_q), 126.82 (CH), 79.74 (CH), 52.13 (CH_3), 33.48 (CH_2), 28.41 (CH_3).

IR (ATR): $\tilde{v}$ = 1265, 907, 728, 703, 650 cm^{-1} .

MS (ESI): m/z (relative intensity) 314 (80) $[M+Na]^+$, 605 (100) [2M+Na], [3M+Na], 896 (45).

HR-MS (ESI): *m/z* calcd for $C_{16}H_{21}NNaO_4^+$ $[M+Na]^+$: 314.1363, found: 314.1356.

Cyclic Voltammetry studies

CV measurements were conducted with a Metrohm Autolab PGSTAT204 potentiostat and Nova 2.1 software. A glassy carbon working electrode (disk, diameter: 3mm), a coiled platinum wire counter electrode, and a non-aqueous Ag-wire/ferrocene reference electrode were employed. The voltammograms were recorded at room temperature in CH_3CN, at a substrate concentration of 75umol and 0.1 mM $LiClO_4$ as supporting electrolytes. All solutions were degassed with N_2 before the measurement and an overpressure of protective gas was maintained throughout the experiment. The scan rate is 100 mV/s. The respective figures and descriptions indicate deviations from the general experimental conditions.

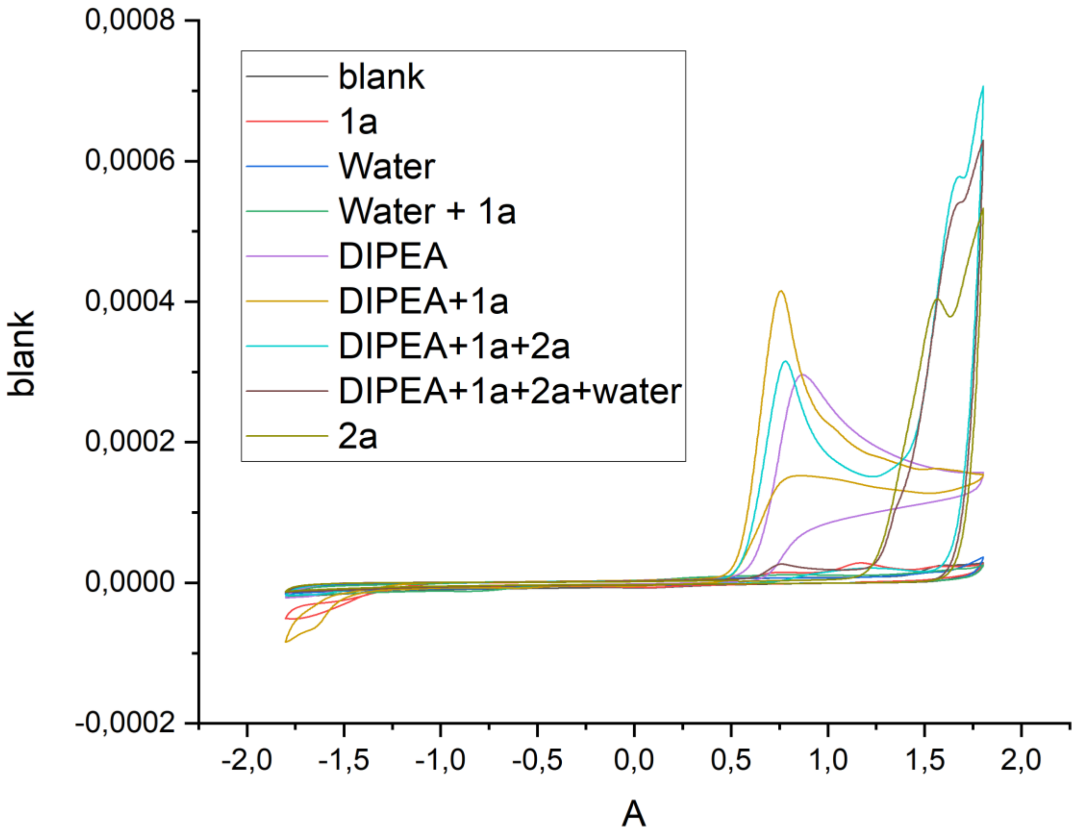

Figure S1: Cyclic voltammetry of the reactants in CH_3CN.

References

(1) (a) Doyle, L. M.; O'Sullivan, S.; Di Salvo, C.; McKinney, M.; McArdle, P.; Murphy, P. V. Stereoselective Epimerizations of Glycosyl Thiols. *Org. Lett.* **2017**, *19*, 5802-5805. (b) Wu, J.; Kopp, A.; Ackermann, L. Synthesis of C-Oligosaccharides through Versatile C(sp(3))-H Glycosylation of Glycosides. *Angew. Chem. Int. Ed.* **2022**, *61*, e202114993.
(2) Petracca, R.; Bowen, K. A.; McSweeney, L.; O'Flaherty, S.; Genna, V.; Twamley, B.; Devocelle, M.; Scanlan, E. M. Chemoselective Synthesis of N-Terminal Cysteinyl Thioesters via β,γ-C,S Thiol-Michael Addition. *Org. Lett.* **2019**, *21*, 3281-3285.
(3) (a) Shrestha, R.; Dorn, S. C. M.; Weix, D. J. Nickel-Catalyzed Reductive Conjugate Addition to Enones via Allylnickel Intermediates. *J. Am. Chem. Soc.* **2013**, *135*, 751-762. (b) Yang, G.; Wang, Y.; Qiu, Y. Electroreductive Cross-Electrophile Coupling of Aziridines and Aryl Bromides. *Chem. Eur. J.* **2023**, *29*, e202300959.

^{1}H- and ^{13}C-NMR Spectra

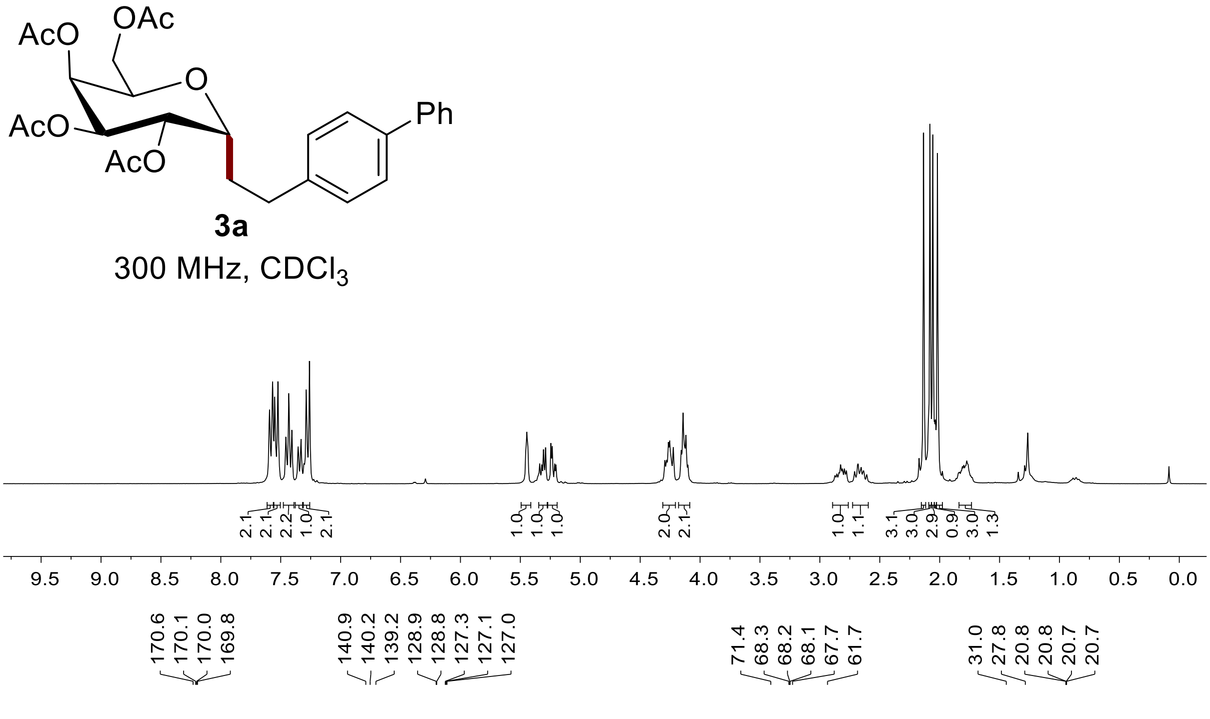

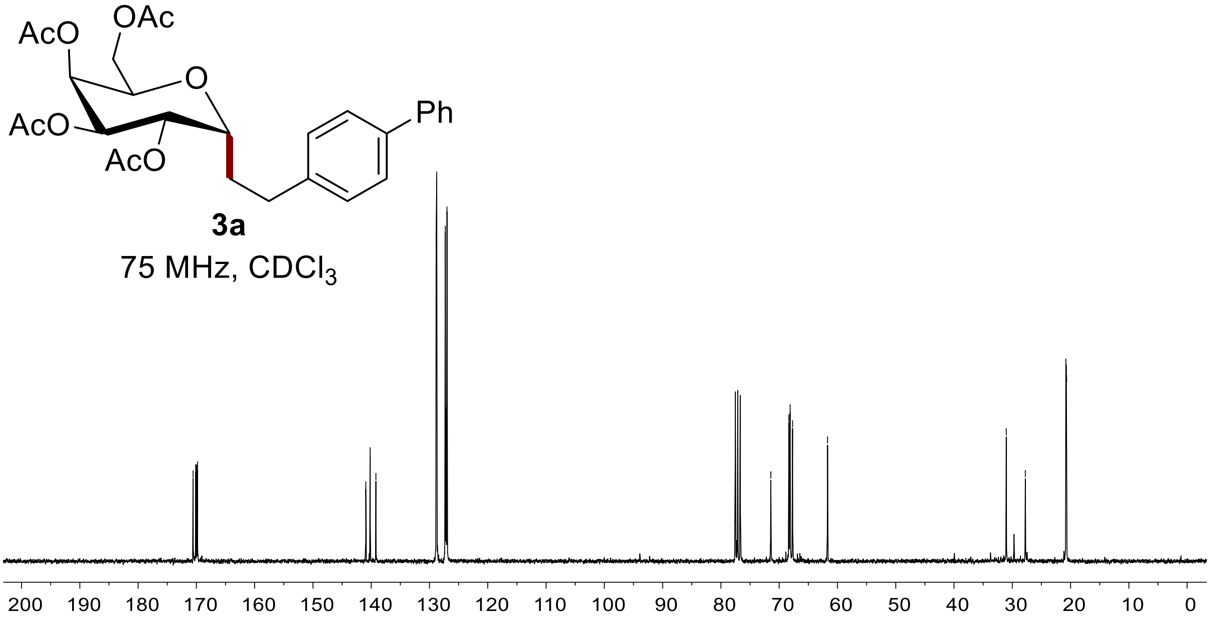

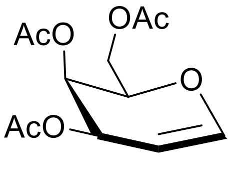

3b
(300 MHz, CDCl3)

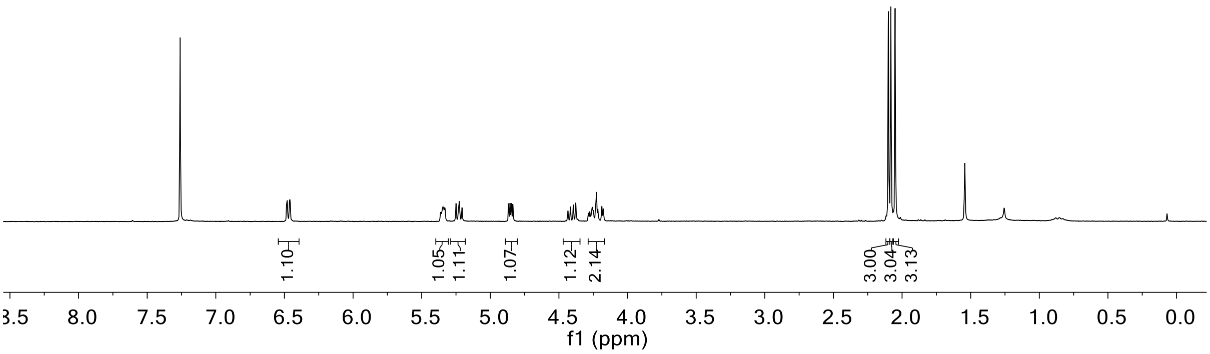

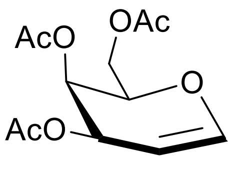

3b
(75 MHz, CDCl3)

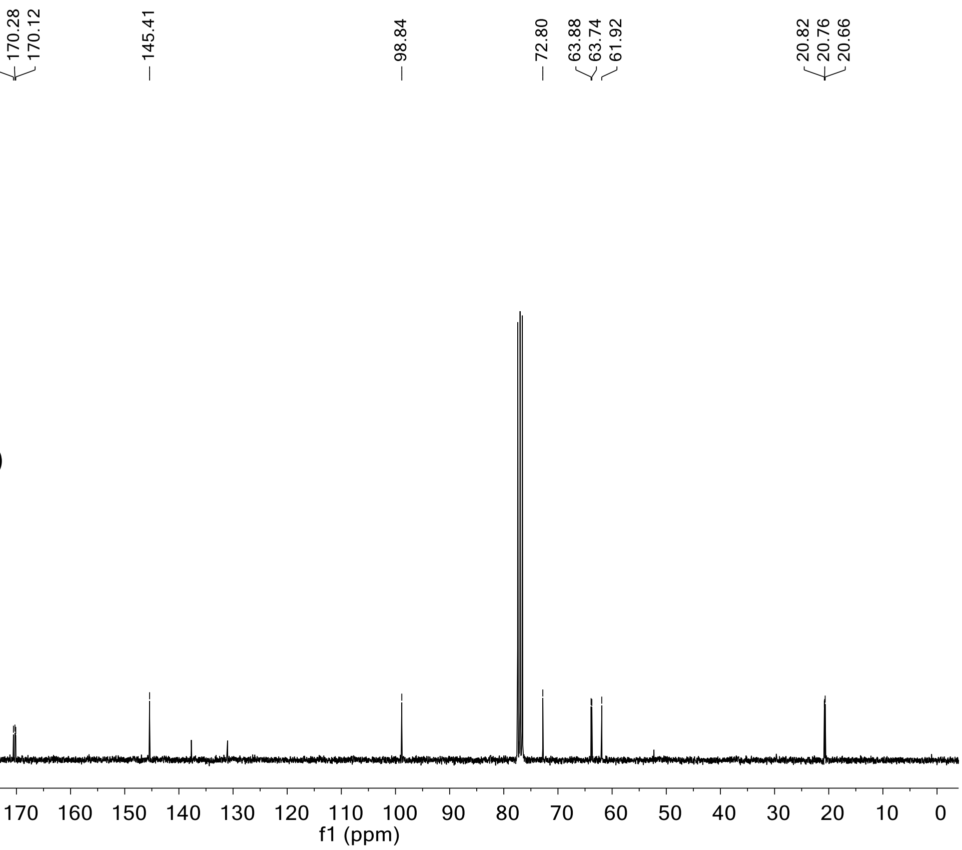

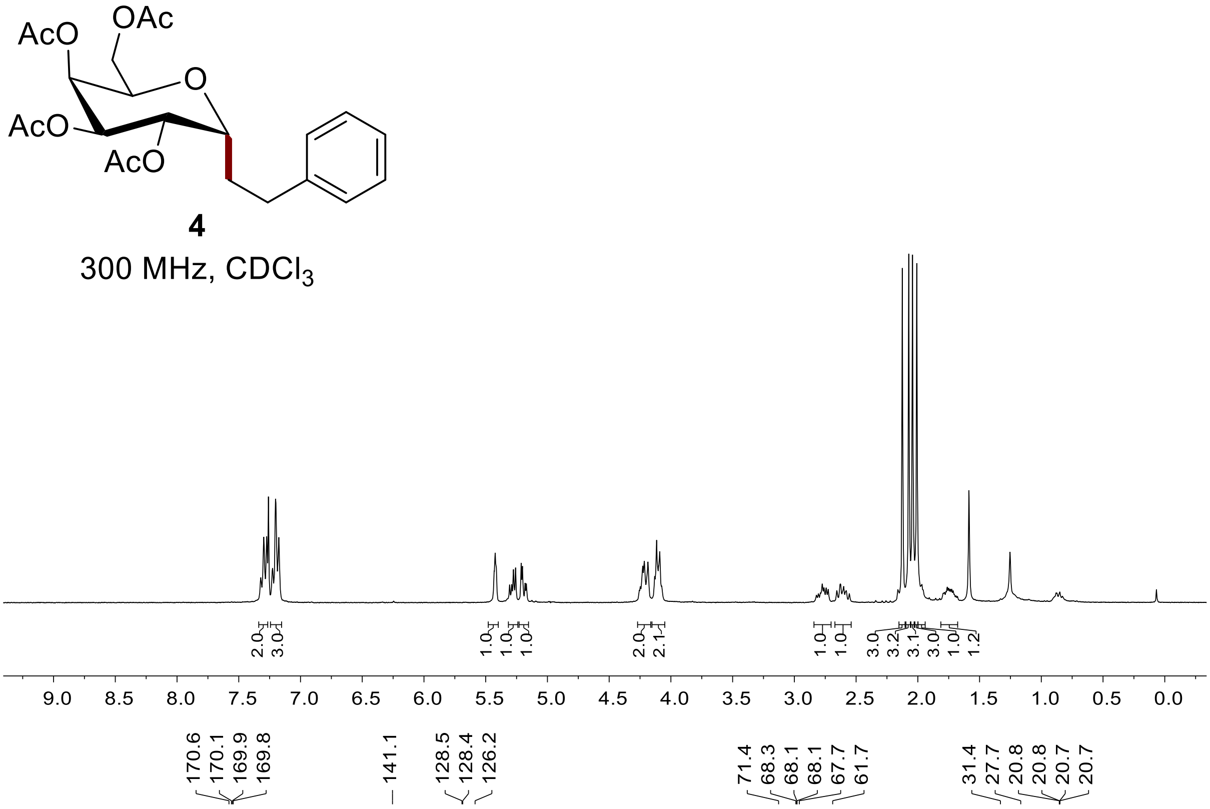
OAc
AcO
O
AcO
AcO
4
300 MHz, CDCl$_3$
2.0
3.0
1.0
1.0
1.0
2.0
2.1
1.0
1.0
3.0
3.2
3.1
3.0
1.0
1.2
9.0 8.5 8.0 7.5 7.0 6.5 6.0 5.5 5.0 4.5 4.0 3.5 3.0 2.5 2.0 1.5 1.0 0.5 0.0
170.6
170.1
169.9
169.8
141.1
128.5
128.4
126.2
71.4
68.3
68.1
68.1
67.7
61.7
31.4
27.7
20.8
20.8
20.7
20.7

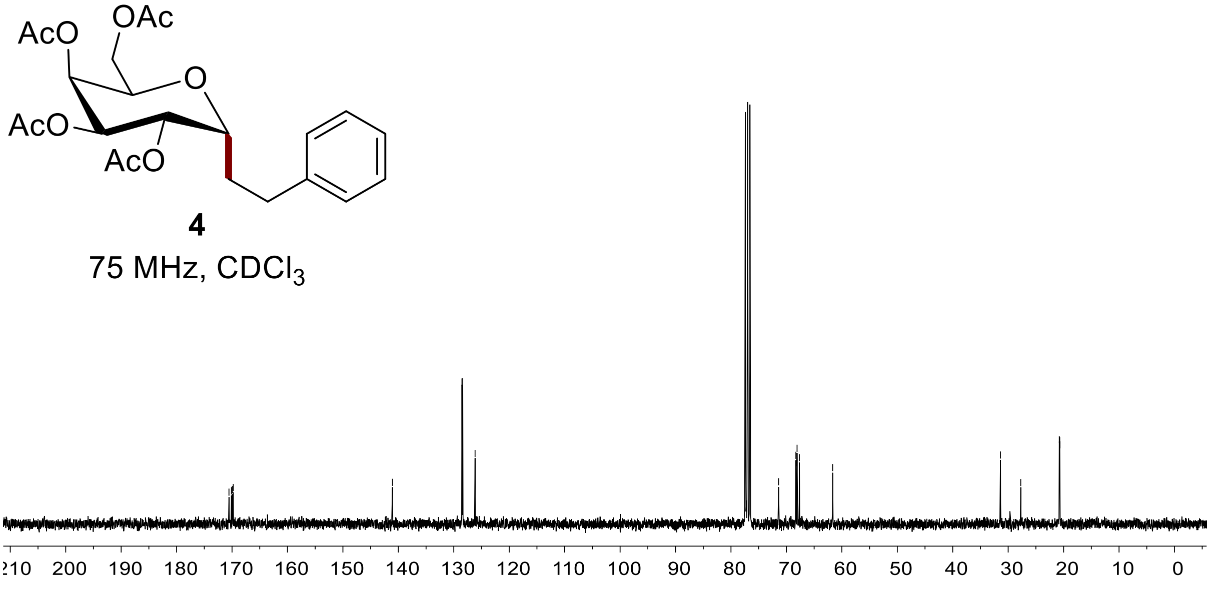
OAc
AcO
O
AcO
AcO
4
75 MHz, CDCl$_3$
210 200 190 180 170 160 150 140 130 120 110 100 90 80 70 60 50 40 30 20 10 0

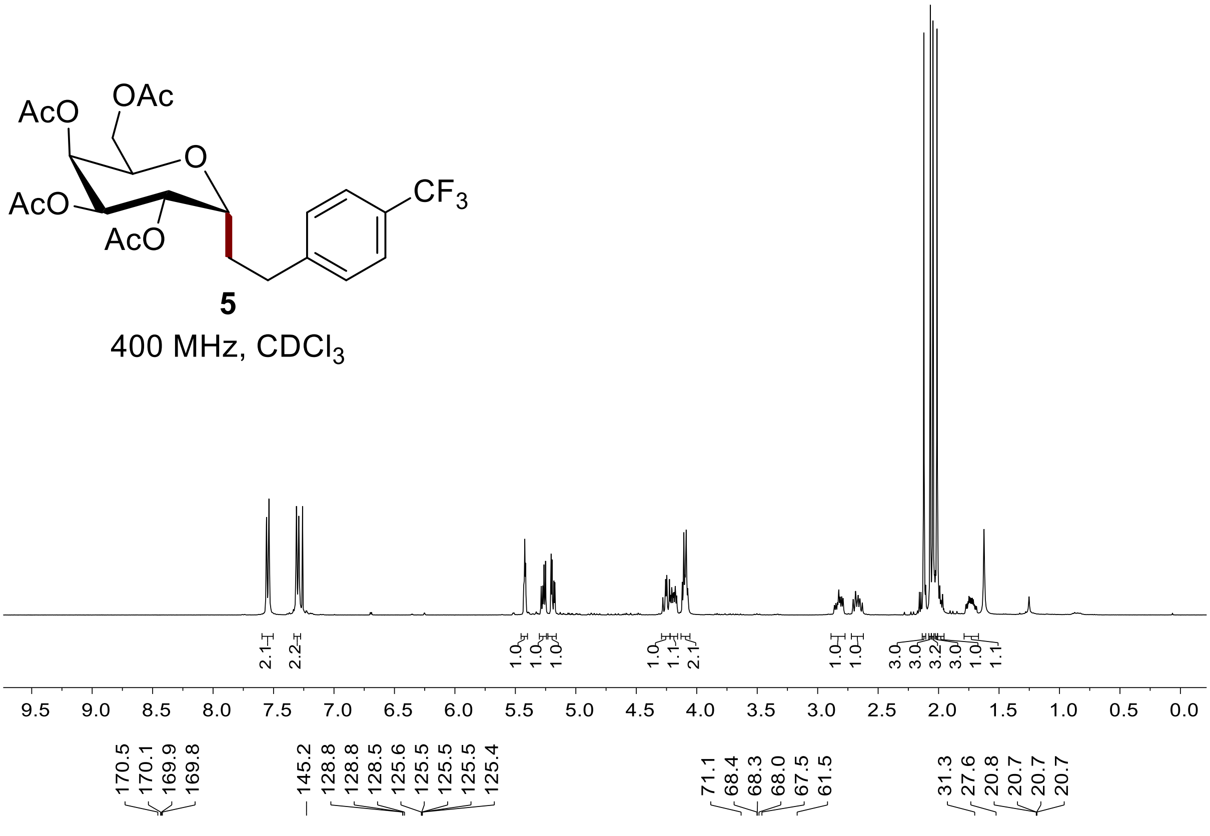

5

400 MHz, $CDCl_3$

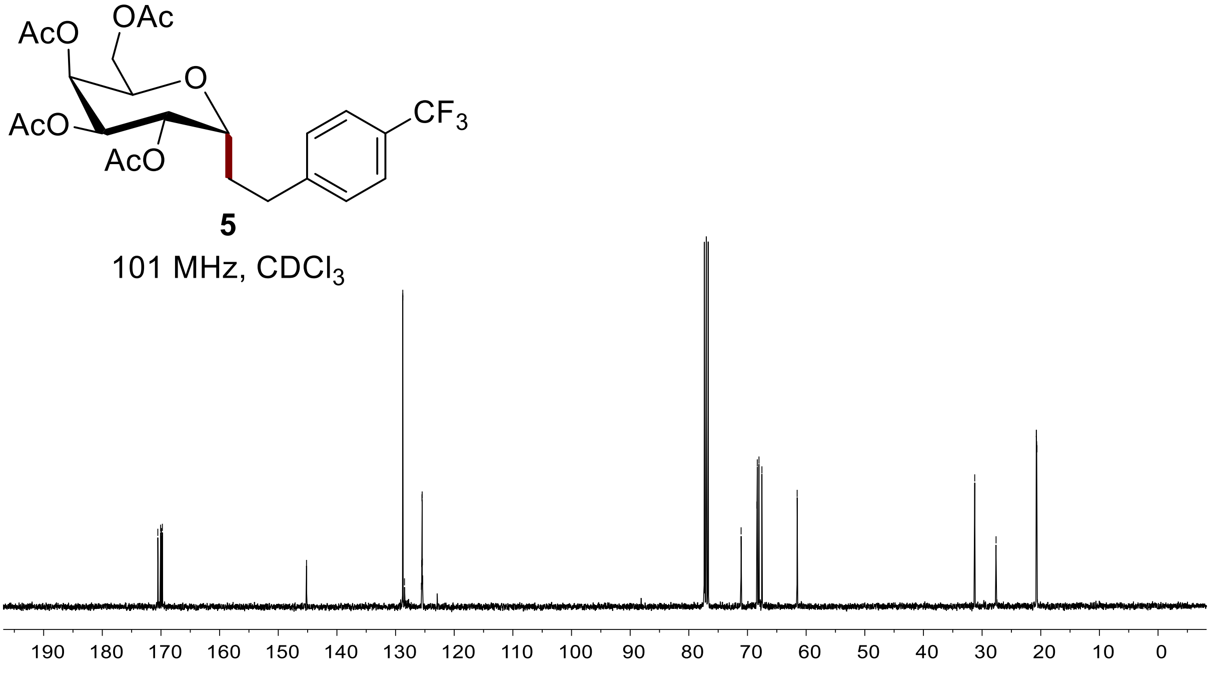

5

101 MHz, $CDCl_3$

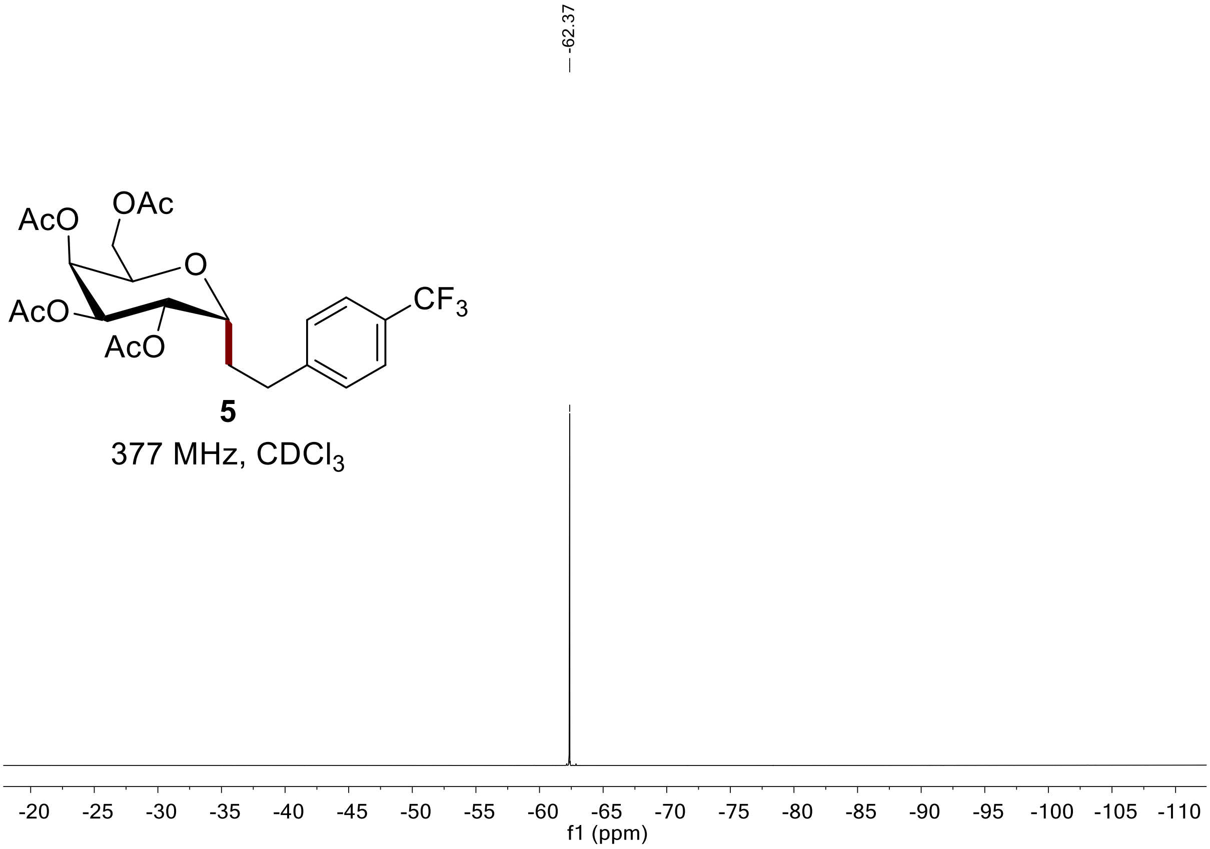

5

377 MHz, $CDCl_3$

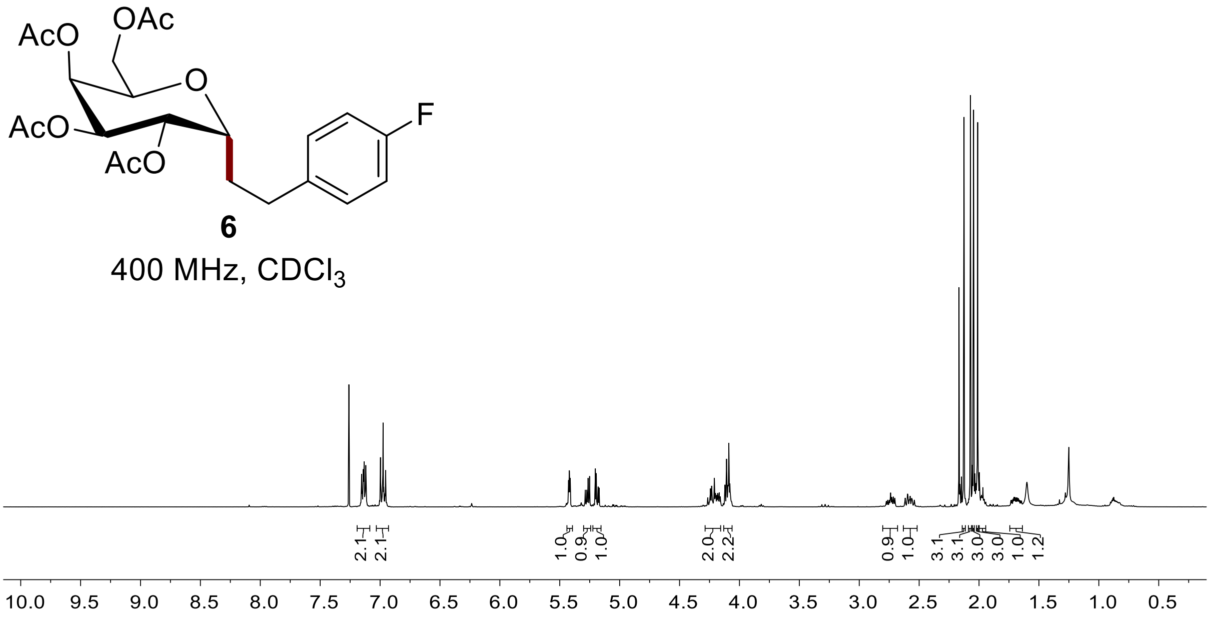

6

400 MHz, $CDCl_3$

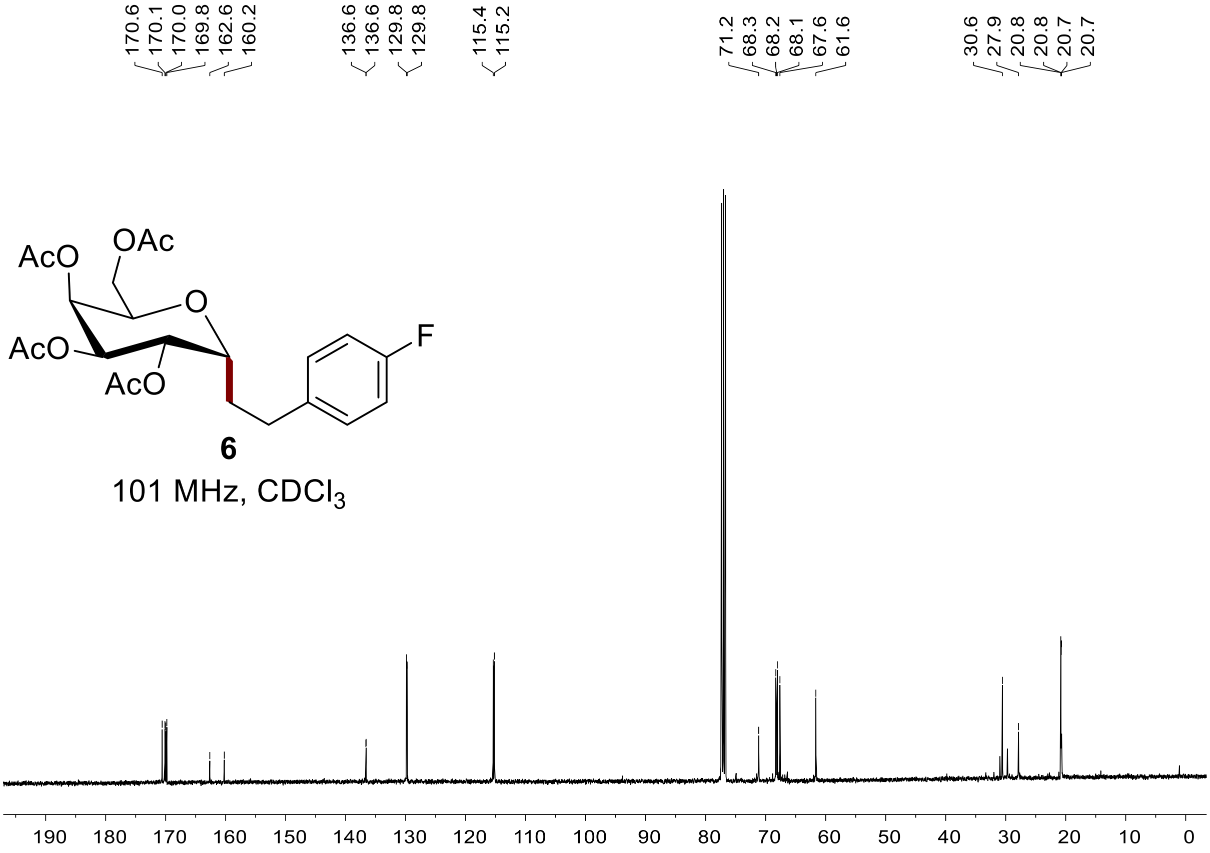
170.6
170.1
170.0
169.8
162.6
160.2
136.6
136.6
129.8
129.8
115.4
115.2
71.2
68.3
68.2
68.1
67.6
61.6
30.6
27.9
20.8
20.8
20.7
20.7
AcO
OAc
O
AcO
AcO
F
6
101 MHz, CDCl3
190 180 170 160 150 140 130 120 110 100 90 80 70 60 50 40 30 20 10 0

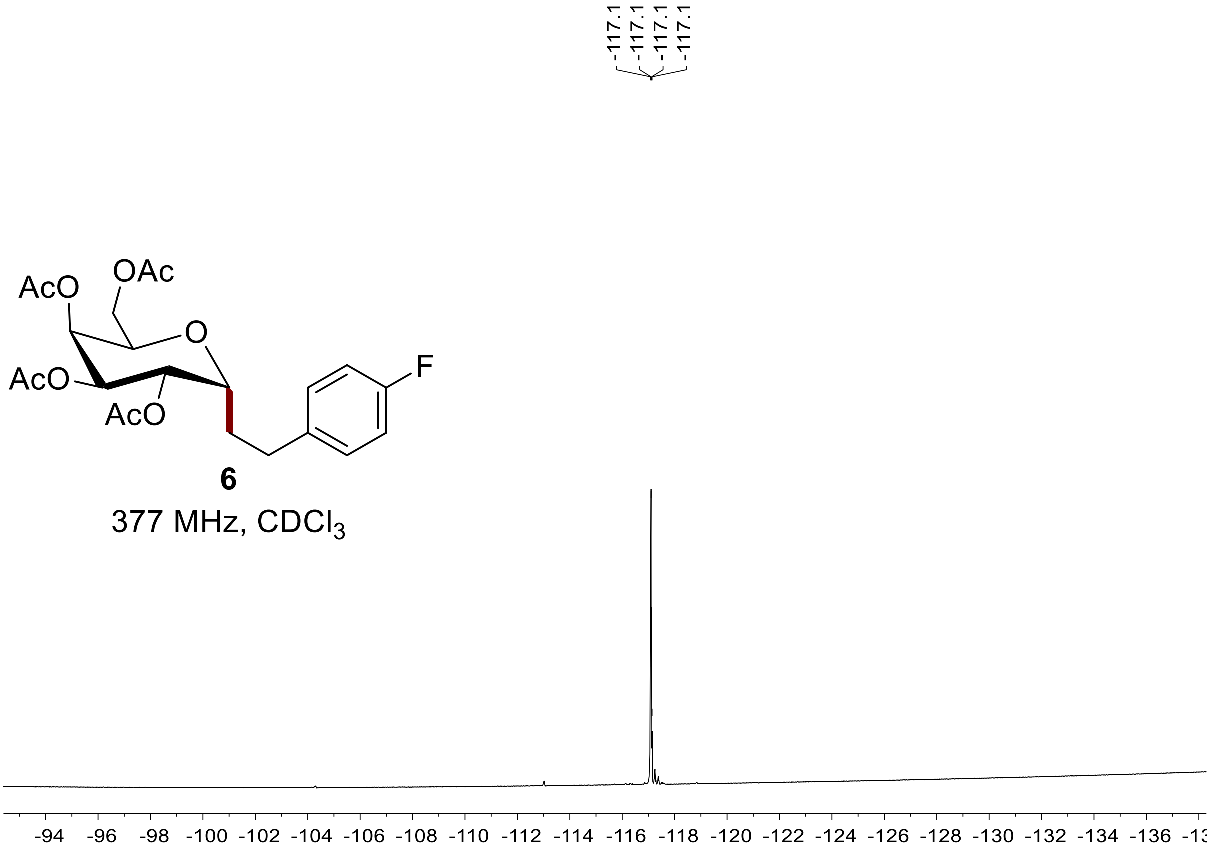
-117.1
-117.1
-117.1
-117.1
AcO
OAc
O
AcO
AcO
F
6
377 MHz, CDCl3
-94 -96 -98 -100 -102 -104 -106 -108 -110 -112 -114 -116 -118 -120 -122 -124 -126 -128 -130 -132 -134 -136

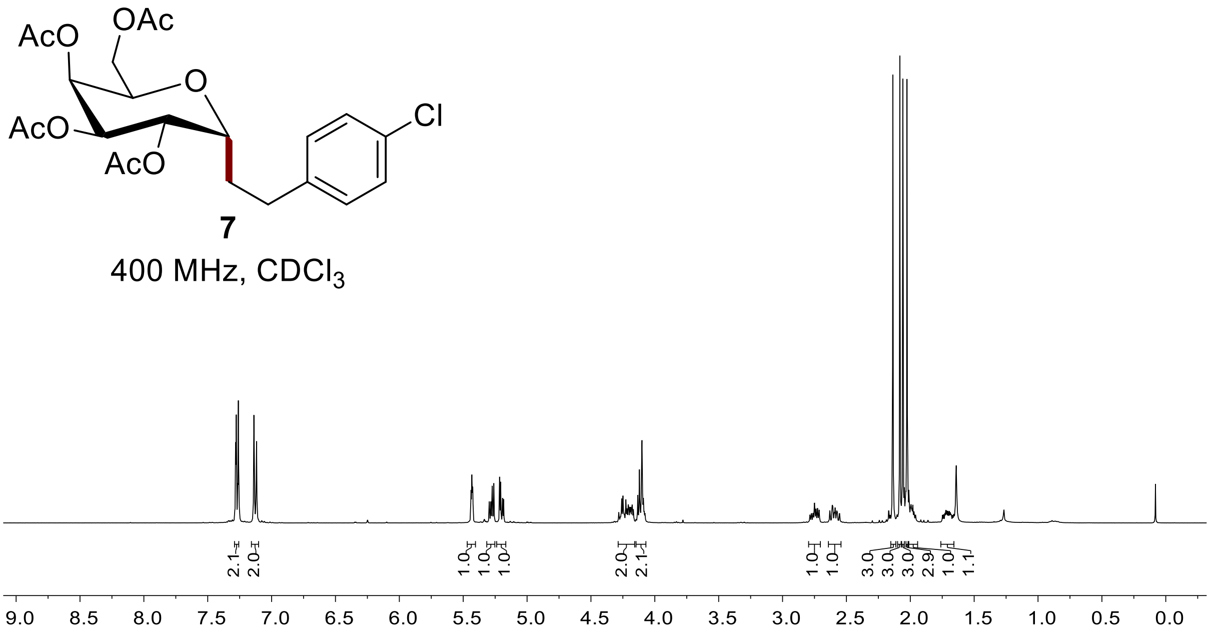
AcO
OAc
AcO
AcO
O
Cl
7
400 MHz, CDCl3
2.1
2.0
1.0
1.0
1.0
2.0
2.1
1.0
1.0
3.0
3.0
3.0
2.9
1.0
1.1
9.0 8.5 8.0 7.5 7.0 6.5 6.0 5.5 5.0 4.5 4.0 3.5 3.0 2.5 2.0 1.5 1.0 0.5 0.0

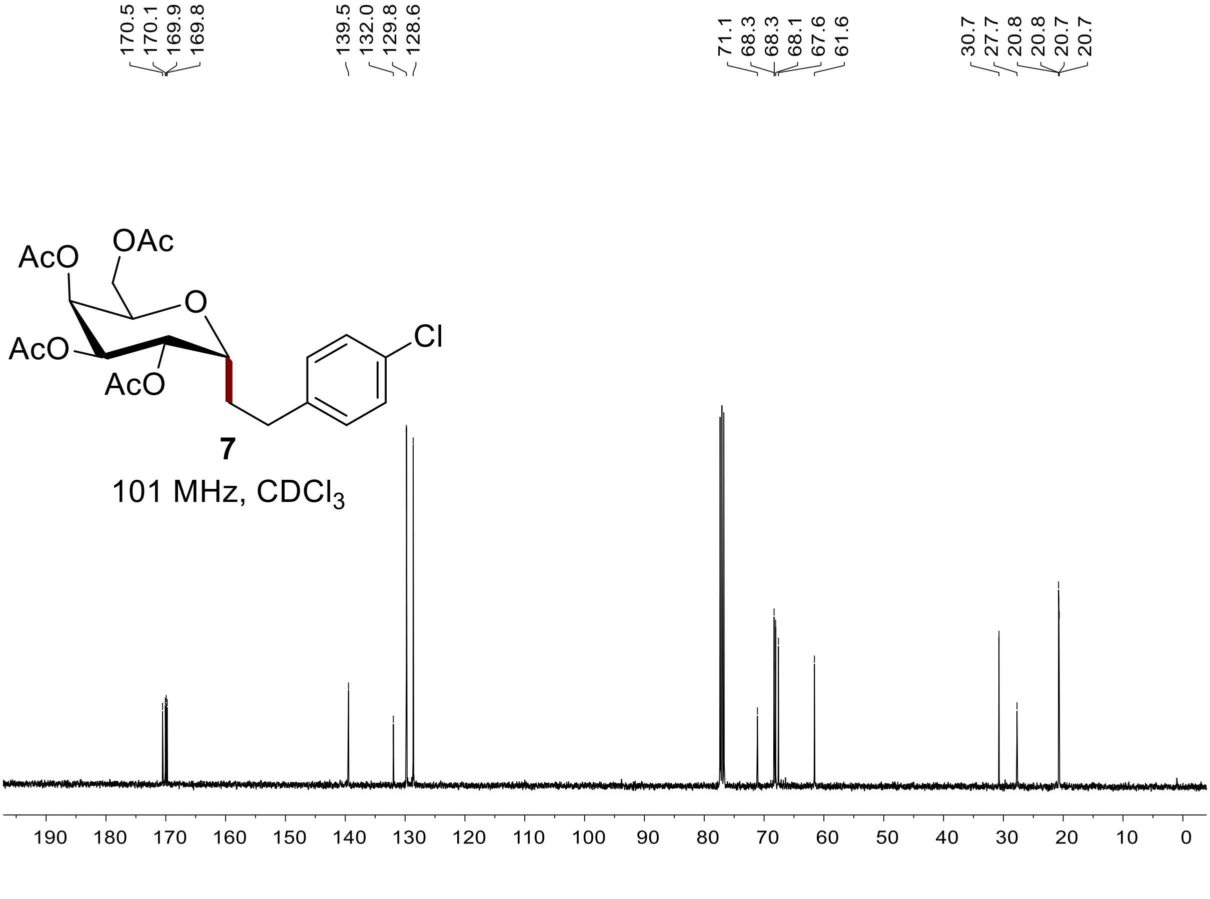
170.5
170.1
169.9
169.8
139.5
132.0
129.8
128.6
71.1
68.3
68.3
68.1
67.6
61.6
30.7
27.7
20.8
20.8
20.7
20.7
AcO
OAc
AcO
AcO
O
Cl
7
101 MHz, CDCl3
190 180 170 160 150 140 130 120 110 100 90 80 70 60 50 40 30 20 10 0

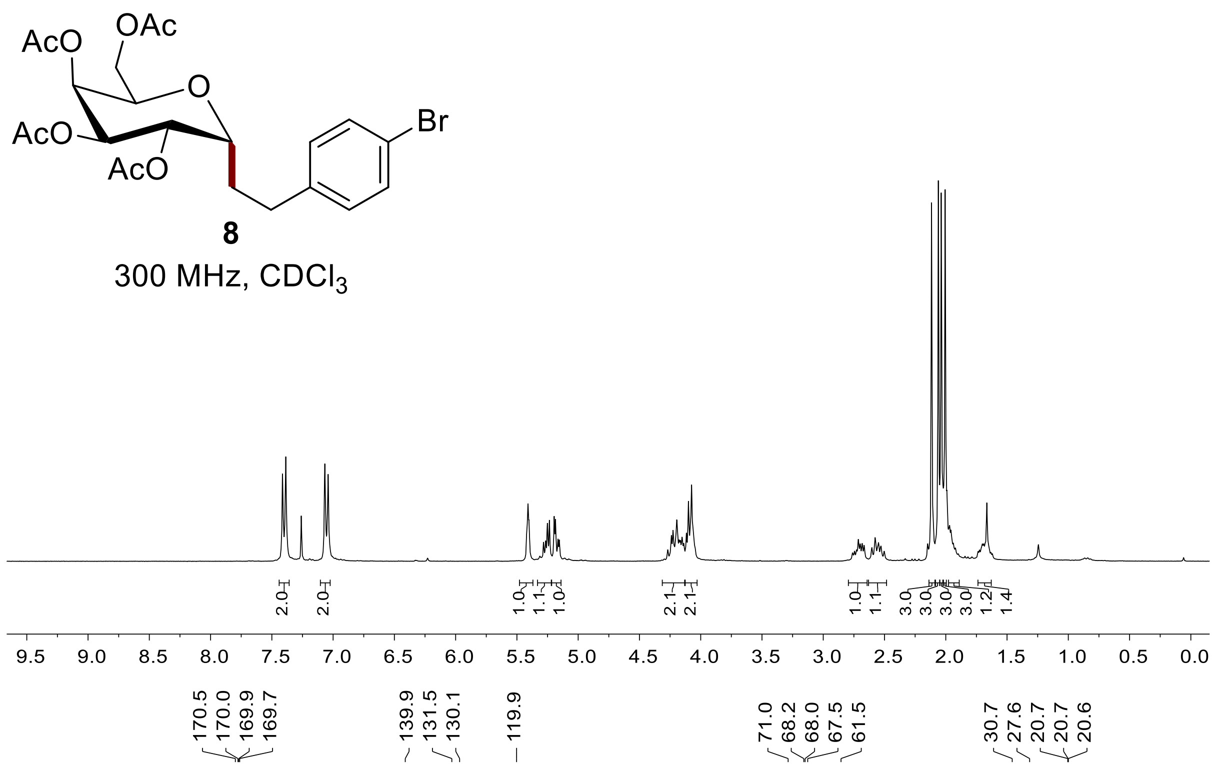

AcO
OAc
O
AcO
AcO
Br
8
300 MHz, CDCl3
2.0
2.0
1.0
1.1
1.0
2.1
2.1
1.0
1.1
3.0
3.0
3.0
3.0
1.2
1.4
9.5 9.0 8.5 8.0 7.5 7.0 6.5 6.0 5.5 5.0 4.5 4.0 3.5 3.0 2.5 2.0 1.5 1.0 0.5 0.0
170.5
170.0
169.9
169.7
139.9
131.5
130.1
119.9
71.0
68.2
68.0
67.5
61.5
30.7
27.6
20.7
20.7
20.6

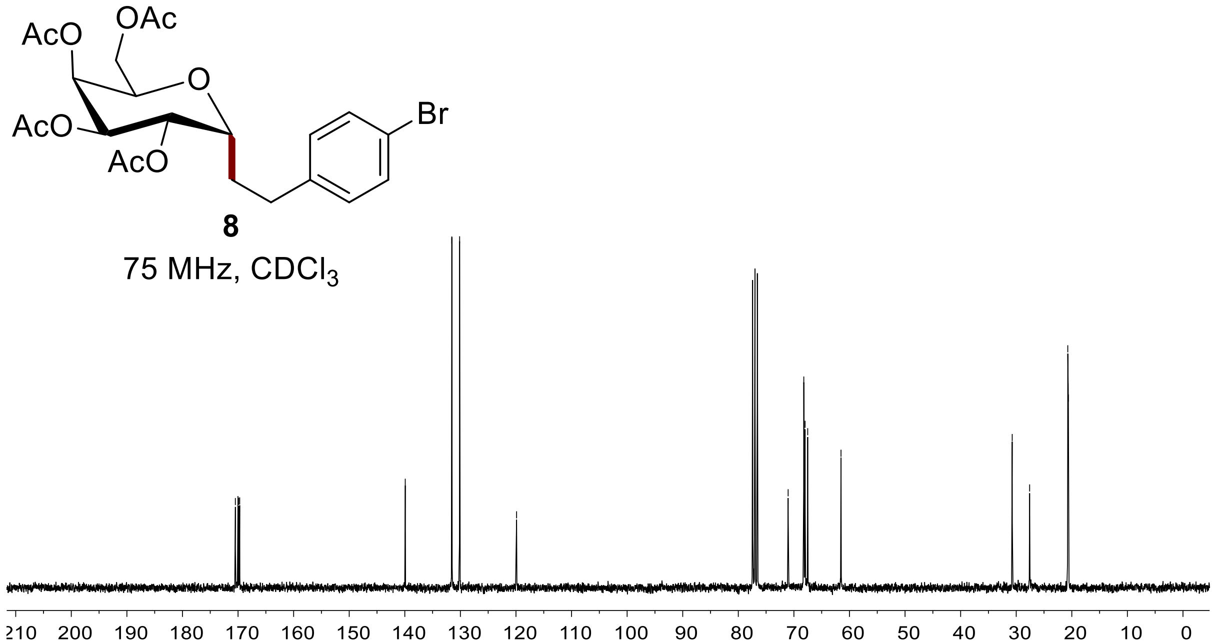

AcO
OAc
O
AcO
AcO
Br
8
75 MHz, CDCl3
210 200 190 180 170 160 150 140 130 120 110 100 90 80 70 60 50 40 30 20 10 0

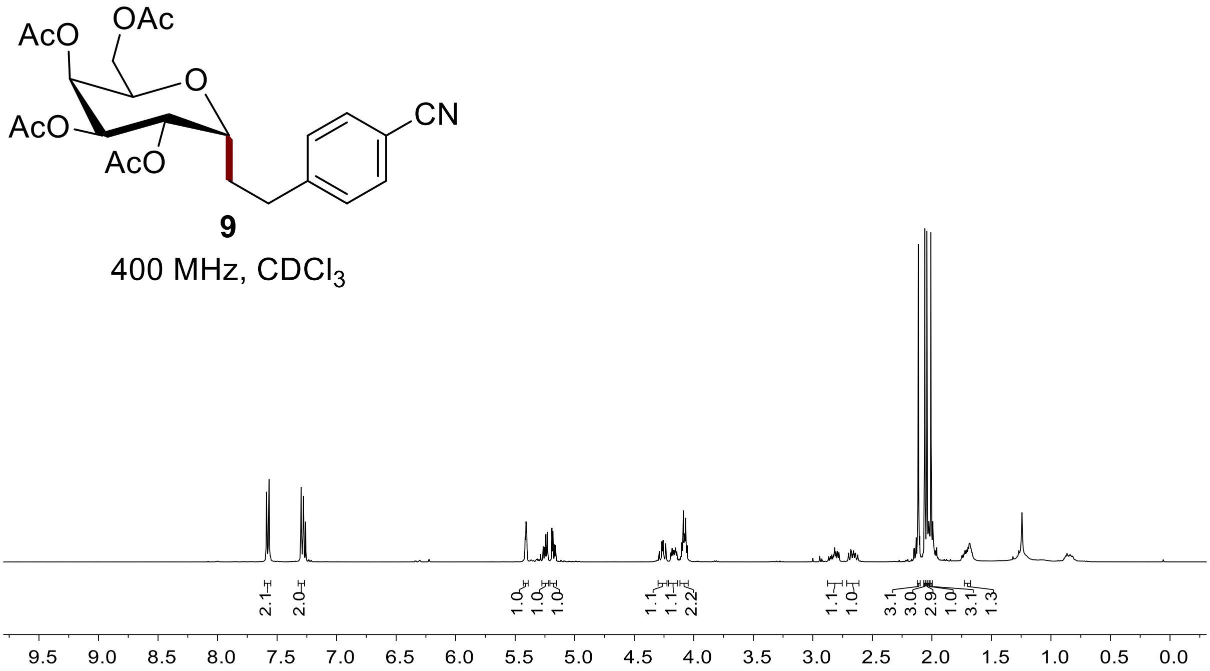

AcO
OAc
O
CN
AcO
AcO
9
400 MHz, CDCl3
2.1
2.0
1.0
1.0
1.0
1.1
1.1
2.2
1.1
1.0
3.1
3.0
2.9
1.0
3.1
1.3
9.5 9.0 8.5 8.0 7.5 7.0 6.5 6.0 5.5 5.0 4.5 4.0 3.5 3.0 2.5 2.0 1.5 1.0 0.5 0.0

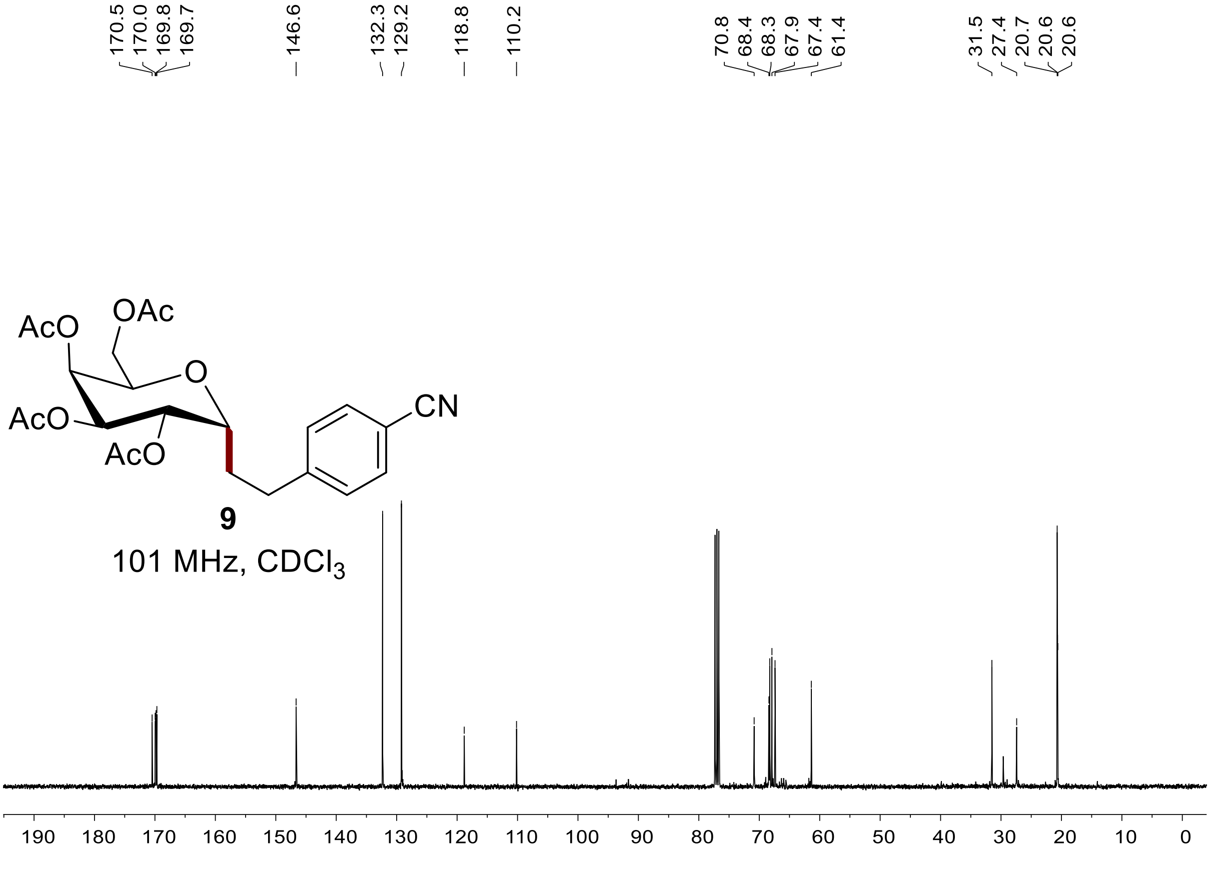

170.5
170.0
169.8
169.7
146.6
132.3
129.2
118.8
110.2
70.8
68.4
68.3
67.9
67.4
61.4
31.5
27.4
20.7
20.6
20.6
AcO
OAc
O
CN
AcO
AcO
9
101 MHz, CDCl3
190 180 170 160 150 140 130 120 110 100 90 80 70 60 50 40 30 20 10 0

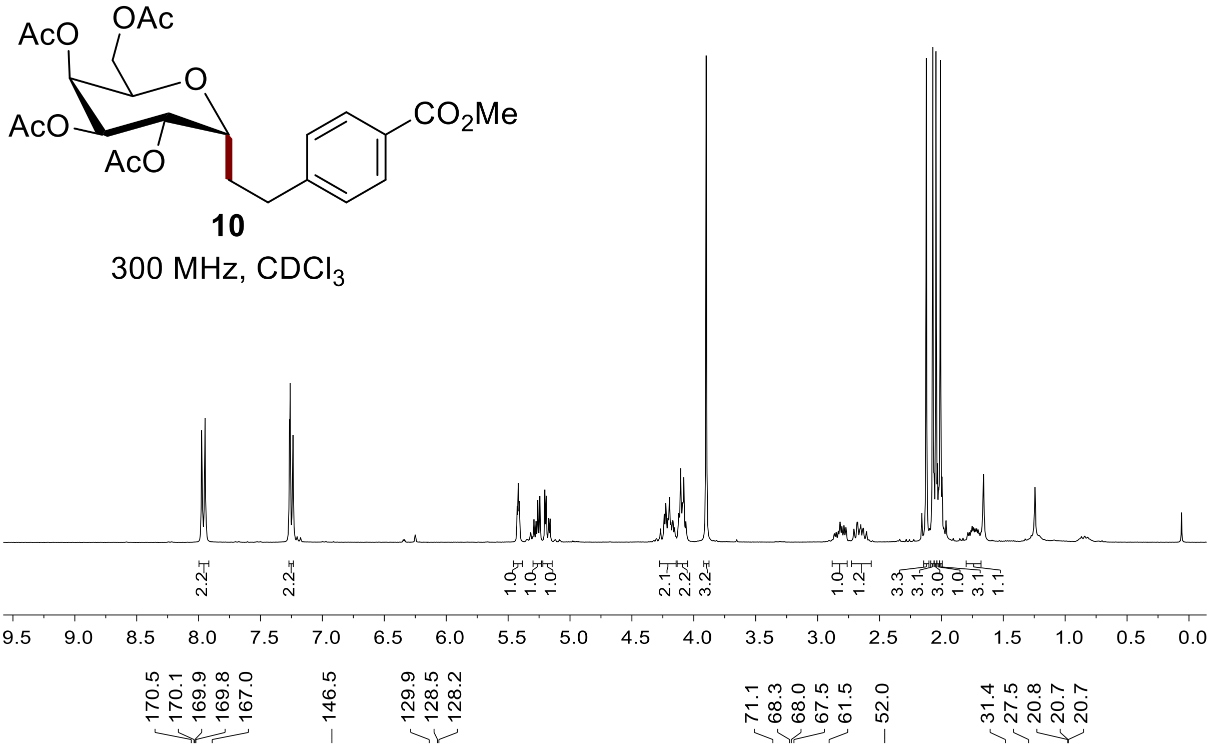

10

300 MHz, $CDCl_3$

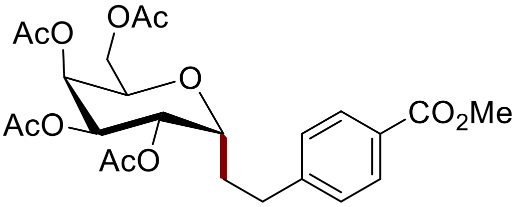

10

75 MHz, $CDCl_3$

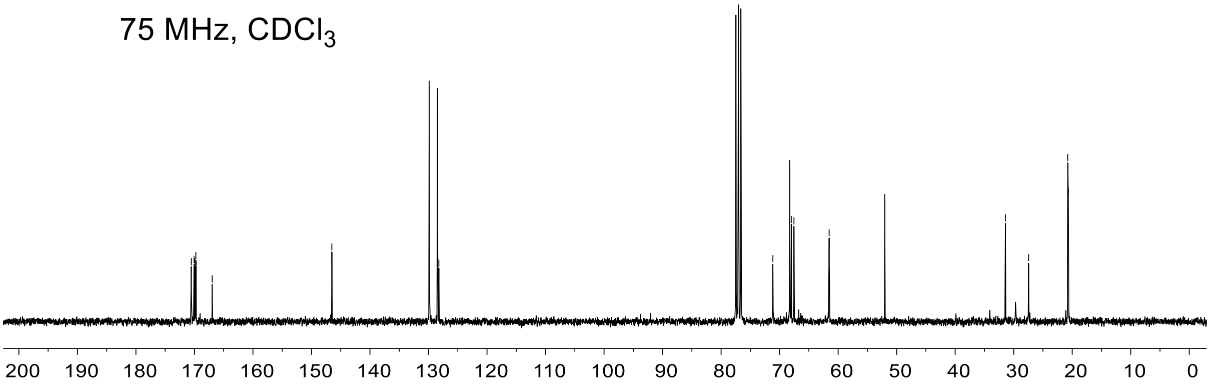

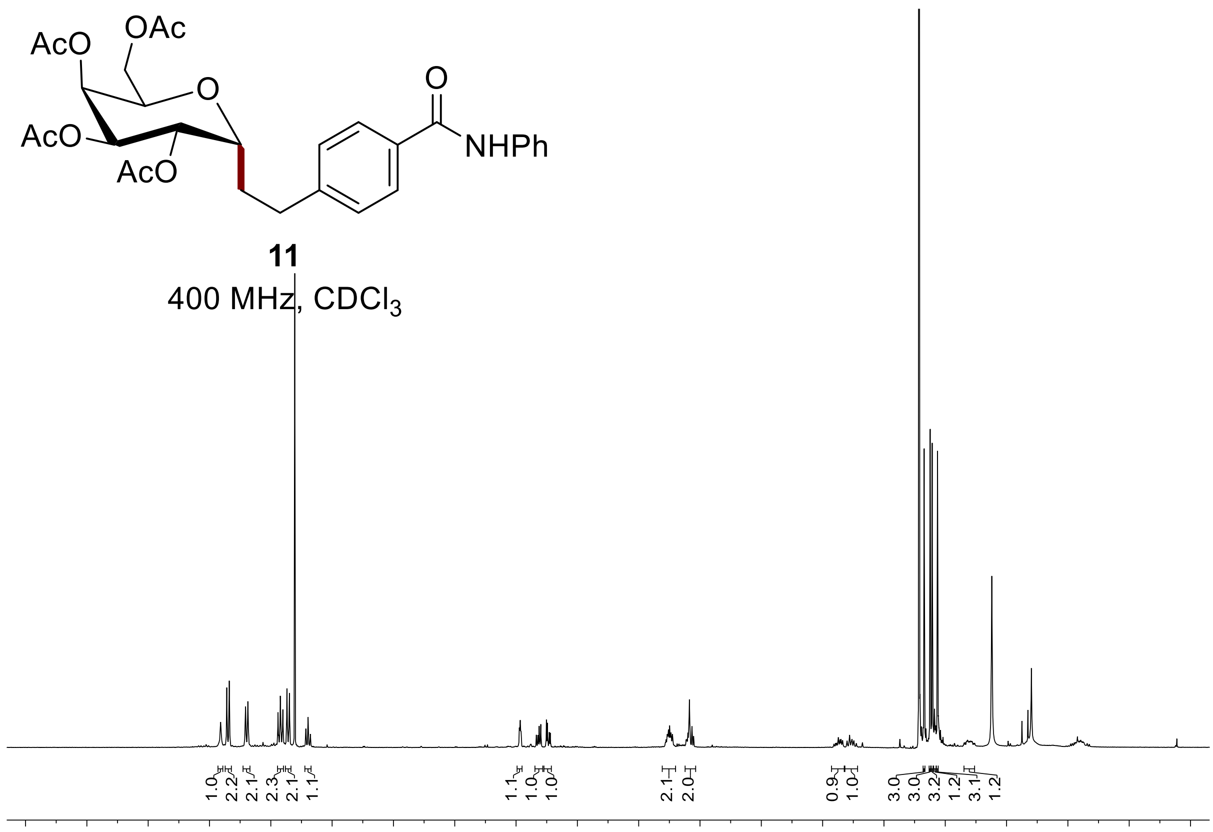
AcO
OAc
O
AcO
AcO
O
NHPh
11
400 MHz, CDCl$_3$
1.0 2.2 2.1 2.3 2.1 1.1 1.1 1.0 1.0 2.1 2.0 0.9 1.0 3.0 3.0 3.2 1.2 3.1 1.2
9.5 9.0 8.5 8.0 7.5 7.0 6.5 6.0 5.5 5.0 4.5 4.0 3.5 3.0 2.5 2.0 1.5 1.0 0.5 0.0

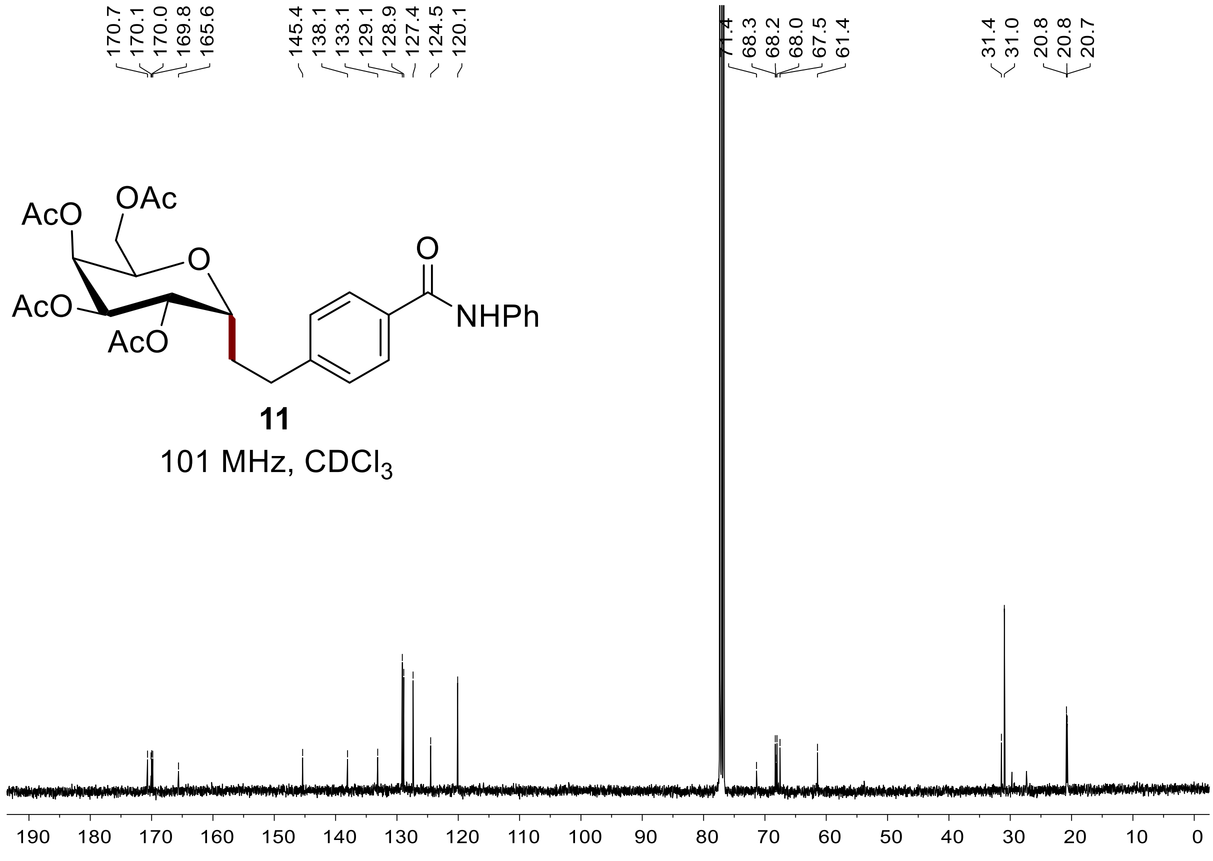
170.7 170.1 170.0 169.8 165.6
145.4 138.1 133.1 129.1 128.9 127.4 124.5 120.1
71.4 68.3 68.2 68.0 67.5 61.4
31.4 31.0 20.8 20.8 20.7
AcO
OAc
O
AcO
AcO
O
NHPh
11
101 MHz, CDCl$_3$
190 180 170 160 150 140 130 120 110 100 90 80 70 60 50 40 30 20 10 0

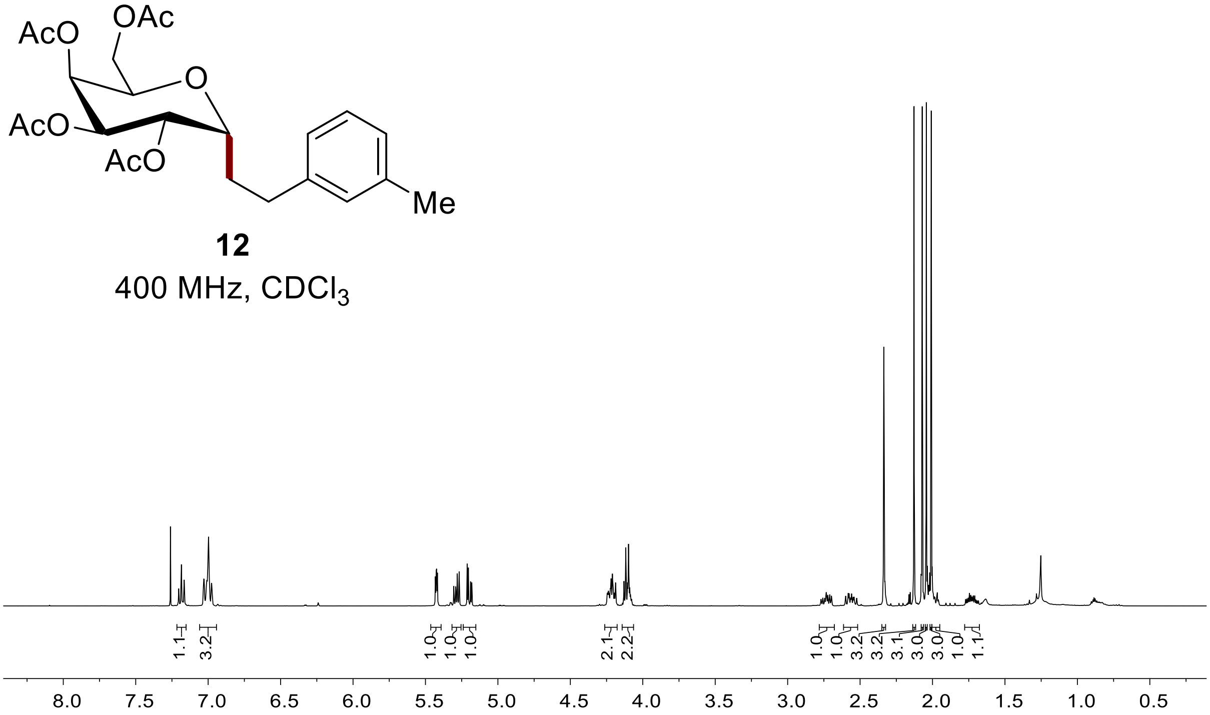
AcO
OAc
O
AcO
AcO
Me
12
400 MHz, CDCl3
1.1
3.2
1.0
1.0
1.0
2.1
2.2
1.0
1.0
3.2
3.2
3.1
3.0
3.0
1.0
1.1
8.0 7.5 7.0 6.5 6.0 5.5 5.0 4.5 4.0 3.5 3.0 2.5 2.0 1.5 1.0 0.5

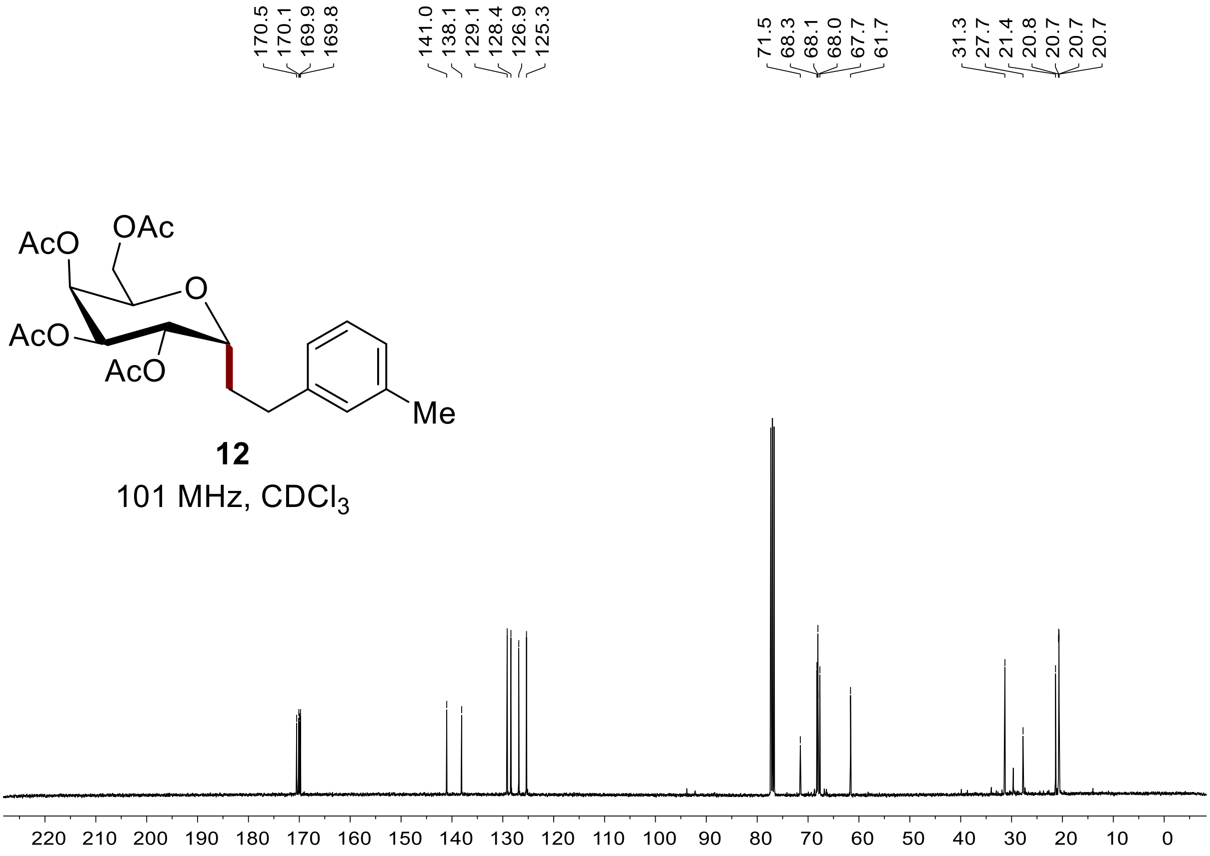
170.5
170.1
169.9
169.8
141.0
138.1
129.1
128.4
126.9
125.3
71.5
68.3
68.1
68.0
67.7
61.7
31.3
27.7
21.4
20.8
20.7
20.7
20.7
AcO
OAc
O
AcO
AcO
Me
12
101 MHz, CDCl3
220 210 200 190 180 170 160 150 140 130 120 110 100 90 80 70 60 50 40 30 20 10 0

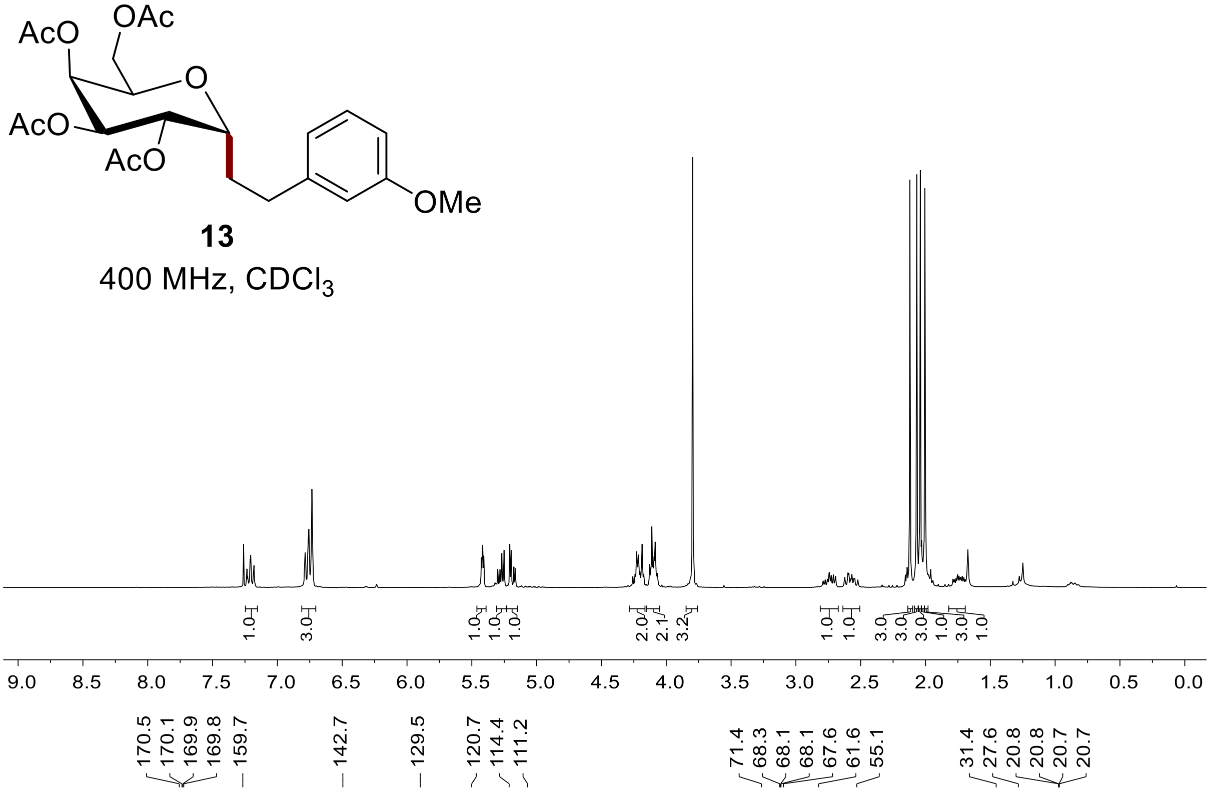
AcO
OAc
O
AcO
AcO
OMe
13
400 MHz, $CDCl_3$
1.0 3.0 1.0 1.0 1.0 2.0 2.1 3.2 1.0 1.0 3.0 3.0 3.0 1.0 3.0 1.0
9.0 8.5 8.0 7.5 7.0 6.5 6.0 5.5 5.0 4.5 4.0 3.5 3.0 2.5 2.0 1.5 1.0 0.5 0.0
170.5 170.1 169.9 169.8 159.7 142.7 129.5 120.7 114.4 111.2 71.4 68.3 68.1 68.1 67.6 61.6 55.1 31.4 27.6 20.8 20.8 20.7 20.7

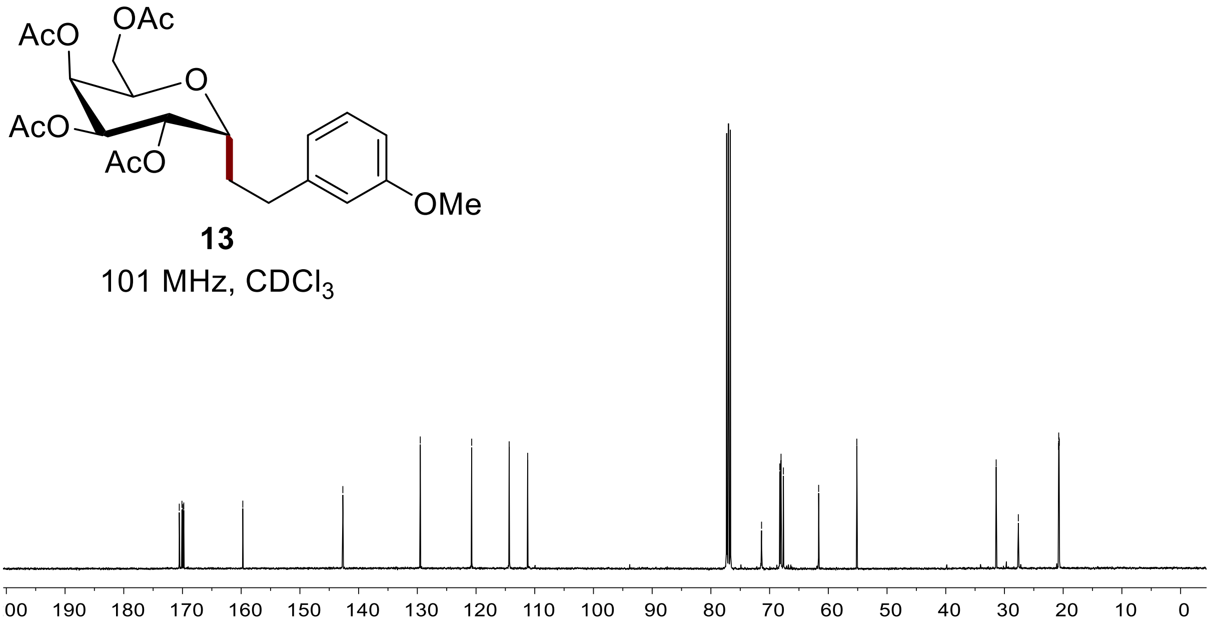
AcO
OAc
O
AcO
AcO
OMe
13
101 MHz, $CDCl_3$
00 190 180 170 160 150 140 130 120 110 100 90 80 70 60 50 40 30 20 10 0

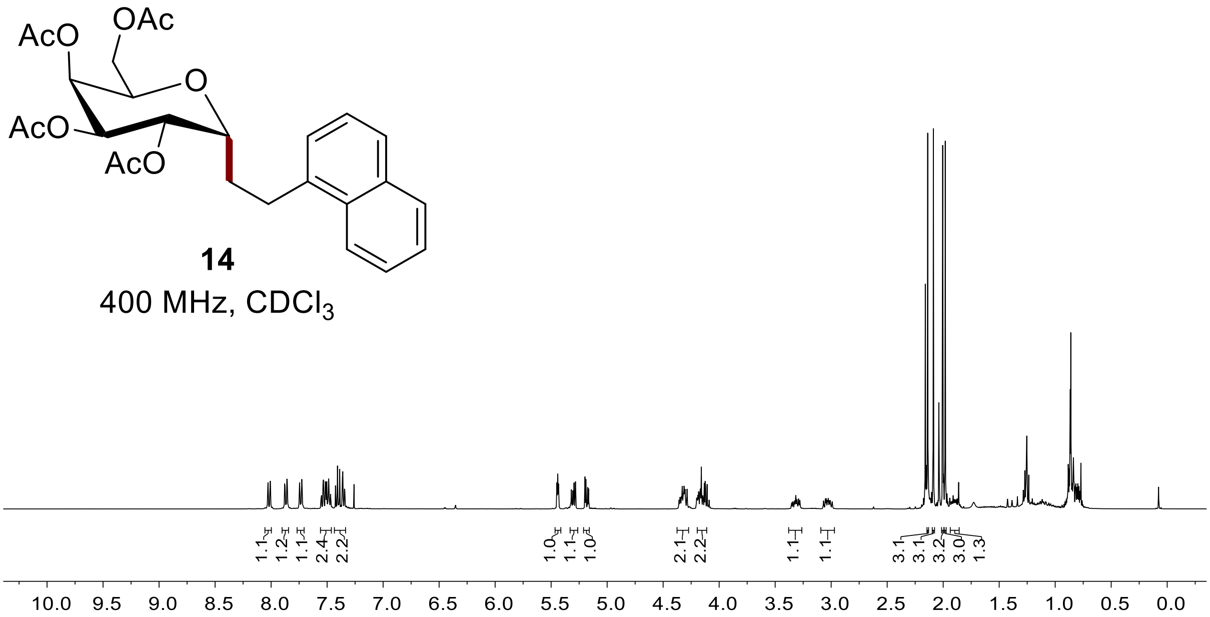

AcO
OAc
O
AcO
AcO
14
400 MHz, CDCl3
1.1
1.2
1.1
2.4
2.2
1.0
1.1
1.0
2.1
2.2
1.1
1.1
3.1
3.1
3.2
3.0
1.3
10.0 9.5 9.0 8.5 8.0 7.5 7.0 6.5 6.0 5.5 5.0 4.5 4.0 3.5 3.0 2.5 2.0 1.5 1.0 0.5 0.0

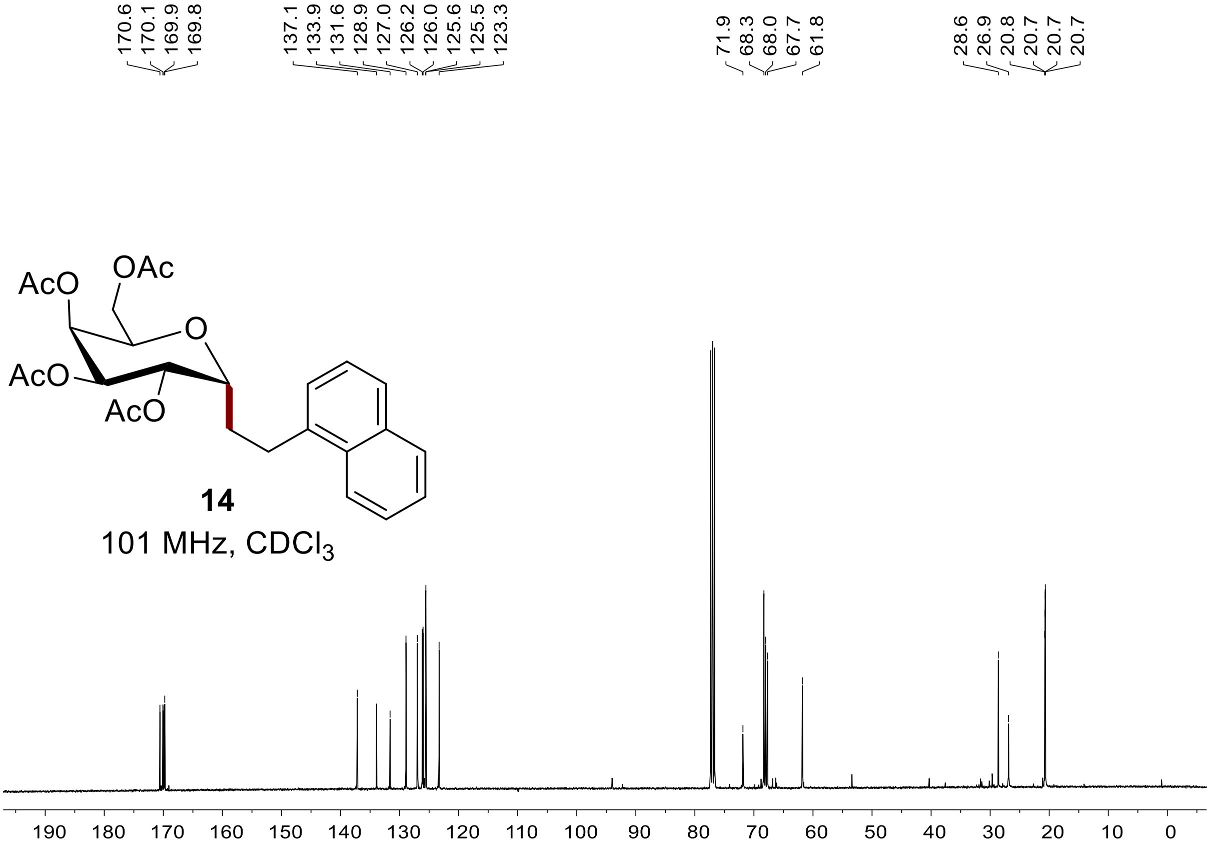

170.6
170.1
169.9
169.8
137.1
133.9
131.6
128.9
127.0
126.2
126.0
125.6
125.5
123.3
71.9
68.3
68.0
67.7
61.8
28.6
26.9
20.8
20.7
20.7
20.7
AcO
OAc
O
AcO
AcO
14
101 MHz, CDCl3
190 180 170 160 150 140 130 120 110 100 90 80 70 60 50 40 30 20 10 0

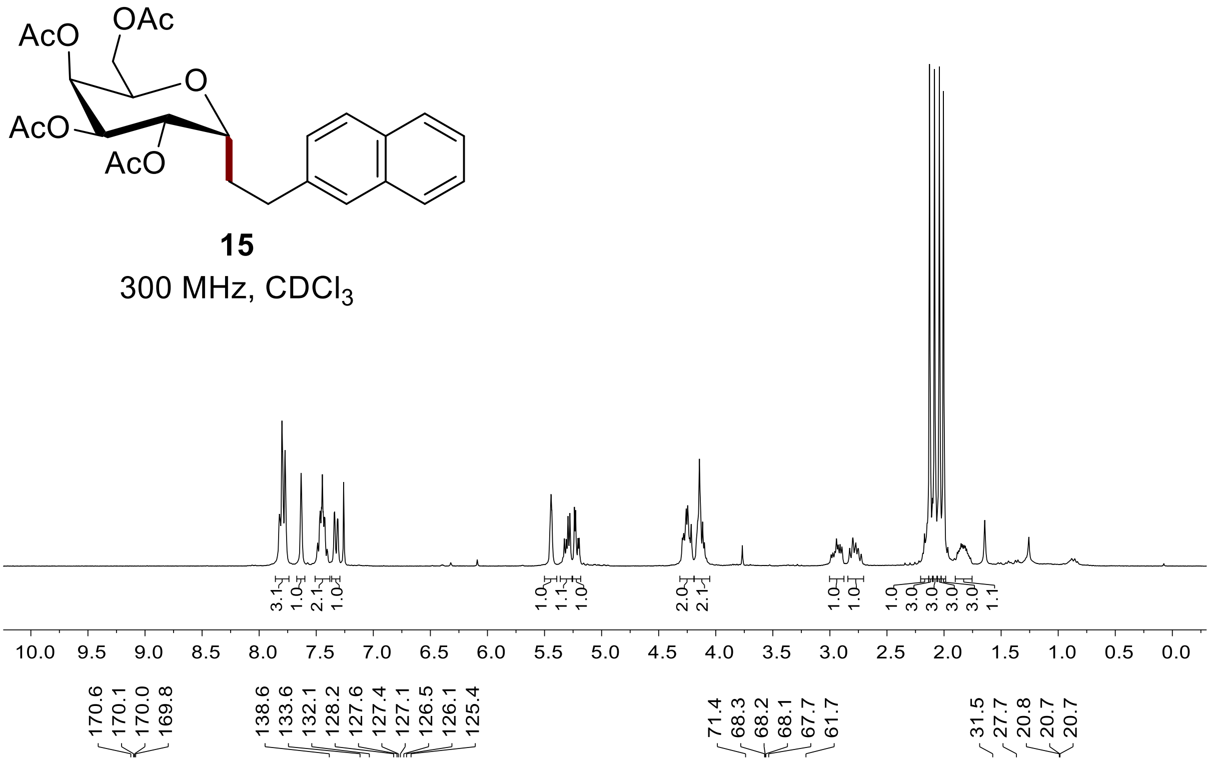
AcO
OAc
O
AcO
AcO
15
300 MHz, CDCl3

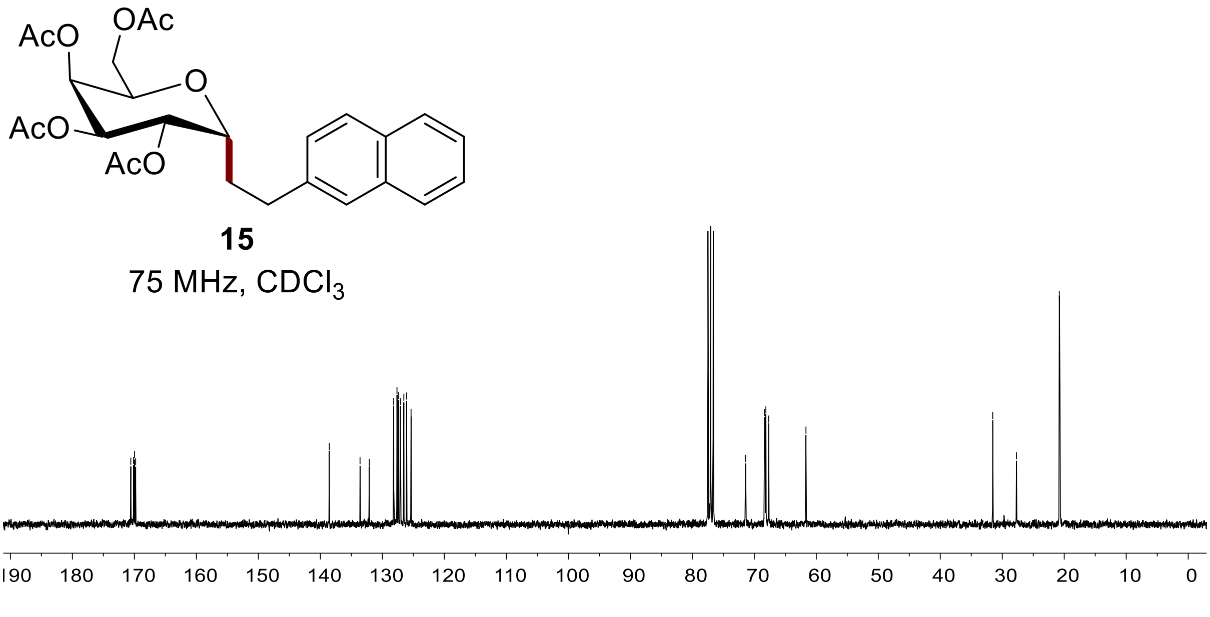
AcO
OAc
O
AcO
AcO
15
75 MHz, CDCl3

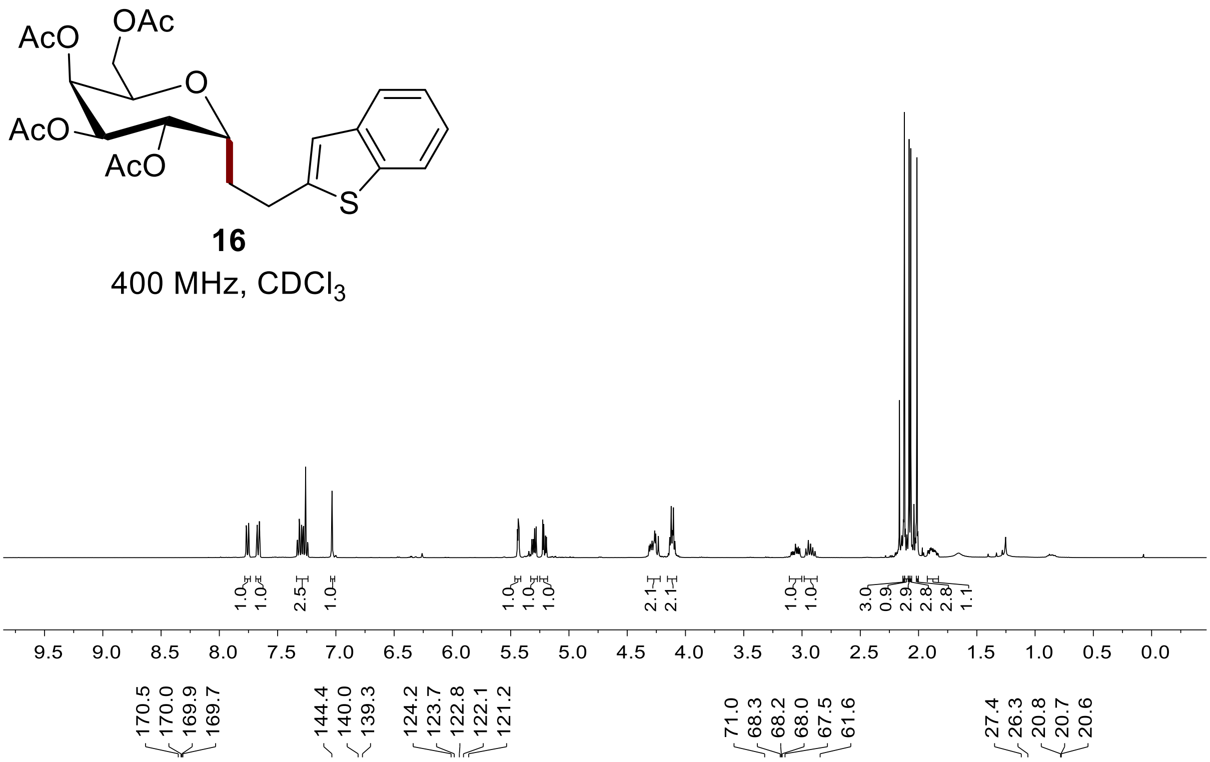
AcO
OAc
O
AcO
AcO
S
16
400 MHz, CDCl$_3$
1.0
1.0
2.5
1.0
1.0
1.0
1.0
2.1
2.1
1.0
1.0
3.0
0.9
2.9
2.8
2.8
1.1
9.5 9.0 8.5 8.0 7.5 7.0 6.5 6.0 5.5 5.0 4.5 4.0 3.5 3.0 2.5 2.0 1.5 1.0 0.5 0.0
170.5
170.0
169.9
169.7
144.4
140.0
139.3
124.2
123.7
122.8
122.1
121.2
71.0
68.3
68.2
68.0
67.5
61.6
27.4
26.3
20.8
20.7
20.6

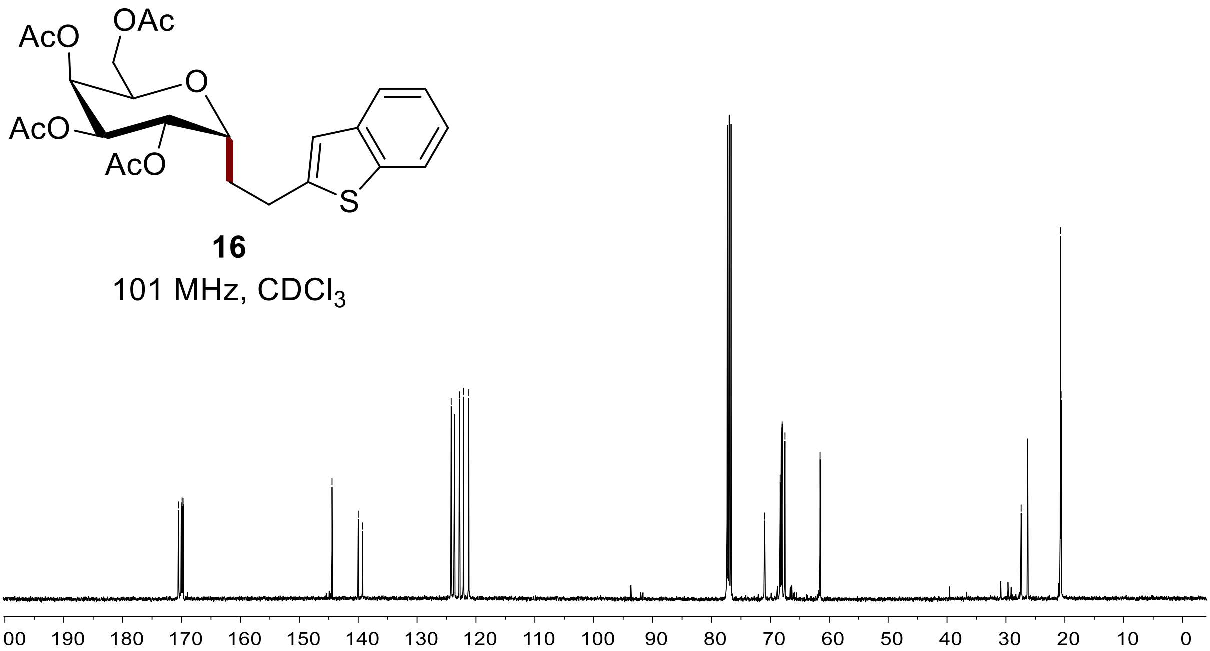
AcO
OAc
O
AcO
AcO
S
16
101 MHz, CDCl$_3$
00 190 180 170 160 150 140 130 120 110 100 90 80 70 60 50 40 30 20 10 0

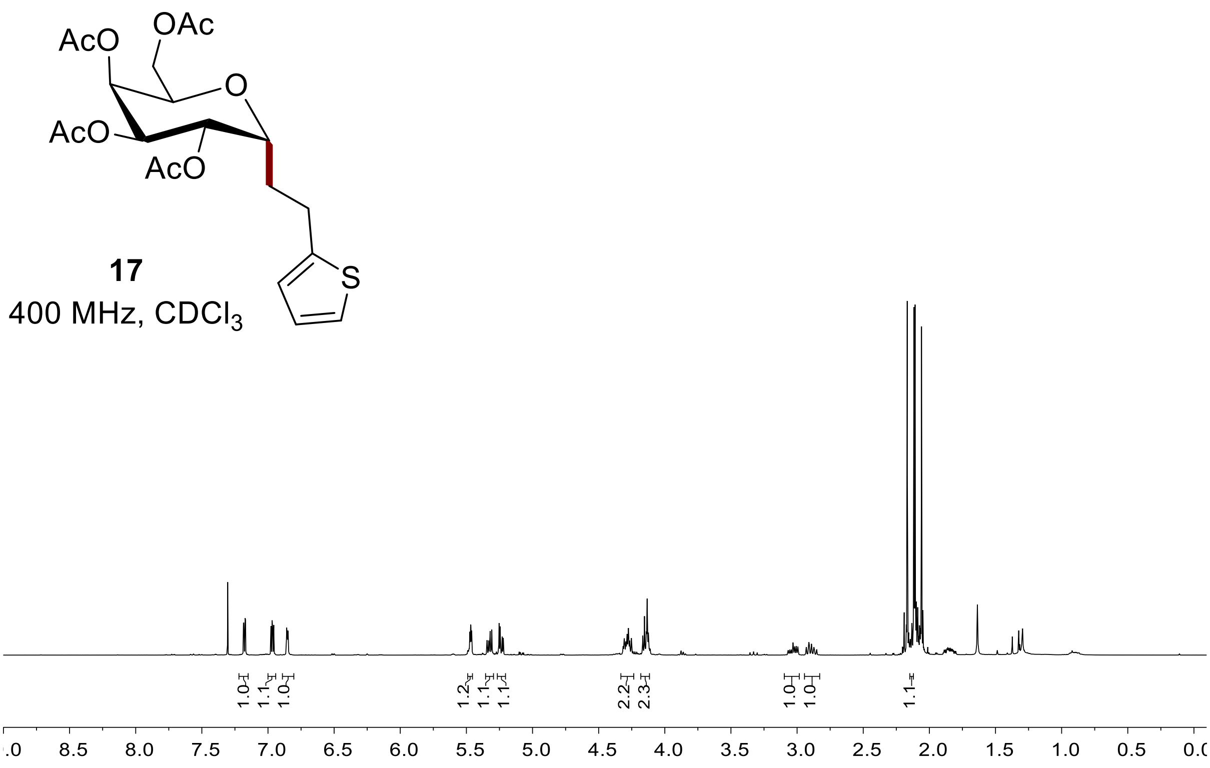
AcO
OAc
O
AcO
AcO
S
17
400 MHz, CDCl3
1.0
1.1
1.0
1.2
1.1
1.1
2.2
2.3
1.0
1.0
1.1
8.5 8.0 7.5 7.0 6.5 6.0 5.5 5.0 4.5 4.0 3.5 3.0 2.5 2.0 1.5 1.0 0.5

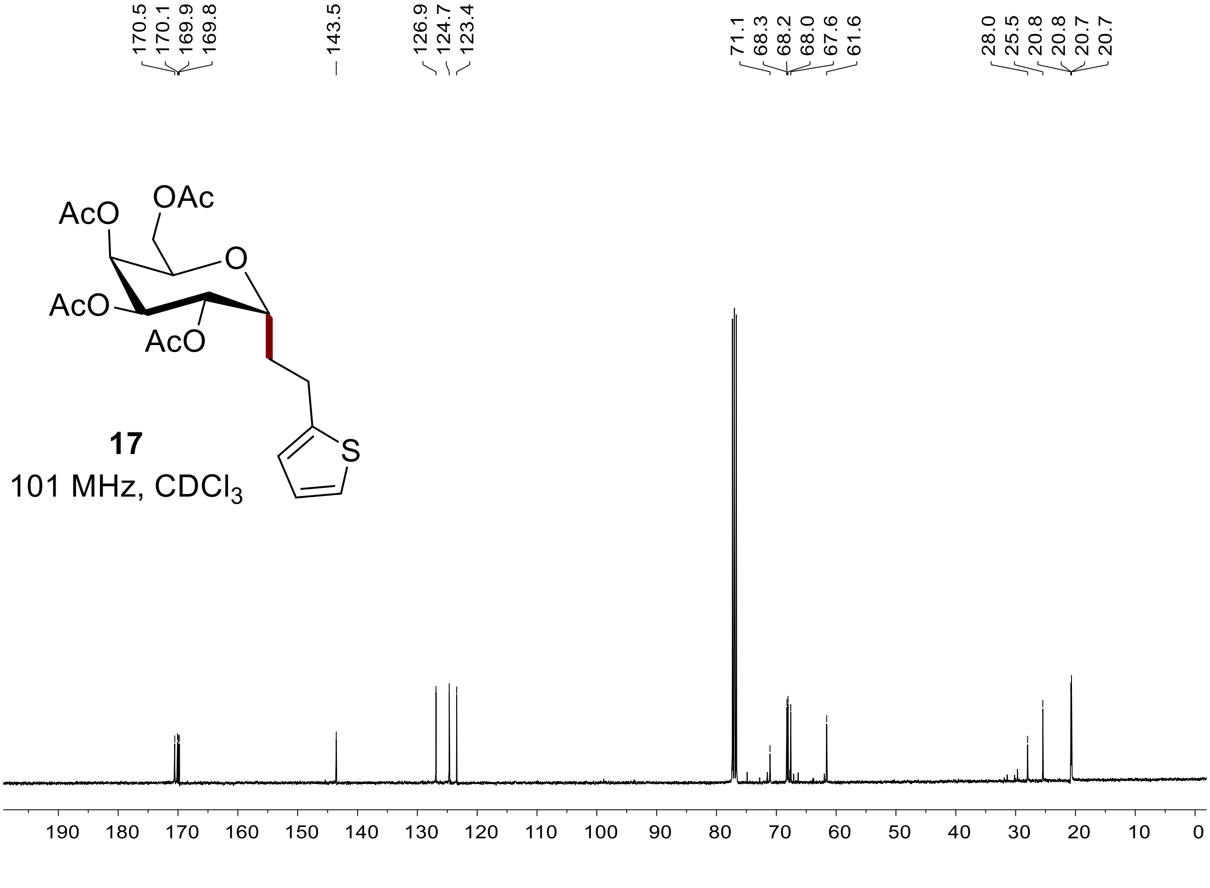
170.5 170.1 169.9 169.8
143.5
126.9 124.7 123.4
71.1 68.3 68.2 68.0 67.6 61.6
28.0 25.5 20.8 20.8 20.7 20.7
AcO
OAc
O
AcO
AcO
S
17
101 MHz, CDCl3
190 180 170 160 150 140 130 120 110 100 90 80 70 60 50 40 30 20 10 0

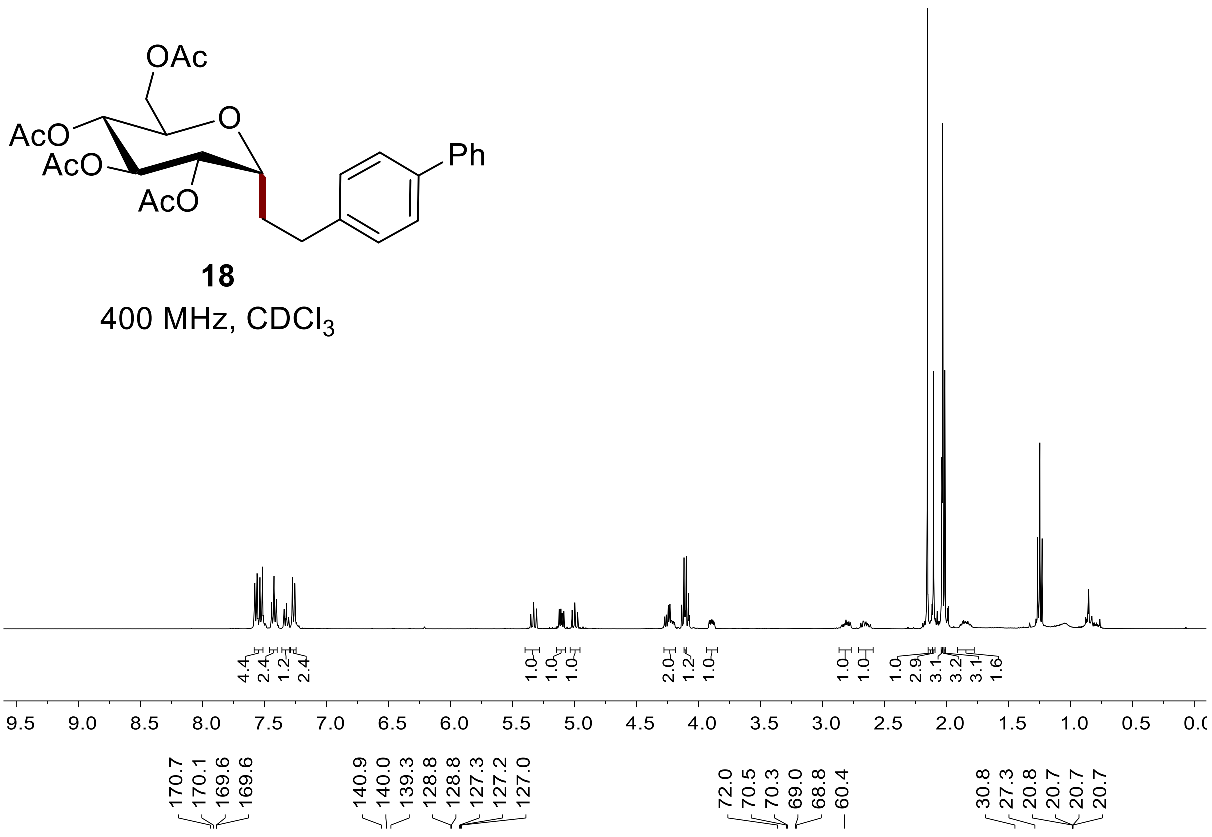
OAc
AcO
AcO
AcO
O
Ph
18
400 MHz, $CDCl_3$
4.4 2.4 1.2 2.4
1.0 1.0 1.0
2.0 1.2 1.0
1.0 1.0
1.0 2.9 3.1 3.2 3.1 1.6
9.5 9.0 8.5 8.0 7.5 7.0 6.5 6.0 5.5 5.0 4.5 4.0 3.5 3.0 2.5 2.0 1.5 1.0 0.5
170.7 170.1 169.6 169.6
140.9 140.0 139.3 128.8 128.8 127.3 127.2 127.0
72.0 70.5 70.3 69.0 68.8 60.4
30.8 27.3 20.8 20.7 20.7 20.7

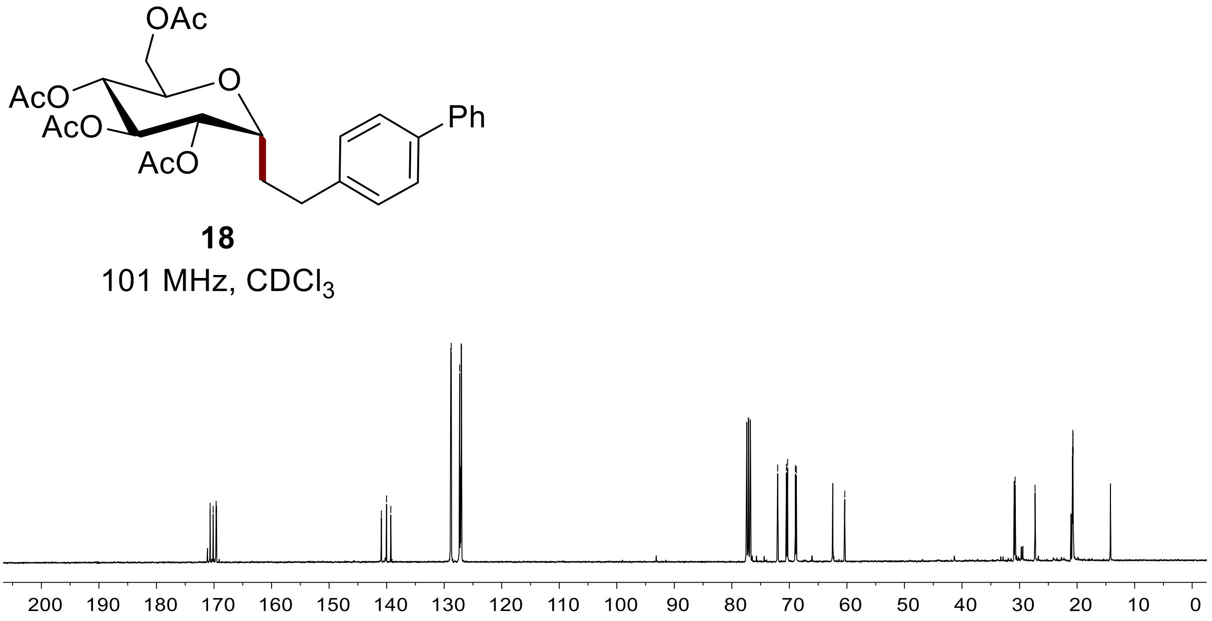
OAc
AcO
AcO
AcO
O
Ph
18
101 MHz, $CDCl_3$
200 190 180 170 160 150 140 130 120 110 100 90 80 70 60 50 40 30 20 10 0

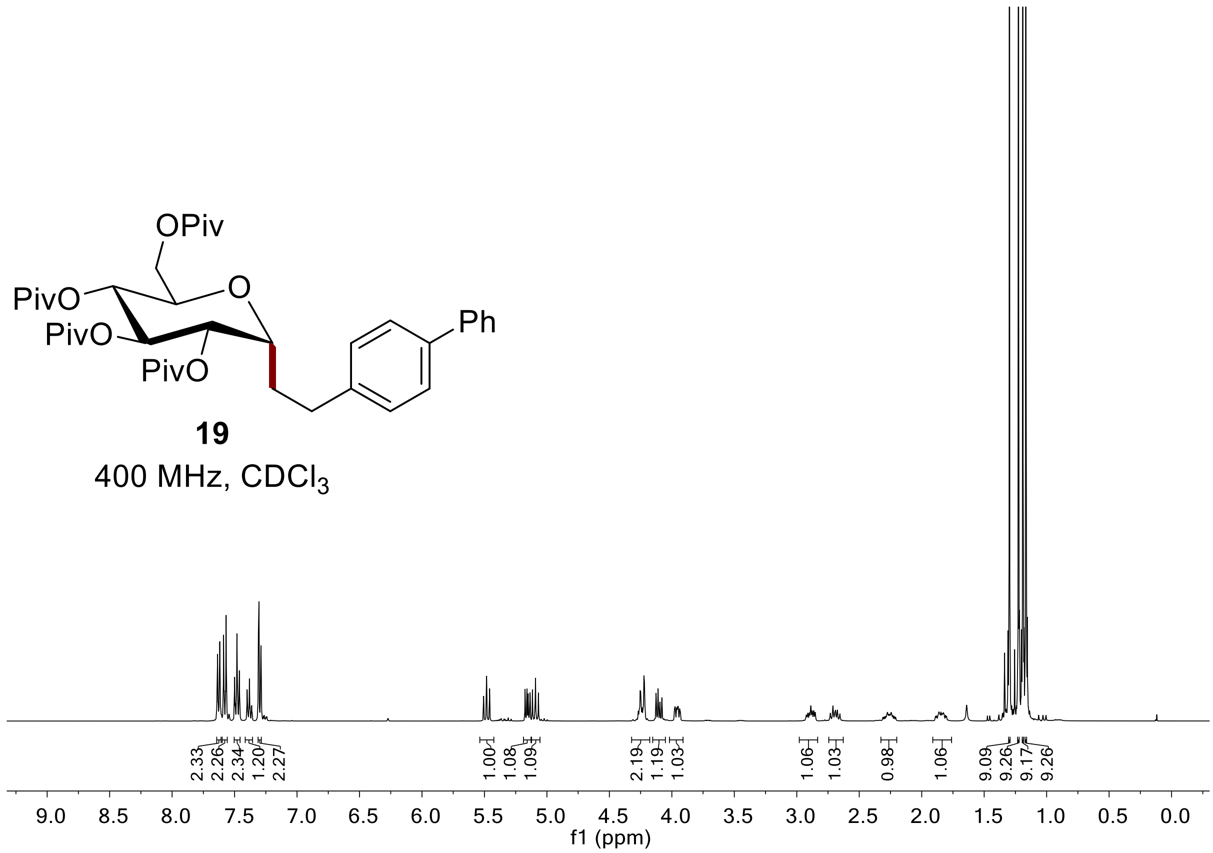
OPiv
PivO
PivO
PivO
O
Ph
19
400 MHz, CDCl3
2.33
2.26
2.34
1.20
2.27
1.00
1.08
1.09
2.19
1.19
1.03
1.06
1.03
0.98
1.06
9.09
9.26
9.17
9.26
9.0 8.5 8.0 7.5 7.0 6.5 6.0 5.5 5.0 4.5 4.0 3.5 3.0 2.5 2.0 1.5 1.0 0.5 0.0
f1 (ppm)

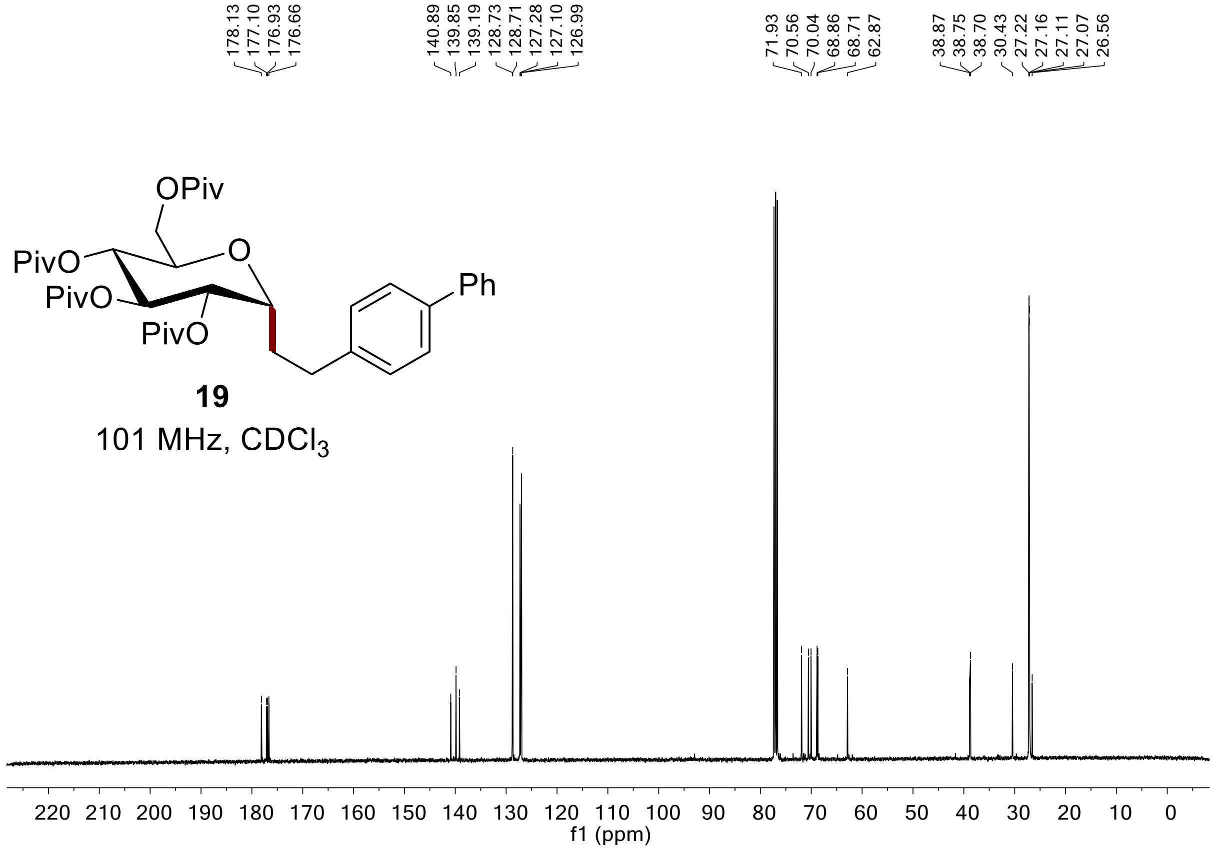
178.13
177.10
176.93
176.66
140.89
139.85
139.19
128.73
128.71
127.28
127.10
126.99
71.93
70.56
70.04
68.86
68.71
62.87
38.87
38.75
38.70
30.43
27.22
27.16
27.11
27.07
26.56
OPiv
PivO
PivO
PivO
O
Ph
19
101 MHz, CDCl3
220 210 200 190 180 170 160 150 140 130 120 110 100 90 80 70 60 50 40 30 20 10 0
f1 (ppm)

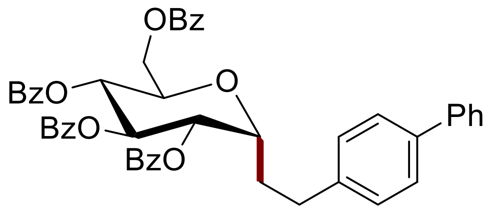

20

400 MHz, $CDCl_3$

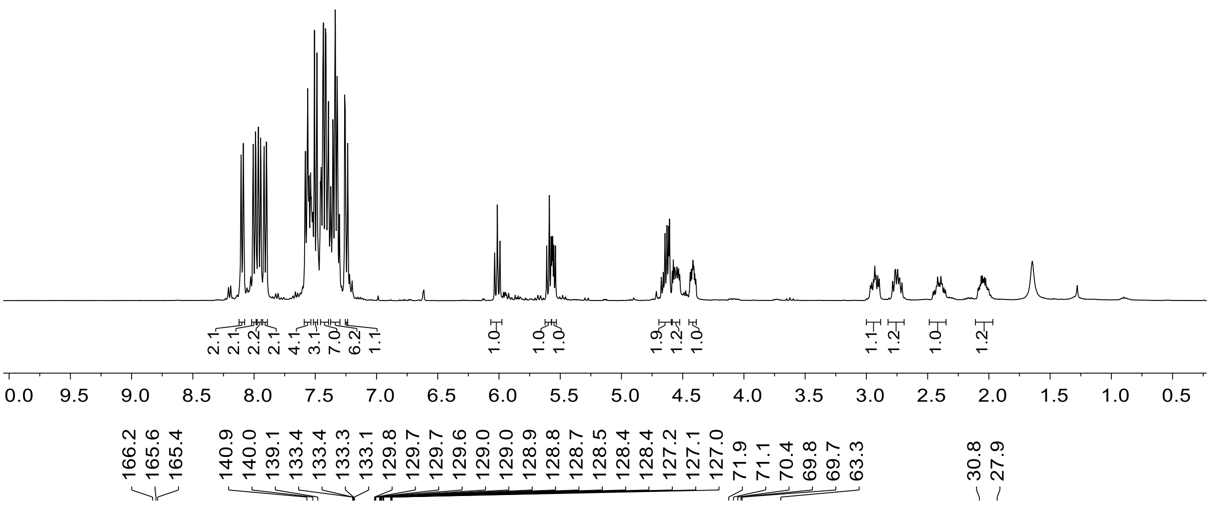

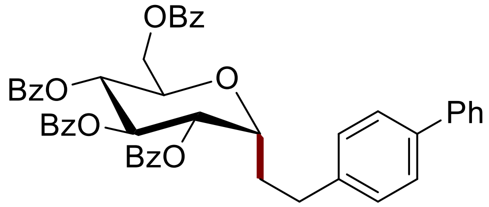

20

101 MHz, $CDCl_3$

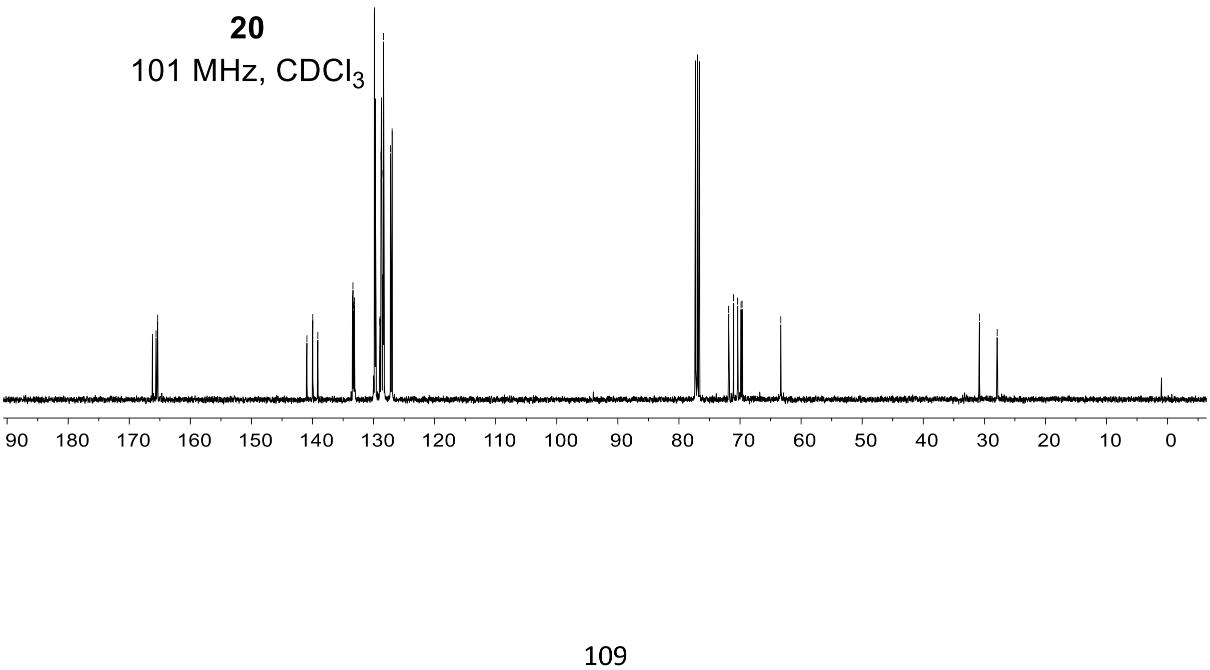

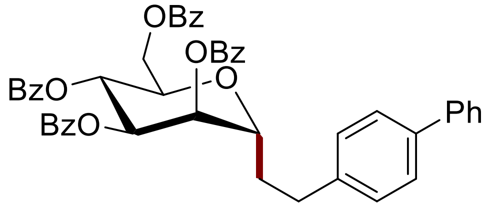

21a

300 MHz, $CDCl_3$

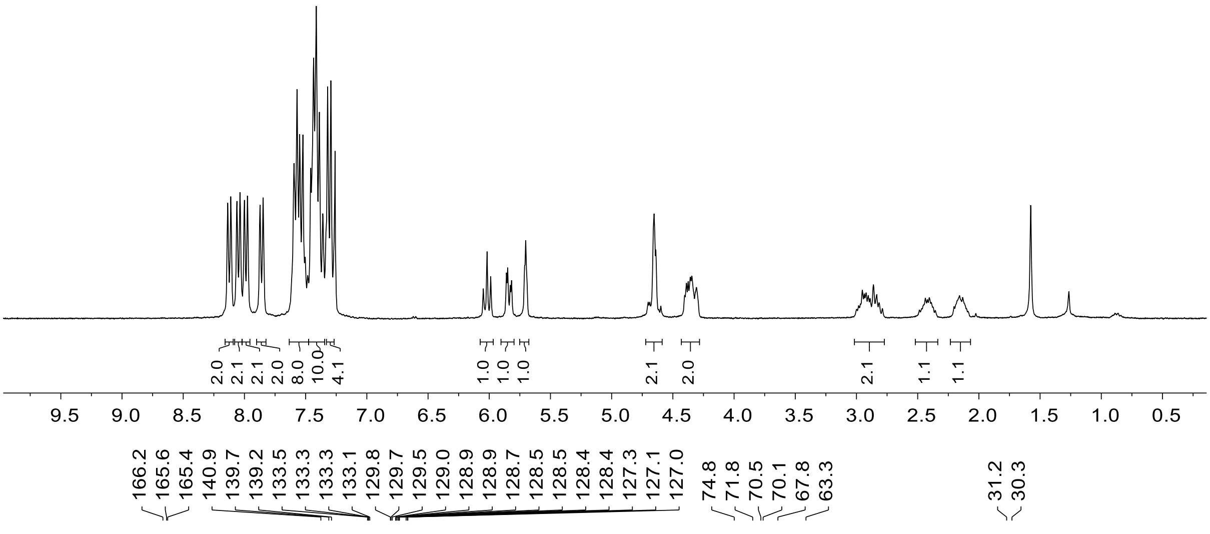

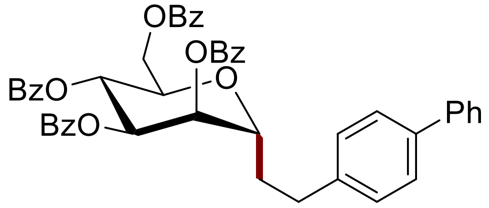

21a

75 MHz, $CDCl_3$

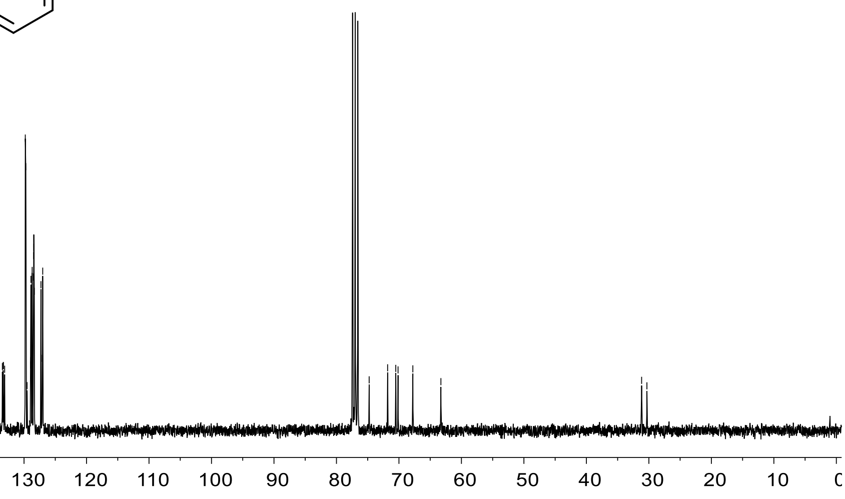

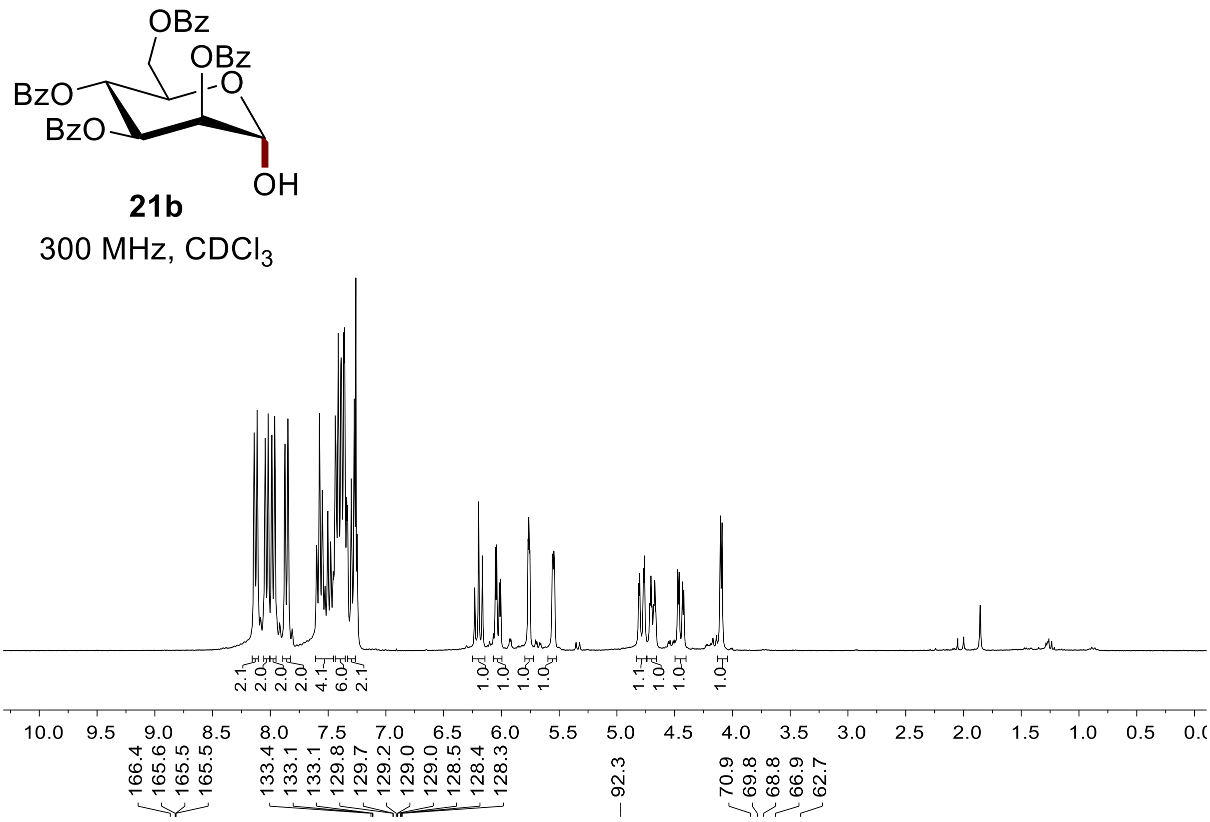
OBz
OBz
BzO
O
BzO
OH
21b
300 MHz, $CDCl_3$
2.1 2.0 2.0 2.0 4.1 6.0 2.1 1.0 1.0 1.0 1.0 1.1 1.0 1.0 1.0
10.0 9.5 9.0 8.5 8.0 7.5 7.0 6.5 6.0 5.5 5.0 4.5 4.0 3.5 3.0 2.5 2.0 1.5 1.0 0.5
166.4 165.6 165.5 165.5
133.4 133.1 133.1 129.8 129.7 129.2 129.0 129.0 128.5 128.4 128.3
92.3
70.9 69.8 68.8 66.9 62.7

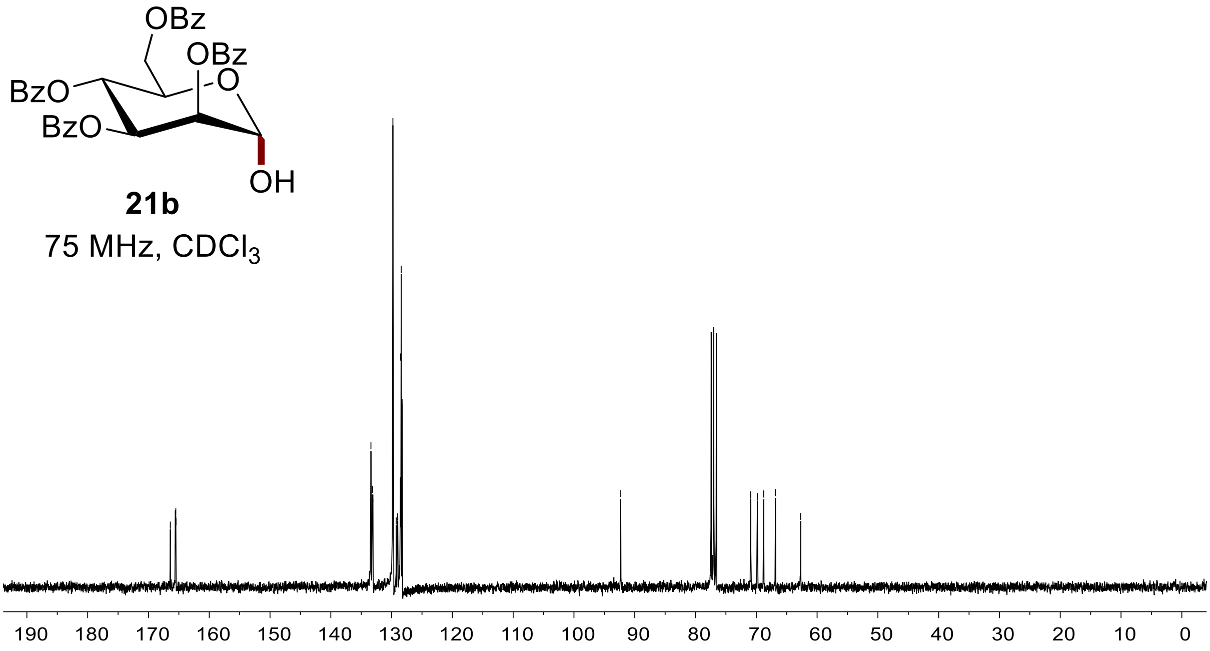
OBz
OBz
BzO
O
BzO
OH
21b
75 MHz, $CDCl_3$
190 180 170 160 150 140 130 120 110 100 90 80 70 60 50 40 30 20 10 0

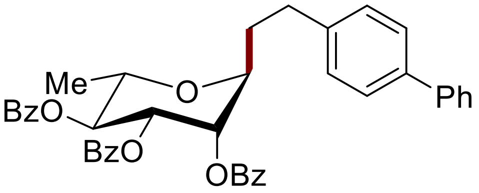

22
400 MHz, CDCl$_3$

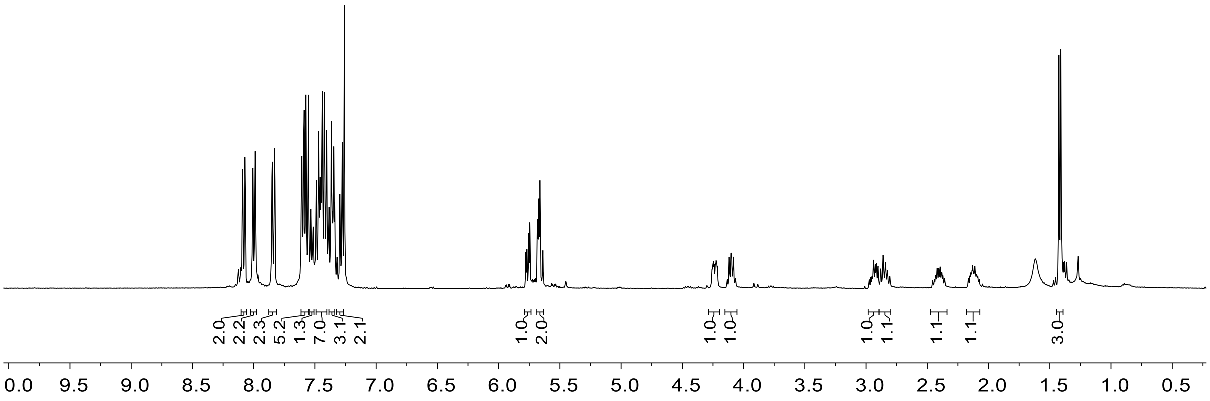

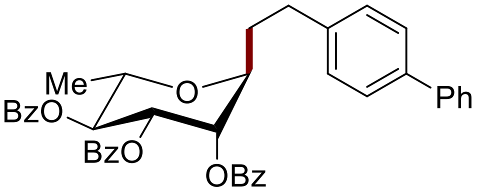

22
101 MHz, CDCl$_3$

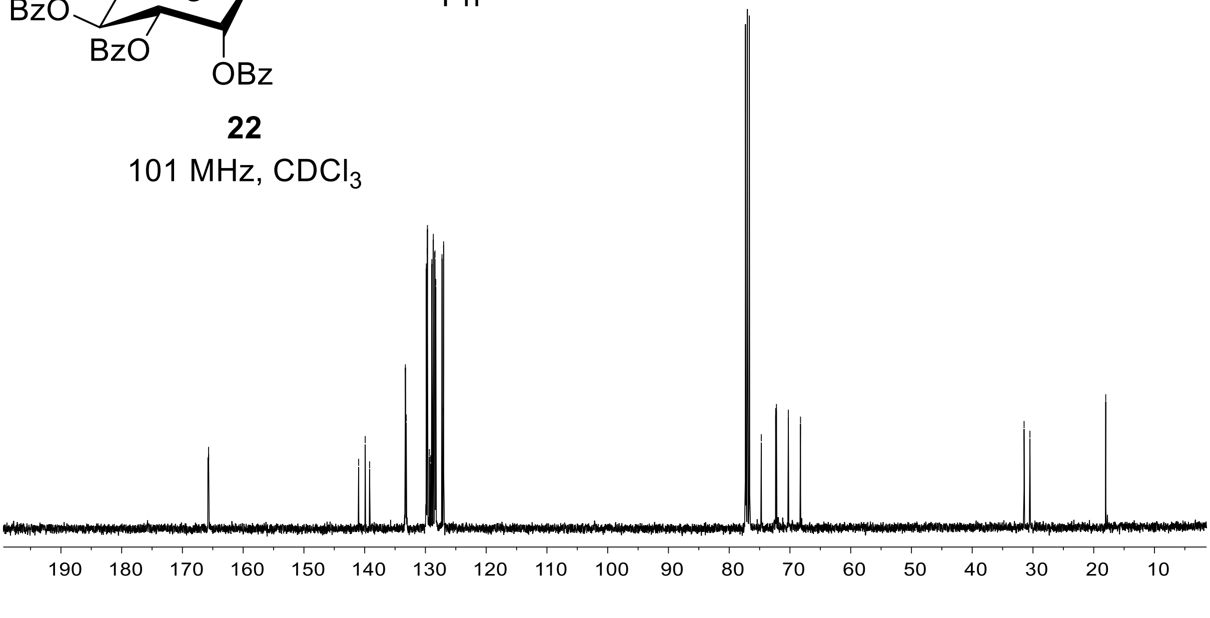

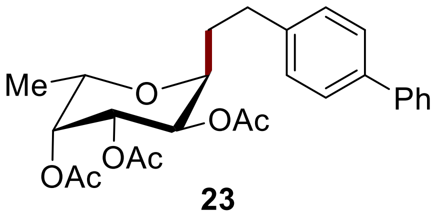

23

400 MHz, $CDCl_3$

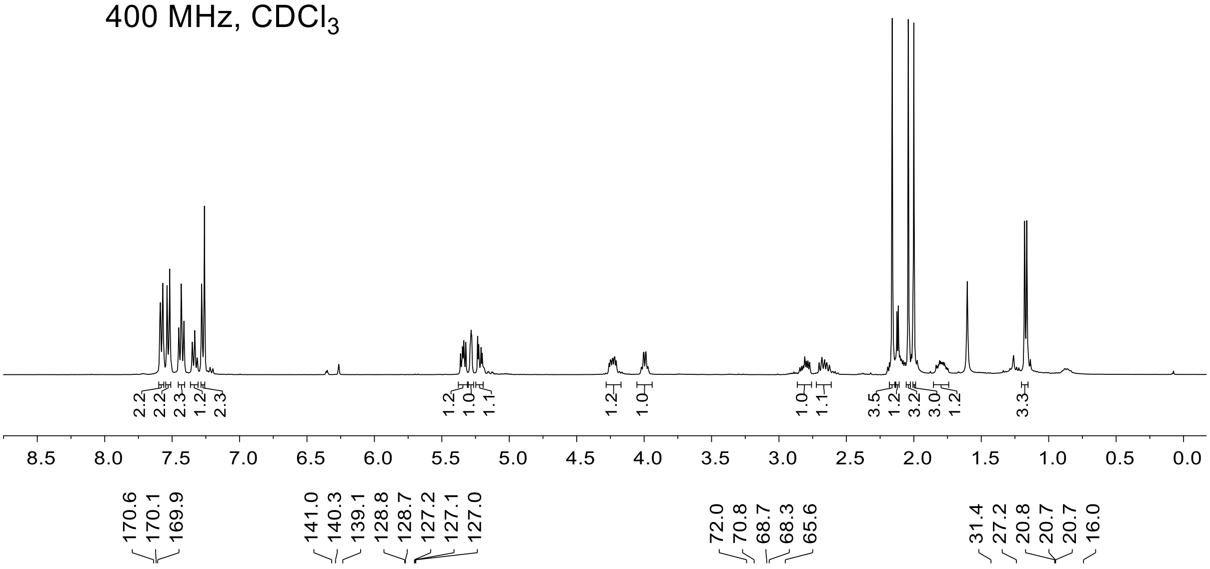

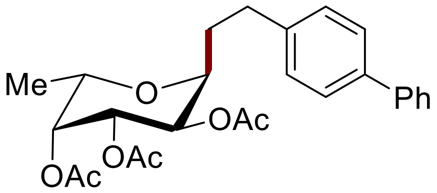

23

101 MHz, $CDCl_3$

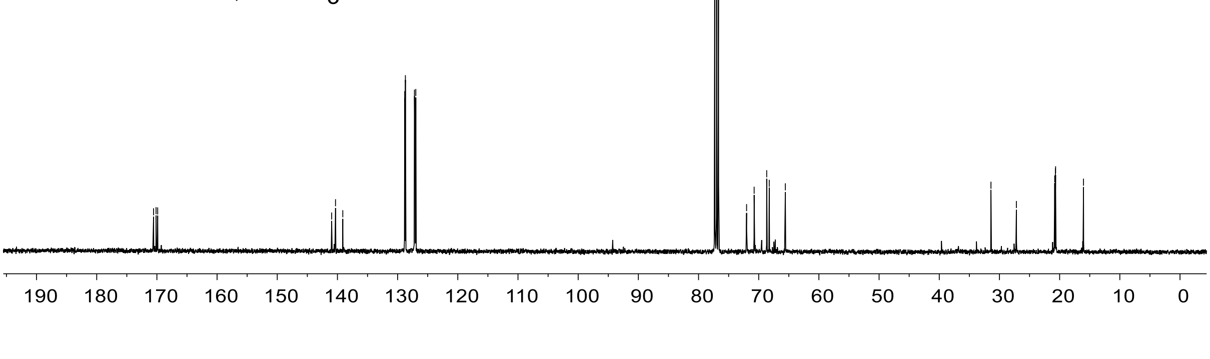

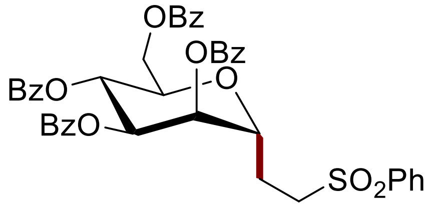

24

300 MHz, $CDCl_3$

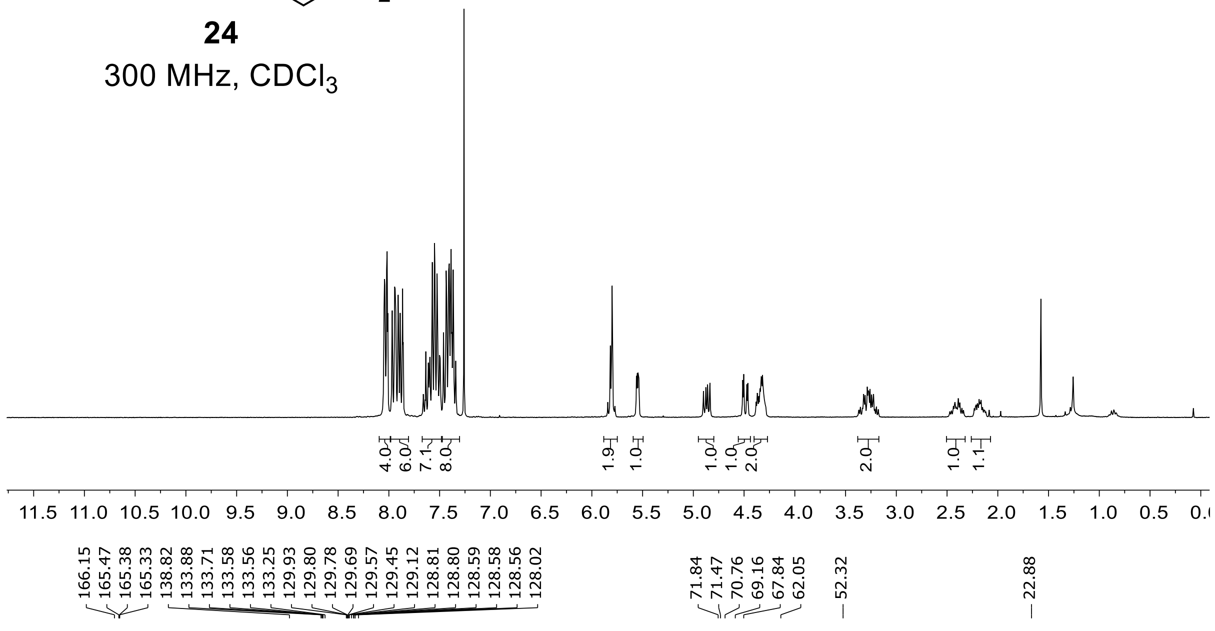

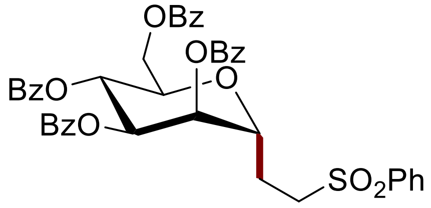

24

101 MHz, $CDCl_3$

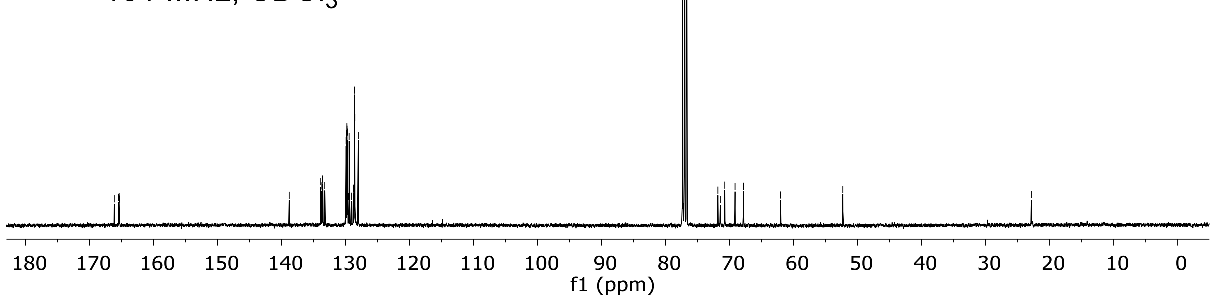

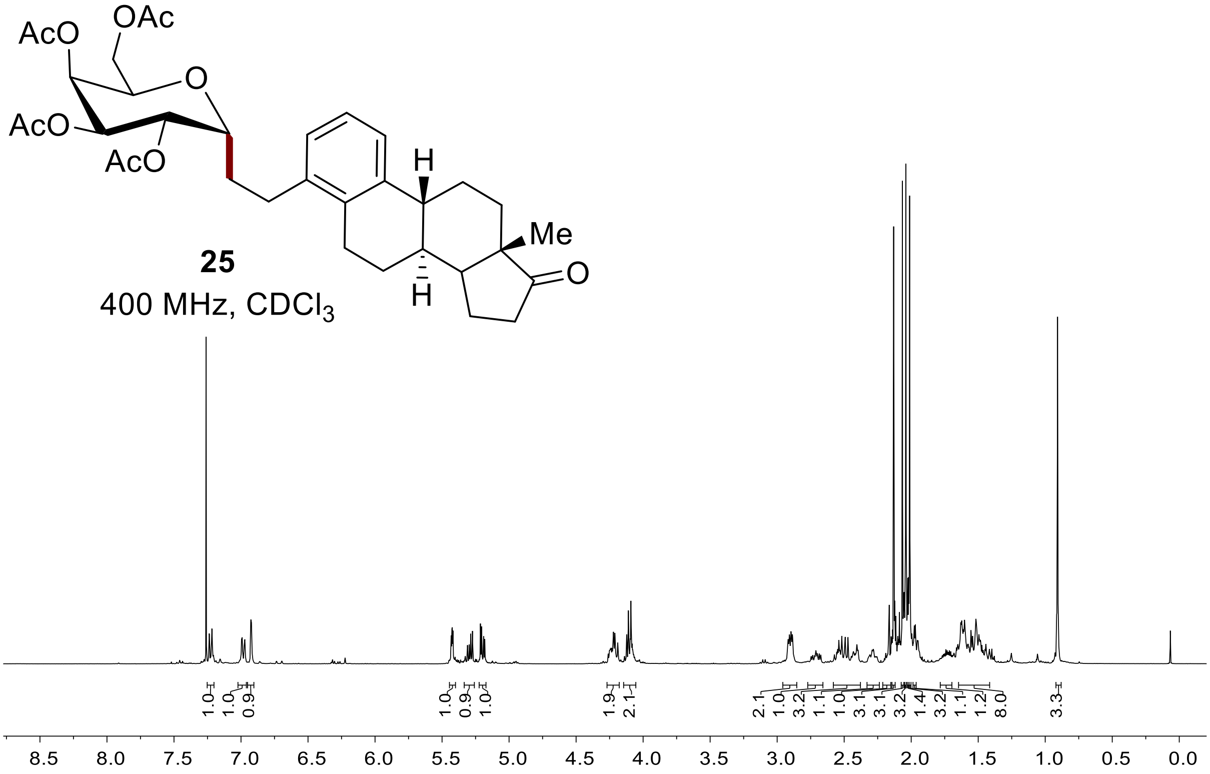
AcO
OAc
AcO
AcO
H
H
Me
O
25
400 MHz, $CDCl_3$
1.0 1.0 0.9
1.0 0.9 1.0
1.9 2.1
2.1 1.0 3.2 1.1 1.0 3.1 3.1 3.2 1.4 3.2 1.1 1.2 8.0
3.3
8.5 8.0 7.5 7.0 6.5 6.0 5.5 5.0 4.5 4.0 3.5 3.0 2.5 2.0 1.5 1.0 0.5 0.0

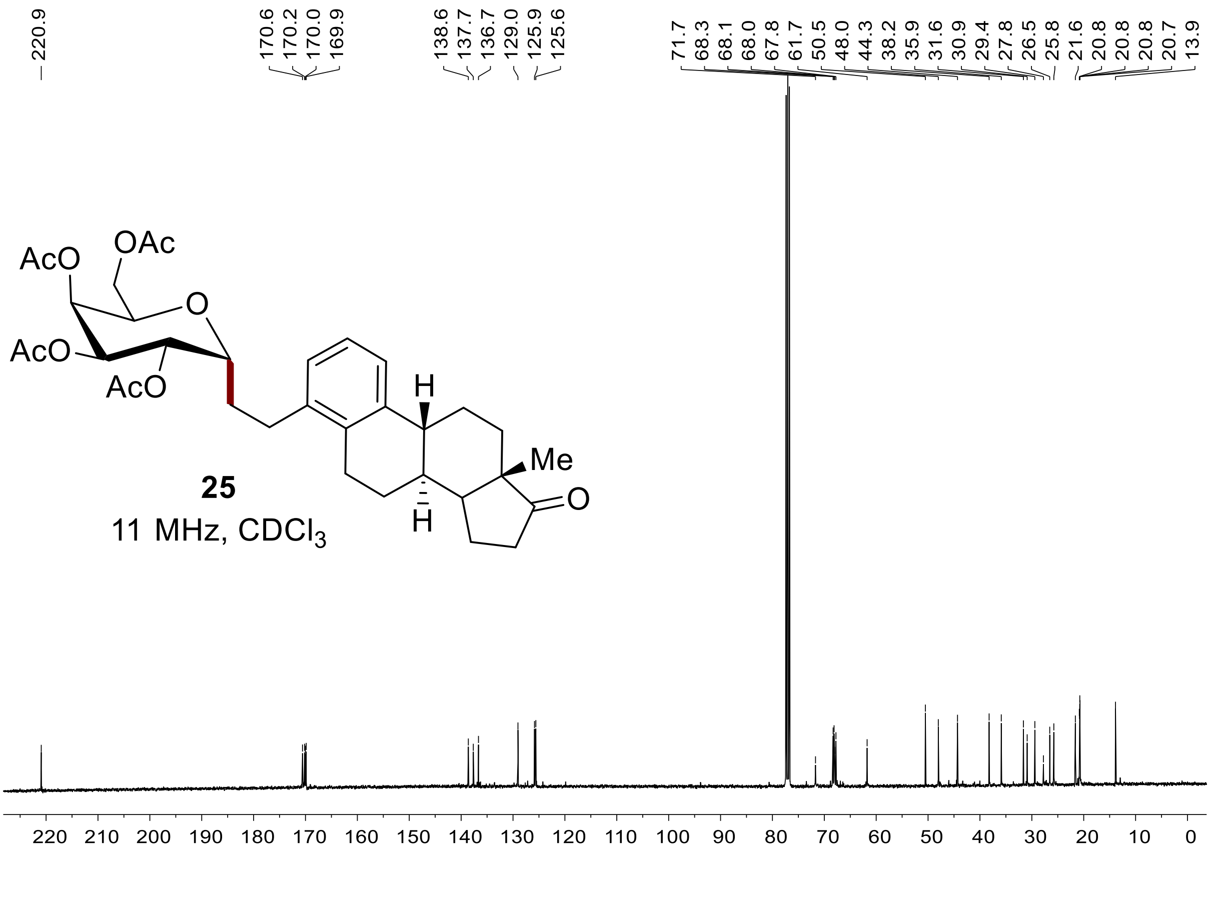
220.9
170.6 170.2 170.0 169.9
138.6 137.7 136.7 129.0 125.9 125.6
71.7 68.3 68.1 68.0 67.8 61.7 50.5 48.0 44.3 38.2 35.9 31.6 30.9 29.4 27.8 26.5 25.8 21.6 20.8 20.8 20.7 13.9
AcO
OAc
AcO
AcO
H
H
Me
O
25
11 MHz, $CDCl_3$
220 210 200 190 180 170 160 150 140 130 120 110 100 90 80 70 60 50 40 30 20 10 0

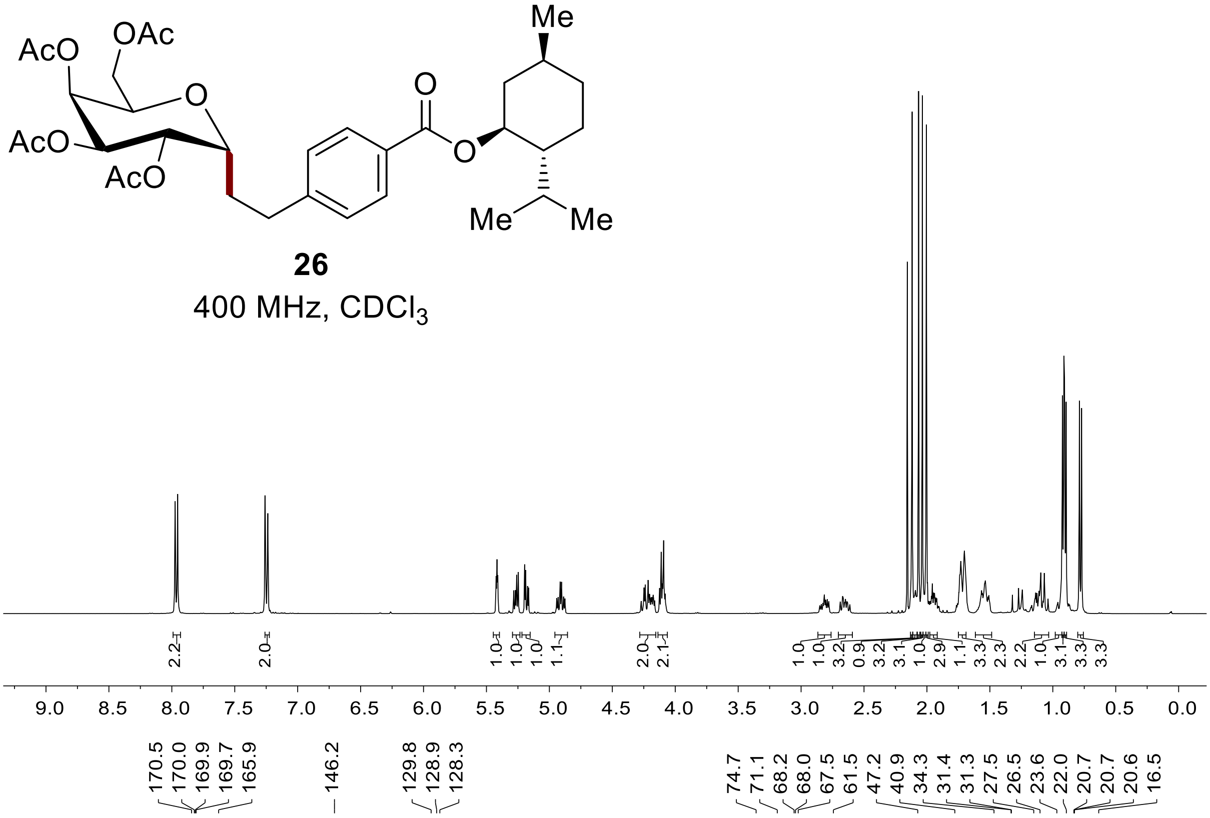
AcO
OAc
O
AcO
AcO
O
O
Me
Me
Me
26
400 MHz, CDCl3
2.2
2.0
1.0
1.0
1.0
1.1
2.0
2.1
1.0
1.0
3.2
0.9
3.2
3.1
1.0
2.9
1.1
3.3
2.3
2.2
1.0
3.1
3.3
3.3
9.0 8.5 8.0 7.5 7.0 6.5 6.0 5.5 5.0 4.5 4.0 3.5 3.0 2.5 2.0 1.5 1.0 0.5 0.0
170.5
170.0
169.9
169.7
165.9
146.2
129.8
128.9
128.3
74.7
71.1
68.2
68.0
67.5
61.5
47.2
40.9
34.3
31.4
31.3
27.5
26.5
23.6
22.0
20.7
20.7
20.6
16.5

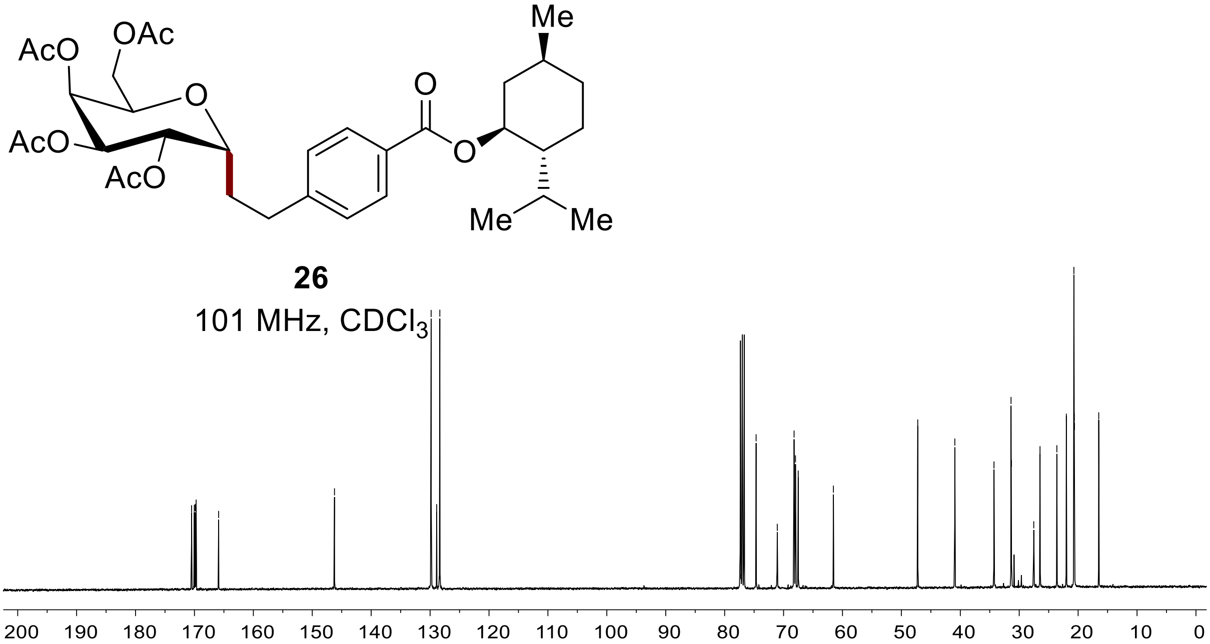
AcO
OAc
O
AcO
AcO
O
O
Me
Me
Me
26
101 MHz, CDCl3
200 190 180 170 160 150 140 130 120 110 100 90 80 70 60 50 40 30 20 10 0

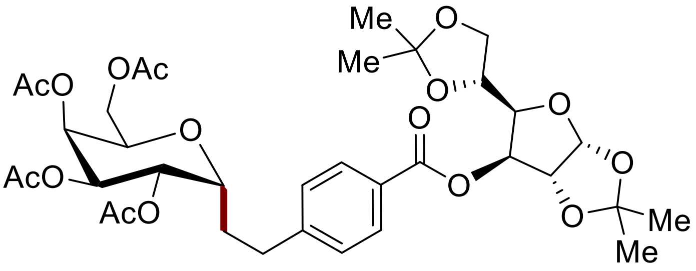

27
400 MHz, $CDCl_3$

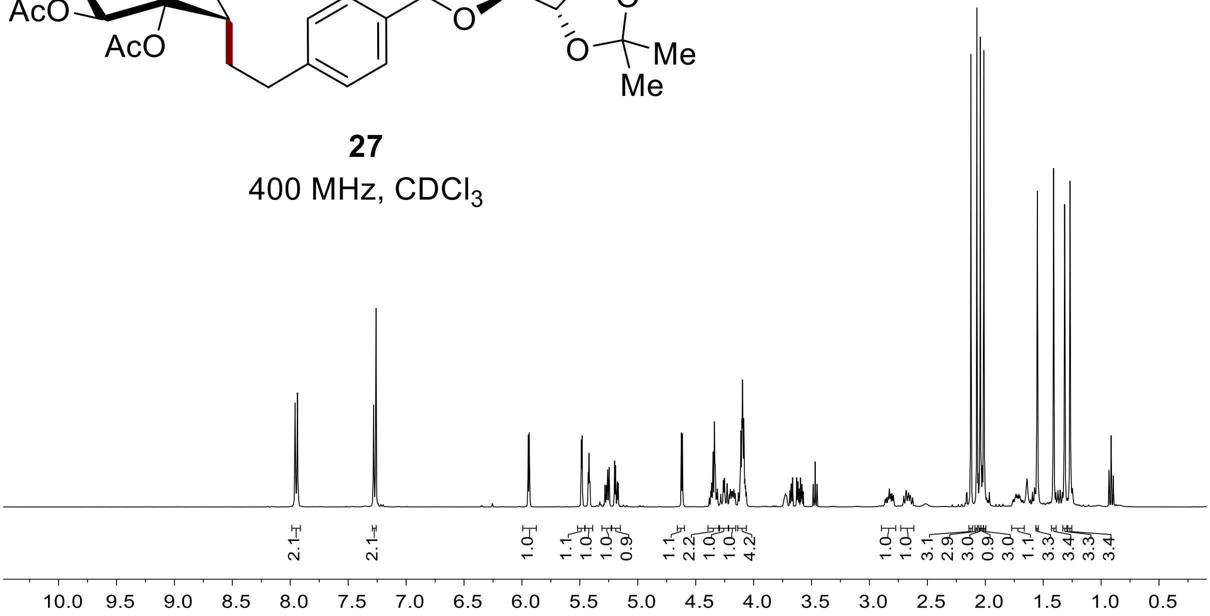

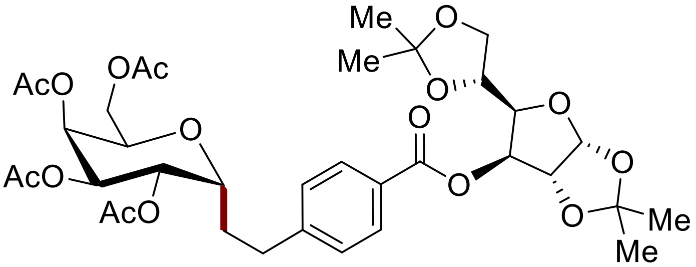

27
101 MHz, $CDCl_3$

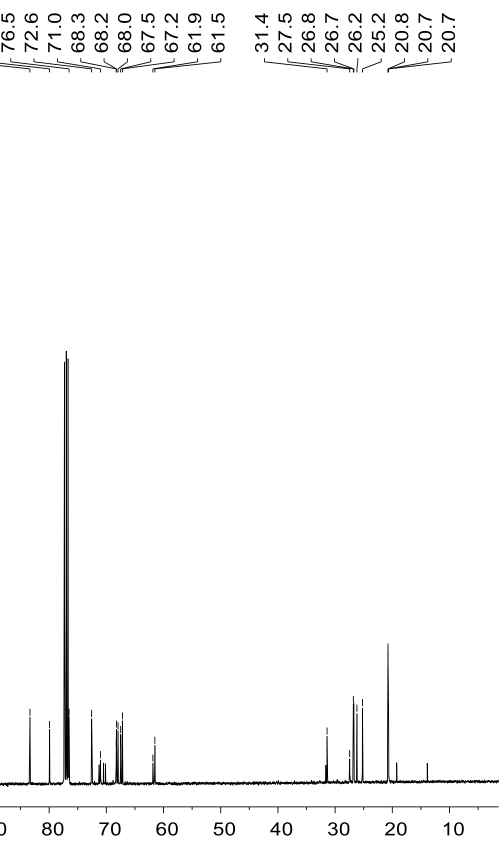

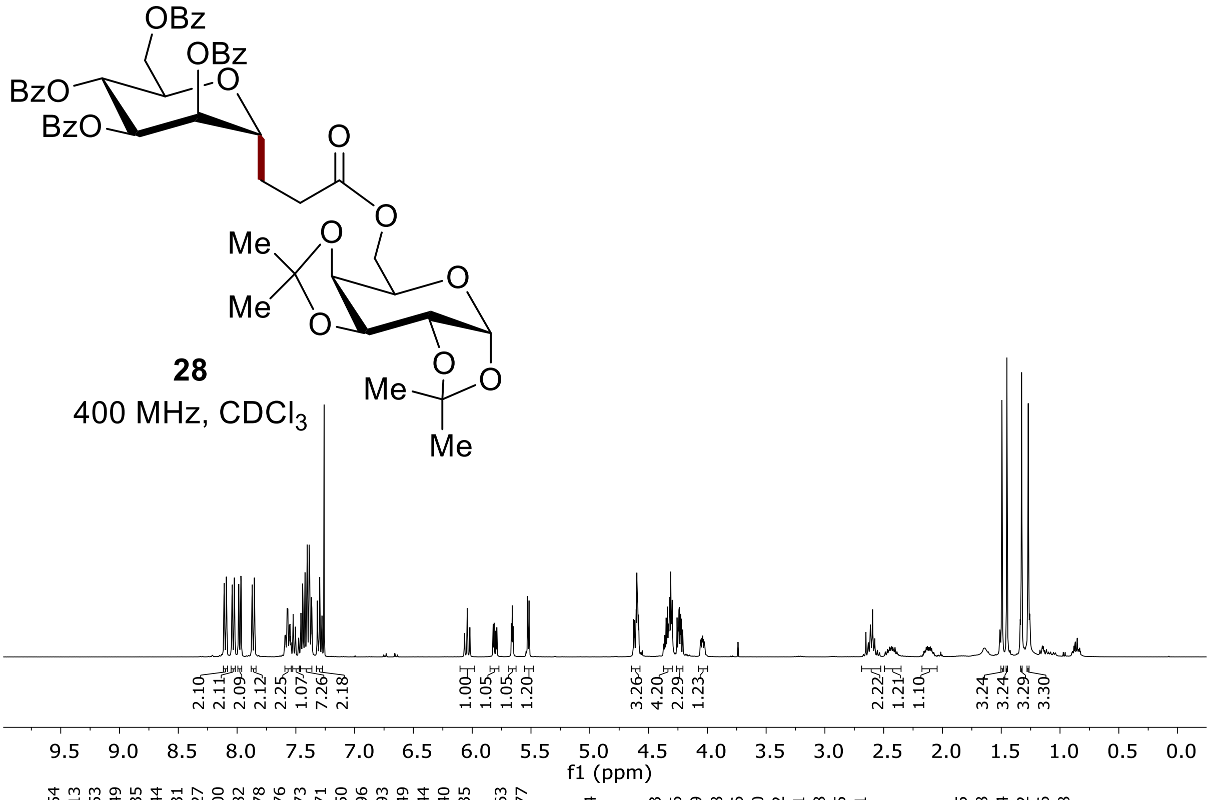
OBz
OBz
BzO
BzO
O
O
O
Me
Me
O
O
O
O
Me
Me
28
400 MHz, $CDCl_3$
2.10
2.11
2.09
2.12
2.25
1.07
7.26
2.18
1.00
1.05
1.05
1.20
3.26
4.20
2.29
1.23
2.22
1.21
1.10
3.24
3.24
3.29
3.30
9.5 9.0 8.5 8.0 7.5 7.0 6.5 6.0 5.5 5.0 4.5 4.0 3.5 3.0 2.5 2.0 1.5 1.0 0.5 0.0
f1 (ppm)

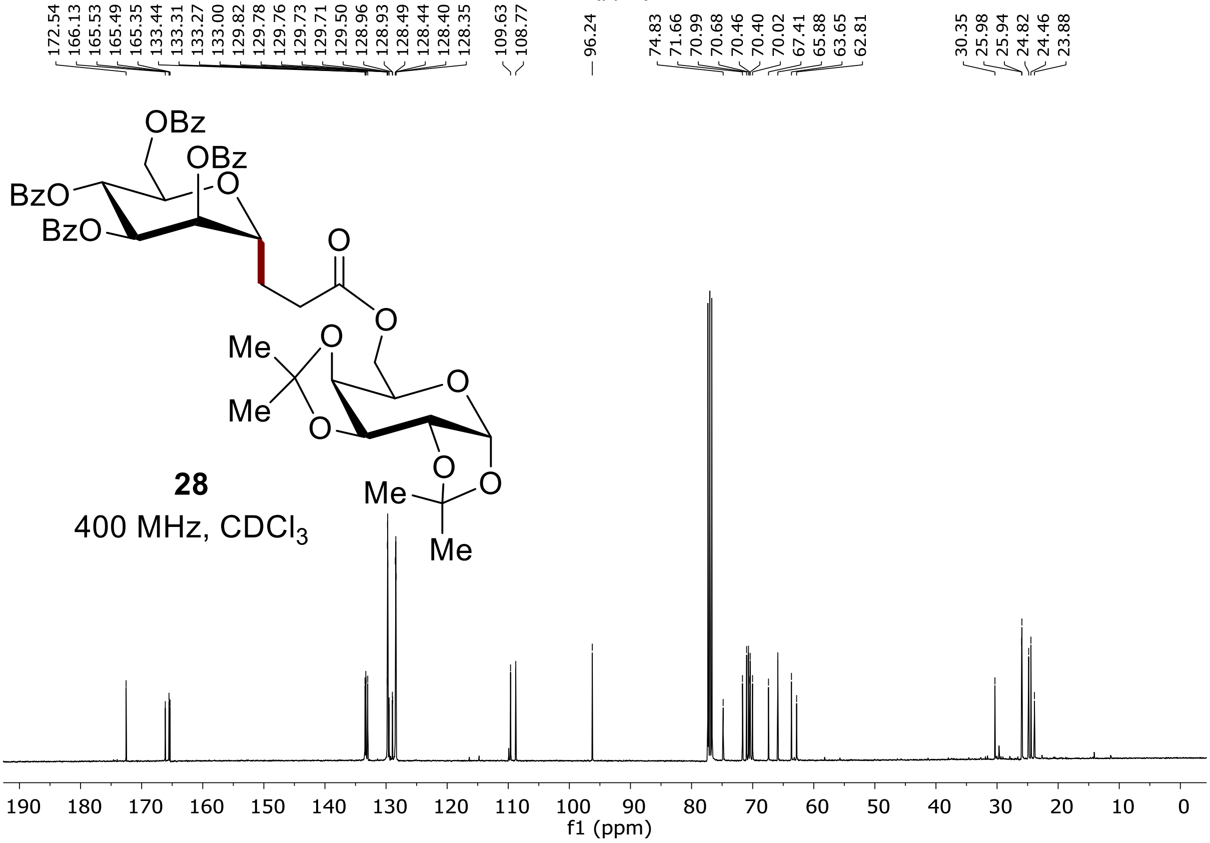
172.54
166.13
165.53
165.49
165.35
133.44
133.31
133.27
133.00
129.82
129.78
129.76
129.73
129.71
129.50
128.96
128.93
128.49
128.44
128.40
128.35
109.63
108.77
96.24
74.83
71.66
70.99
70.68
70.46
70.40
70.02
67.41
65.88
63.65
62.81
30.35
25.98
25.94
24.82
24.46
23.88
OBz
OBz
BzO
BzO
O
O
O
Me
Me
O
O
O
O
Me
Me
28
400 MHz, $CDCl_3$
190 180 170 160 150 140 130 120 110 100 90 80 70 60 50 40 30 20 10 0
f1 (ppm)

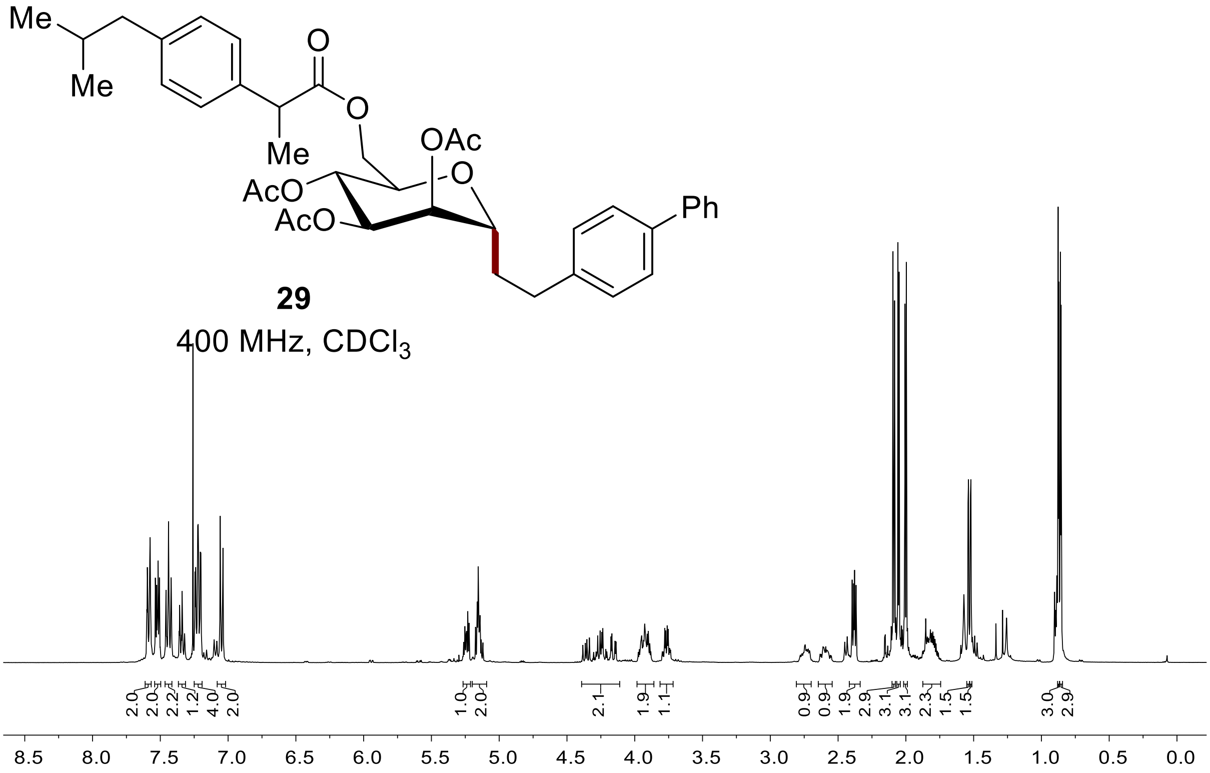
Me
Me
O
O
Me
OAc
O
AcO
AcO
Ph
29
400 MHz, CDCl$_3$
2.0 2.0 2.2 1.2 4.0 2.0 1.0 2.0 2.1 1.9 1.1 0.9 0.9 1.9 2.9 3.1 3.1 2.3 1.5 1.5 3.0 2.9
8.5 8.0 7.5 7.0 6.5 6.0 5.5 5.0 4.5 4.0 3.5 3.0 2.5 2.0 1.5 1.0 0.5 0.0

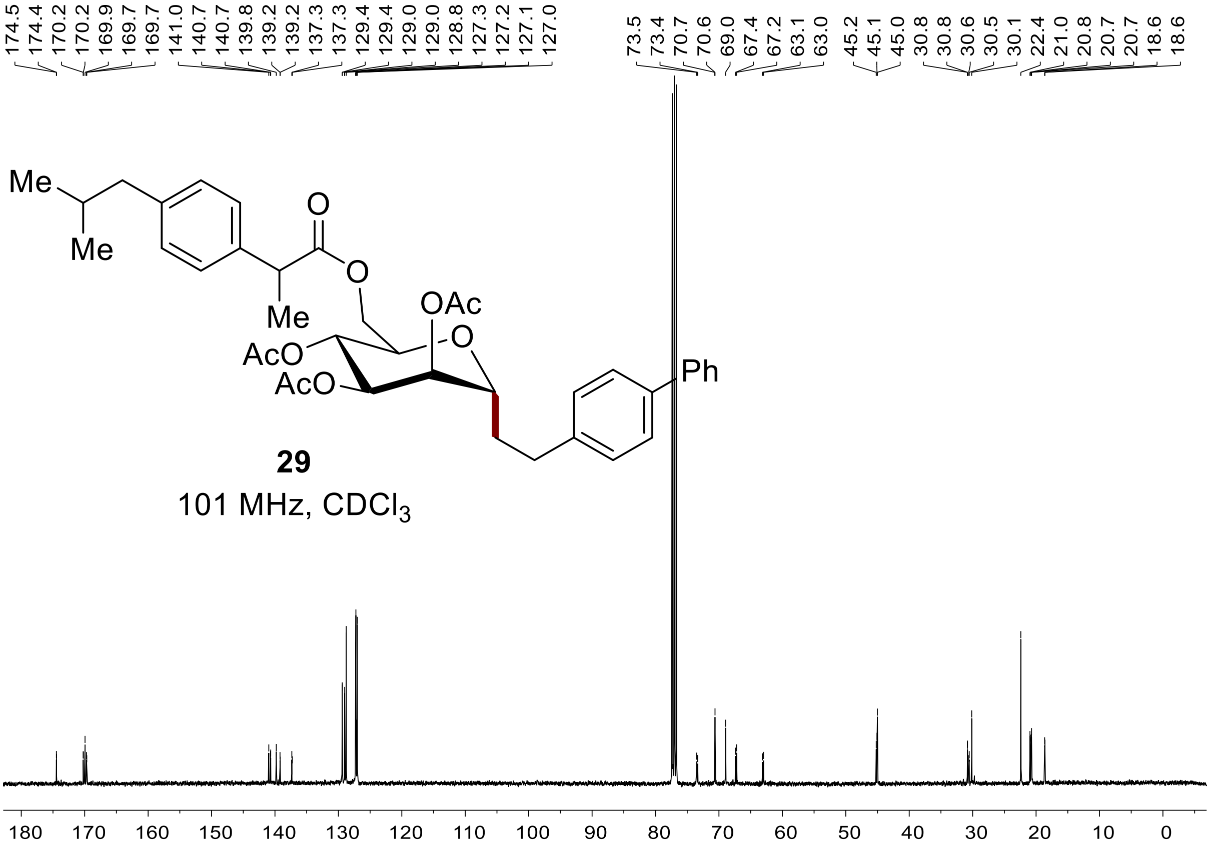
174.5 174.4 170.2 170.2 169.9 169.7 169.7 141.0 140.7 140.7 139.8 139.2 139.2 137.3 137.3 129.4 129.4 129.0 129.0 128.8 127.3 127.2 127.1 127.0
73.5 73.4 70.7 70.6 69.0 67.4 67.2 63.1 63.0 45.2 45.1 45.0 30.8 30.8 30.6 30.5 30.1 22.4 21.0 20.8 20.7 20.7 18.6 18.6
Me
Me
O
O
Me
OAc
O
AcO
AcO
Ph
29
101 MHz, CDCl$_3$
180 170 160 150 140 130 120 110 100 90 80 70 60 50 40 30 20 10 0

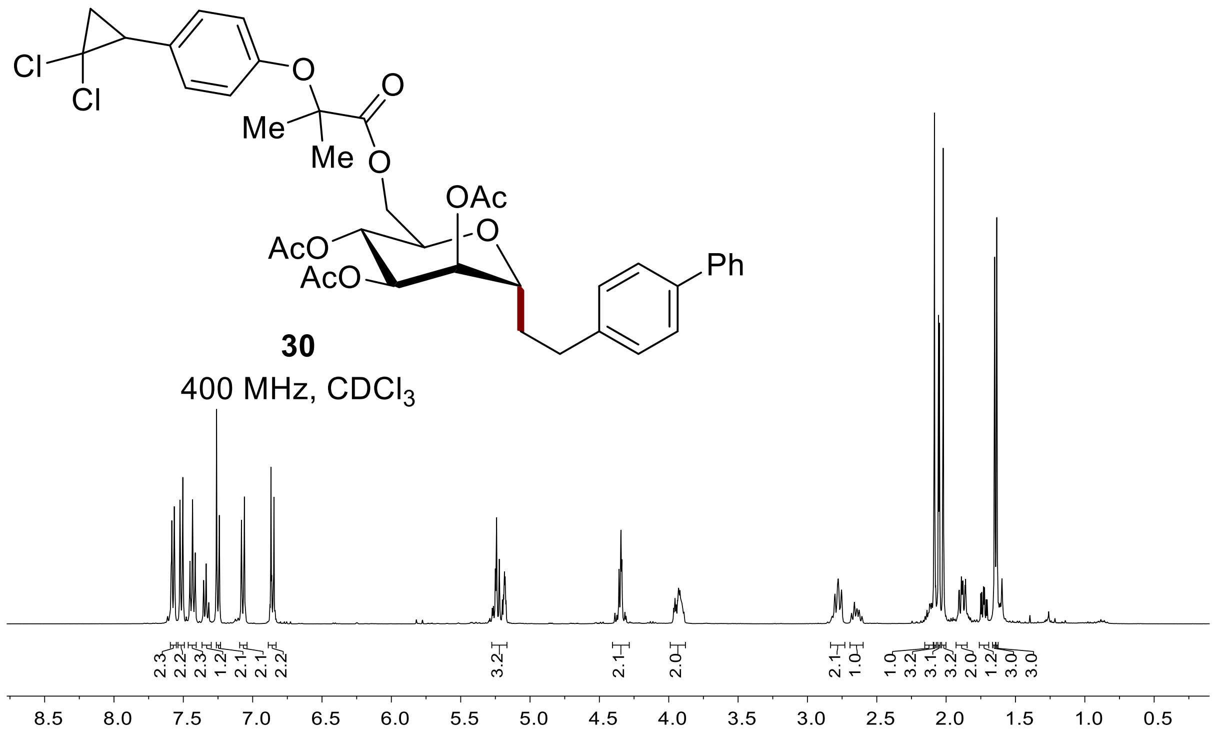

Cl
Cl
O
O
Me
Me
O
OAc
O
AcO
AcO
Ph
30
400 MHz, $CDCl_3$
2.3 2.2 2.3 1.2 2.1 2.1 2.2
3.2
2.1
2.0
2.1 1.0
1.0 3.2 3.1 3.2 2.0 1.2 3.0 3.0
8.5 8.0 7.5 7.0 6.5 6.0 5.5 5.0 4.5 4.0 3.5 3.0 2.5 2.0 1.5 1.0 0.5

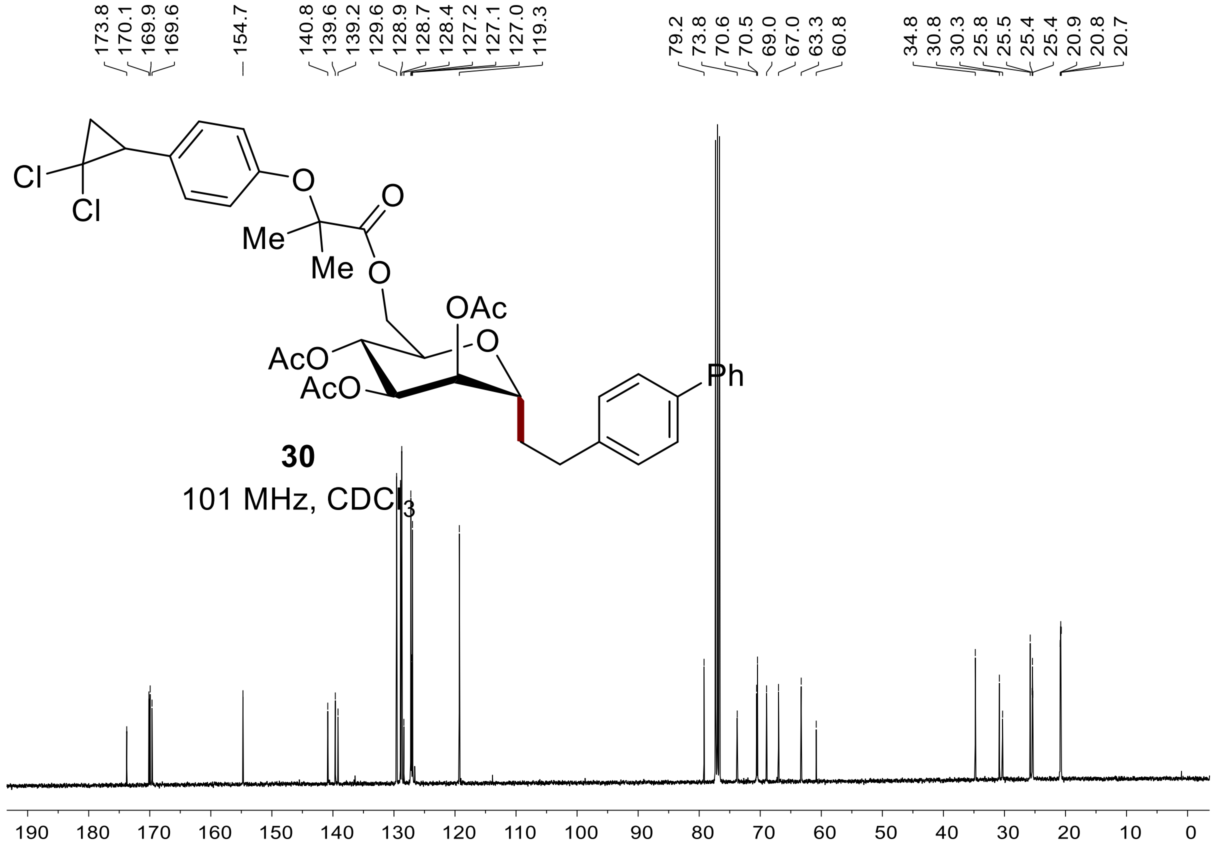

173.8 170.1 169.9 169.6
154.7
140.8 139.6 139.2 129.6 128.9 128.7 128.4 127.2 127.1 127.0 119.3
79.2 73.8 70.6 70.5 69.0 67.0 63.3 60.8
34.8 30.8 30.3 25.8 25.5 25.4 25.4 20.9 20.8 20.7
Cl
Cl
O
O
Me
Me
O
OAc
O
AcO
AcO
Ph
30
101 MHz, $CDCl_3$
190 180 170 160 150 140 130 120 110 100 90 80 70 60 50 40 30 20 10 0

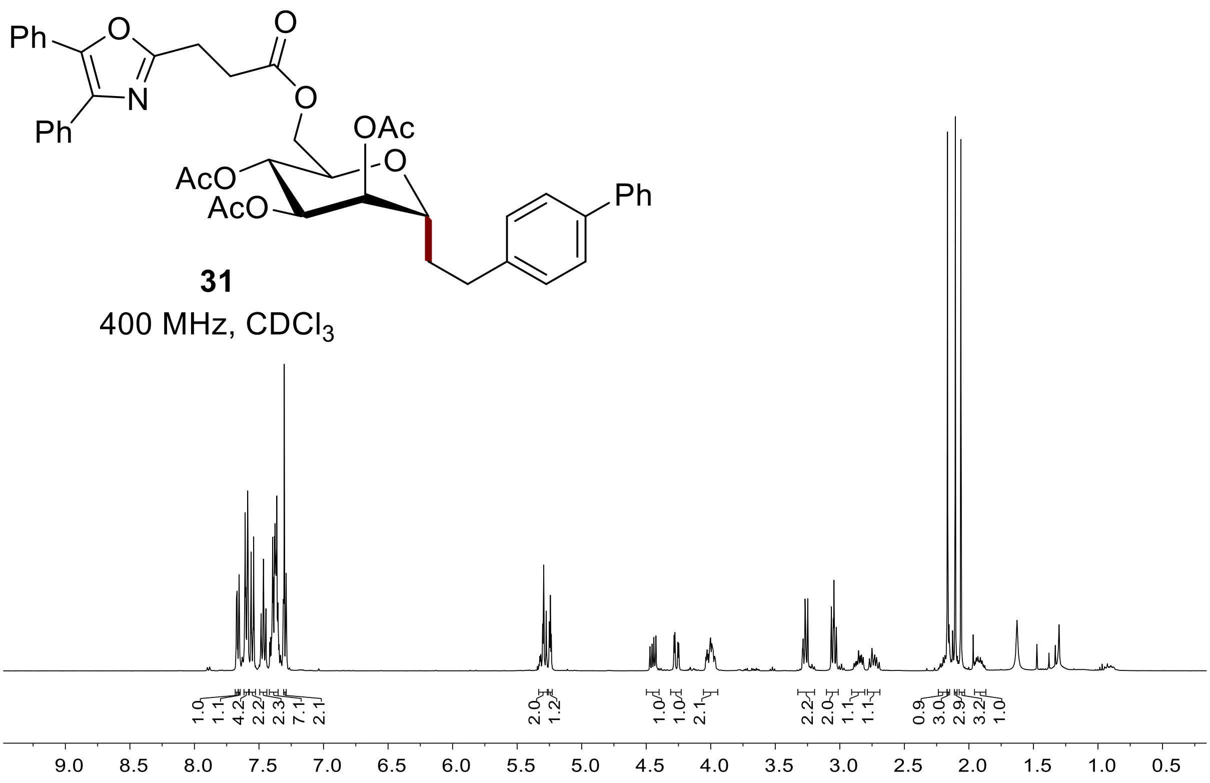
Ph
O
O
N
Ph
O
OAc
AcO
O
AcO
Ph
31
400 MHz, CDCl$_3$
1.0
1.1
4.2
2.2
2.3
7.1
2.1
2.0
1.2
1.0
1.0
2.1
2.2
2.0
1.1
1.1
0.9
3.0
2.9
3.2
1.0
9.0
8.5
8.0
7.5
7.0
6.5
6.0
5.5
5.0
4.5
4.0
3.5
3.0
2.5
2.0
1.5
1.0
0.5

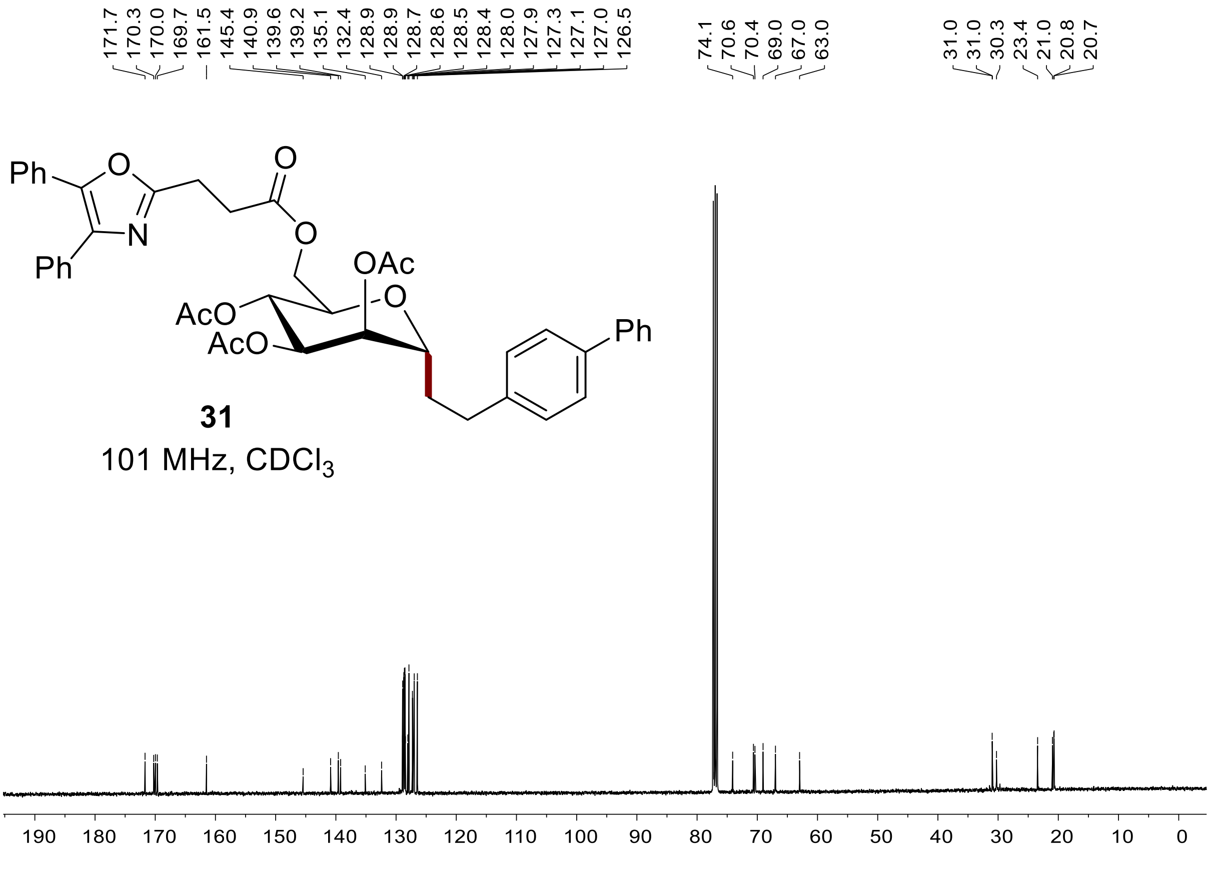
171.7
170.3
170.0
169.7
161.5
145.4
140.9
139.6
139.2
135.1
132.4
128.9
128.9
128.7
128.6
128.5
128.4
128.0
127.9
127.3
127.1
127.0
126.5
74.1
70.6
70.4
69.0
67.0
63.0
31.0
31.0
30.3
23.4
21.0
20.8
20.7
Ph
O
O
N
Ph
O
OAc
AcO
O
AcO
Ph
31
101 MHz, CDCl$_3$
190
180
170
160
150
140
130
120
110
100
90
80
70
60
50
40
30
20
10
0

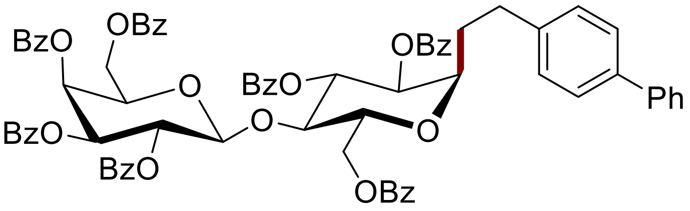

32

400 MHz, $CDCl_3$

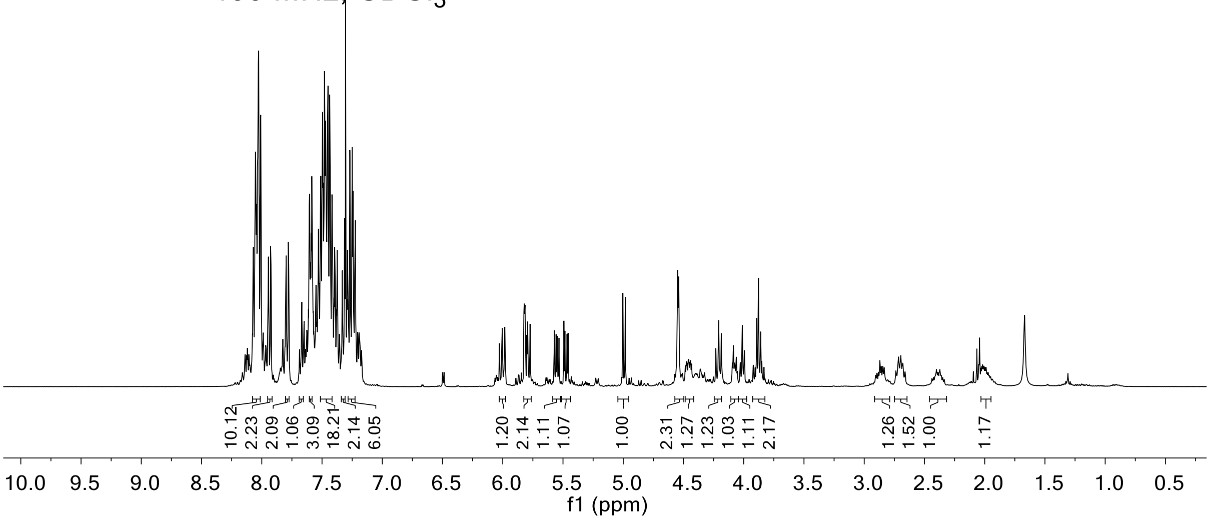

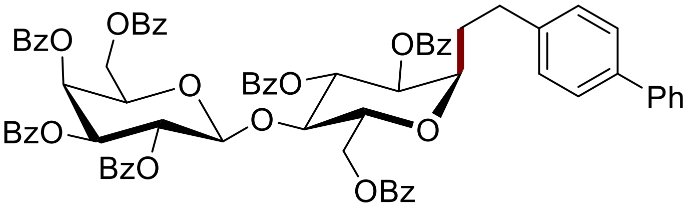

32

101 MHz, $CDCl_3$

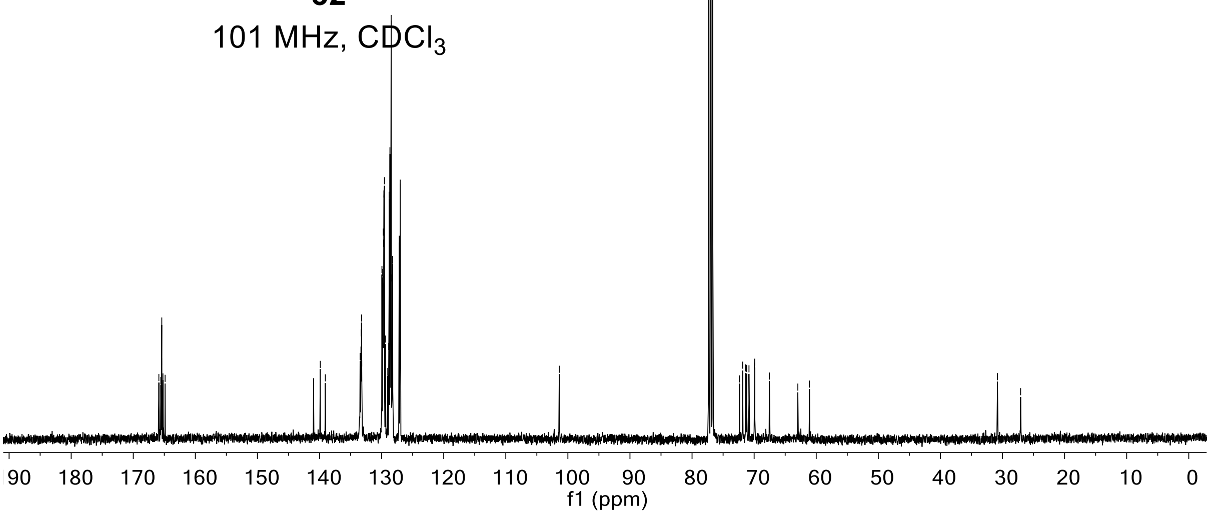

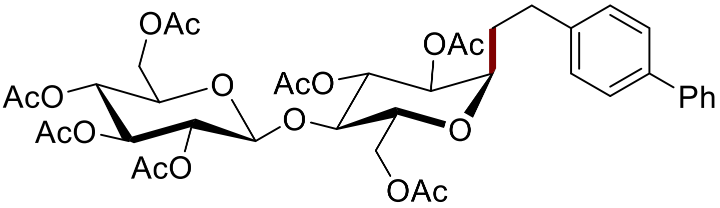

33
400 MHz, $CDCl_3$

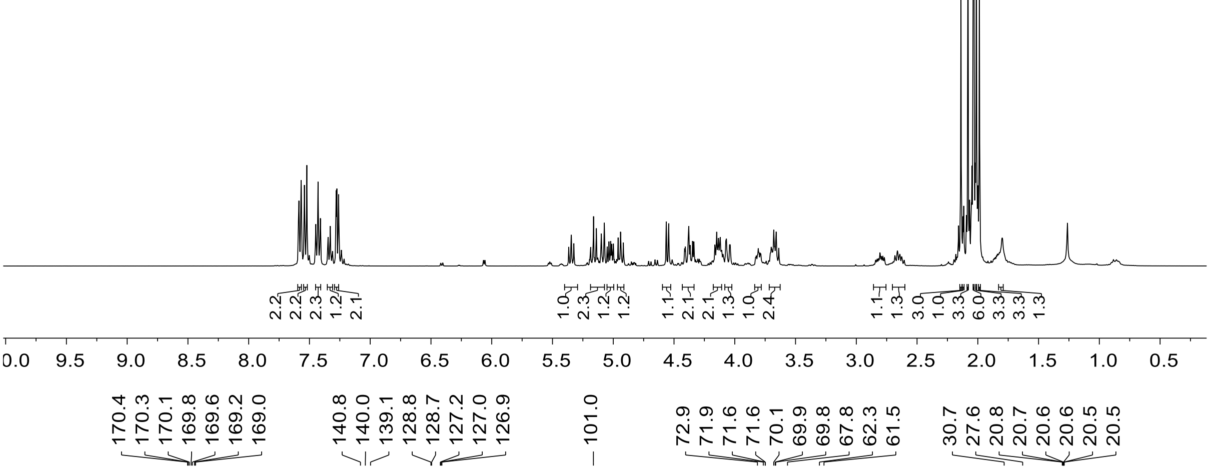

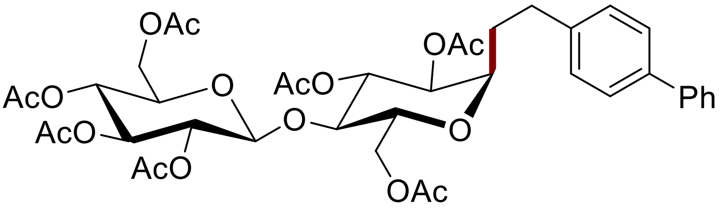

33
101 MHz, $CDCl_3$

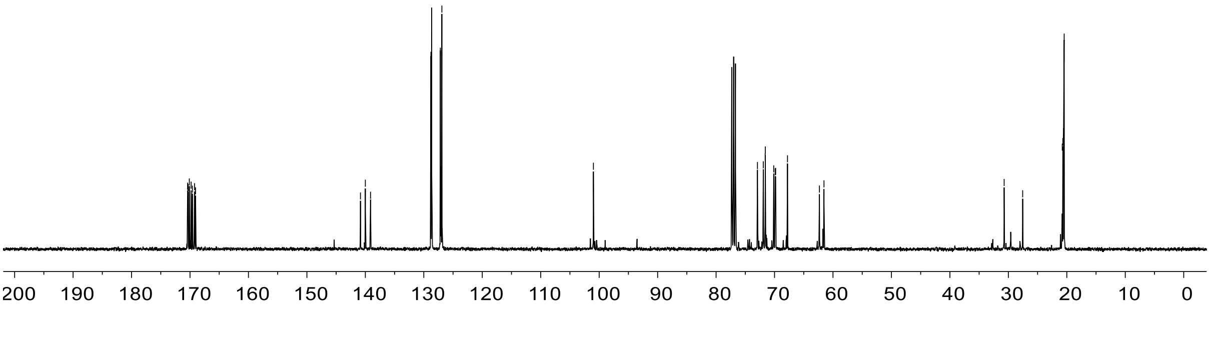

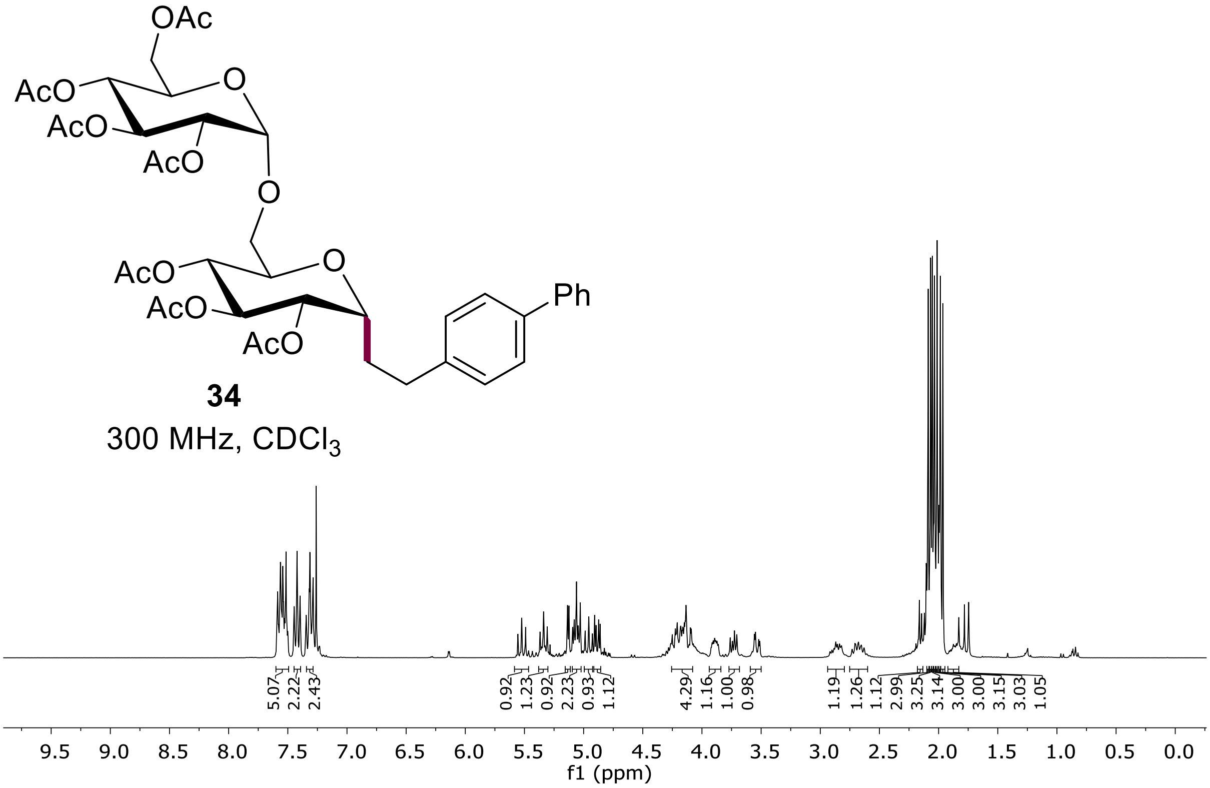
OAc
AcO
AcO
AcO
O
O
AcO
AcO
AcO
O
Ph
34
300 MHz, CDCl3
5.07
2.22
2.43
0.92
1.23
0.92
2.23
0.93
1.12
4.29
1.16
1.00
0.98
1.19
1.26
1.12
2.99
3.25
3.14
3.00
3.00
3.15
3.03
1.05
9.5 9.0 8.5 8.0 7.5 7.0 6.5 6.0 5.5 5.0 4.5 4.0 3.5 3.0 2.5 2.0 1.5 1.0 0.5 0.0
f1 (ppm)

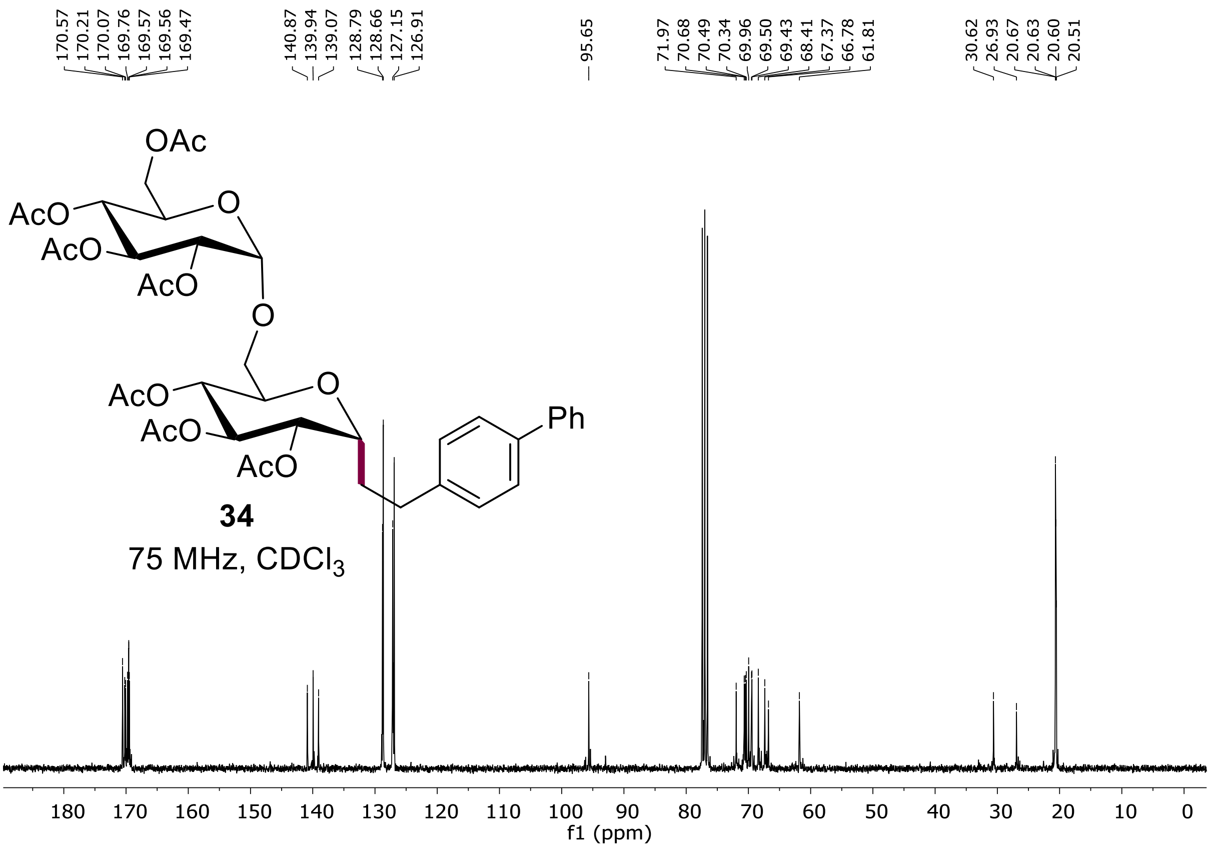
170.57
170.21
170.07
169.76
169.57
169.56
169.47
140.87
139.94
139.07
128.79
128.66
127.15
126.91
95.65
71.97
70.68
70.49
70.34
69.96
69.50
69.43
68.41
67.37
66.78
61.81
30.62
26.93
20.67
20.63
20.60
20.51
OAc
AcO
AcO
AcO
O
O
AcO
AcO
AcO
O
Ph
34
75 MHz, CDCl3
180 170 160 150 140 130 120 110 100 90 80 70 60 50 40 30 20 10 0
f1 (ppm)

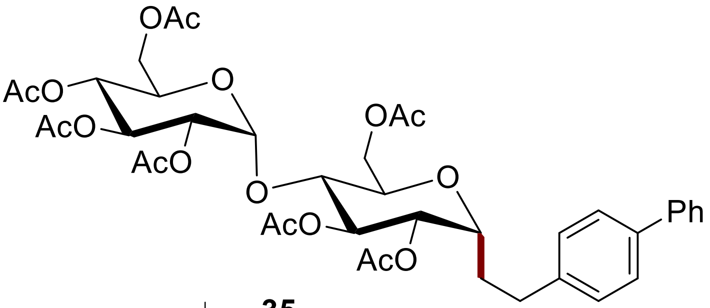

35

400 MHz, $CDCl_3$

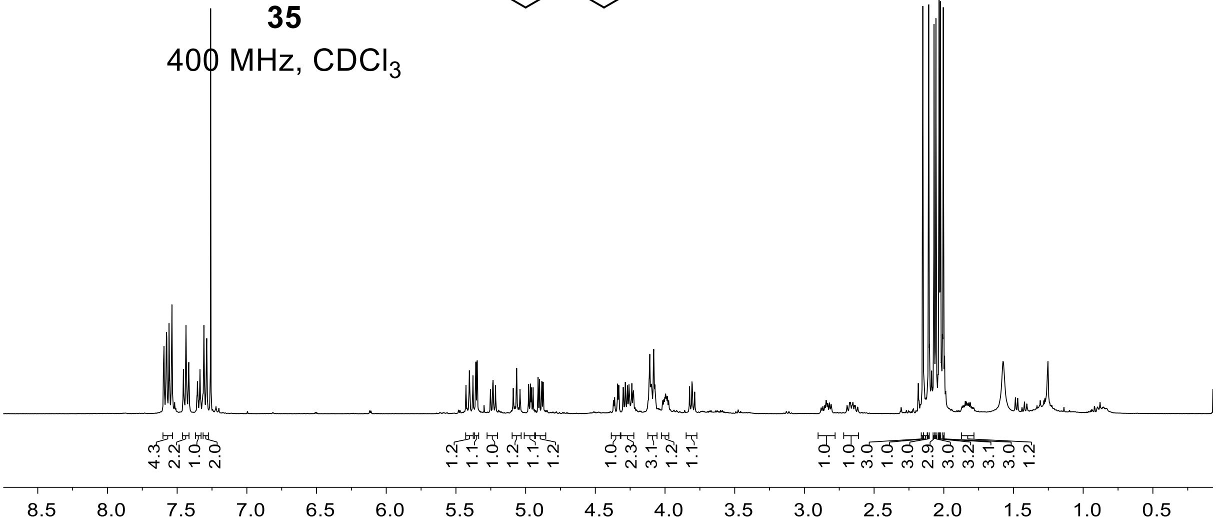

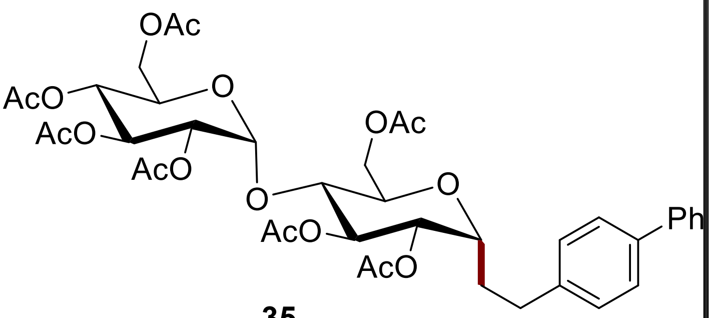

35

101 MHz, $CDCl_3$

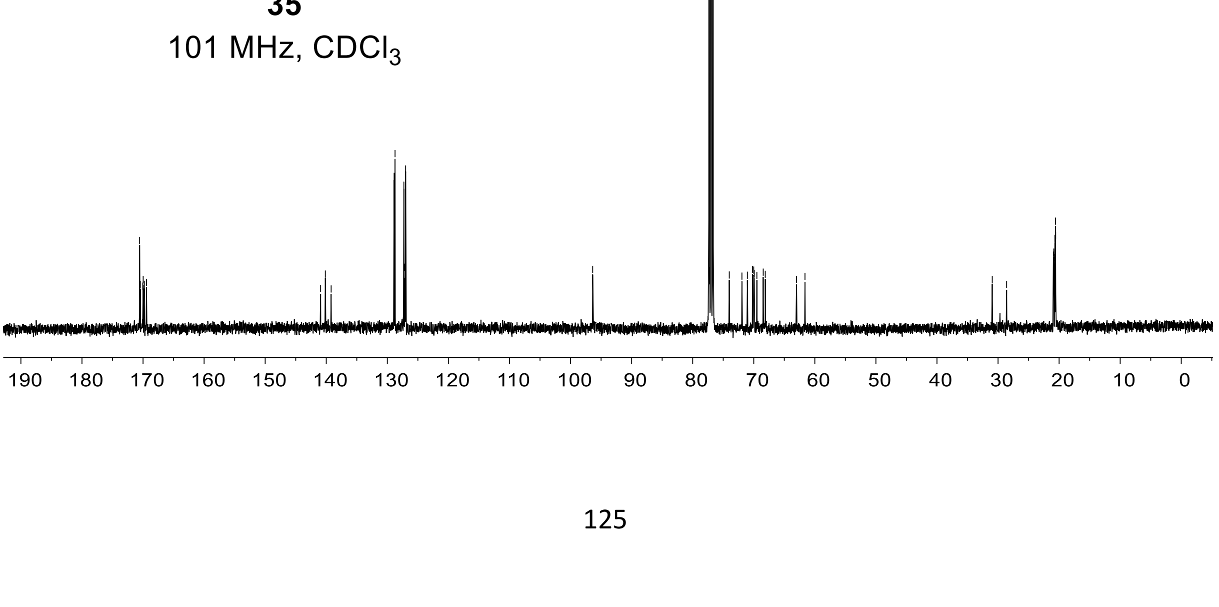

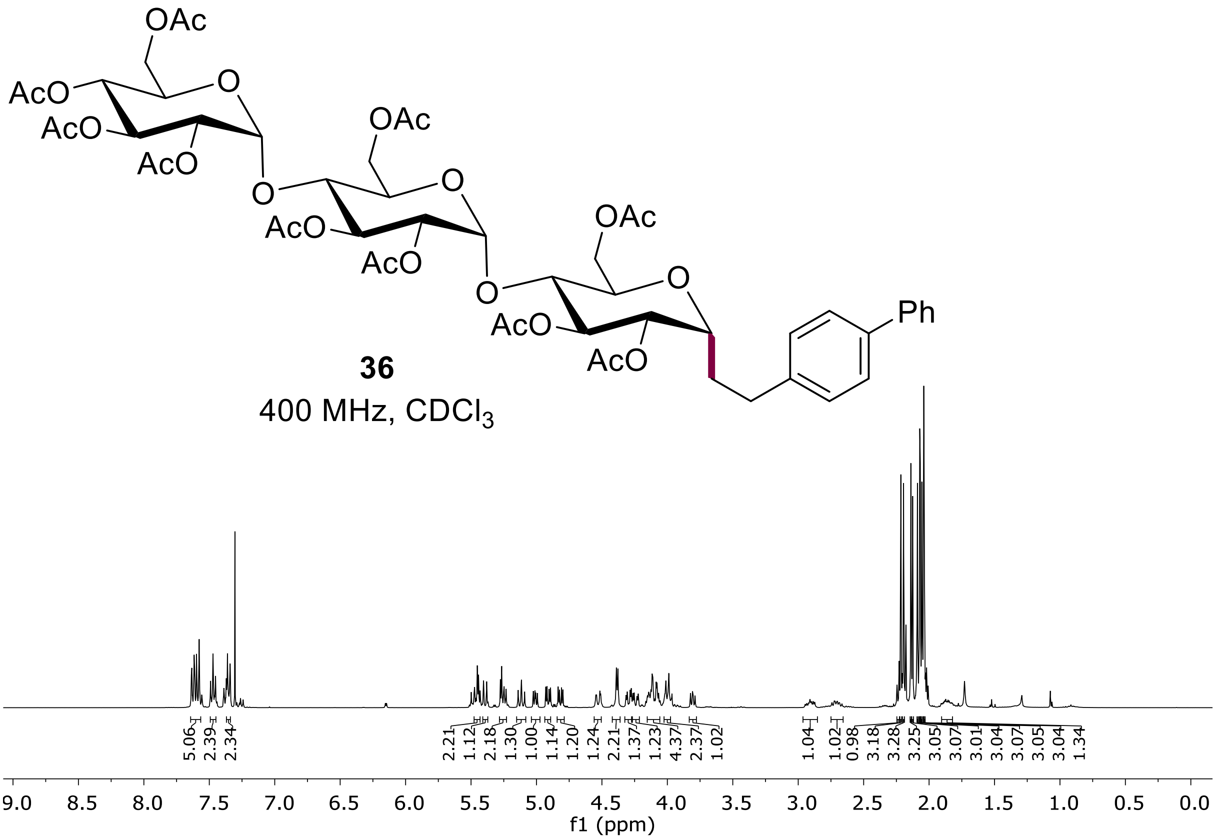
OAc
AcO
AcO
AcO
O
O
OAc
AcO
AcO
O
O
OAc
AcO
AcO
O
Ph
36
400 MHz, CDCl3
5.06
2.39
2.34
2.21
1.12
2.18
1.30
1.00
1.14
1.20
1.24
2.21
1.37
1.23
4.37
2.37
1.02
1.04
1.02
0.98
3.18
3.28
3.25
3.05
3.07
3.01
3.04
3.07
3.05
3.04
1.34
9.0 8.5 8.0 7.5 7.0 6.5 6.0 5.5 5.0 4.5 4.0 3.5 3.0 2.5 2.0 1.5 1.0 0.5 0.0
f1 (ppm)

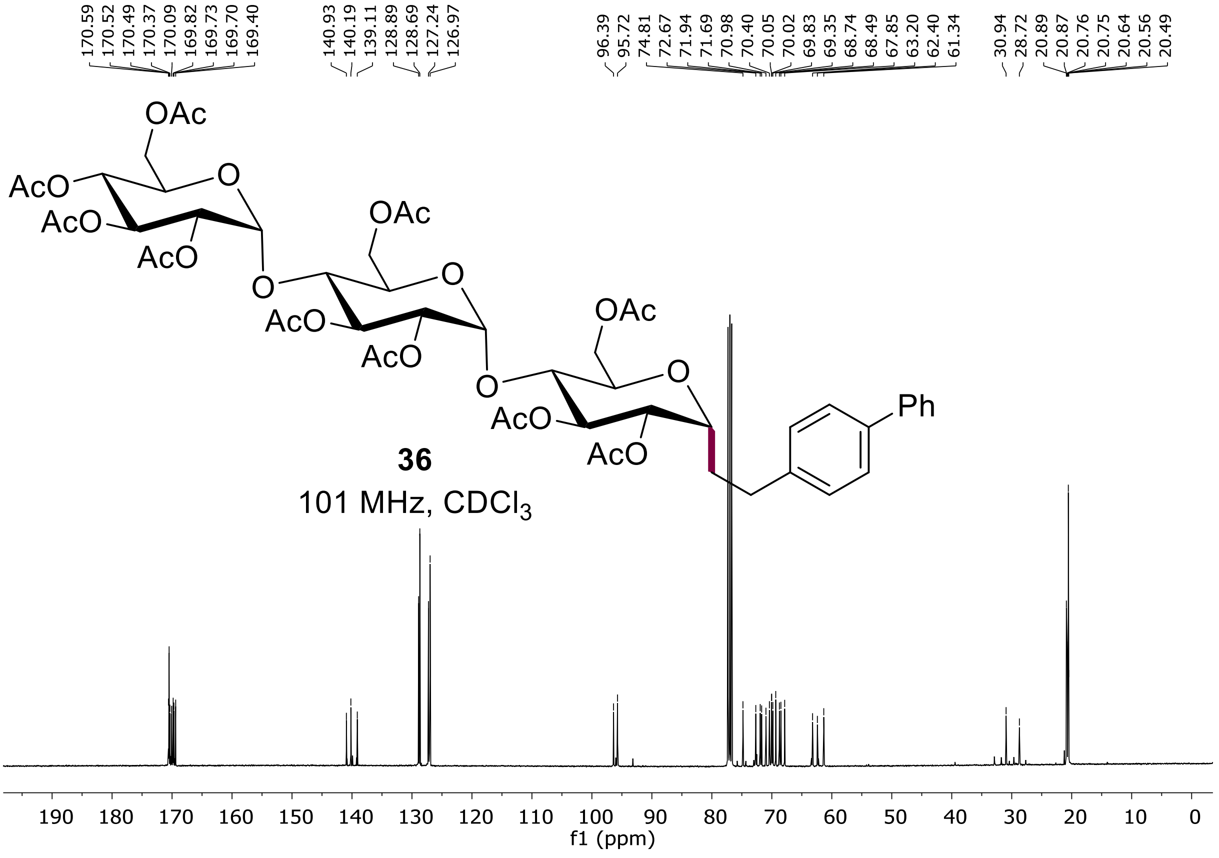
170.59
170.52
170.49
170.37
170.09
169.82
169.73
169.70
169.40
140.93
140.19
139.11
128.89
128.69
127.24
126.97
96.39
95.72
74.81
72.67
71.94
71.69
70.98
70.40
70.05
70.02
69.83
69.35
68.74
68.49
67.85
63.20
62.40
61.34
30.94
28.72
20.89
20.87
20.76
20.75
20.64
20.56
20.49
OAc
AcO
AcO
AcO
O
O
OAc
AcO
AcO
O
O
OAc
AcO
AcO
O
Ph
36
101 MHz, CDCl3
190 180 170 160 150 140 130 120 110 100 90 80 70 60 50 40 30 20 10 0
f1 (ppm)

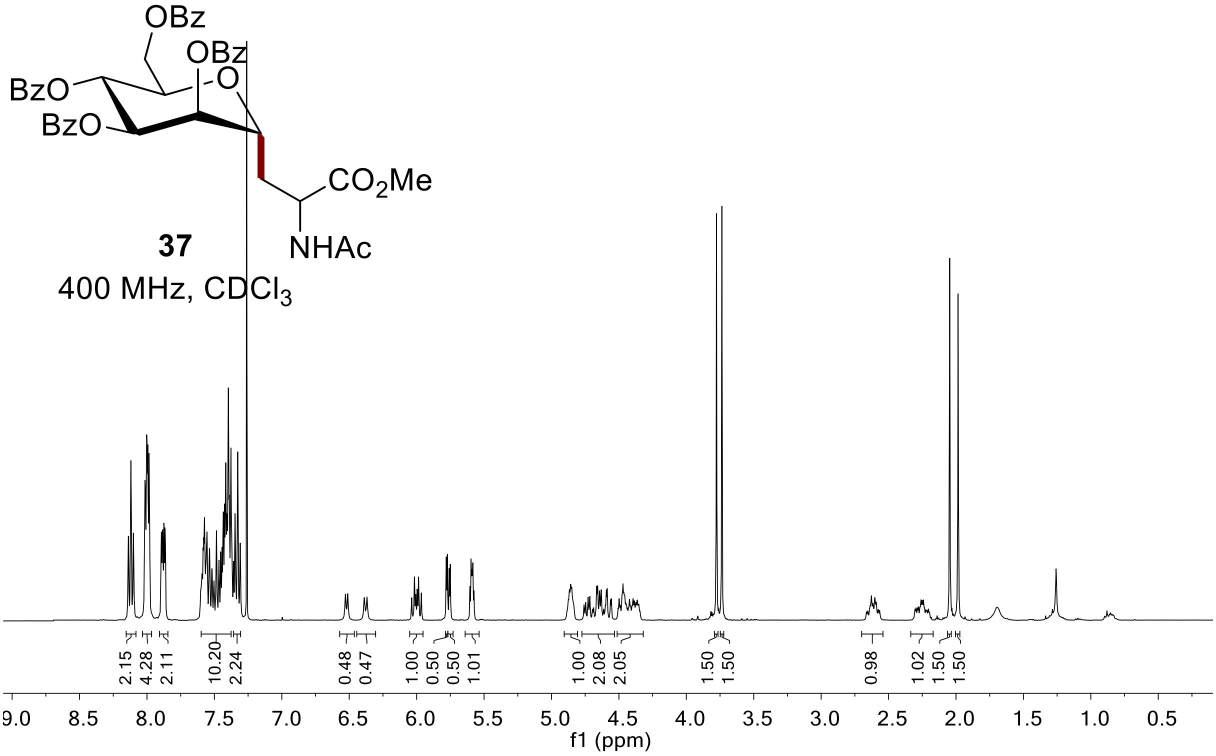
OBz
OBz
BzO
BzO
O
CO2Me
NHAc
37
400 MHz, CDCl3
f1 (ppm)

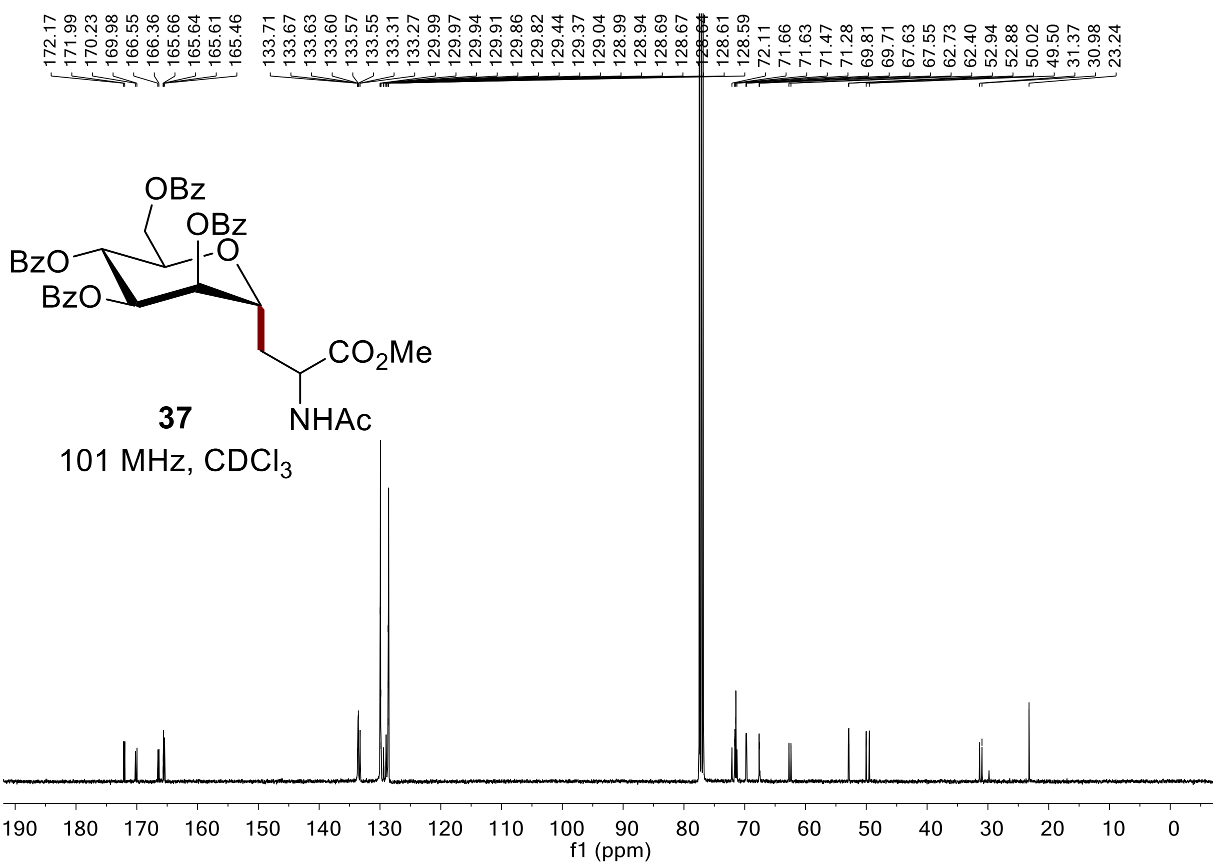
OBz
OBz
BzO
BzO
O
CO2Me
NHAc
37
101 MHz, CDCl3
f1 (ppm)

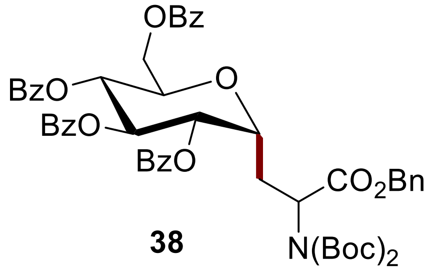

38

400 MHz, $CDCl_3$

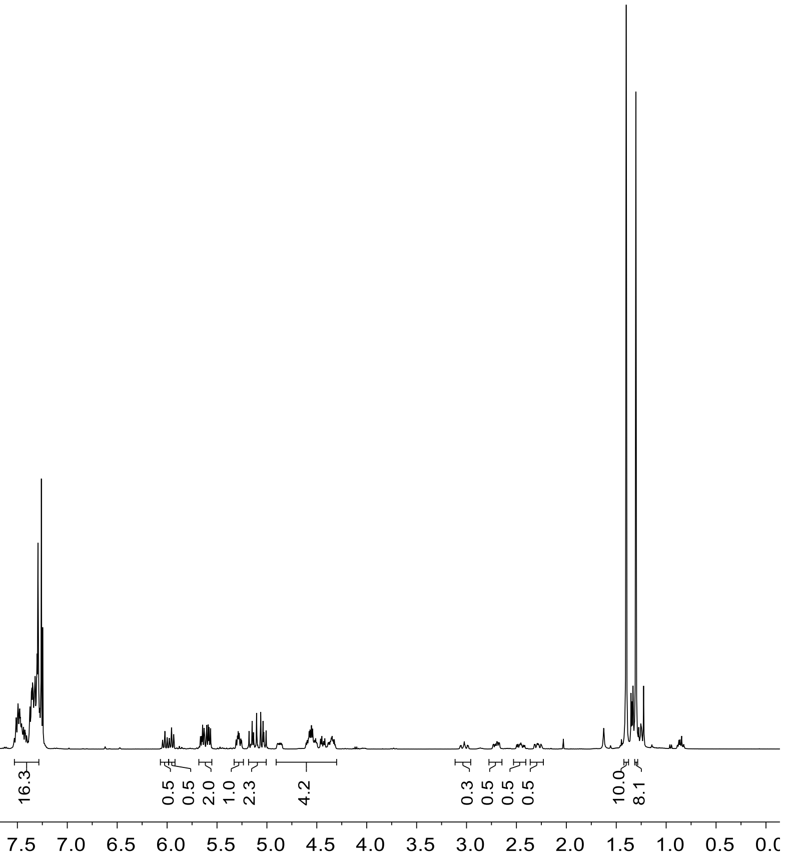

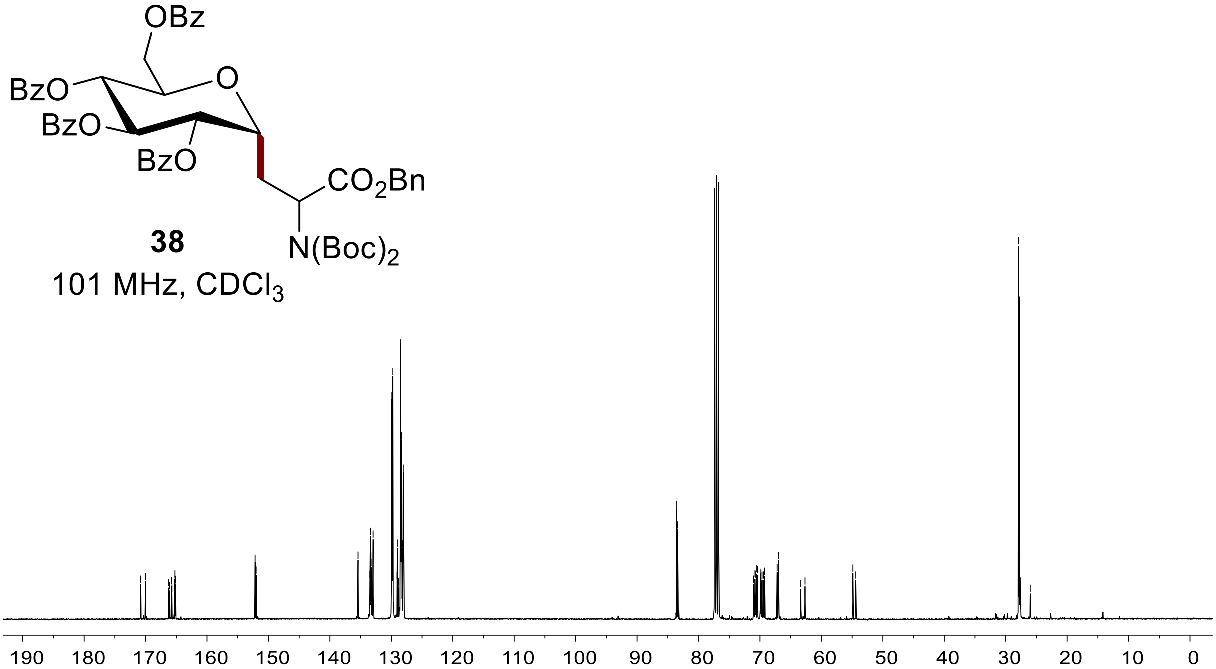

38

101 MHz, $CDCl_3$

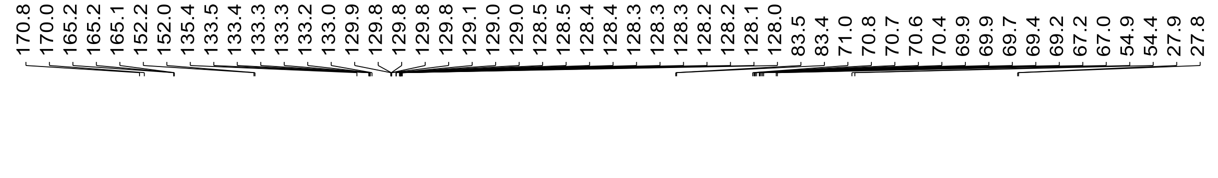

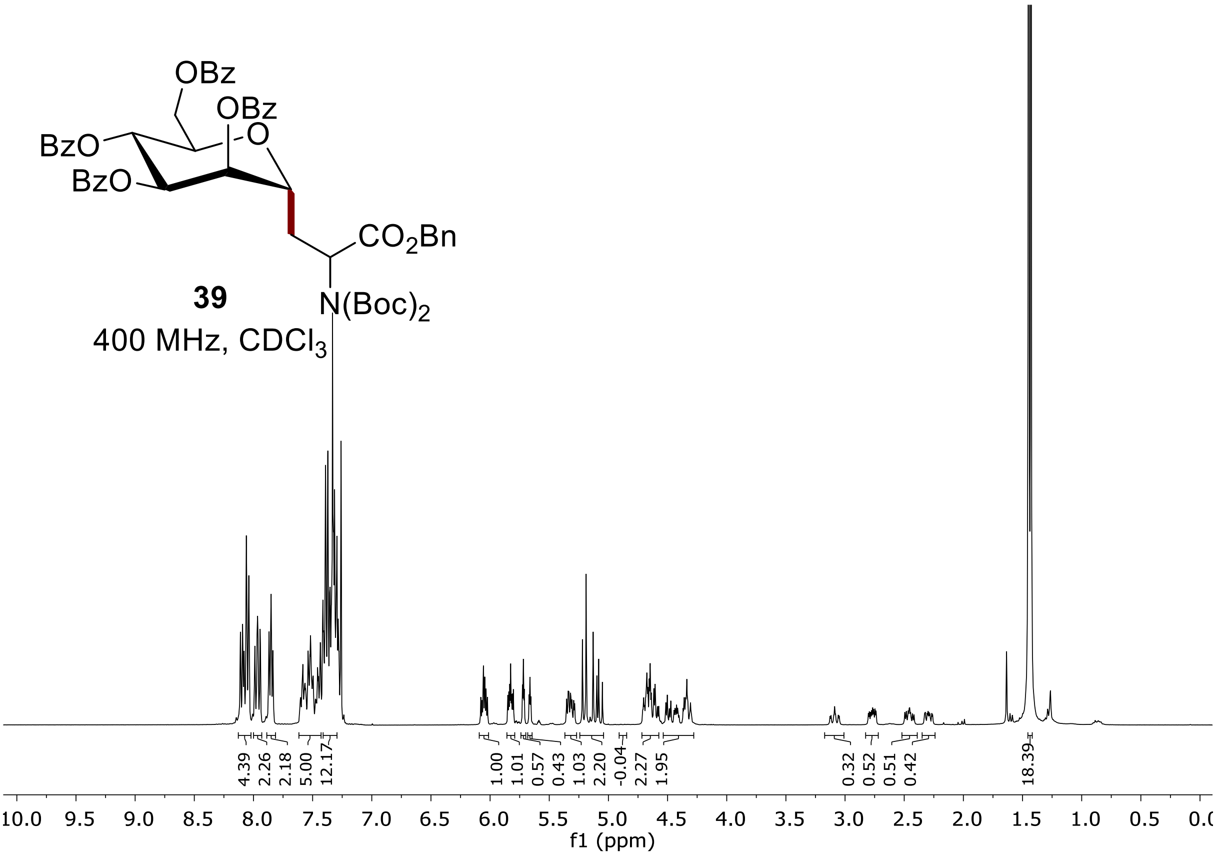
OBz
OBz
O
BzO
BzO
CO2Bn
39
N(Boc)2
400 MHz, CDCl3
4.39 2.26 2.18 5.00 12.17 1.00 1.01 0.57 0.43 1.03 2.20 -0.04 2.27 1.95 0.32 0.52 0.51 0.42 18.39
f1 (ppm)

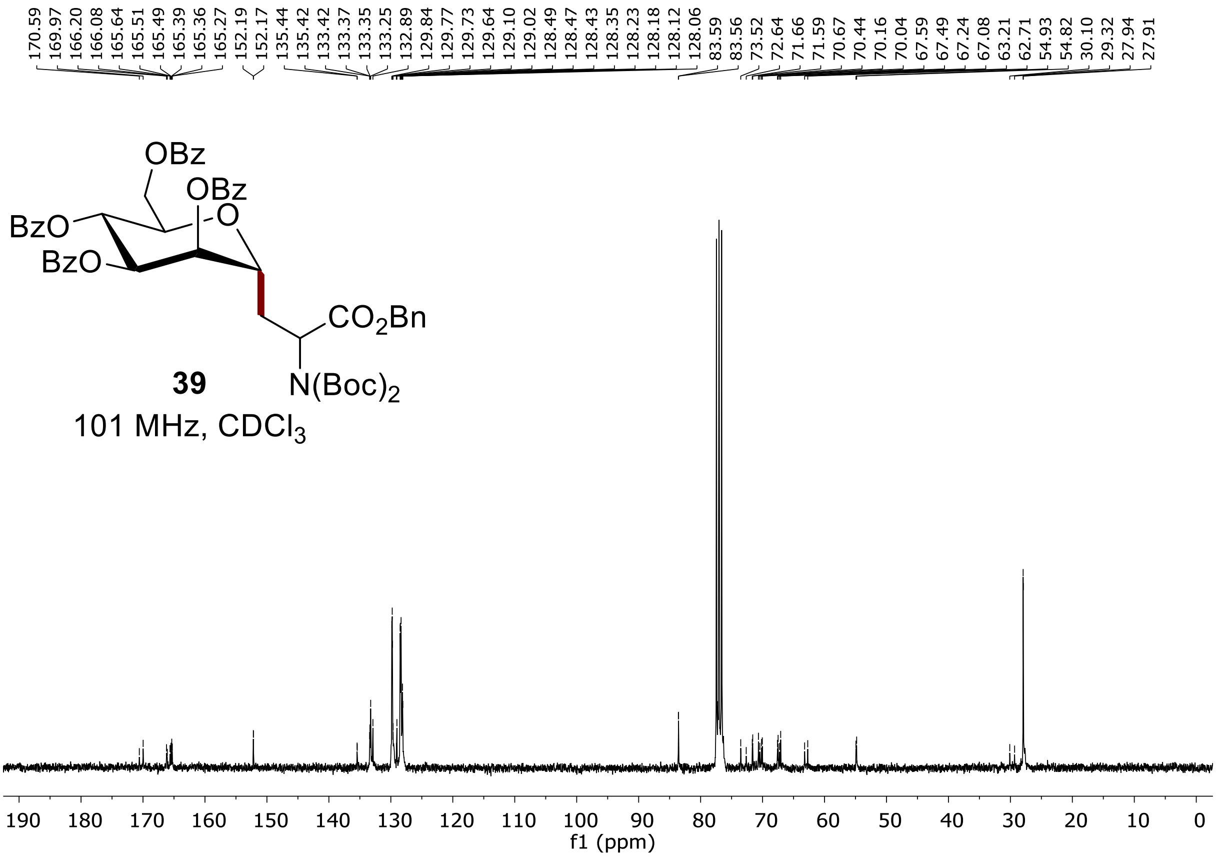
170.59 169.97 166.20 166.08 165.64 165.51 165.49 165.39 165.36 165.27 152.19 152.17 135.44 135.42 133.42 133.37 133.35 133.25 132.89 129.84 129.77 129.73 129.64 129.10 129.02 128.49 128.47 128.43 128.35 128.23 128.18 128.12 128.06 83.59 83.56 73.52 72.64 71.66 71.59 70.67 70.44 70.16 70.04 67.59 67.49 67.24 67.08 63.21 62.71 54.93 54.82 30.10 29.32 27.94 27.91
OBz
OBz
O
BzO
BzO
CO2Bn
39
N(Boc)2
101 MHz, CDCl3
f1 (ppm)

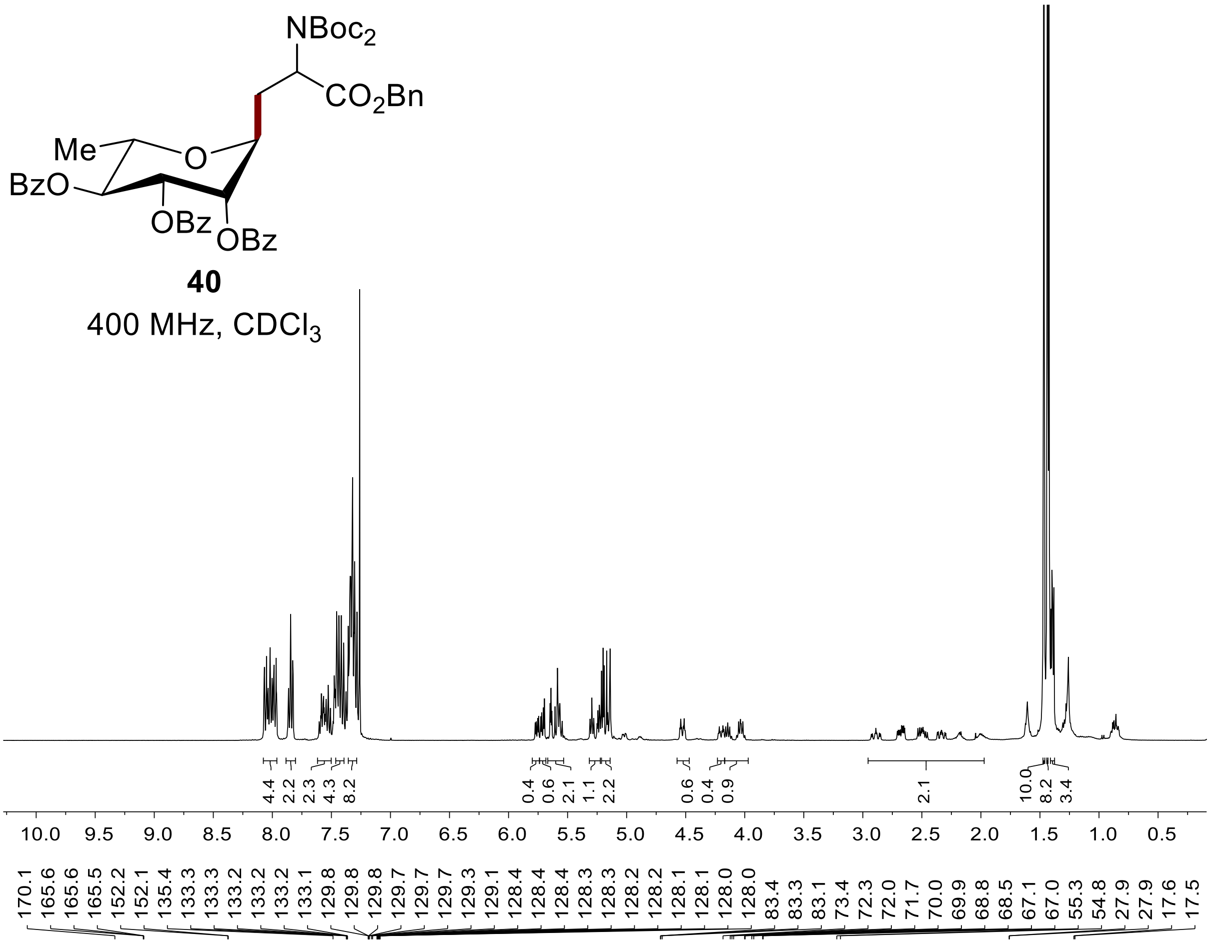

40

400 MHz, $CDCl_3$

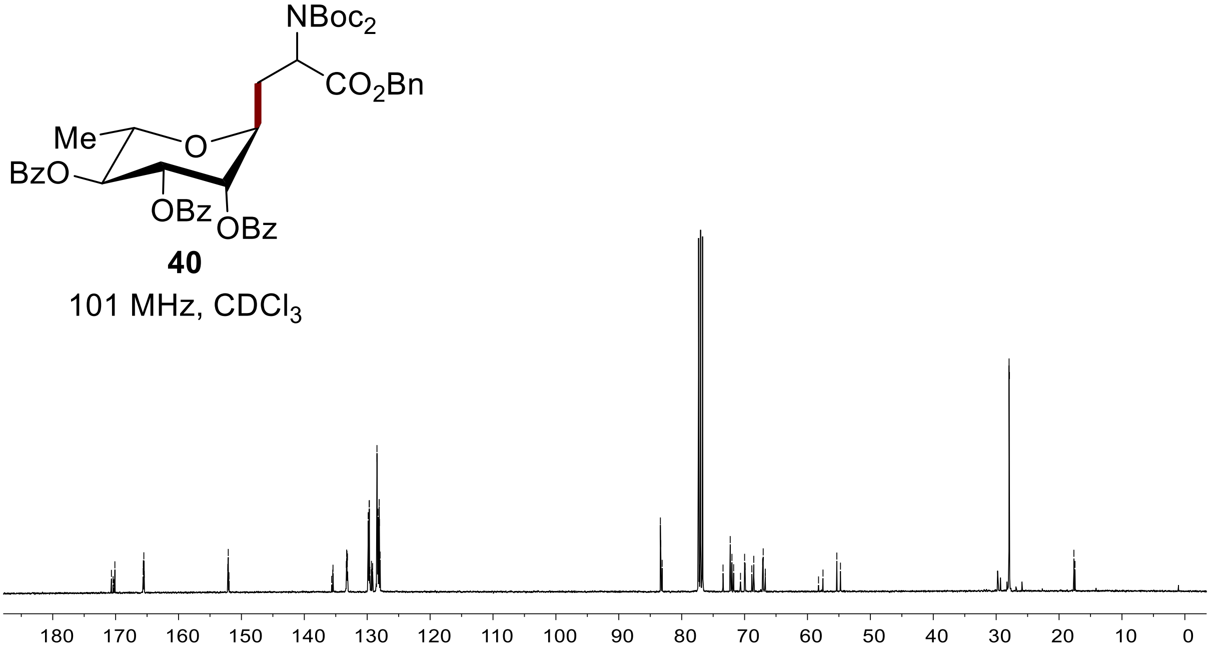

40

101 MHz, $CDCl_3$

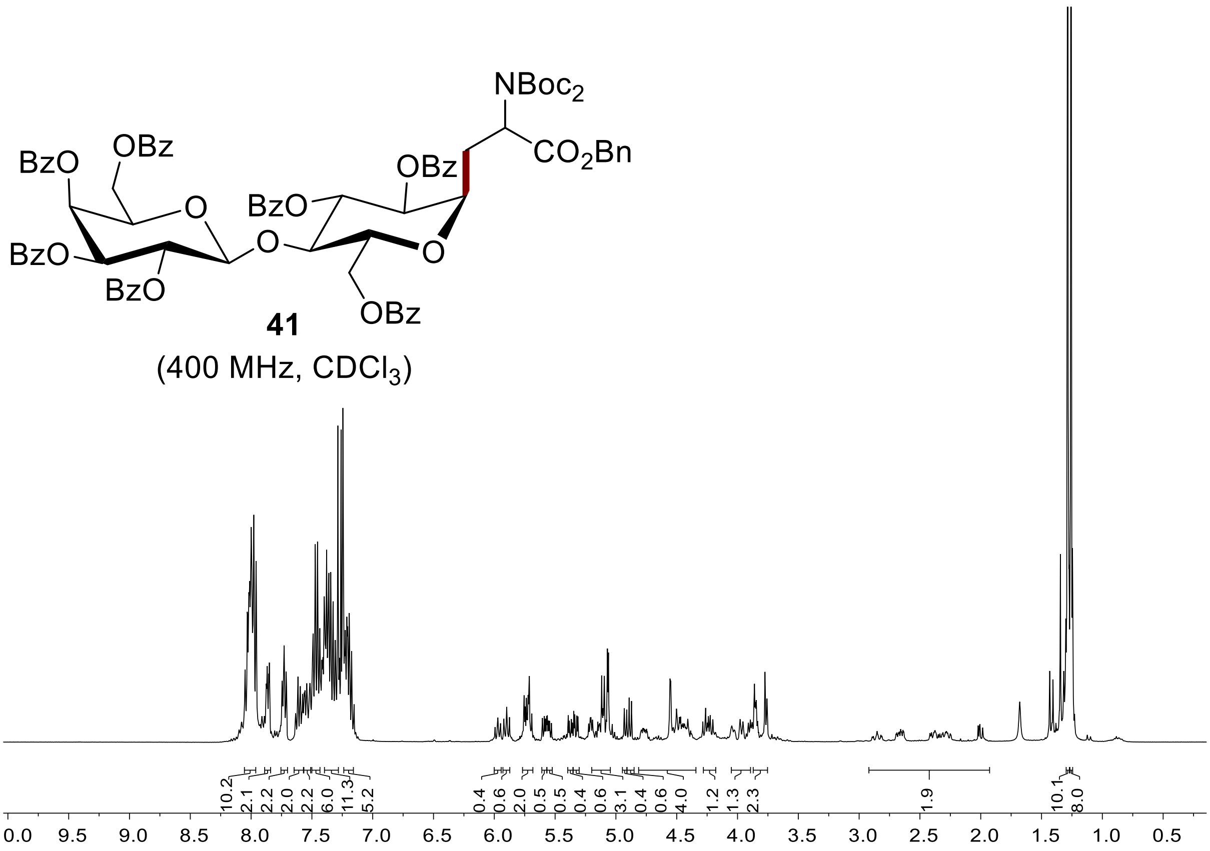
NBoc2
CO2Bn
BzO
OBz
BzO
BzO
BzO
OBz
OBz
O
O
O
41
(400 MHz, CDCl3)
10.2
2.1
2.2
2.0
2.2
6.0
11.3
5.2
0.4
0.6
2.0
0.5
0.5
0.4
0.6
3.1
0.4
0.6
4.0
1.2
1.3
2.3
1.9
10.1
8.0
0.0 9.5 9.0 8.5 8.0 7.5 7.0 6.5 6.0 5.5 5.0 4.5 4.0 3.5 3.0 2.5 2.0 1.5 1.0 0.5

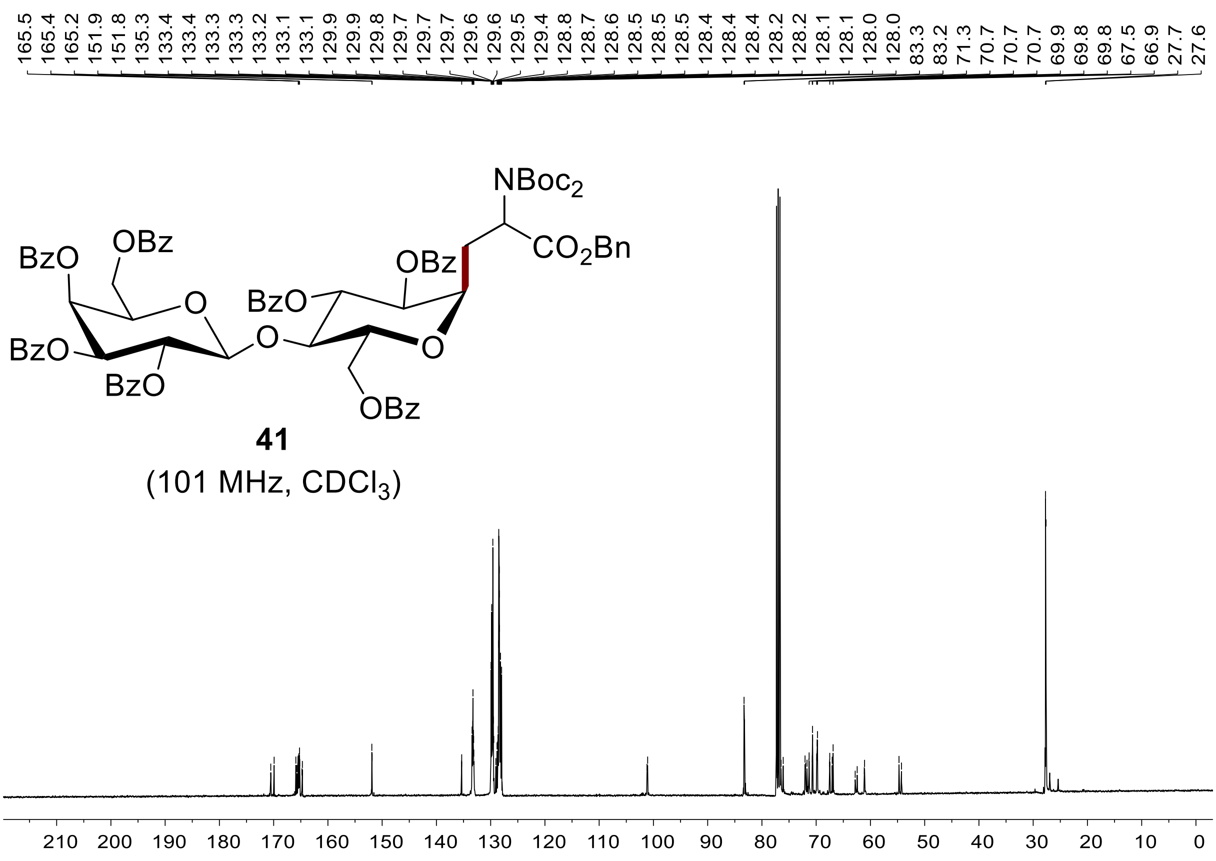
165.5
165.4
165.2
151.9
151.8
135.3
133.4
133.4
133.3
133.3
133.2
133.1
133.1
129.9
129.9
129.8
129.7
129.7
129.7
129.6
129.6
129.5
129.4
128.8
128.7
128.6
128.5
128.5
128.5
128.4
128.4
128.4
128.2
128.2
128.1
128.1
128.0
128.0
83.3
83.2
71.3
70.7
70.7
70.7
69.9
69.8
69.8
67.5
66.9
27.7
27.6
NBoc2
CO2Bn
BzO
OBz
BzO
BzO
BzO
OBz
OBz
O
O
O
41
(101 MHz, CDCl3)
210 200 190 180 170 160 150 140 130 120 110 100 90 80 70 60 50 40 30 20 10 0

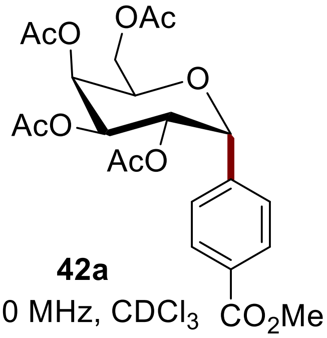

42a
400 MHz, $CDCl_3$

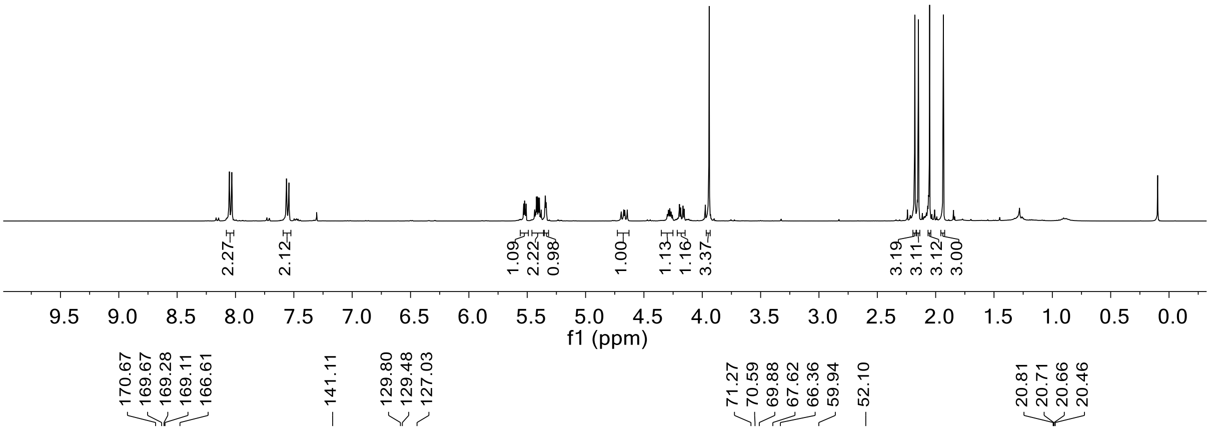

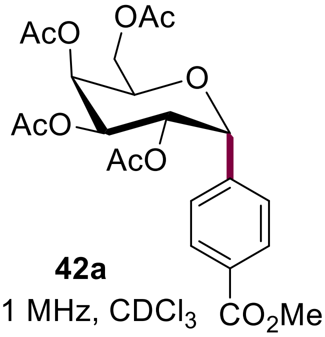

42a
101 MHz, $CDCl_3$

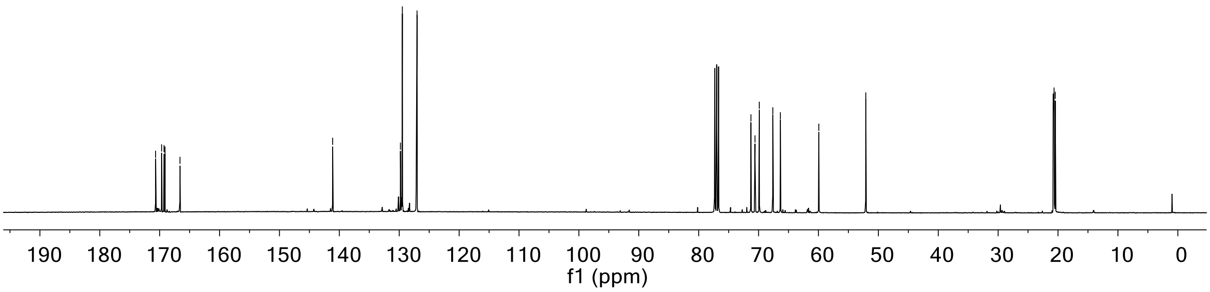

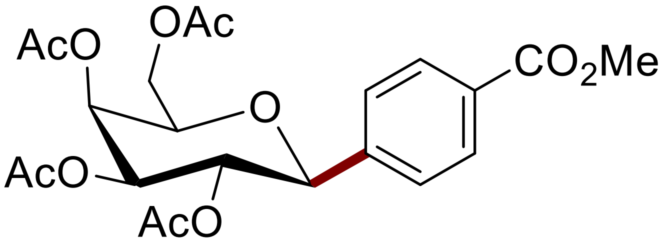

42ba

400 MHz, $CDCl_3$

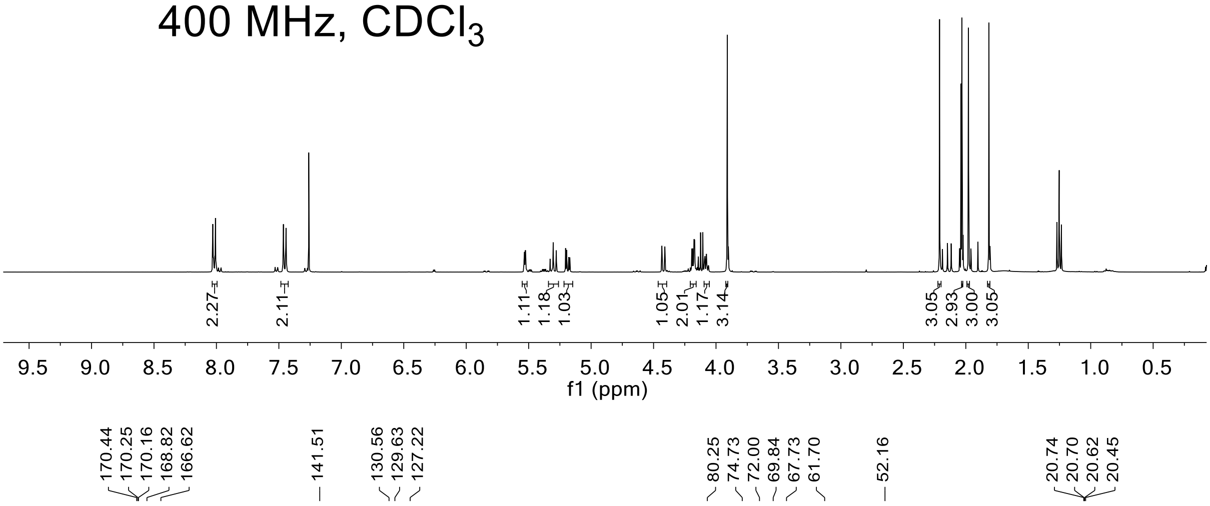

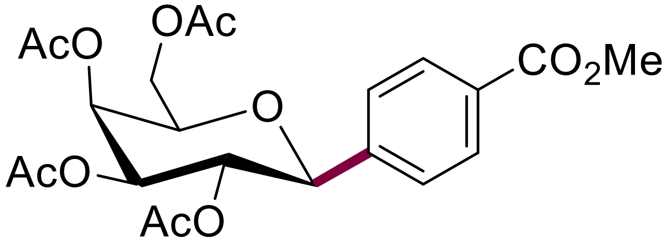

42ba

101 MHz, $CDCl_3$

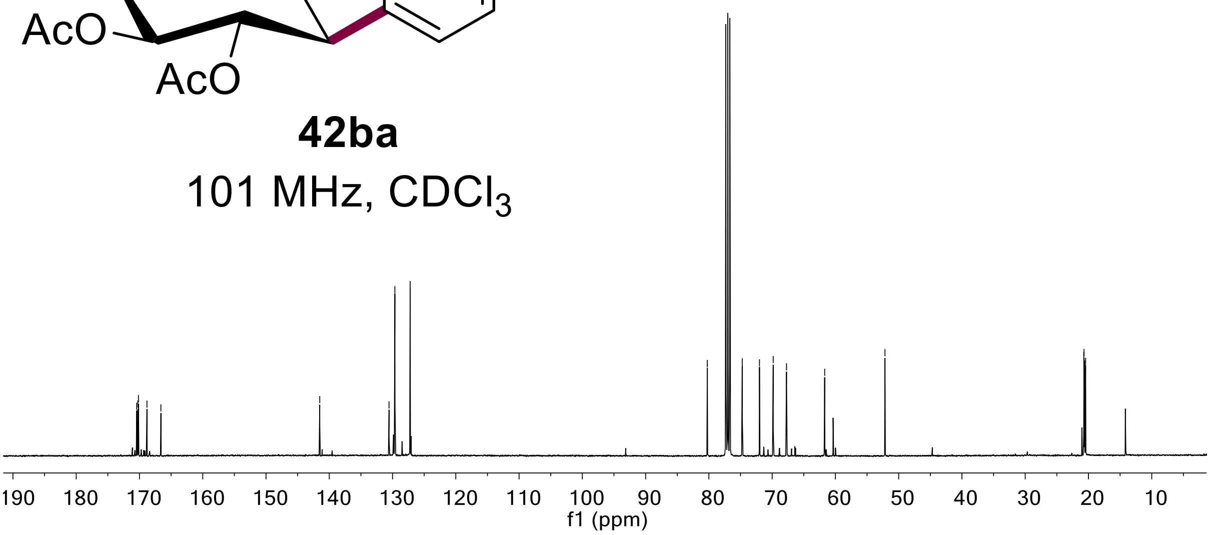

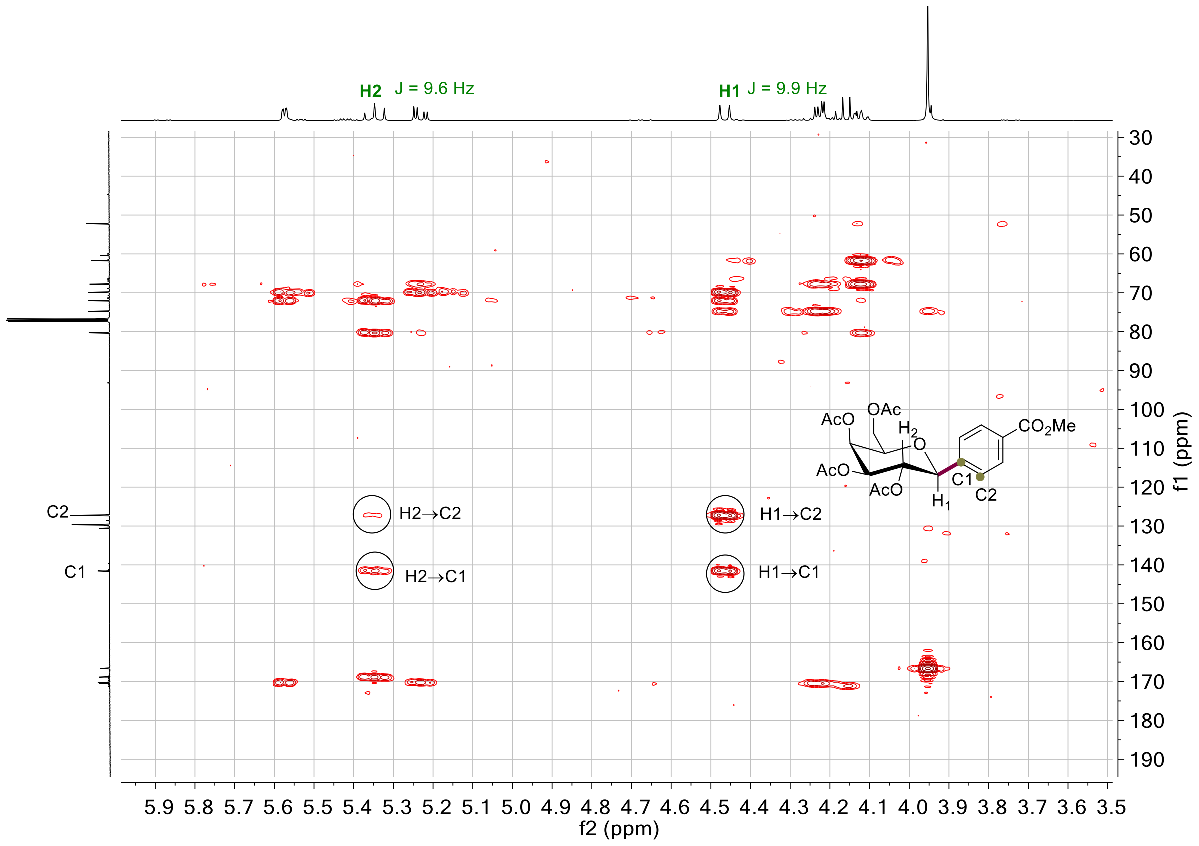
H2 J = 9.6 Hz
H1 J = 9.9 Hz
H2→C2
H2→C1
H1→C2
H1→C1
C2
C1
AcO
OAc
AcO
AcO
O
CO2Me
f1 (ppm)
f2 (ppm)

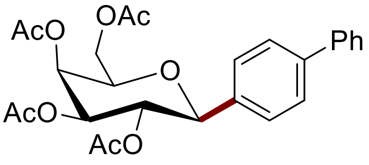

42bb
400 MHz, $CDCl_3$

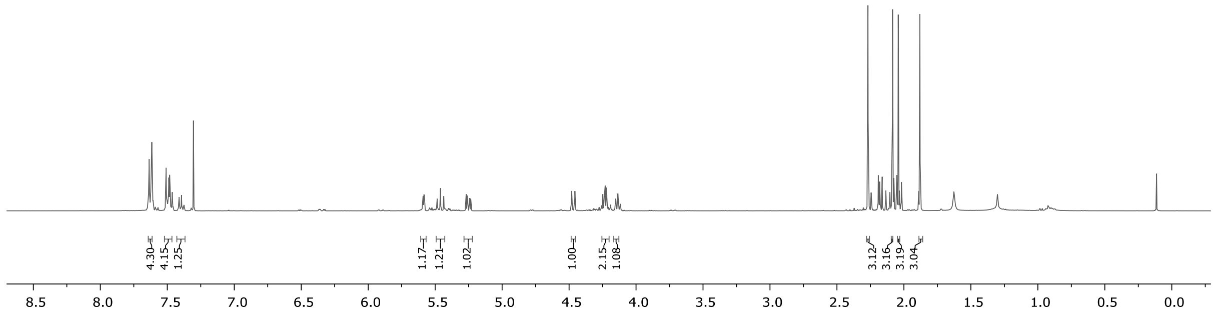

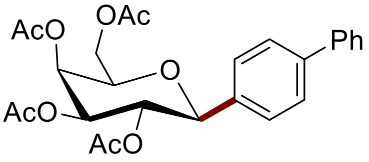

42bb
101 MHz, $CDCl_3$

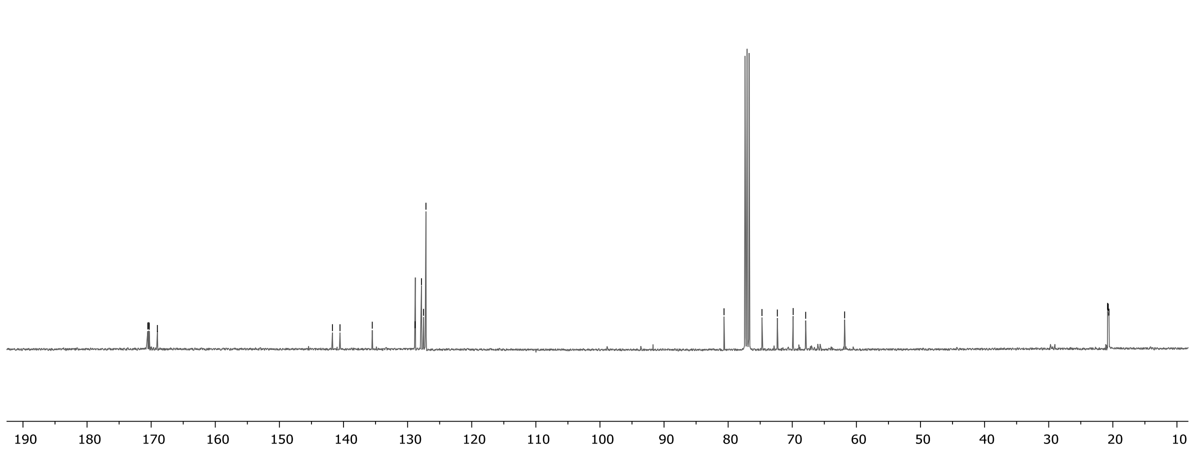

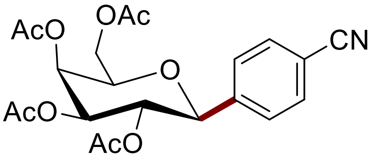

42bc
400 MHz, $CDCl_3$

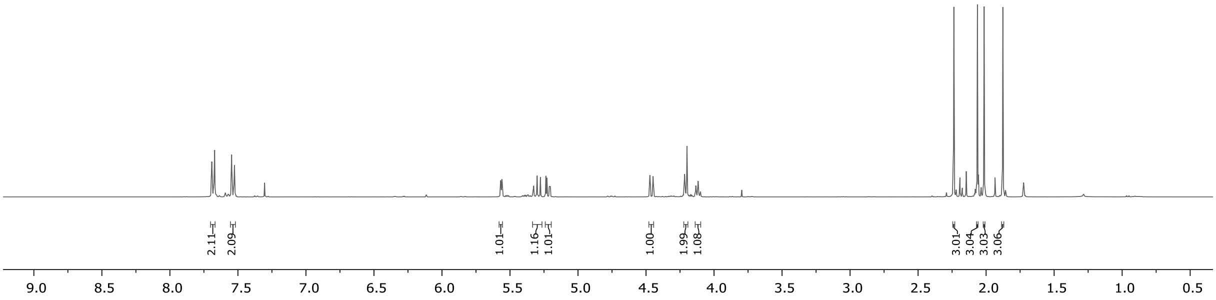

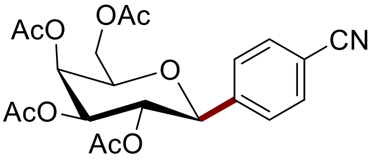

42bc
101 MHz, $CDCl_3$

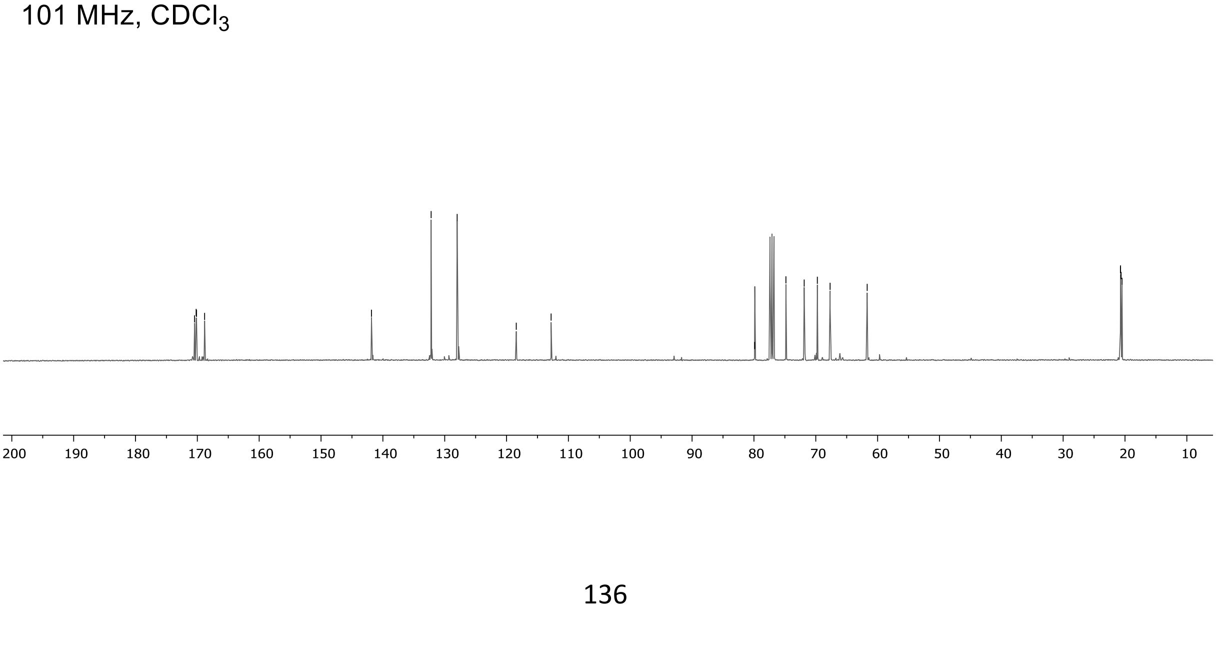

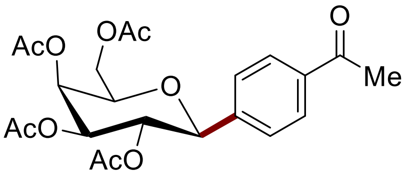

42bd

400 MHz, $CDCl_3$

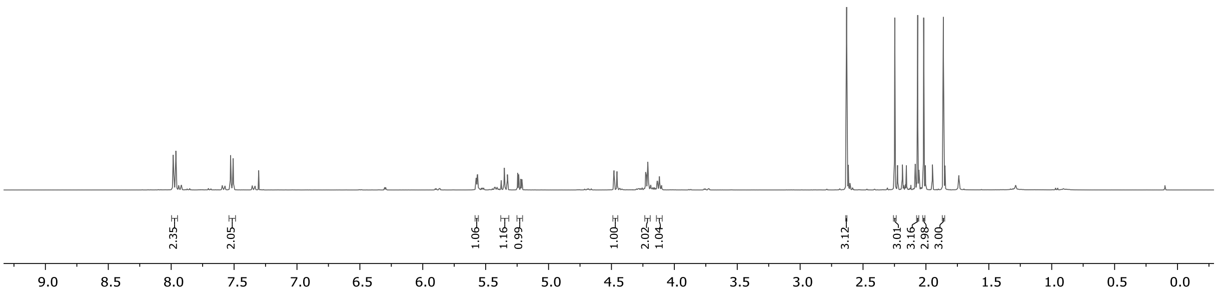

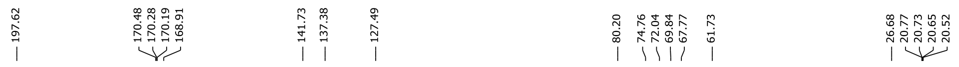

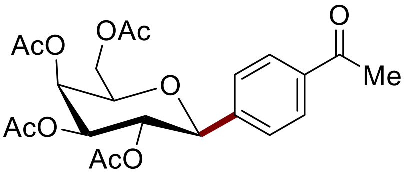

42bd

101 MHz, $CDCl_3$

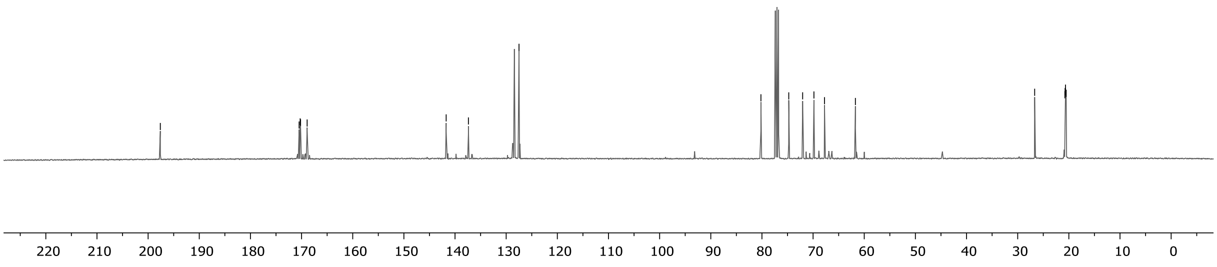

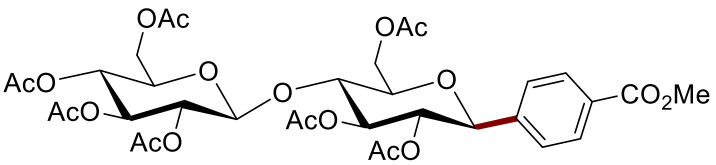

42be
400 MHz, $CDCl_3$

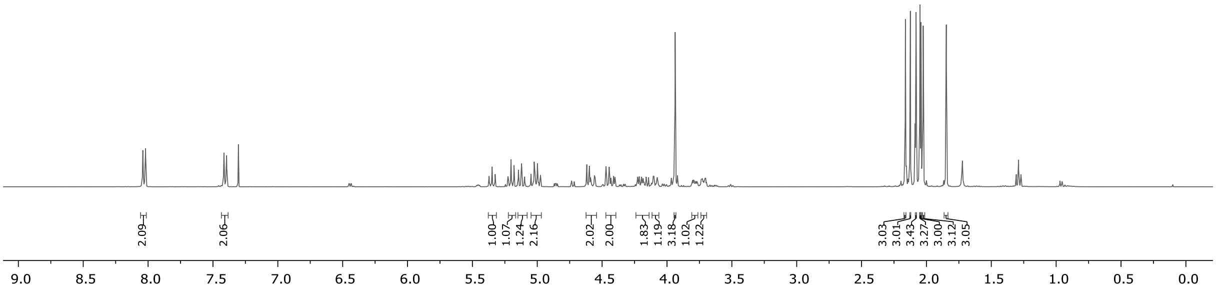

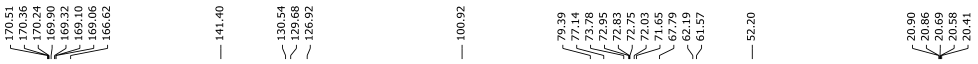

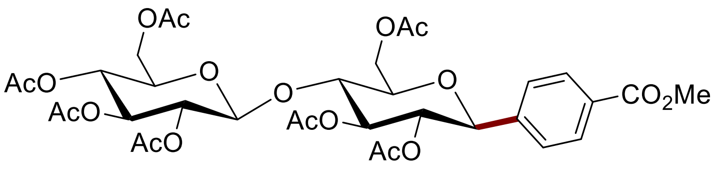

42be
101 MHz, $CDCl_3$

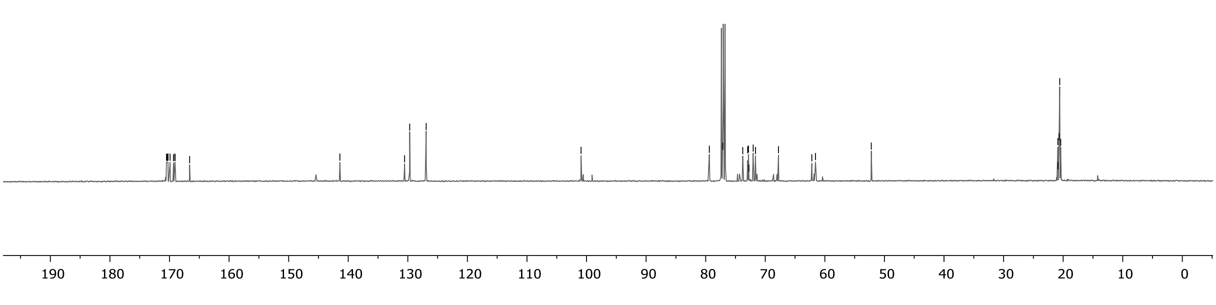

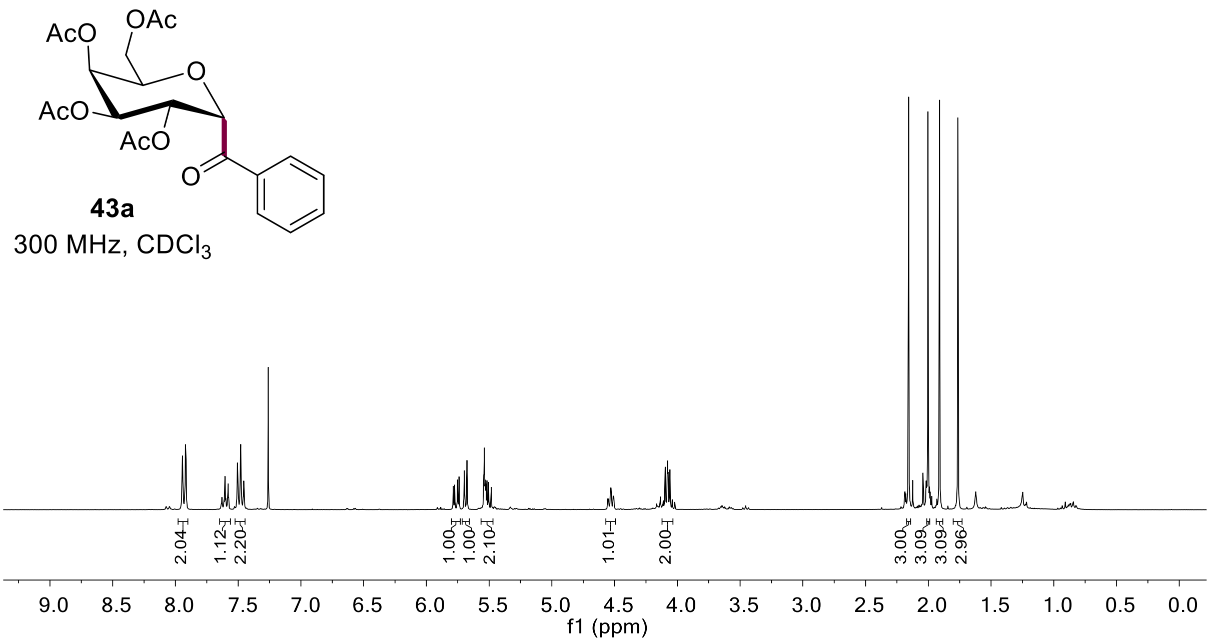
AcO
OAc
O
AcO
AcO
O
43a
300 MHz, CDCl3
2.04
1.12
2.20
1.00
1.00
2.10
1.01
2.00
3.00
3.09
3.09
2.96
9.0 8.5 8.0 7.5 7.0 6.5 6.0 5.5 5.0 4.5 4.0 3.5 3.0 2.5 2.0 1.5 1.0 0.5 0.0
f1 (ppm)

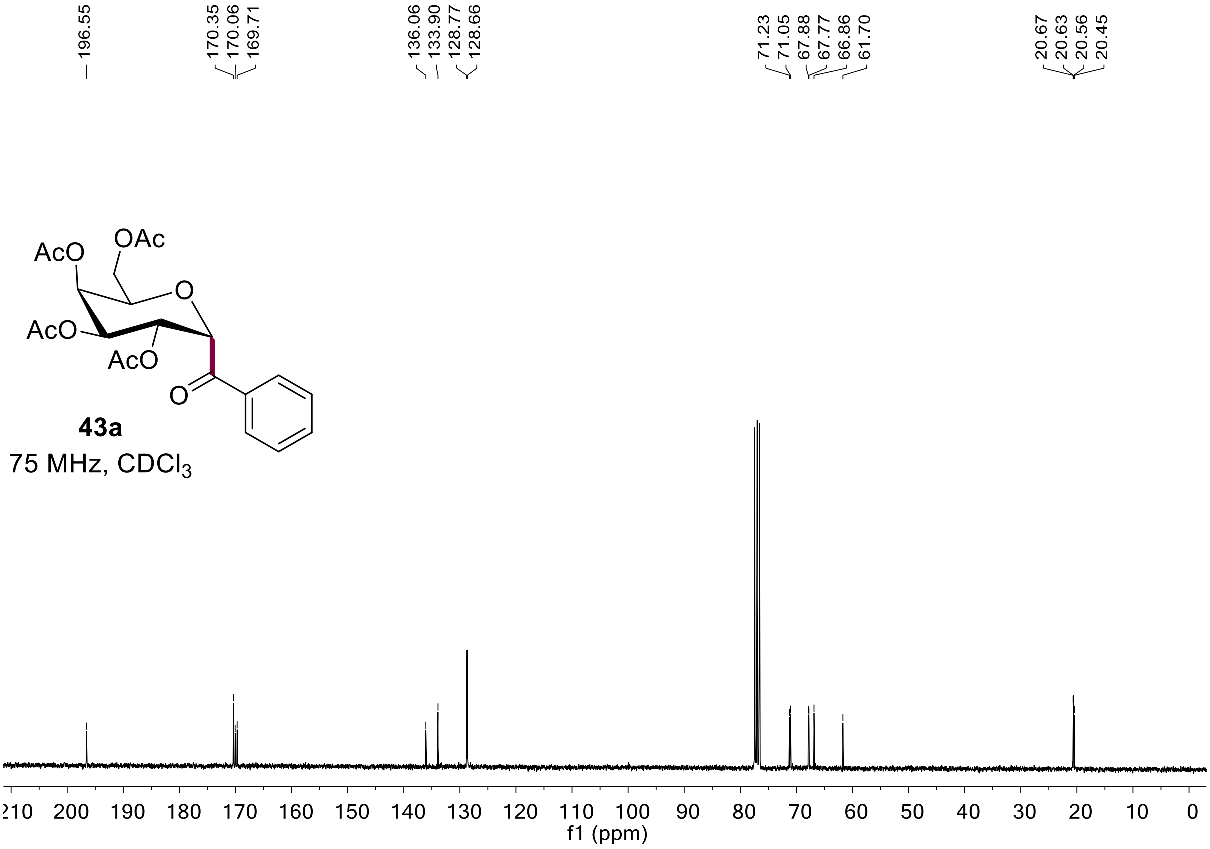
196.55
170.35
170.06
169.71
136.06
133.90
128.77
128.66
71.23
71.05
67.88
67.77
66.86
61.70
20.67
20.63
20.56
20.45
AcO
OAc
O
AcO
AcO
O
43a
75 MHz, CDCl3
210 200 190 180 170 160 150 140 130 120 110 100 90 80 70 60 50 40 30 20 10 0
f1 (ppm)

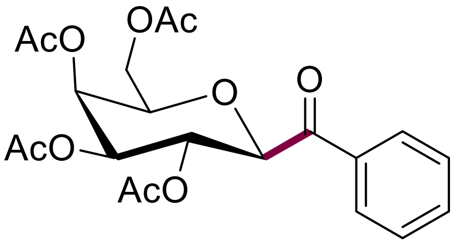

43ba

300 MHz, $CDCl_3$

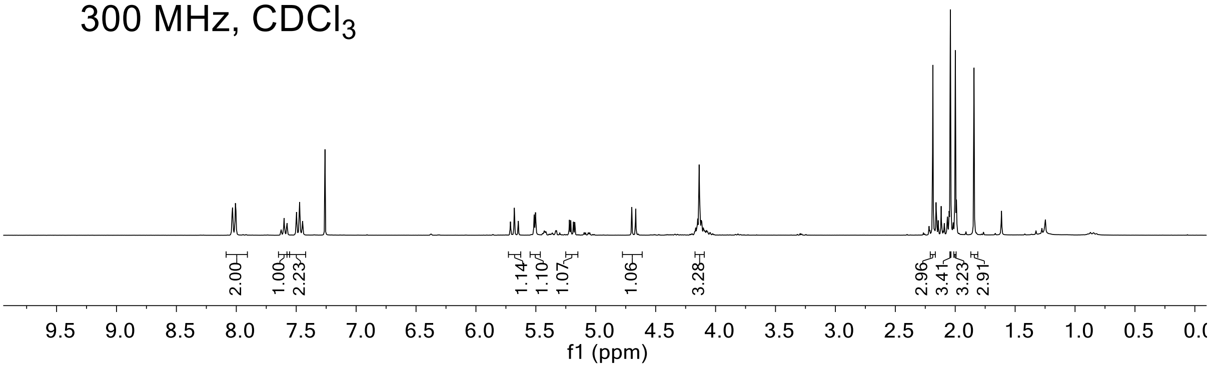

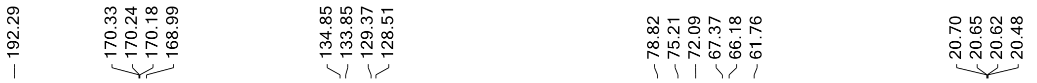

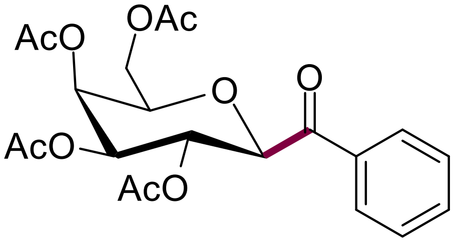

43ba

75 MHz, $CDCl_3$

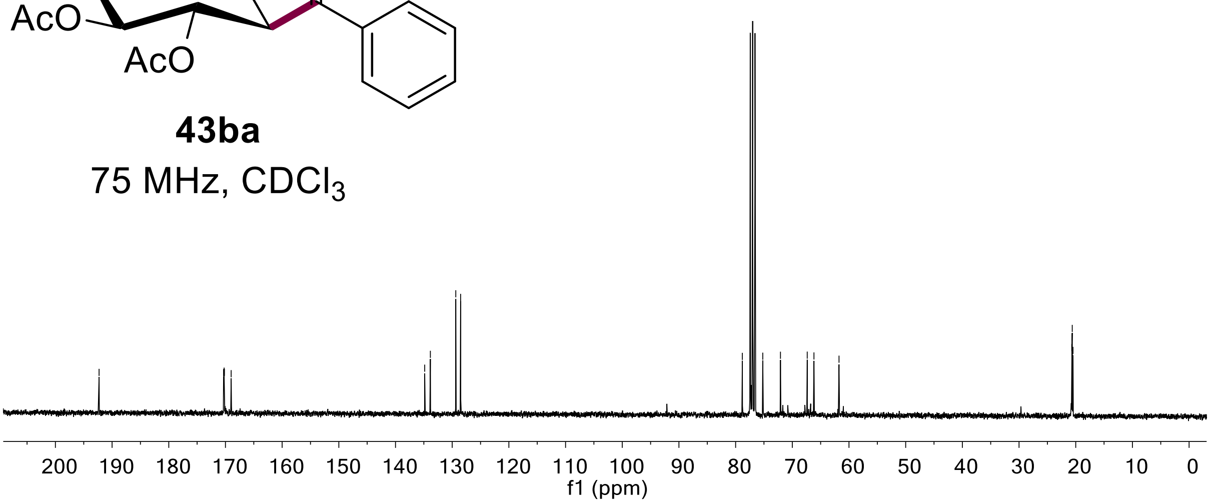

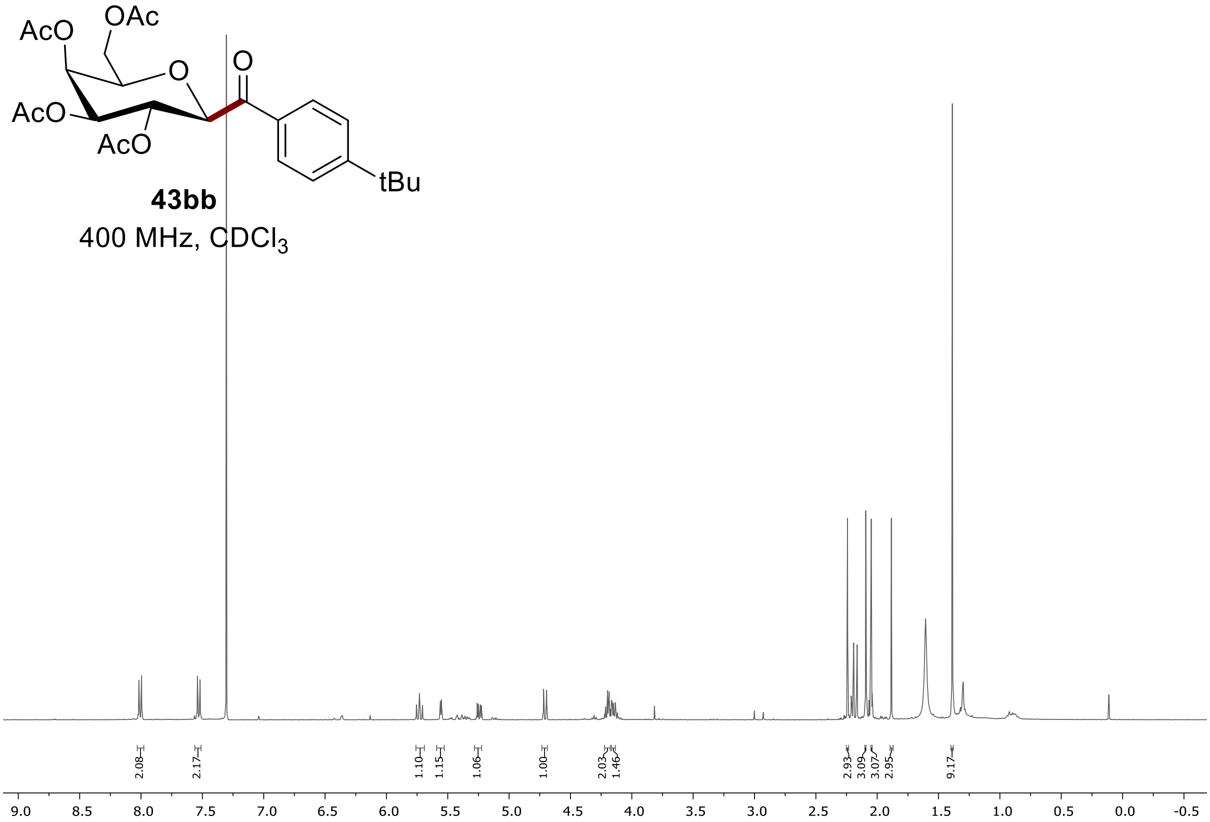
AcO
OAc
O
O
AcO
AcO
tBu
43bb
400 MHz, CDCl3
2.08
2.17
1.10
1.15
1.06
1.00
2.03
1.46
2.93
3.09
3.07
2.95
9.17
9.0 8.5 8.0 7.5 7.0 6.5 6.0 5.5 5.0 4.5 4.0 3.5 3.0 2.5 2.0 1.5 1.0 0.5 0.0 -0.5

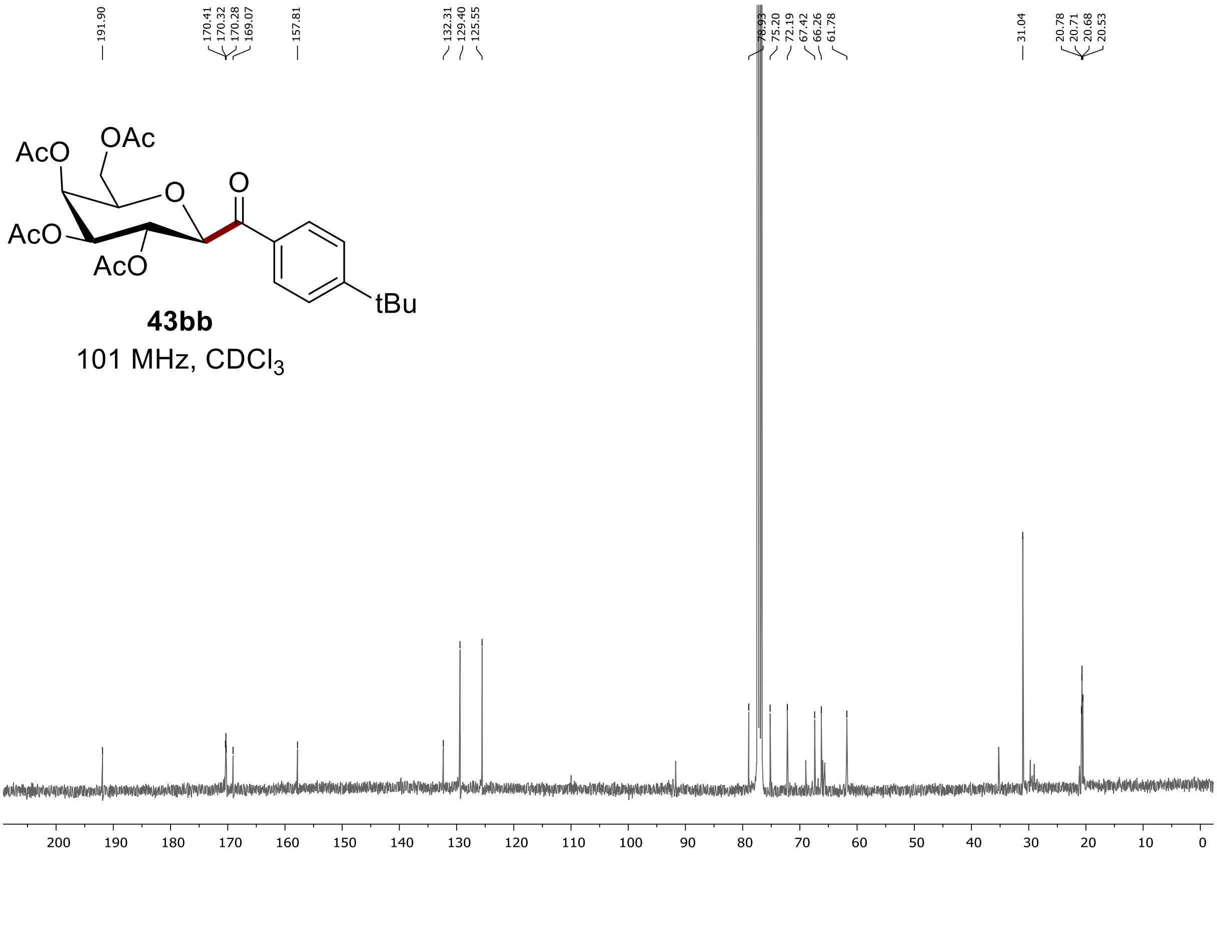
191.90
170.41
170.32
170.28
169.07
157.81
132.31
129.40
125.55
78.93
75.20
72.19
67.42
66.26
61.78
31.04
20.78
20.71
20.68
20.53
AcO
OAc
O
O
AcO
AcO
tBu
43bb
101 MHz, CDCl3
200 190 180 170 160 150 140 130 120 110 100 90 80 70 60 50 40 30 20 10 0

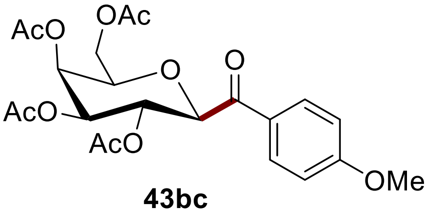

43bc

400 MHz, $CDCl_3$

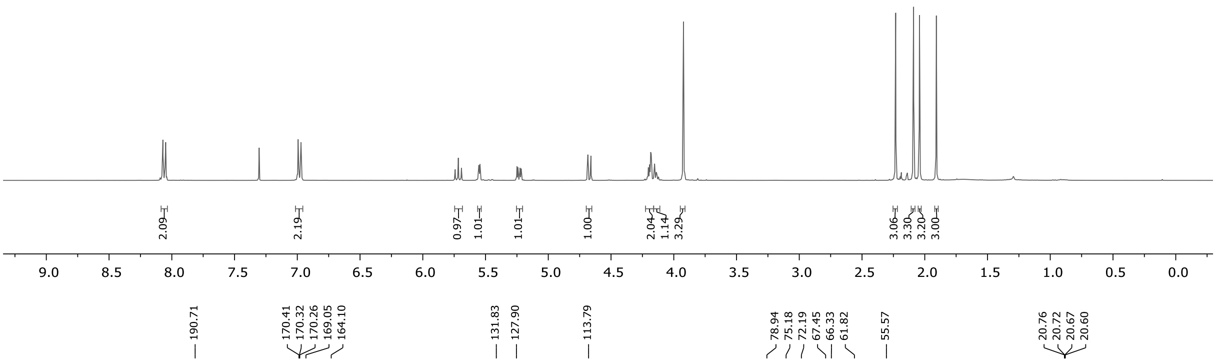

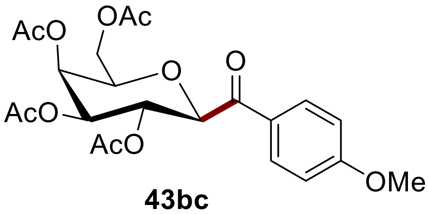

43bc

101 MHz, $CDCl_3$

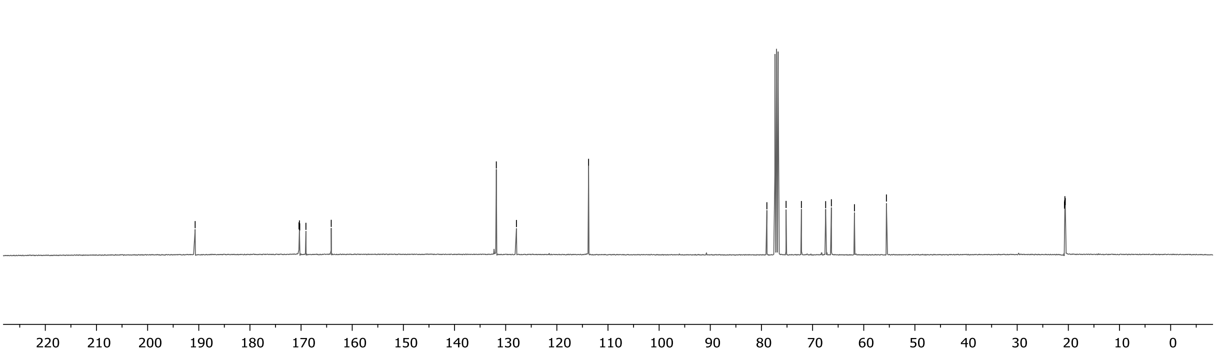

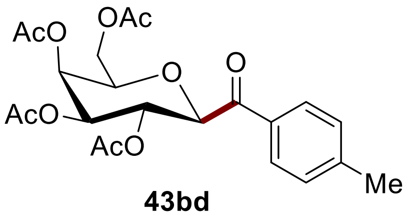

43bd
400 MHz, $CDCl_3$

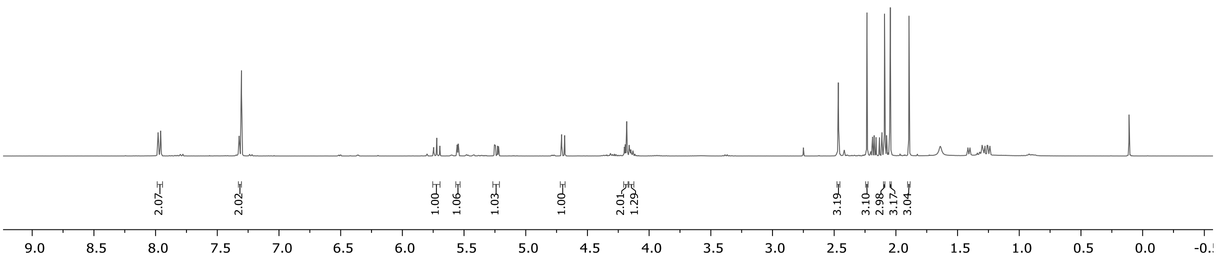

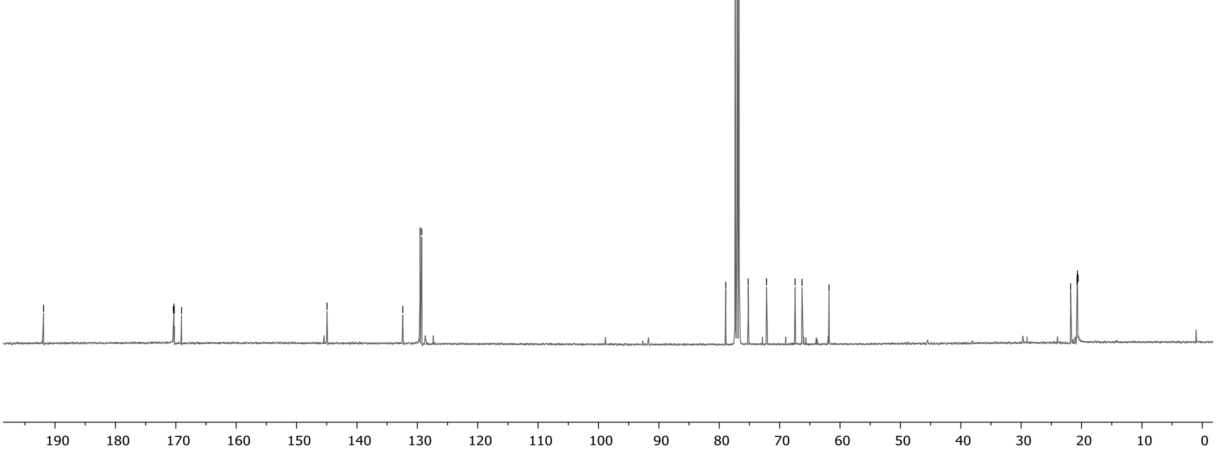

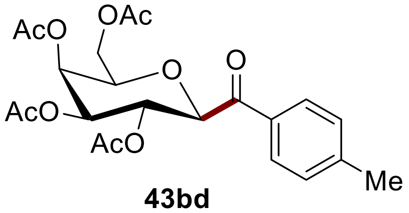

43bd
101 MHz, $CDCl_3$

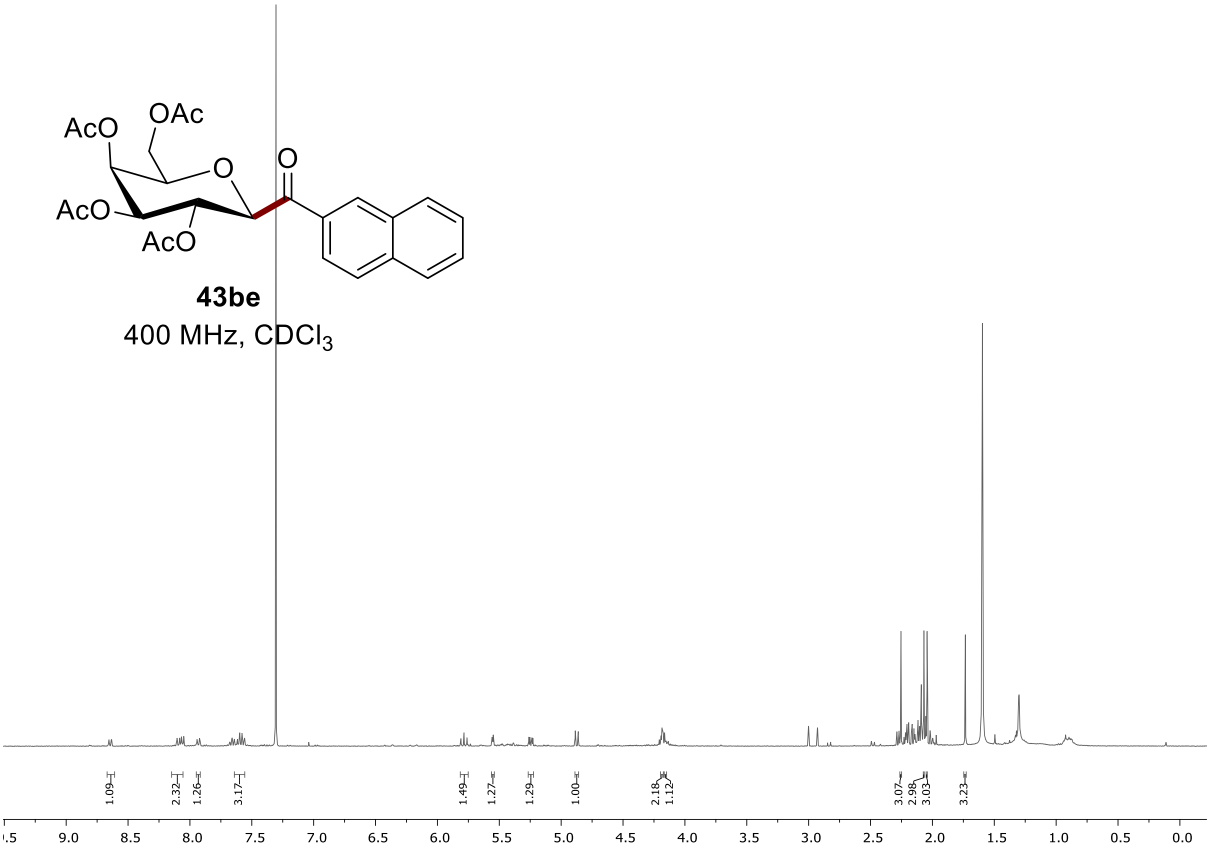
AcO
OAc
O
O
AcO
AcO
43be
400 MHz, CDCl3
1.09
2.32
1.26
3.17
1.49
1.27
1.29
1.00
2.18
1.12
3.07
2.98
3.03
3.23
9.0 8.5 8.0 7.5 7.0 6.5 6.0 5.5 5.0 4.5 4.0 3.5 3.0 2.5 2.0 1.5 1.0 0.5 0.0

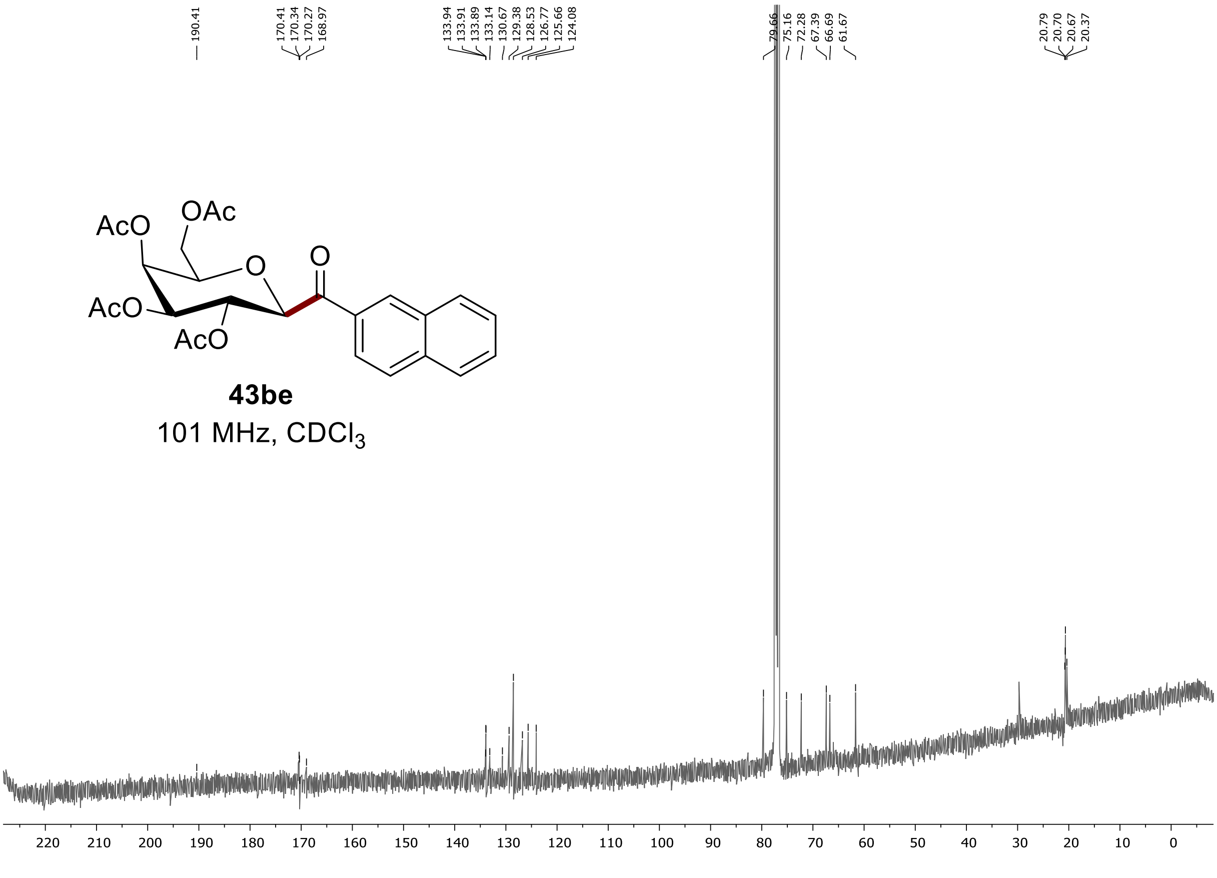
190.41
170.41
170.34
170.27
168.97
133.94
133.91
133.89
133.14
130.67
129.38
128.53
126.77
125.66
124.08
79.66
75.16
72.28
67.39
66.69
61.67
20.79
20.70
20.67
20.37
AcO
OAc
O
O
AcO
AcO
43be
101 MHz, CDCl3
220 210 200 190 180 170 160 150 140 130 120 110 100 90 80 70 60 50 40 30 20 10 0

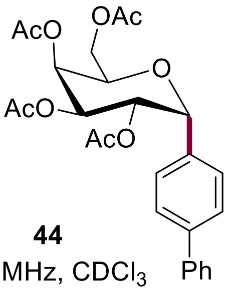

44

400 MHz, $CDCl_3$

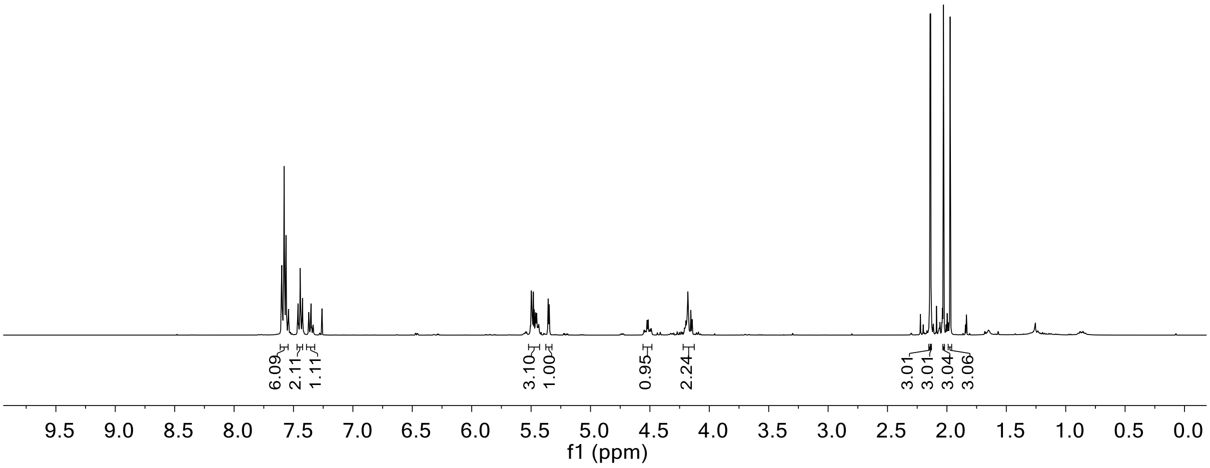

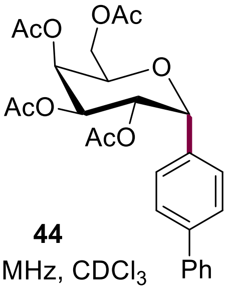

44

101 MHz, $CDCl_3$

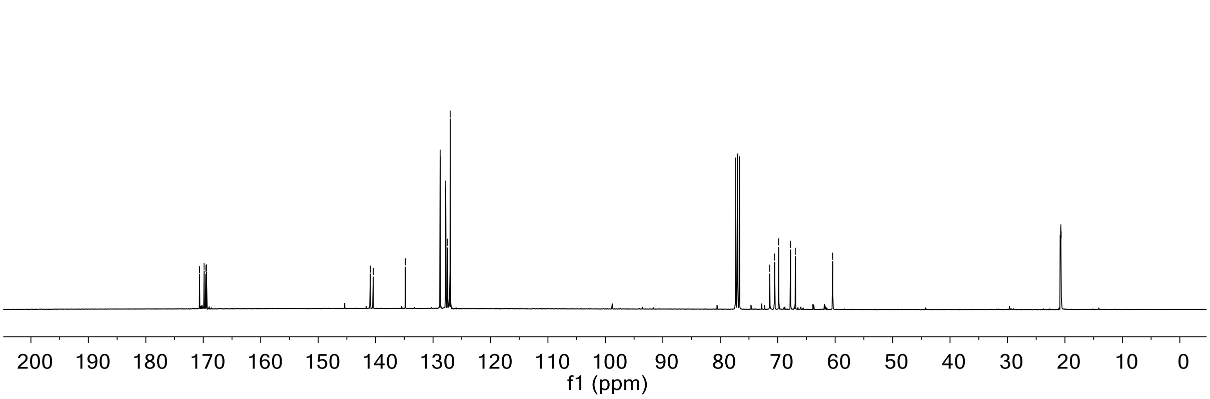

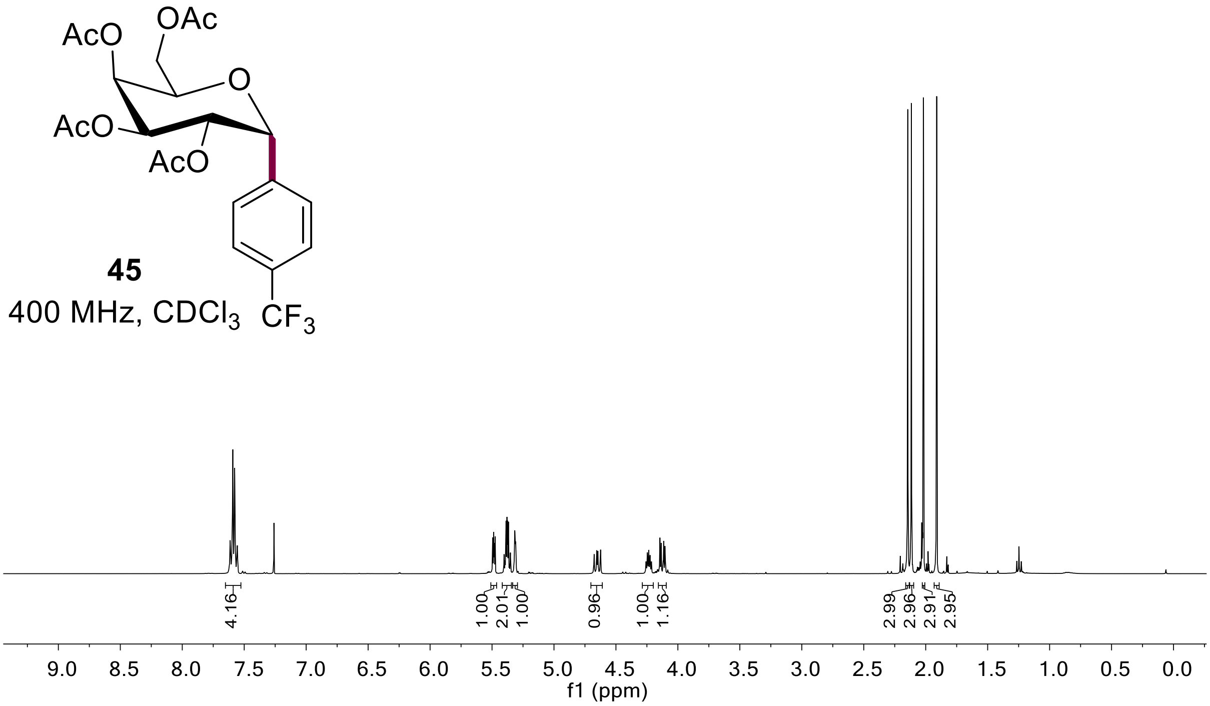
AcO
OAc
O
AcO
AcO
CF3
45
400 MHz, CDCl3
4.16
1.00
2.01
1.00
0.96
1.00
1.16
2.99
2.96
2.91
2.95
9.0 8.5 8.0 7.5 7.0 6.5 6.0 5.5 5.0 4.5 4.0 3.5 3.0 2.5 2.0 1.5 1.0 0.5 0.0
f1 (ppm)

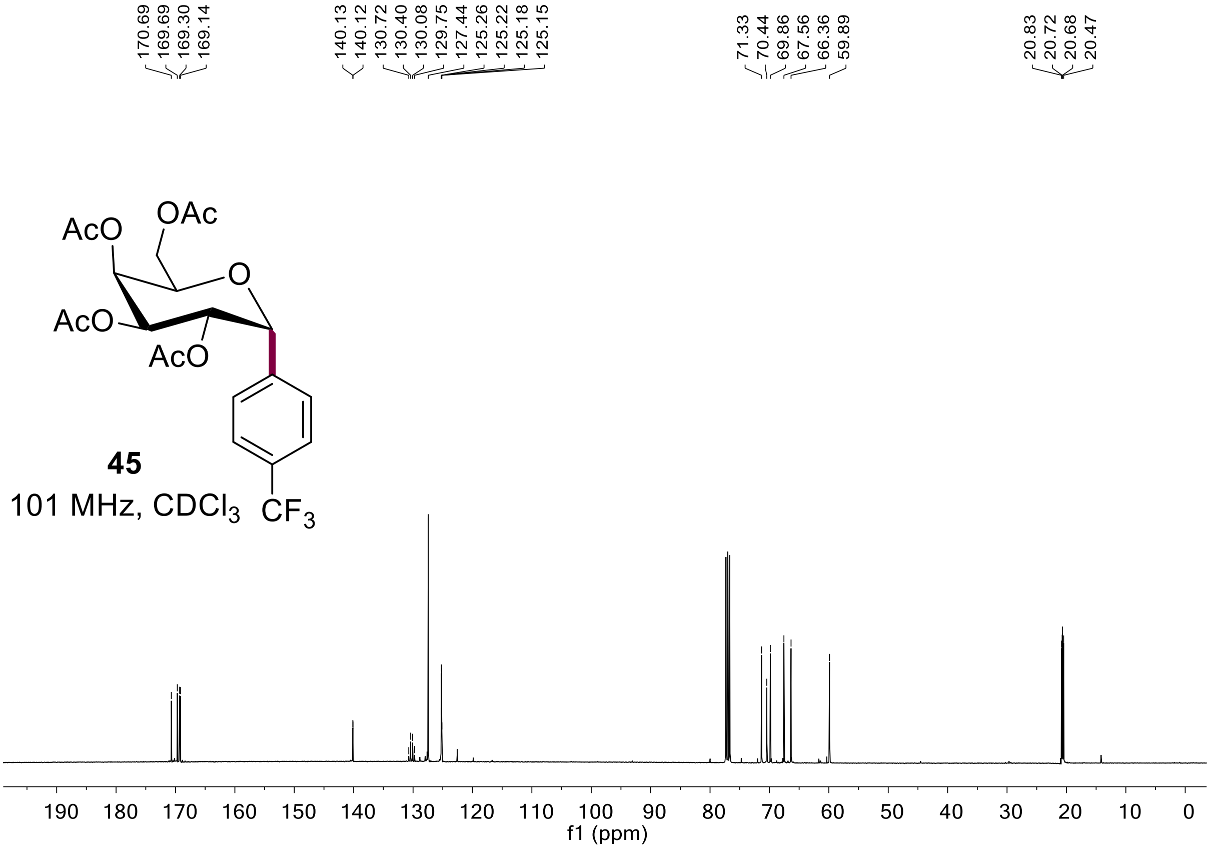
170.69
169.69
169.30
169.14
140.13
140.12
130.72
130.40
130.08
129.75
127.44
125.26
125.22
125.18
125.15
71.33
70.44
69.86
67.56
66.36
59.89
20.83
20.72
20.68
20.47
AcO
OAc
O
AcO
AcO
CF3
45
101 MHz, CDCl3
190 180 170 160 150 140 130 120 110 100 90 80 70 60 50 40 30 20 10 0
f1 (ppm)

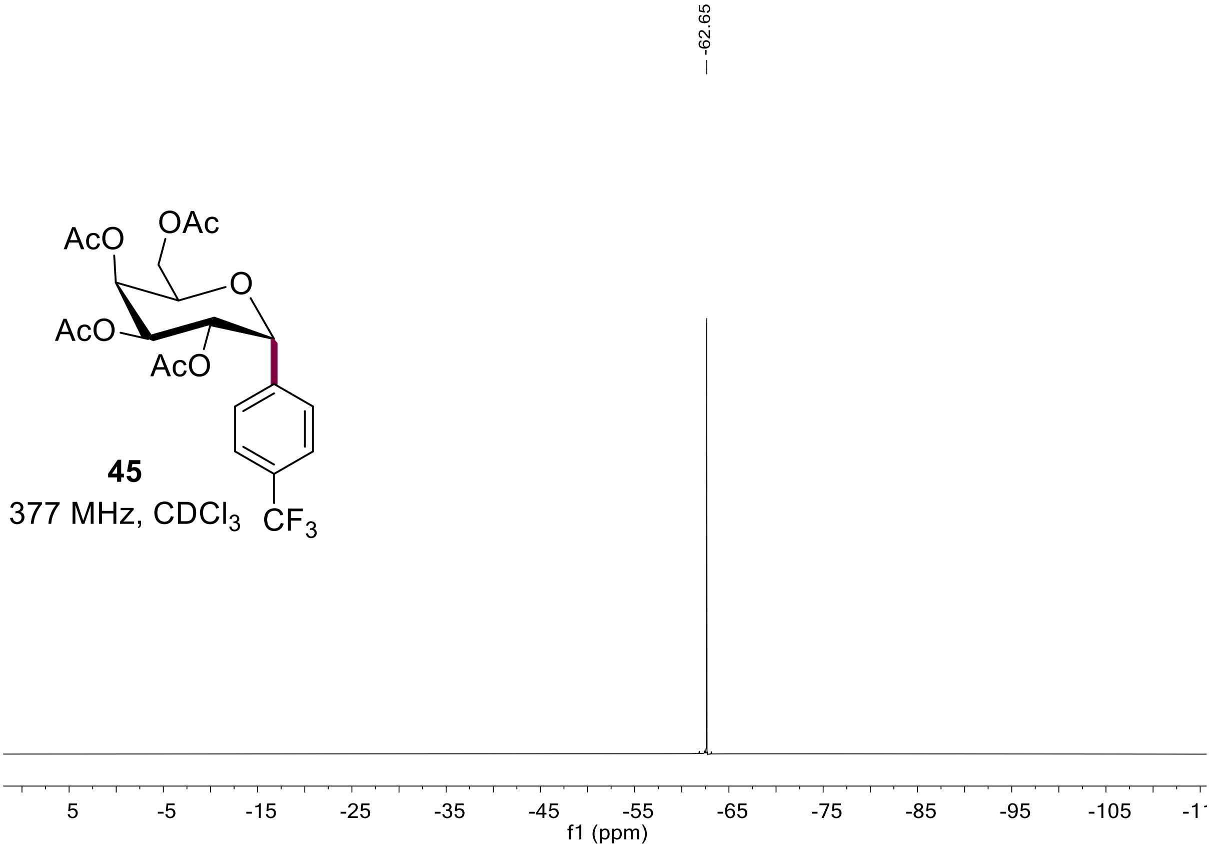

45

377 MHz, $CDCl_3$

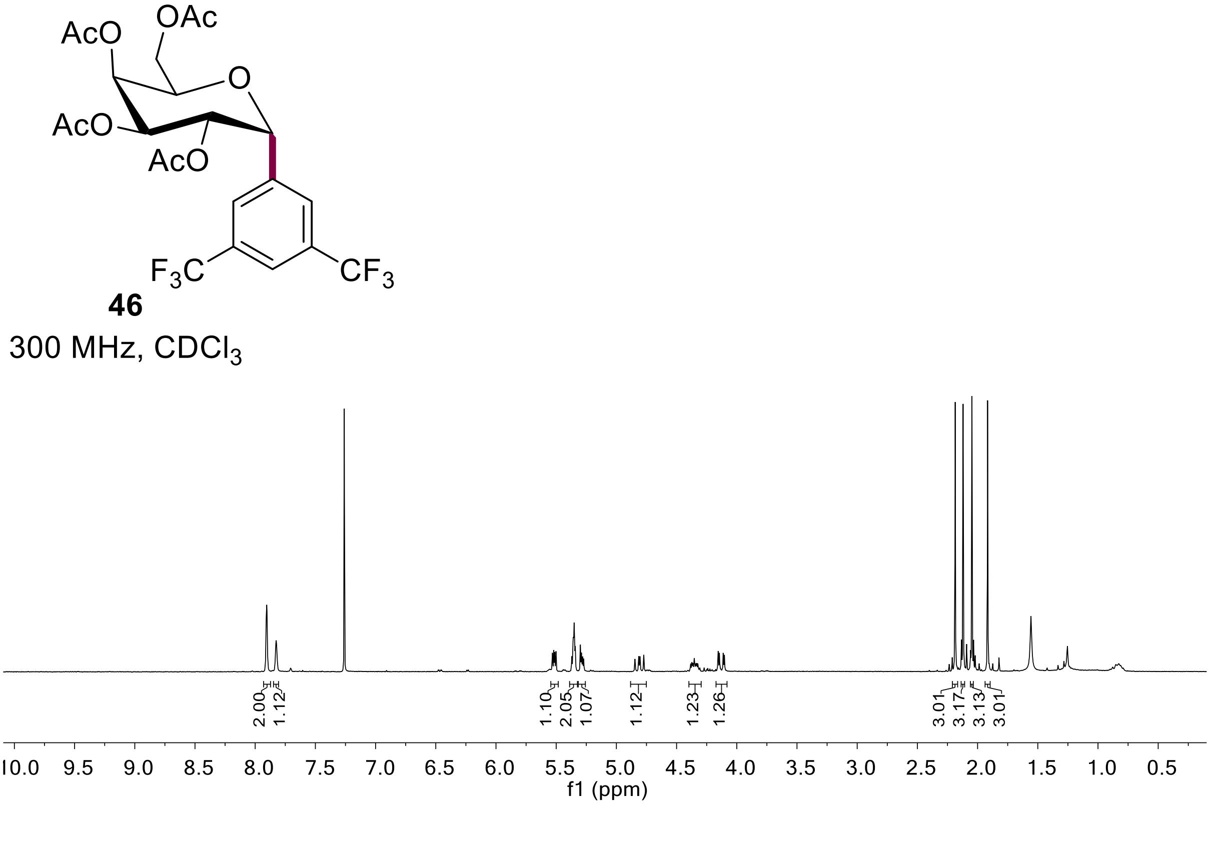

46

300 MHz, $CDCl_3$

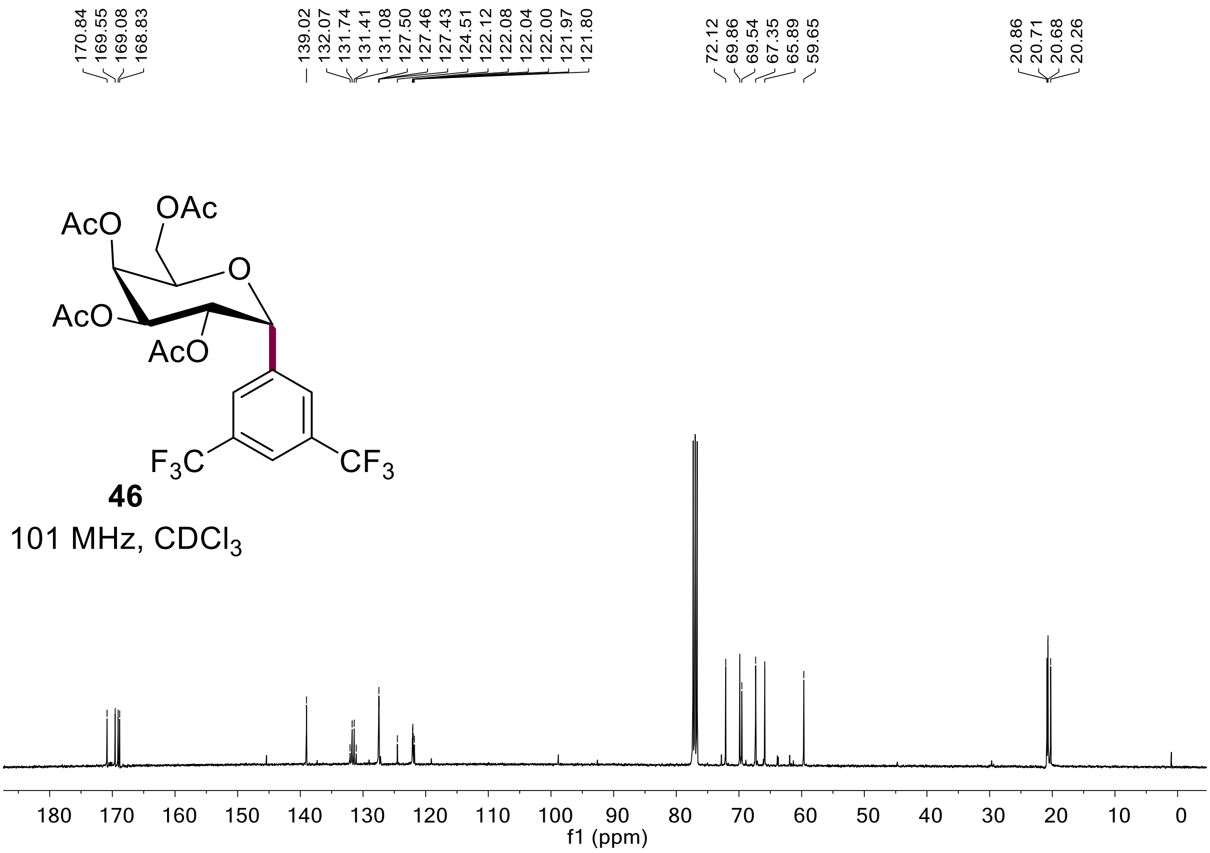
170.84
169.55
169.08
168.83
139.02
132.07
131.74
131.41
131.08
127.50
127.46
127.43
124.51
122.12
122.08
122.04
122.00
121.97
121.80
72.12
69.86
69.54
67.35
65.89
59.65
20.86
20.71
20.68
20.26
AcO
OAc
O
AcO
AcO
F$_3$C
CF$_3$
46
101 MHz, CDCl$_3$
f1 (ppm)

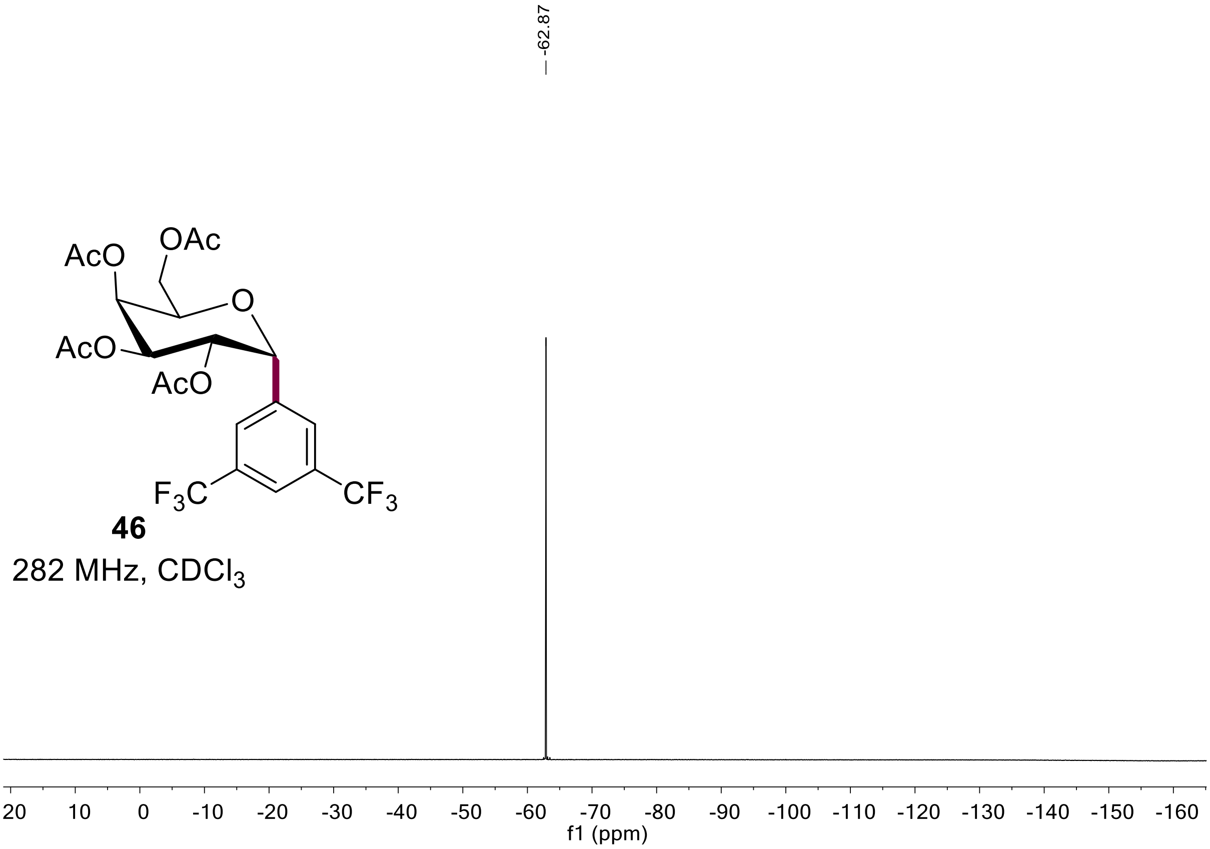
-62.87
AcO
OAc
O
AcO
AcO
F$_3$C
CF$_3$
46
282 MHz, CDCl$_3$
f1 (ppm)

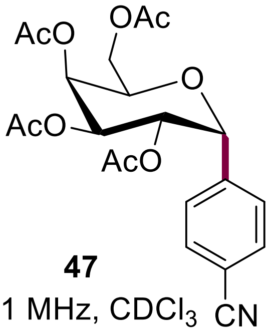

47

101 MHz, $CDCl_3$

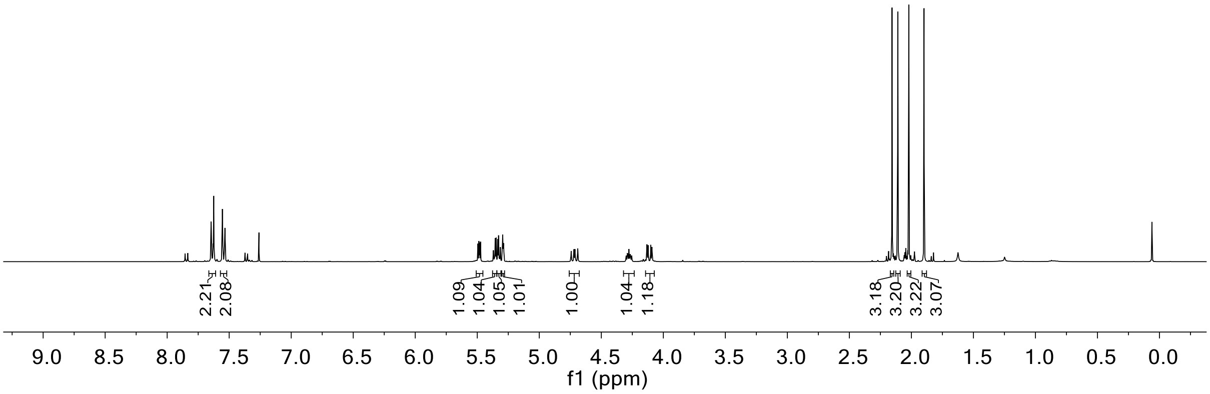

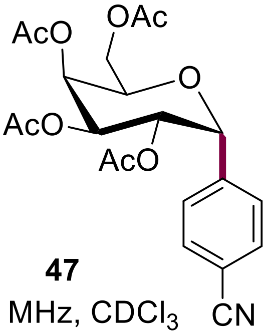

47

101 MHz, $CDCl_3$

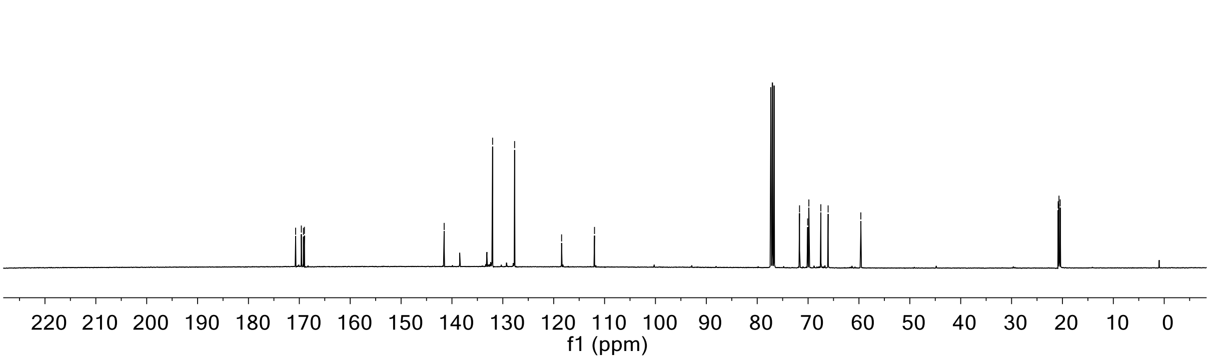

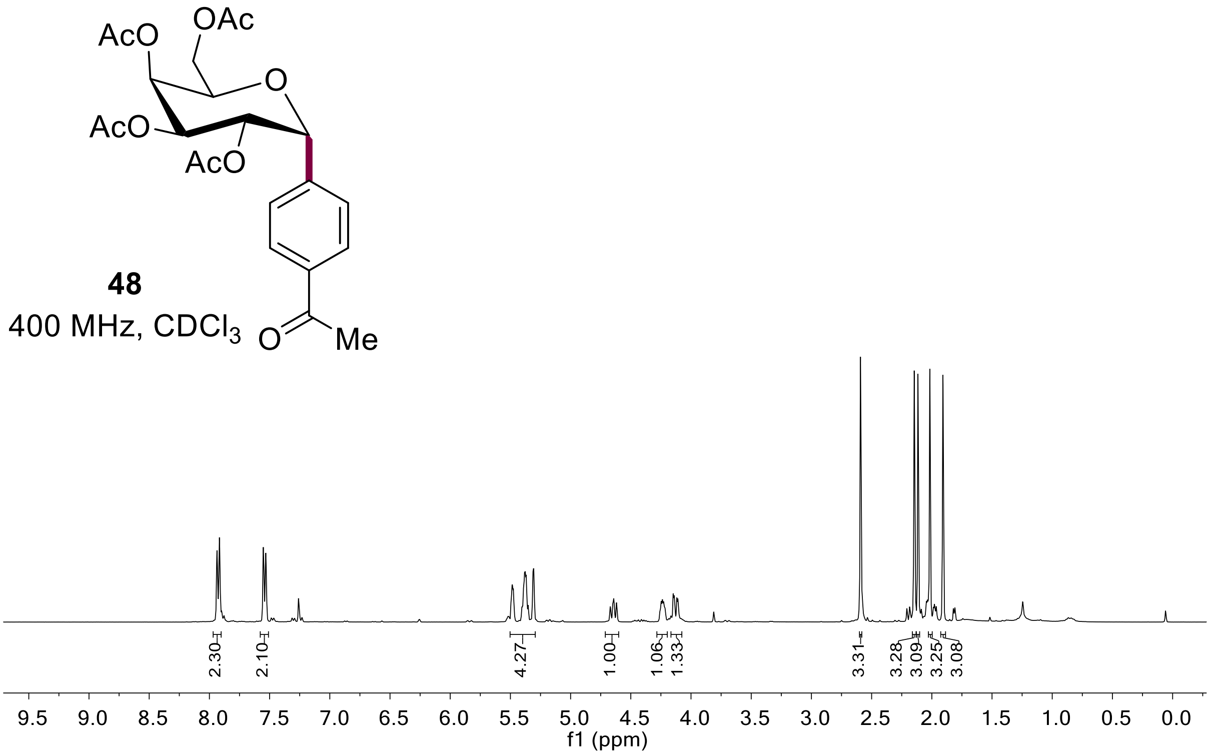

AcO
OAc
O
AcO
AcO
48
400 MHz, CDCl3
O
Me
2.30
2.10
4.27
1.00
1.06
1.33
3.31
3.28
3.09
3.25
3.08
9.5 9.0 8.5 8.0 7.5 7.0 6.5 6.0 5.5 5.0 4.5 4.0 3.5 3.0 2.5 2.0 1.5 1.0 0.5 0.0
f1 (ppm)

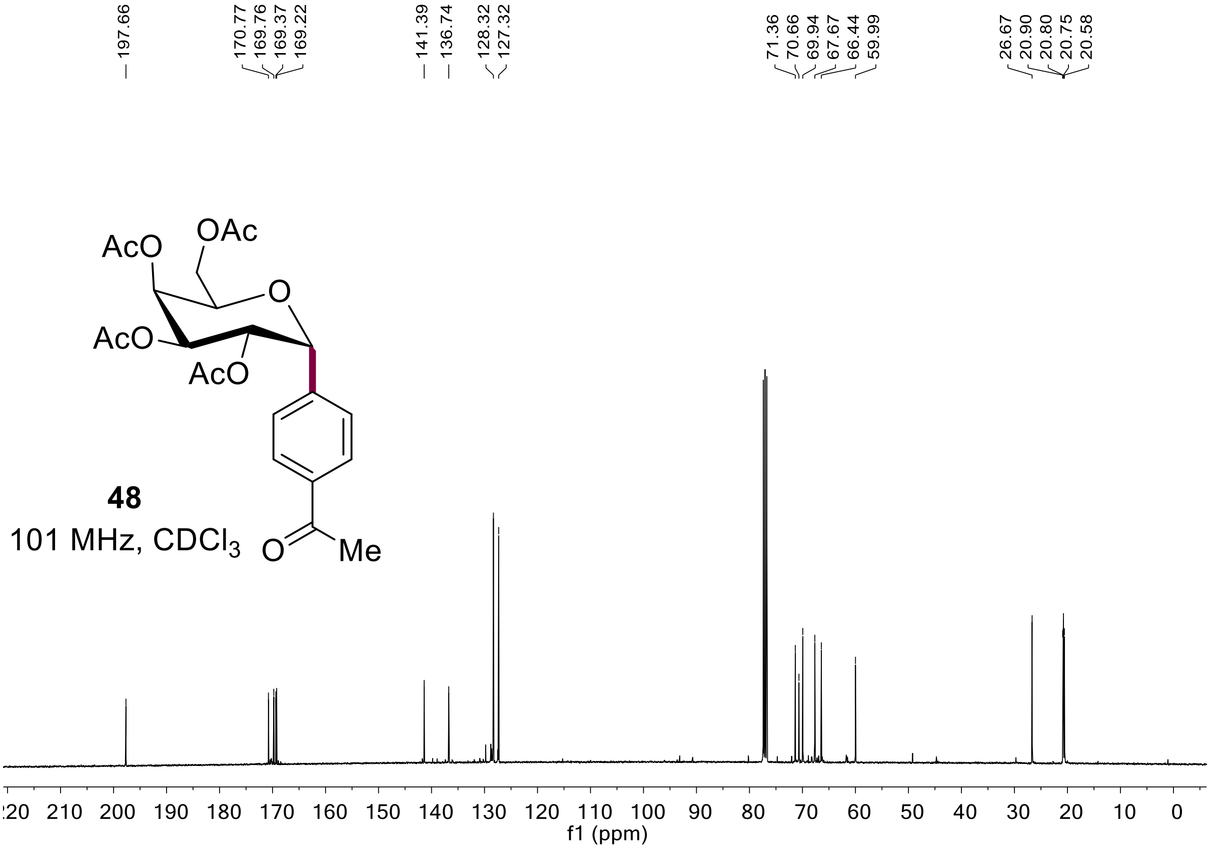

197.66
170.77
169.76
169.37
169.22
141.39
136.74
128.32
127.32
71.36
70.66
69.94
67.67
66.44
59.99
26.67
20.90
20.80
20.75
20.58
AcO
OAc
O
AcO
AcO
48
101 MHz, CDCl3
O
Me
20 210 200 190 180 170 160 150 140 130 120 110 100 90 80 70 60 50 40 30 20 10 0
f1 (ppm)

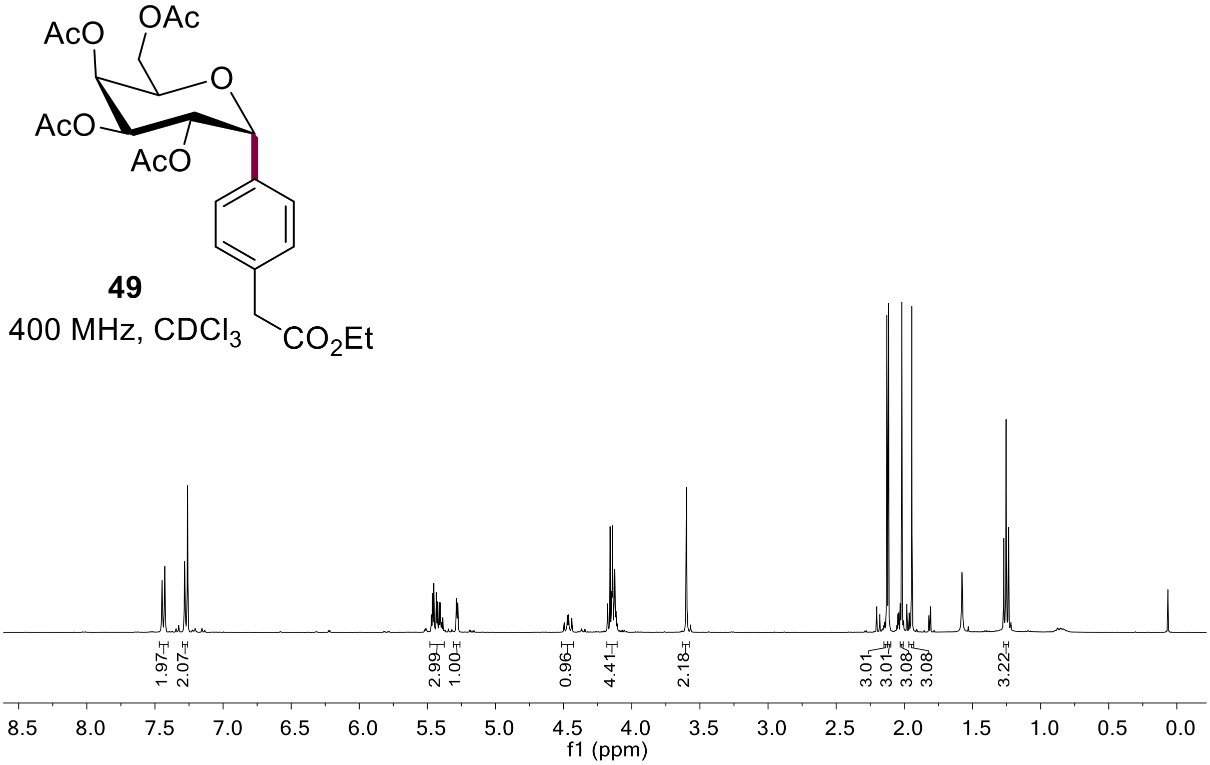
AcO
OAc
O
AcO
AcO
49
400 MHz, CDCl3
CO2Et
1.97
2.07
2.99
1.00
0.96
4.41
2.18
3.01
3.01
3.08
3.08
3.22
8.5 8.0 7.5 7.0 6.5 6.0 5.5 5.0 4.5 4.0 3.5 3.0 2.5 2.0 1.5 1.0 0.5 0.0
f1 (ppm)

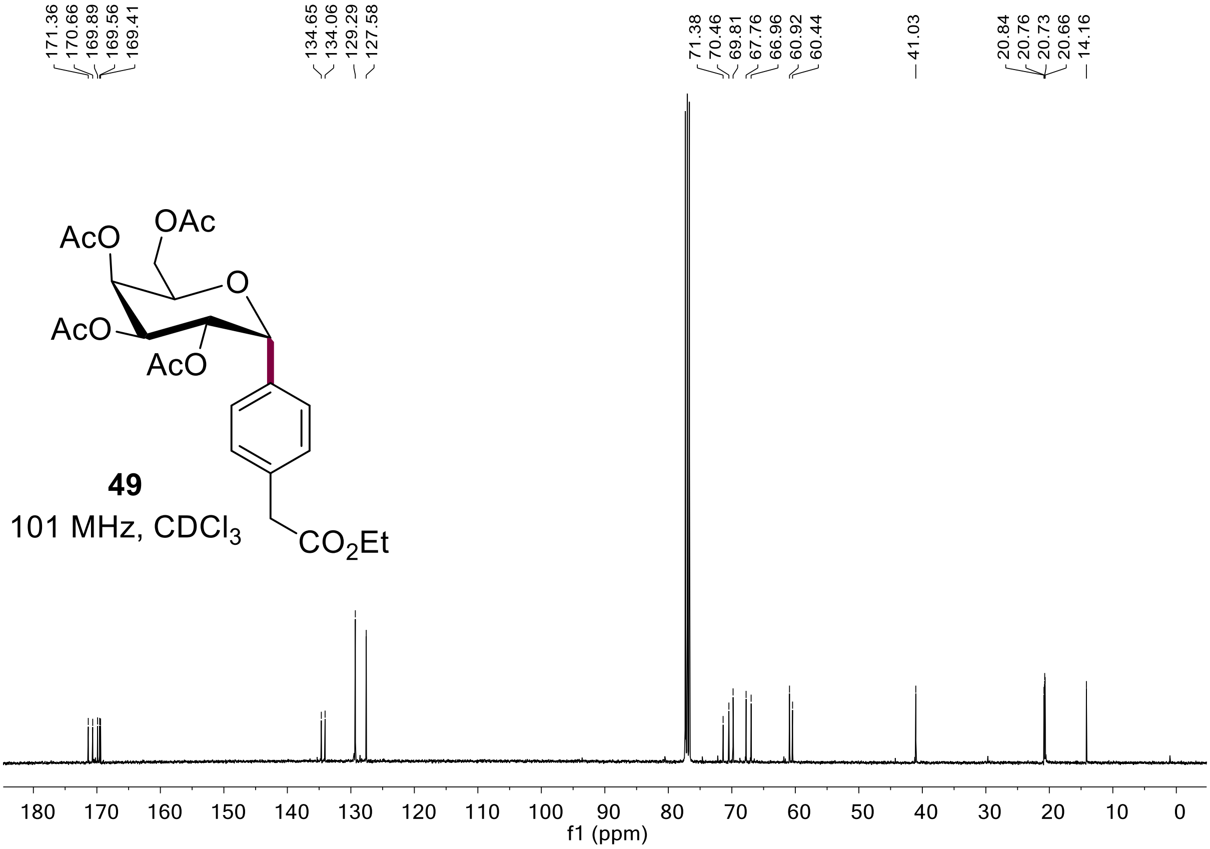
171.36
170.66
169.89
169.56
169.41
134.65
134.06
129.29
127.58
71.38
70.46
69.81
67.76
66.96
60.92
60.44
41.03
20.84
20.76
20.73
20.66
14.16
AcO
OAc
O
AcO
AcO
49
101 MHz, CDCl3
CO2Et
180 170 160 150 140 130 120 110 100 90 80 70 60 50 40 30 20 10 0
f1 (ppm)

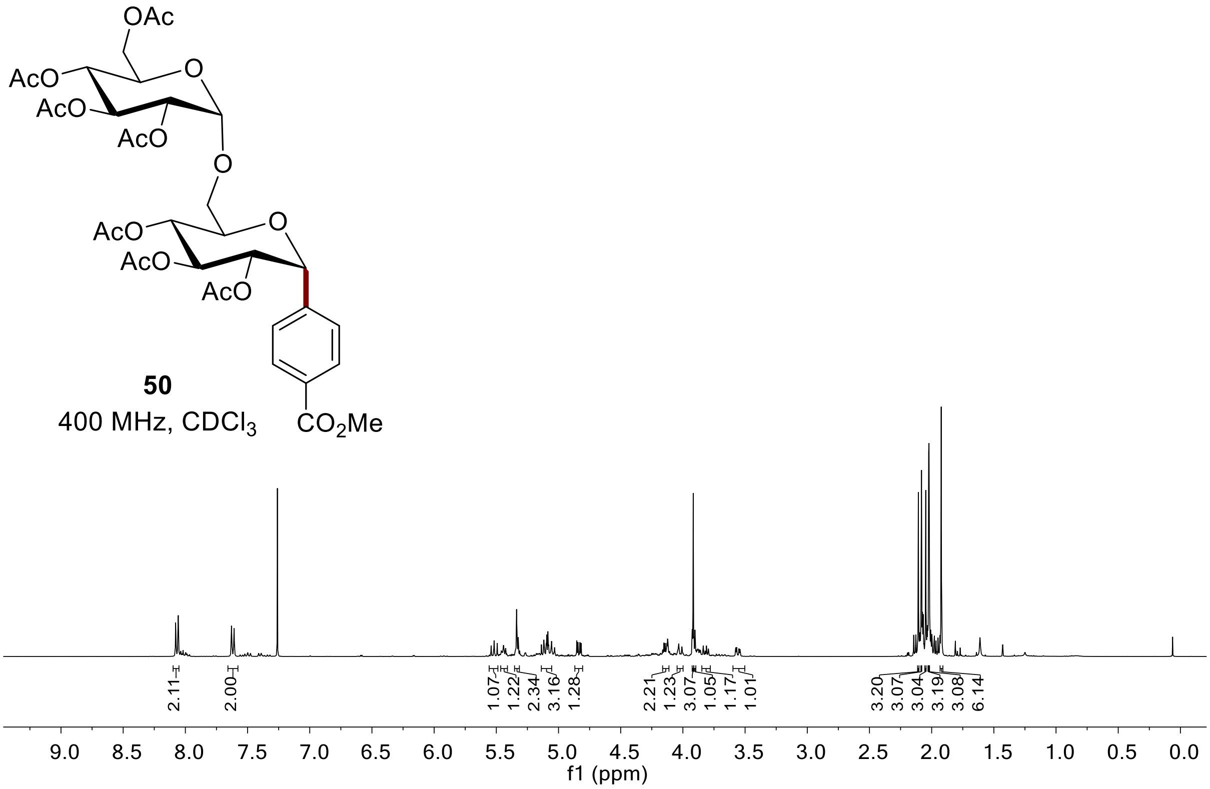
OAc
AcO
AcO
AcO
O
O
AcO
AcO
AcO
O
CO2Me
50
400 MHz, CDCl3
2.11
2.00
1.07
1.22
2.34
3.16
1.28
2.21
1.23
3.07
1.05
1.17
1.01
3.20
3.07
3.04
3.19
3.08
6.14
9.0 8.5 8.0 7.5 7.0 6.5 6.0 5.5 5.0 4.5 4.0 3.5 3.0 2.5 2.0 1.5 1.0 0.5 0.0
f1 (ppm)

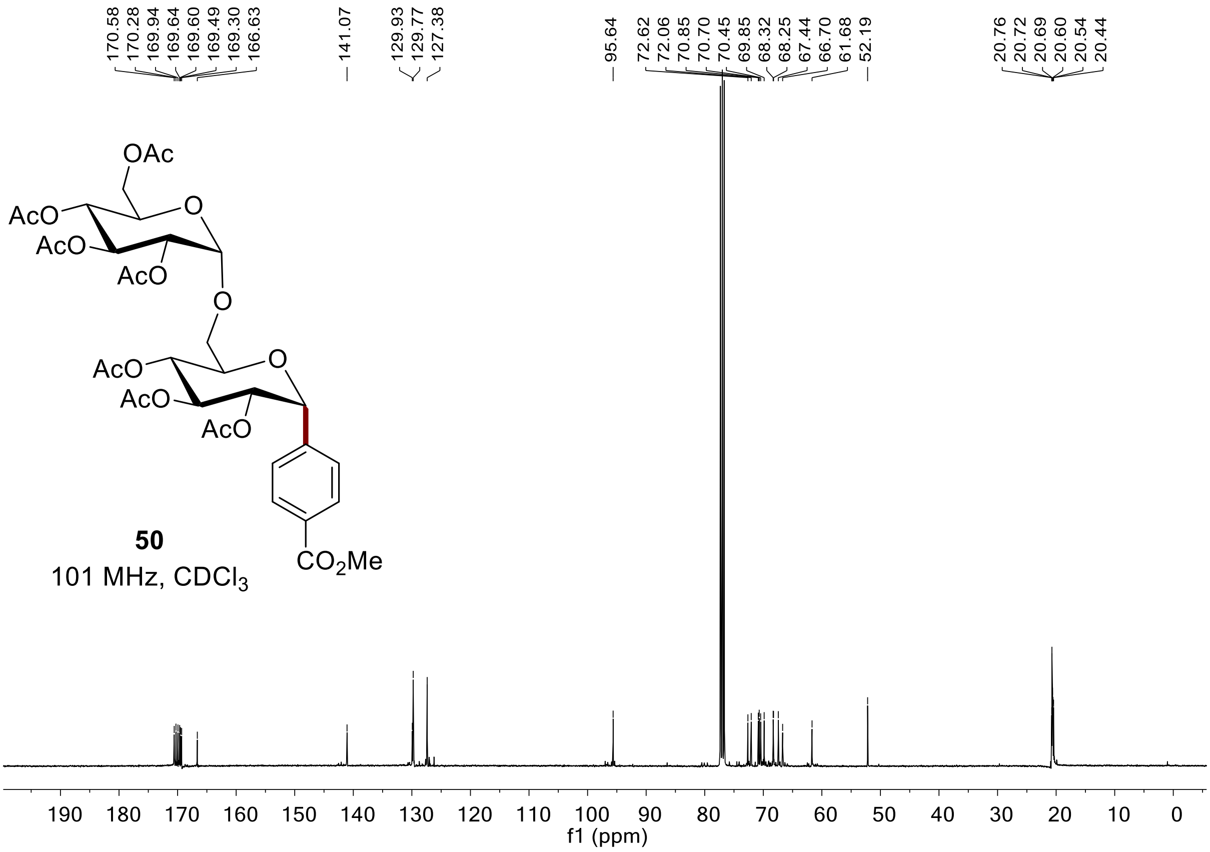
170.58
170.28
169.94
169.64
169.60
169.49
169.30
166.63
141.07
129.93
129.77
127.38
95.64
72.62
72.06
70.85
70.70
70.45
69.85
68.32
68.25
67.44
66.70
61.68
52.19
20.76
20.72
20.69
20.60
20.54
20.44
OAc
AcO
AcO
AcO
O
O
AcO
AcO
AcO
O
CO2Me
50
101 MHz, CDCl3
190 180 170 160 150 140 130 120 110 100 90 80 70 60 50 40 30 20 10 0
f1 (ppm)

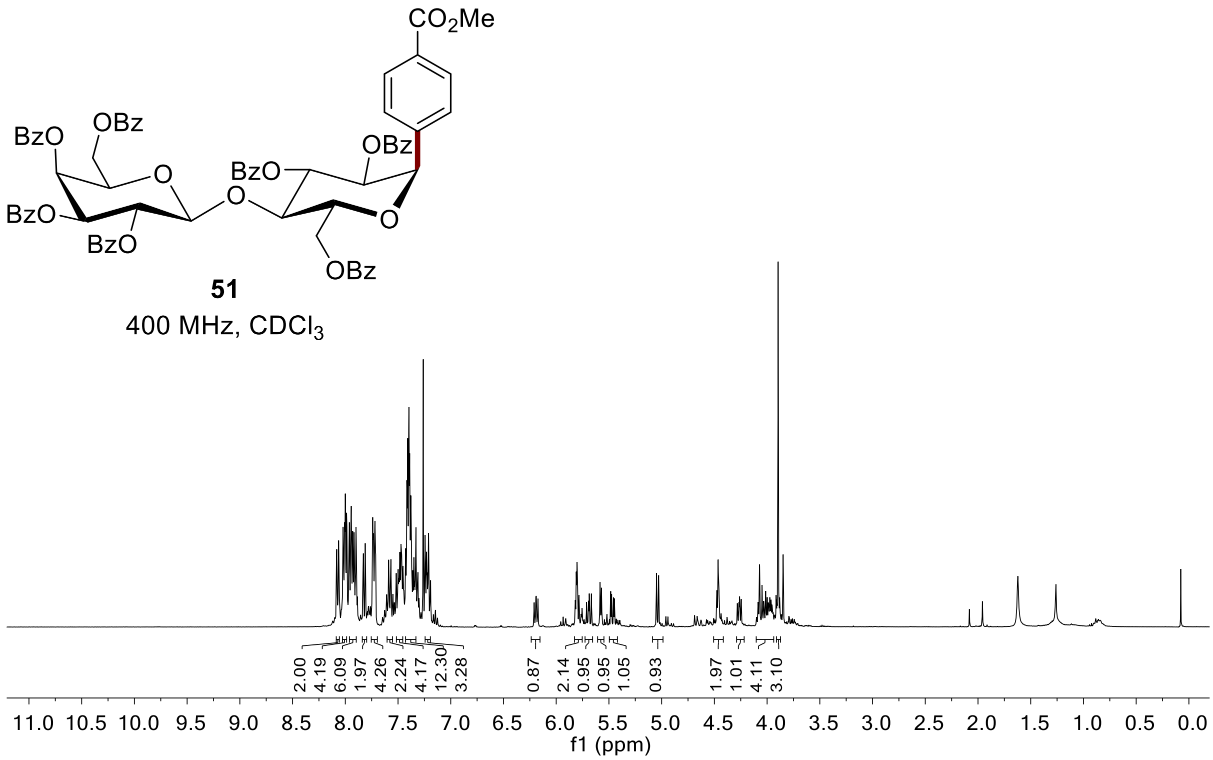
CO$_2$Me
BzO
OBz
BzO
BzO
OBz
BzO
O
O
O
OBz
51
400 MHz, CDCl$_3$
2.00 4.19 6.09 1.97 4.26 2.24 4.17 12.30 3.28 0.87 2.14 0.95 0.95 1.05 0.93 1.97 1.01 4.11 3.10
11.0 10.5 10.0 9.5 9.0 8.5 8.0 7.5 7.0 6.5 6.0 5.5 5.0 4.5 4.0 3.5 3.0 2.5 2.0 1.5 1.0 0.5 0.0
f1 (ppm)

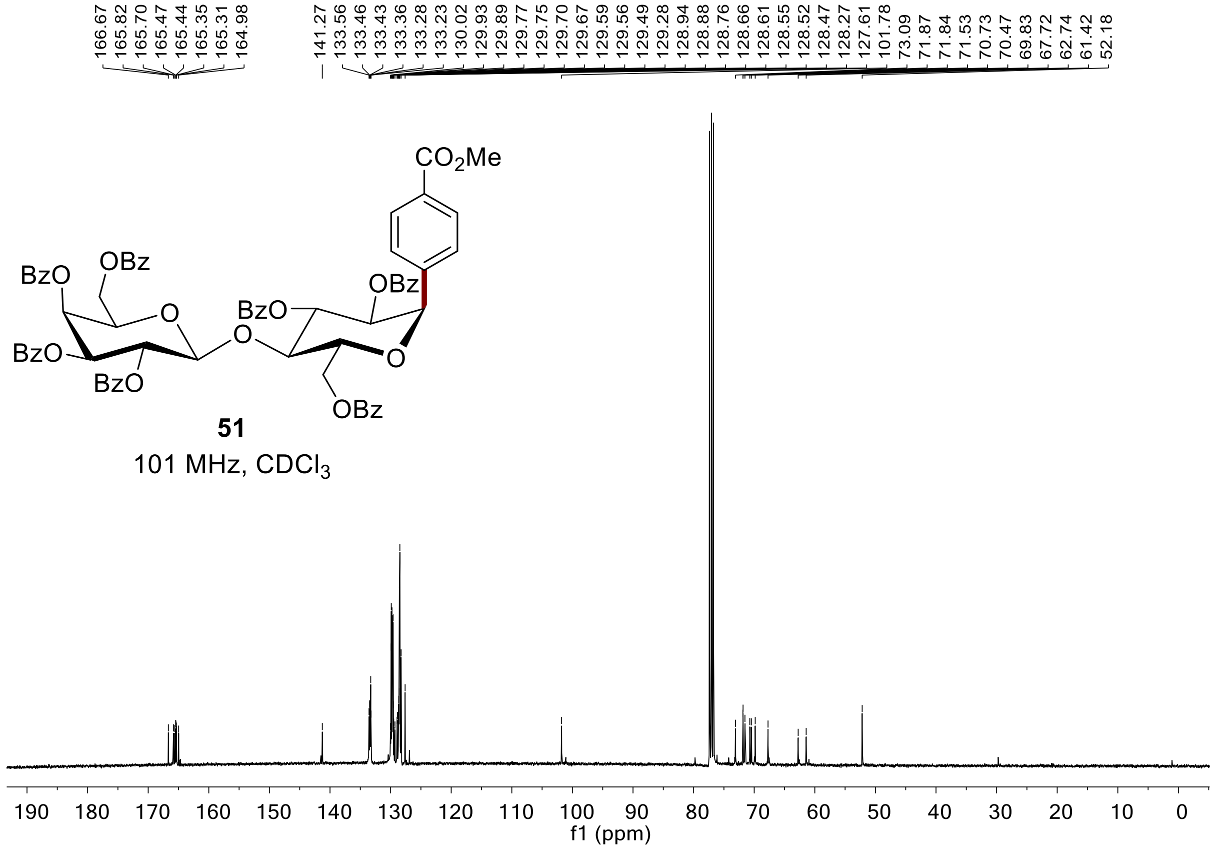
166.67 165.82 165.70 165.47 165.44 165.35 165.31 164.98
141.27 133.56 133.46 133.43 133.36 133.28 133.23 130.02 129.93 129.89 129.77 129.75 129.70 129.67 129.59 129.56 129.49 129.28 128.94 128.88 128.76 128.66 128.61 128.55 128.52 128.47 128.27 127.61 101.78 73.09 71.87 71.84 71.53 70.73 70.47 69.83 67.72 62.74 61.42 52.18
CO$_2$Me
BzO
OBz
BzO
BzO
OBz
BzO
O
O
O
OBz
51
101 MHz, CDCl$_3$
190 180 170 160 150 140 130 120 110 100 90 80 70 60 50 40 30 20 10 0
f1 (ppm)

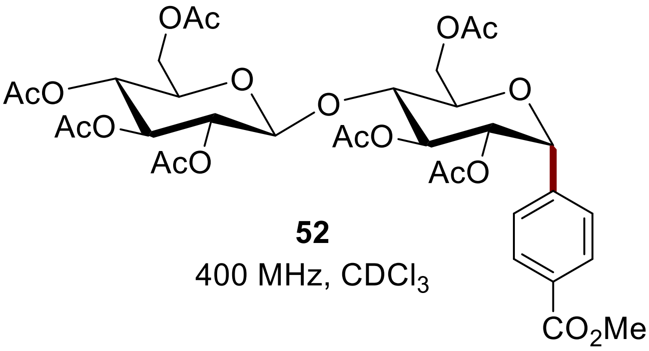

52
400 MHz, $CDCl_3$

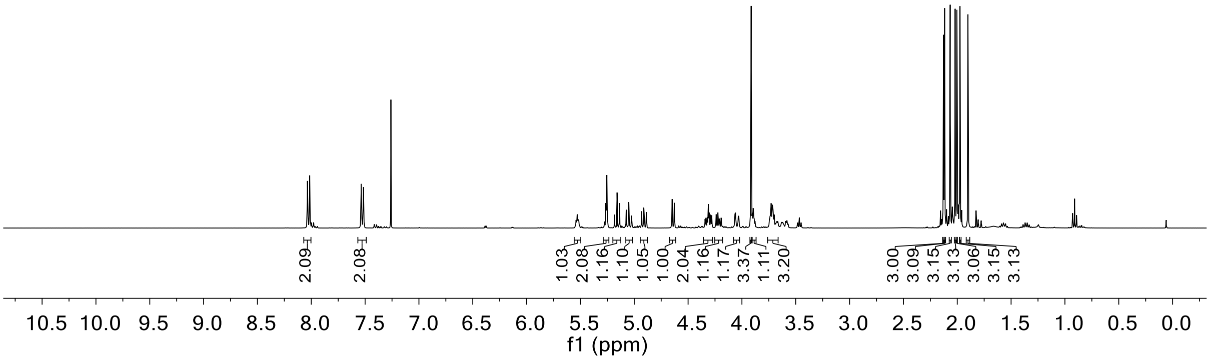

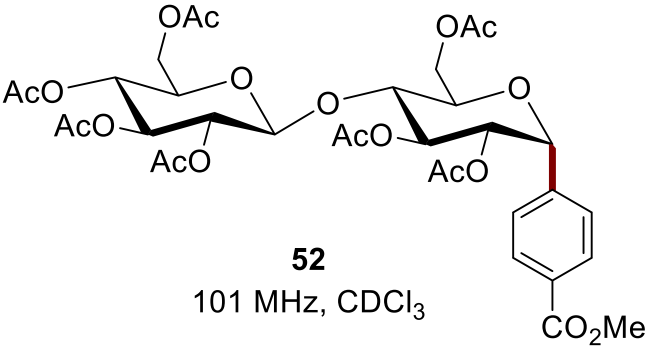

52
101 MHz, $CDCl_3$

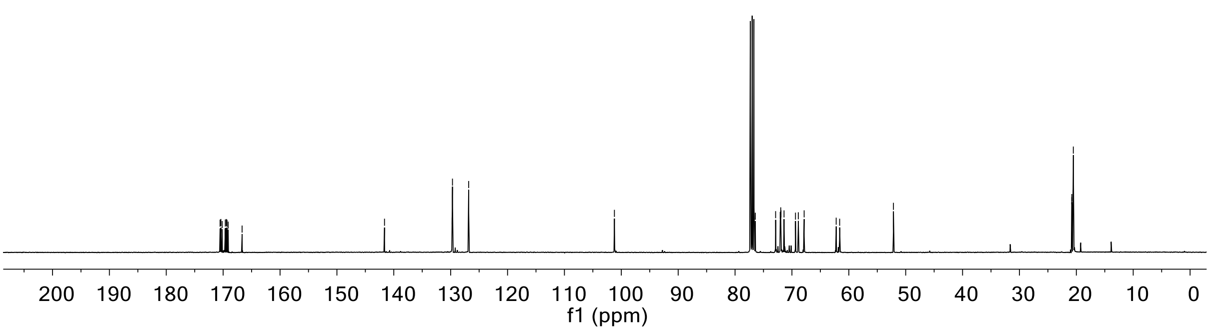

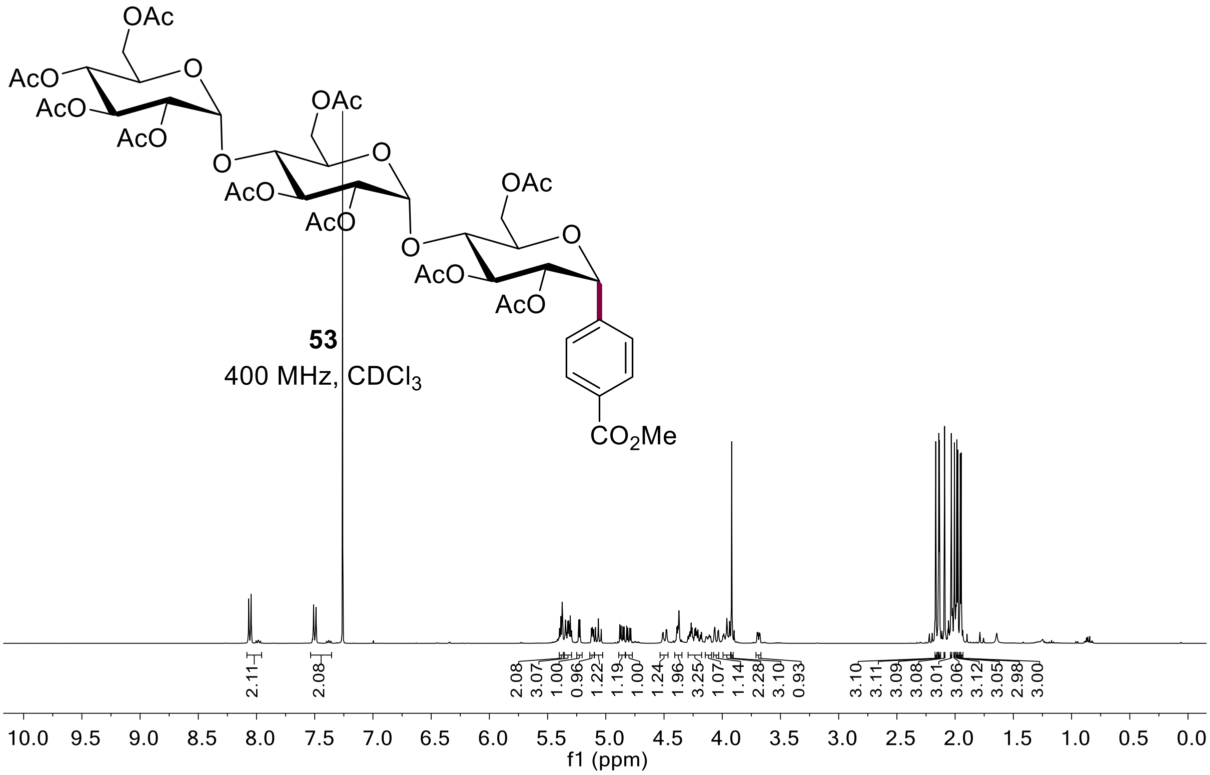
OAc
AcO
AcO
AcO
O
AcO
AcO
CO2Me
53
400 MHz, CDCl3
2.11
2.08
2.08
3.07
1.00
0.96
1.22
1.19
1.00
1.24
1.96
3.25
1.07
1.14
2.28
3.10
0.93
3.10
3.11
3.09
3.08
3.01
3.06
3.12
3.05
2.98
3.00
10.0 9.5 9.0 8.5 8.0 7.5 7.0 6.5 6.0 5.5 5.0 4.5 4.0 3.5 3.0 2.5 2.0 1.5 1.0 0.5 0.0
f1 (ppm)

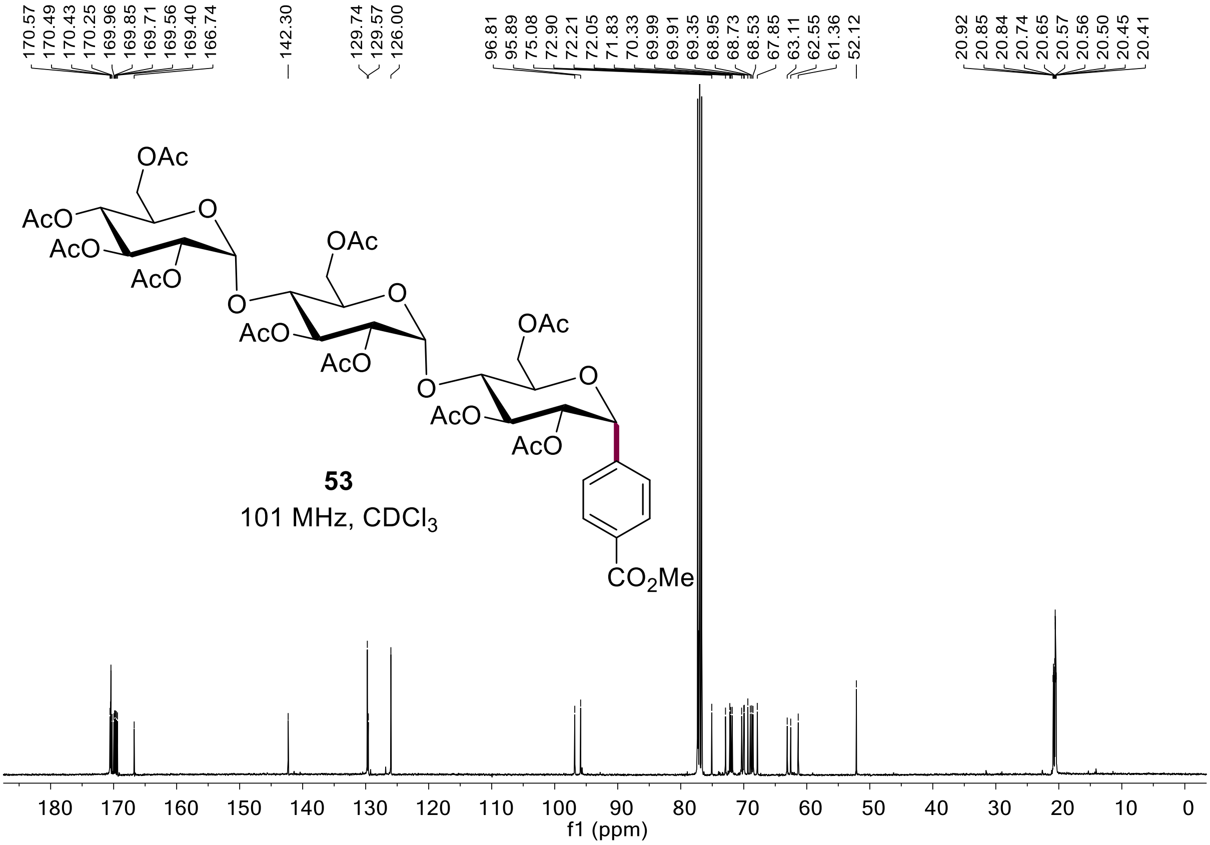
170.57
170.49
170.43
170.25
169.96
169.85
169.71
169.56
169.40
166.74
142.30
129.74
129.57
126.00
96.81
95.89
75.08
72.90
72.21
72.05
71.83
70.33
69.99
69.91
69.35
68.95
68.73
68.53
67.85
63.11
62.55
61.36
52.12
20.92
20.85
20.84
20.74
20.65
20.57
20.56
20.50
20.45
20.41
OAc
AcO
AcO
AcO
O
AcO
AcO
CO2Me
53
101 MHz, CDCl3
180 170 160 150 140 130 120 110 100 90 80 70 60 50 40 30 20 10 0
f1 (ppm)

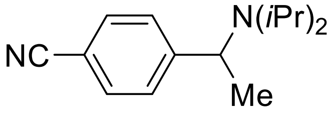

54

300 MHz, $CDCl_3$

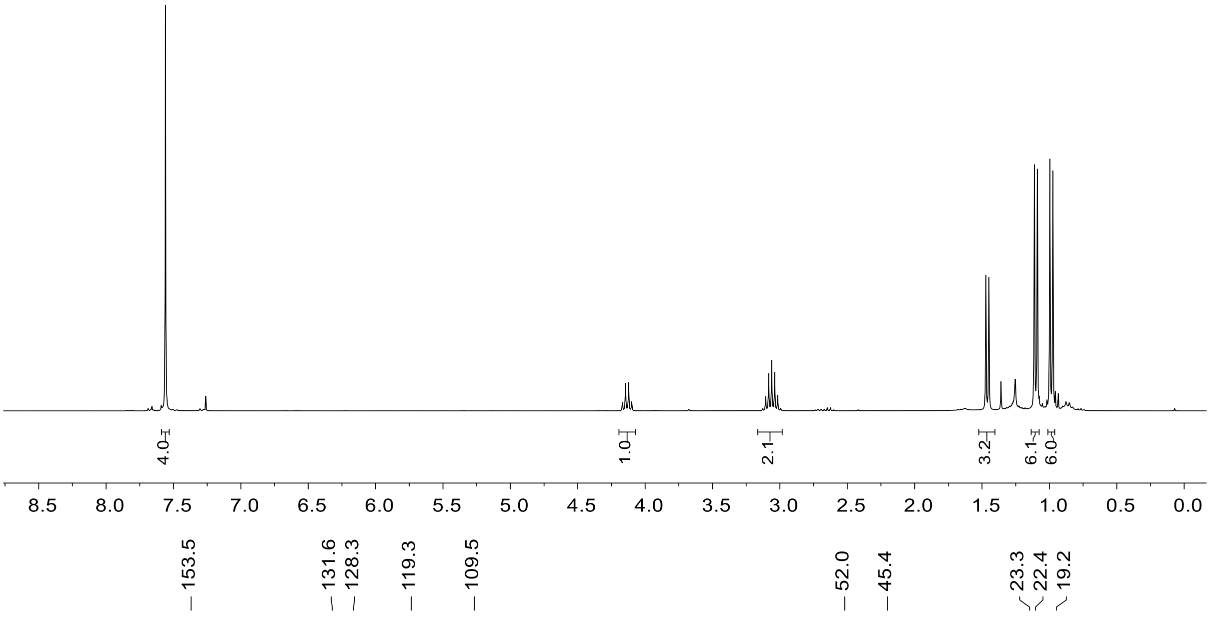

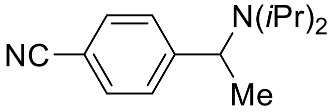

54

75 MHz, $CDCl_3$

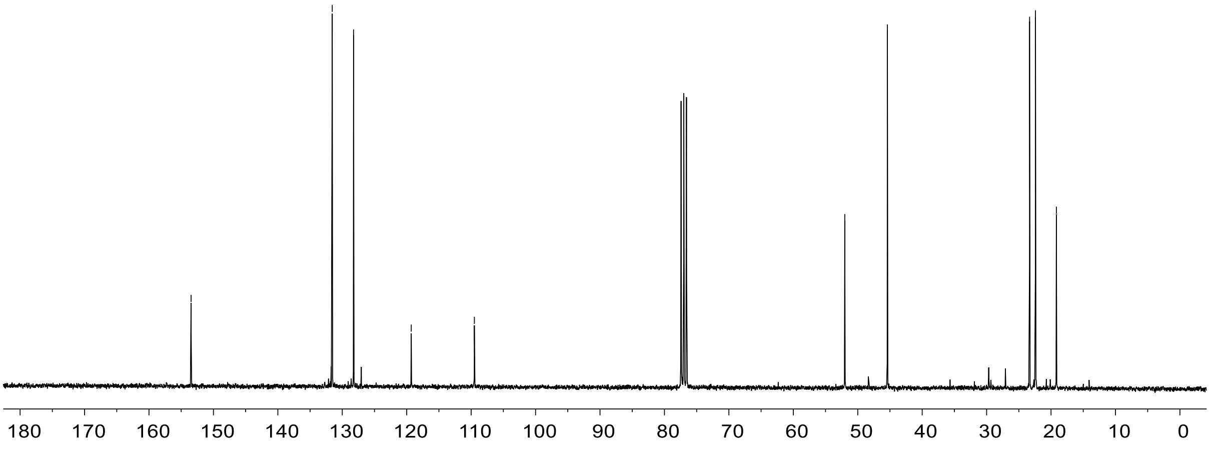

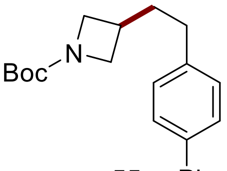

55

400 MHz, $CDCl_3$

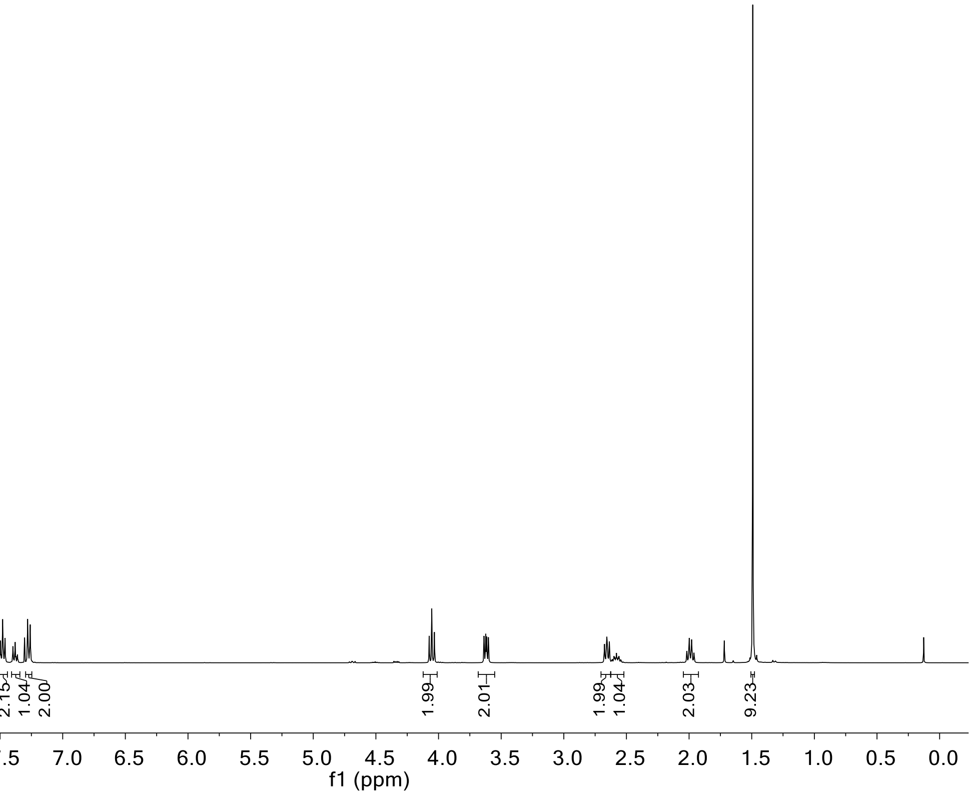

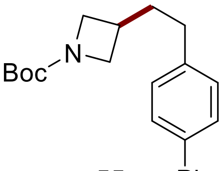

55

101 MHz, $CDCl_3$

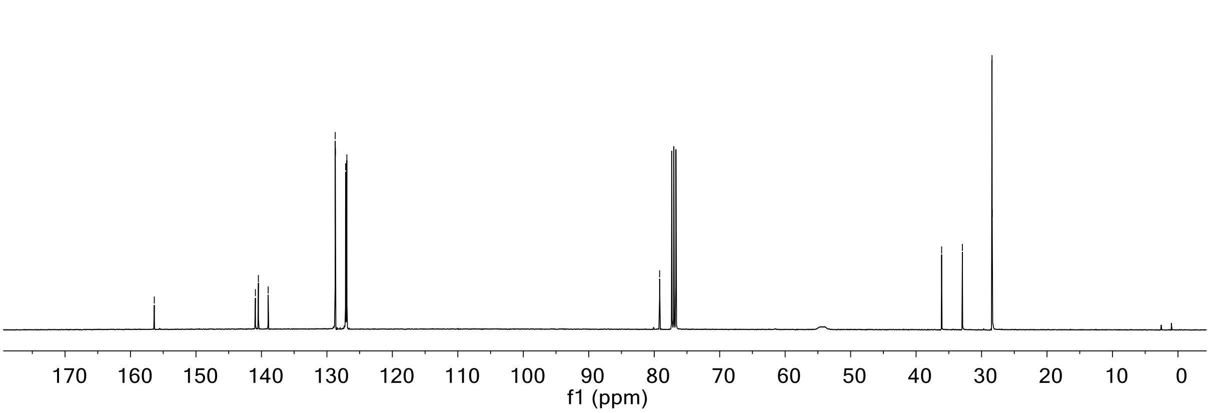

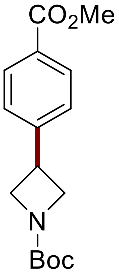

56

300 MHz, $CDCl_3$

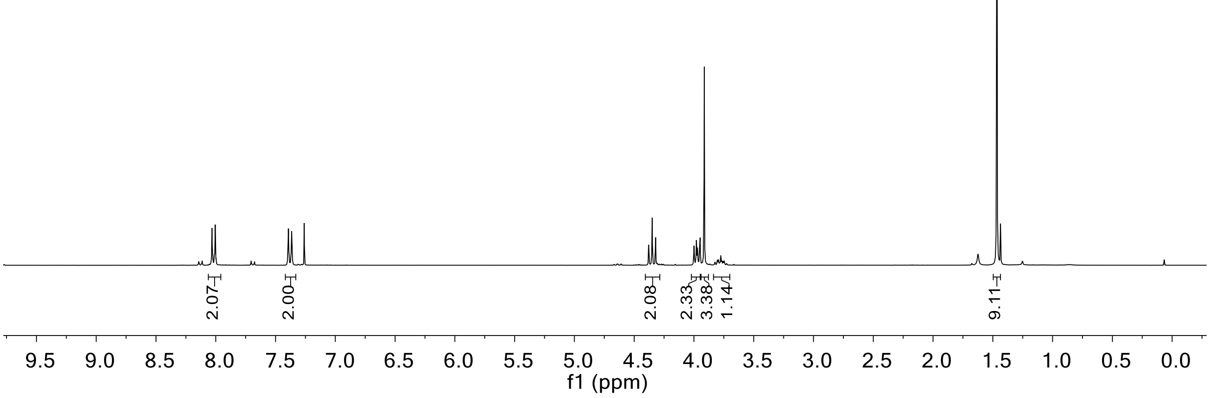

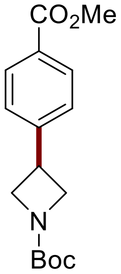

56

75 MHz, $CDCl_3$

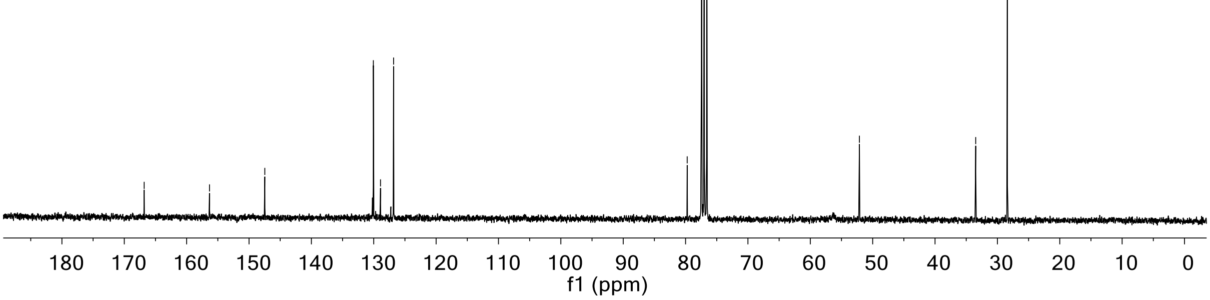

Supplement: Supplementary file 1 — cs4c02322_si_001.pdf [file cs4c02322_si_001.pdf]
